# Building *Streptomyces albus* as a chassis for synthesis of bacterial terpenoids†

**DOI:** 10.1039/d2sc06033g

**Published:** 2023-03-06

**Authors:** Yi Ling Hu, Qi Zhang, Shuang He Liu, Jia Li Sun, Fang Zhou Yin, Zi Ru Wang, Jing Shi, Rui Hua Jiao, Hui Ming Ge

**Affiliations:** a State Key Laboratory of Pharmaceutical Biotechnology, Institute of Functional Biomolecules, Chemistry and Biomedicine Innovation Centre (ChemBIC), School of Life Sciences, Nanjing University Nanjing 210023 China rhjiao@nju.edu.cn hmge@nju.edu.cn

## Abstract

Terpenoids comprise the most chemically and structurally diverse family of natural products. In contrast to the huge numbers of terpenoids discovered from plants and fungi, only a relatively small number of terpenoids were reported from bacteria. Recent genomic data in bacteria suggest that a large number of biosynthetic gene clusters encoding terpenoids remain uncharacterized. In order to enable the functional characterization of terpene synthase and relevant tailoring enzymes, we selected and optimized an expression system based on a *Streptomyces* chassis. Through genome mining, 16 distinct bacterial terpene biosynthetic gene clusters were selected and 13 of them were successfully expressed in the *Streptomyces* chassis, leading to characterization of 11 terpene skeletons including three new ones, representing an ∼80% success rate. In addition, after functional expression of tailoring genes, 18 novel distinct terpenoids were isolated and characterized. This work demonstrates the advantages of a *Streptomyces* chassis which not only enabled the successful production of bacterial terpene synthases, but also enabled functional expression of tailoring genes, especially P450, for terpenoid modification.

## Abstract 2

We built a *Streptomyces* expression system to discovery bacterial terpenoids. Though genome mining and expression of terpene BGCs, 11 terpene skeletons including 3 new ones, and 18 novel distinct terpenoids were isolated and characterized.

## Graphical abstract

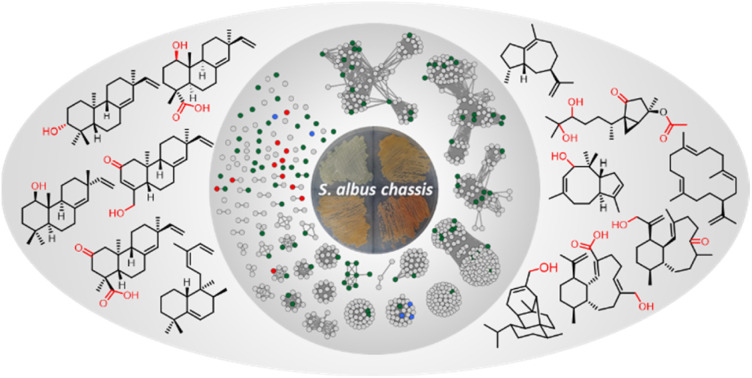

## Introduction

Terpenoids are the largest class of natural products that have attracted considerable interest due to their chemical diversity and potent biological activity.^1–4^ Most terpenoids, which include some of the pharmaceutically important natural products, are produced from plants and fungi.^5–7^ In addition, terpenoids are found in all kingdoms of life including animals, bacteria and archaea,^1^ and fulfil a wide range of essential and specialized functions. Biosynthetically, nature has evolved two ways, the mevalonic acid (MVA) and the methylerythritol 4-phosphate (MEP) pathways to form the basic building blocks of terpenes, isopentenyl diphosphate (IPP) and dimethylallyl diphosphate (DAMPP), which can be fused to form geranyl diphosphate (GPP), farnesyl diphosphate (FPP), geranylgeranyl diphosphate (GGPP), and geranylfarnesyl diphosphate (GFPP), in a head-to-tail manner under the catalysis of prenyl transferases.^8,9^

The key step in terpenoid biosynthesis is the cyclization reaction catalyzed by terpene synthase (TS) that can utilize the acyclic precursors, GPP, FPP, GGPP, GFPP or FFPP, to synthesize structurally diverse terpene skeletons.^10,11^ Based on the initial carbocation formation, TSs can be divided into two classes: type I TS utilizes a tri-magnesium ion cluster to trigger the ionization of isoprenoid diphosphate to yield a cation and inorganic pyrophosphate, and type II TSs protonate a double bond or an epoxide ring to yield a carbocation.^12,13^ After the formation of the terpene skeleton, tailoring enzymes such as cytochrome P450 monooxygenases, flavin-dependent monooxygenases, α-KG dependent dioxygenases, dehydrogenases, and reductases will introduce oxidations and other modifications, further extending the complexity of terpenoids. It is worth noting that these functionalization steps are critical for water-solubility and biological activity, as exemplified by marketed antimalarial drug artemisinin^14^ and anticancer drug taxol (Fig. S1†).^15^

Compared with those of plants and fungi, terpenoids of bacterial origin are relatively rare.^16^ Over the past two decades, the development of genome sequencing technology has accumulated huge amounts of data on bacterial genomes, which prompted us to investigate potential terpenoids in the bacterial genome information.^17^ Bioinformatics analysis combined with a convenient and effective expression system for genes of TSs and other tailoring enzymes will be able to efficiently discover novel terpenoids of bacterial origin.^18^

*Escherichia coli* and yeast are the most common chassis used for heterologous production of terpenoids. Easy genetic manipulation, low cost, and rapid growth make them outstanding hosts for many natural products with high value.^19–22^ However, we consider *Streptomyces* (belonging to actinobacteria) as a more suitable chassis for bacterial terpenoid production because of three reasons. Firstly, a statistical study on biosynthetic gene clusters (BGCs) showed that *Streptomyces* is the most abundant genus of gene cluster families (GCFs) in bacteria,^23^ and its ability to produce abundant natural products reflects the sufficient supply of precursors in the cell, which can eliminate the complicated pathway engineering of precursor biosynthesis. Secondly, sophisticated gene editing systems have been established in some model *Streptomyces* species after almost half a century of intensive research.^24^ Moreover, the proper functions of post-modification enzymes encoded in terpenoid clusters usually require corresponding coenzymes or cofactors. For example, P450s are some of the most exquisite and versatile tailoring enzymes, whose function usually requires native or compatible redox protein partners, ferrodoxin and ferrodoxin reductase.^25^ However, these accessory proteins do not exist in *E. coli* or yeast. Thus, in this work we selected *Streptomyces* strain as a chassis to discover new bacterial terpenoids through the genome mining strategy.

## Results and discussion

### Evaluation and optimization of a *Streptomyces* expression system

In order to conveniently express terpenoid BGCs, four popular *Streptomyces* strains with rapid growth and sophisticated genetic manipulation were chosen as the candidates of the chassis: *S. coelicolor* M1154, *S. lividans* SBT18, *S. albus* J1074 and *S. chartreusis* 1018.^26^ We used the production of lycopene, a tetraterpene with red color, as a marker to evaluate the chassis's ability to supply terpene precursors. Three key enzymes were involved in lycopene biosynthesis:^27^ GGPP synthase (CrtE) that can convert endogenous IPP and DMAPP into GGPP, phytoene synthase (CrtB) that catalyses the head-to-head condensation of two GGPPs to phytoene, and phytoene desaturase (CrtI) that extends the double bond conjugation of the C40 backbone to form lycopene with bright red color.

**Fig. 1:**
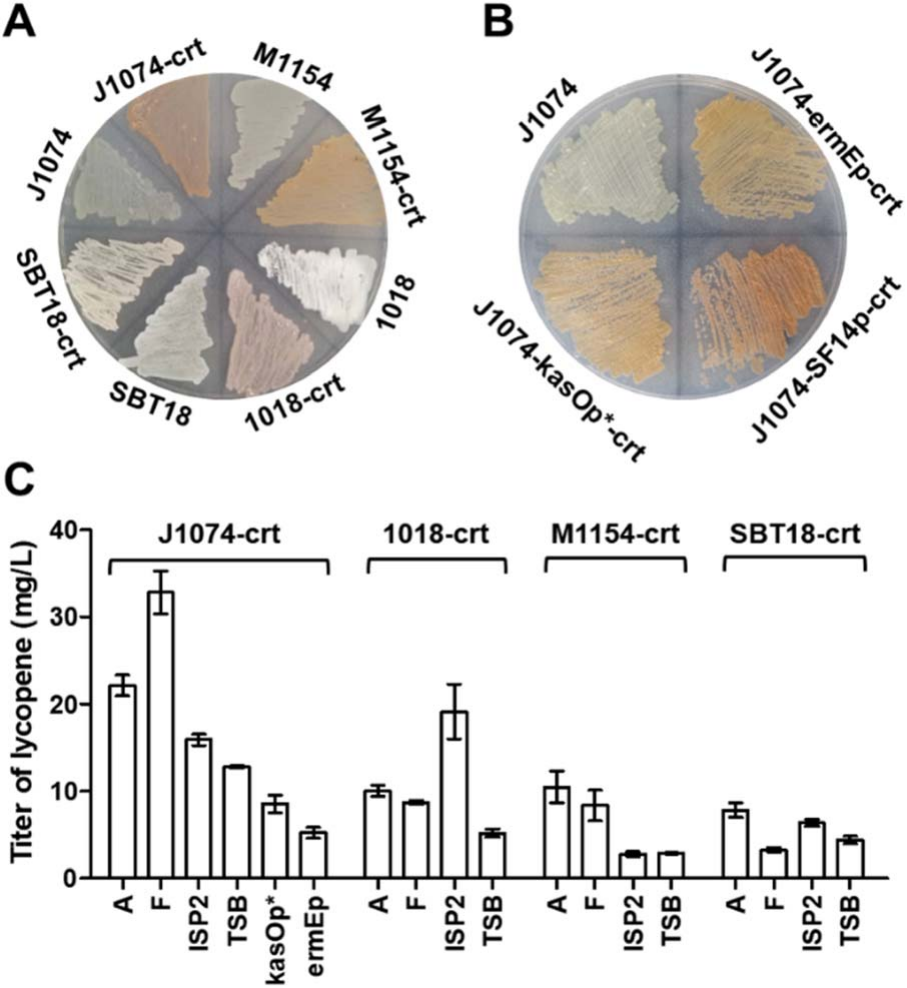
Lycopene gene cluster expressed in *Streptomyces*. (A) A ISP4 plate showing the color of four *Streptomyces* integrated lycopene gene cluster, M1154: *S. coelicolor* M1154, SBT18: *S. lividans* SBT18, 1018: *S. chartreusis* 1018, and J1074: *S. albus* J1074. (B) A ISP4 plate showing the color of *S. albus* J1074 containing *crt* driven by different promoters. (C) The bar chart showing the titer of lycopene produced by *Streptomyces* containing the *crt* cluster and cultured with different media.

To this end, we used a plasmid pSOK804 (ref. 28) containing a VWB integration system to clone the lycopene BGC (*crt*) under the control of constitutive promoter SF14p.^29^ The resulting plasmid was integrated into four strains by conjugation. On the ISP4 plate, we found that after the introduction of *crt*, all four strains had obvious pigment accumulation, especially *S. albus* J1074 (Fig. 1A). To test the actual productivity of these strains, four liquid media, including A, F, ISP2 and TSB, usually used in the literature for natural product production were chosen. After six days of fermentation, the titers of lycopene in these strains were observed (Fig. S2A†) and calibrated by analytical HPLC (Fig. 1C and S2B†). It was found that the *S. albus* J1074 recombinant strain containing *crt* had the highest titer of lycopene in F medium after fermentation for 6 days. Notably, after 6 days of cultivation, *S. albus* J1074 strain has the ability of autolysis (Fig. S2D†). This property could be very convenient and useful for future terpenoid extraction as the low polarity terpenoids are often accumulated in the cell membrane. Thus, the processing for the collection and disruption of cell pellets can be avoided.

The promoter plays a critical role in successful transcription of genes in a heterologous host.^30^ To evaluate the effect of different promoters, we selected two other common constitutive promoters in *Streptomyces*: *ermE*p^31^ and *kasO*p*.^32^ Based on the color of the plate (Fig. 1B) and productions of lycopene (Fig. 1C), it was found that the SF14p promoter resulted in a superior performance with the titer of lycopene at 32.8 ± 2.4 mg L^−1^ (Table S1†), which was 3 to 5-fold higher than that of the other strains. This titer, we thought, could be sufficient for structural elucidation of novel terpenoids.

With this result in hand, we realized that *Streptomyces albus* could already supply sufficient terpenoid precursors without further optimization of the metabolic pathway. Considering that not all terpene BGCs contain FPP synthase or GGPP synthase, we used the plasmid pSET152 (ref. 33) to integrate *crtE* (GGPP synthase) and *ptlB* (FPP synthase) genes,^34^ both of which are under the control of the SF14p promoter, into the wild-type *S. albus* J1074, generating a chassis strain *S. albus* J1074M.

### Genome mining of terpene BGCs

We then intended to mine terpene BGCs from the bacterial genome database. As of yet, it is still very challenging to establish a relationship between the sequences of TSs with their corresponding products. Moreover, the homology similarities between TSs are very low, making it hard to discover potential TSs by using the BLAST algorithm. The protein domain family database (Pfam) searching method based on the use of a hidden Markov model (HMM) can identify previously unrecognized bacterial TSs more effectively.^18^ There are two Pfam items of bacterially derived type I TSs: PF03936 and PF19086. To evaluate whether these two models can be used for TS excavation, a characterized TS set (Table S2†), which included type II TSs and UbiA-related diterpene synthases, was chosen as a test sample. After searching with default parameters, we found that model PF19086 could cover type I TSs very well, and it does not hit type II TSs and UbiA-related diterpene synthases. So we used model PF19086 to search the proteomes of 282 actinobacterial microorganisms, which resulted in obtaining 756 type I TSs.

We constructed a sequence similarity network (SSN) containing 756 recognized items and known sequences to visualize the similarity between uncharacterized sequences and known sequences, which could facilitate the identification of novel TSs. When an *E* value threshold 10^−60^ was applied, two clades for 2-methylisoborneol (2-MIB) synthase and geosmin synthase were very well separated in the network.

**Fig. 2:**
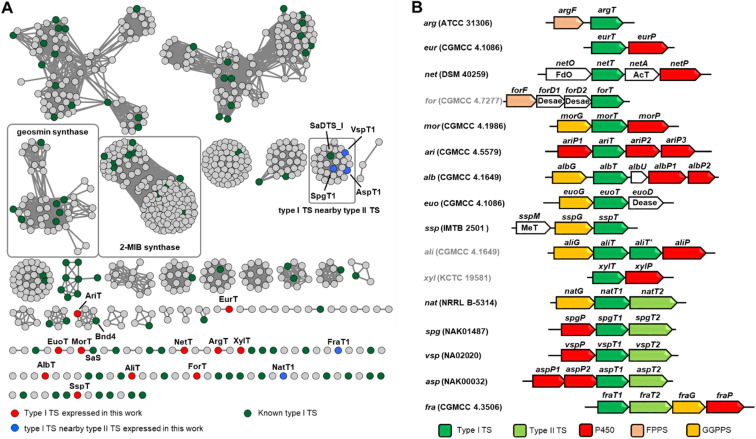
Genome mining of terpenoid BGCs. (A) SSN analysis of recognized and known type I TS with an *E*-value threshold: 10^−60^ and visualized using Cytoscape. (B) The schematic diagram of BGCs selected from SSN analysis. FdO: FAD-dependent oxidoreductase, AcT: acyltransferase, Dease: desaturase, and MeT: methyltransferase.

Our attention was then focused on the clades that are separated from known sequences (green color in Fig. 2A). In order to obtain more decorated terpene molecules, we selected BGCs preferentially so that TS genes were clustered with genes encoding post-tailoring modifications. Finally, we selected 11 terpene BGCs containing only type I TS (red color in Fig. 2A) and 5 BGCs containing type II TS (blue color in Fig. 2A) to test the effectiveness and tolerance of the *Streptomyces* expression system (Fig. 2B).

### Heterologous expression of TSs

We individually cloned all TS genes into the pSOK804 plasmid under the control of the SF14p promoter and expressed them in *S. albus* J1074M (Table S4†). After 6 days of cultivation, TLC analysis of organic extract from 16 constructs showed that 13 constructs had positive results (Fig. S3†). To determine these structures unambiguously, we carried out a 5 L scale fermentation and isolated all products in a sufficient material for NMR analysis.

**Fig. 3:**
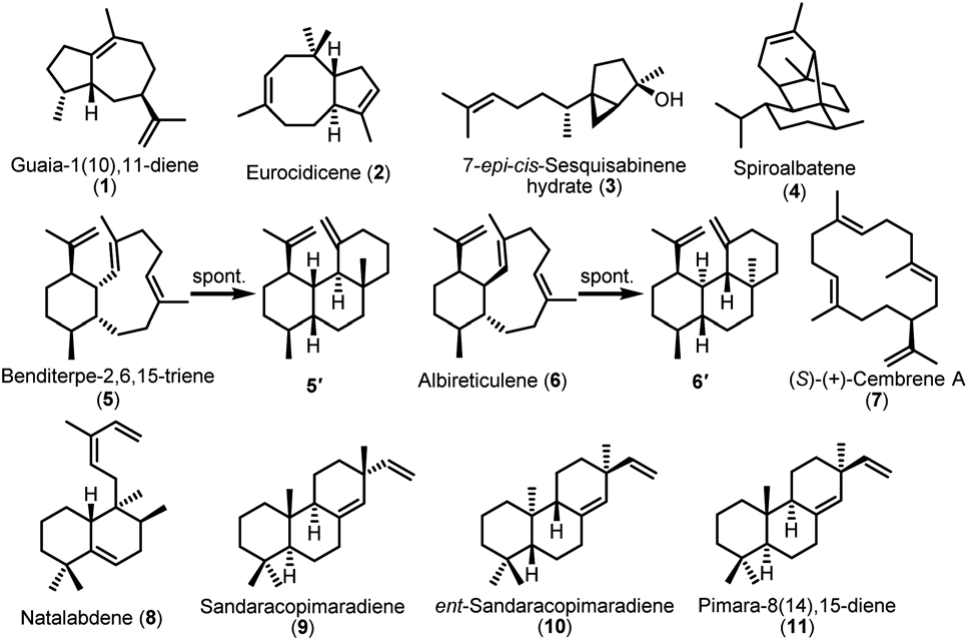
The structures of identified sesquiterpenes and diterpenes produced by heterologous expression of TS genes in *S. albus* J1074M.

Compound 1, purified from the crude extract of *S. albus* strain carrying the TS *argT* gene from strain *Nocardia argentinensis* ATCC 31306 was determined to be a sesquiterpene guaia-1(10),11-diene.^35^1 has previously only been reported as a product of TS GhTPS2 from cotton.^36^ However, ArgT and GhTPS2 showed no sequence homology based on the BLASTp analysis. When we introduced the *eurT* gene from *S. eurocidicus* CGMCC 4.1086 into the *S. albus* J1074M chassis, a new product 2 with *m*/*z* 204.2 corresponding to a molecular formula of C_15_H_24_ was observed in the GC-MS analysis. Compound 2, named eurocidicene, is elucidated as a new *trans*-8/5-bicyclic sesquiterpene based on extensive NMR analysis (Table S6†). The TS gene *netT* from *S. netropsis* DSM 40259 produced an oily substance 3 with strong fragrance. Compound 3 was then identified as a 5/3-bicyclic sesquiterpene 7-*epi-cis*-sesquisabinene hydrate,^37^ a common component of plant essential oils,^38,39^ whose biosynthesis has never been studied so far. The bacterial enzyme NetT is the first reported sesquiterpene synthase responsible for the formation of 3. MorT shares 66% sequence identity with known spiroalbatene synthase (SaS) that can produce spiroalbatene (4).^40^ Expression of *morT* from *S. morookaensis* CGMCC 4.1986 in the chassis indeed produced 4 according to GC-MS, optical rotation and NMR analysis (Fig. 3).

The TS AriT from the *ari* cluster (*Amycolatopsis arida* CGMCC 4.5579) was clustered with an already characterized TS Bnd4 in SSN analysis (Fig. 2A). As expected, it produced a structurally dynamic 6/10-bicyclic diterpene, benditerpe-2,6,15-triene (5) with a *cis*-eunicellane skeleton.^41^ Moreover, we observed that 5 could slowly convert to a 6/6/6-tricyclic framework product 5′ in chloroform (Table S7†). Interesting, a TS AlbT from the *alb* cluster (*S. albireticuli* CGMCC 4.1649) showing 22% sequence identity to AriT, produced a product that could also be converted in chloroform to a 6/6/6-tricyclic framework product 6′ (Table S8†), which has the same planar structure with 5′. The configuration difference between 5′ and 6′ suggests that 5 is an stereoepimer of 6. Indeed, the ^1^H NMR spectrum and ^13^C NMR of 6 also gave a set of broad signals in C_6_D_6_, suggesting that 6 also has a dynamic structure nature. Based on 1D and 2D NMR analysis, the structure of 6 (named albireticulene)^42^ was determined as a *trans*-6/10-bicyclic eunicellane skeleton (Table S9†). Moreover, EuoT (*S. eurocidicus* CGMCC 4.1086) and SspT (*S.* sp. IMTB 2501) could produce the same diterpene (*S*)-(+)-cembrene A (7)^40^ based on the analysis of their NMR data and optical rotation values. Notably, the reported bacterial (*S*)-(+)-cembrene A synthase (CAS)^40^ has only 26% sequence identity to EuoT, but no detectable identity to SspT. Unfortunately, we could not observe the products upon individual expression of four type I TS genes, *forT*, *aliT*, *aliT′* and *xylT* in *S. albus* J1074M.

After successfully obtaining 7 terpenes from 12 Type I TS, we then turned to co-express the identified five type I and type II TS clusters in *S. albus* J1074M. Gratifyingly, we successfully expressed all these genes in *S. albus* J1074M based on TLC analysis (Fig. S3†). When a construct containing *natT1* and *natT2* was cloned from *S. natalensis* NRRL B-5314 and integrated into the *S. albus* J1074M chassis, a novel labdane-related diterpene 8, named natalabdene, was isolated and characterized (Table S10†). The type I and type II TS genes from the *spg* cluster (*Streptosporangium* sp. NAK01487) and *vsp* cluster (*Verrucosispora* sp. NA02020) could produce 9 and 10, respectively. Though their NMR data were identical to each other, the optical rotation of 9 ([*α*]^25^_D_ = −20.5 (*c* 0.7, CHCl_3_)) was almost opposite to that of 10 ([*α*]^25^_D_ = +16.1 (*c* 0.6, CHCl_3_)), indicating that 9 is sandaracopimaradiene^43^ and 10 is *ent*-sandaracopimaradiene.^44^ Co-expression of *aspT1* and *aspT2* from *Actinomadura* sp. NAK0032 led to formation of a known diterpene pimara-8(14),15-diene (11).^45^ By combination expression of type I and II TS genes, *fraT1* and *fraT2*, the product we determined was also *ent*-sandaracopimaradiene (10). Taken together, the expression of 13 out of 16 clusters represented an ∼80% success rate, indicating that *S. albus* J1074M is a suitable chassis for bacterial terpene production.

### Heterologous expression of intact terpene BGCs

We then aim to co-express the genes for tailoring modification together with TSs. New spots were observed on TLC analysis from expression of 9 of 11 BGCs (Fig. S4†). Following co-expression of P450 *eurP* and TS *eurT* in *S. albus* J1074M, a novel terpenoid 2a was purified, which has a *m*/*z* 220.2 ion signal on the GC-MS spectrum. According to comparative NMR analysis, we found an additional hydroxyl group at the C-1 position (Table S11†).

When the intact *net* gene cluster was expressed, we observed the production of 3a (*m*/*z* 313.2012 [M + H]^+^) and 3b (*m*/*z* 313.2010 [M + H]^+^). 3a and 3b (Tables S12 and S13†) were structurally determined to be a pair of diastereoisomers with the same planar structures. To assign the function of three tailoring enzymes, the corresponding encoding genes were individually expressed with NetT (TS). When *netO* was co-expressed with *netT*, a pair of diastereoisomers 3c/3d were produced and identified as dihydroxyl derivatives of 3 (Table S14†). In contrast, co-expression of *netT* and *netP* gave 3e (Table S15†). However, we did not observe any new product in TLC analysis when the acyltransferase gene (*netA*) was introduced individually (Fig. S5†). Thus, we concluded that NetO could act on the C10/C13 alkene group of the terpene skeleton to give an epoxide, which could undergo spontaneous hydrolysis to generate 3c/3d, and NetP would oxidize C-5 the inactivated C15 position to give a ketone group. In addition, the 13-OH group in 3e could be generated by NetP or endogenous oxidase in the heterologous host.

A novel diterpenoid 4a (Table S16†), which has a hydroxyl group at C20 at 4, was purified when P450 gene *cinP* was co-expressed with spiroalbatene synthase *cinT*. After introducing the intact *ari* cluster into *S. albus* J1074M, 5a was characterized (Table S17†). Expression of the intact *alb* cluster led to the production of 6a and 6b (Tables S18 and S19†). Unexpectedly, we found that only AlbP1 is sufficient for dual oxidation of C-6 and C-17 positions as co-expression of *albT* and *albP1* could give 6a and 6b, while no new products could be observed when *albP2* was co-expressed with *albT* (Fig. S5†).

For the expression of five intact BGCs containing type II TS, more structurally diverse products were obtained. Specifically, after addition of P450 gene *spgP* into the *spgT1* and *spgT2* constructs, we got a product 9a (Table S20†) with an additional β-hydroxylation at C12. When the P450 gene, *vspP*, in cluster *vsp* was added in the expression construct, a series of oxidation products, **10a–10d**, were obtained and structurally characterized (Tables S21–S24†). As only one P450 (VspP) is encoded in the *vsp* cluster, we reasoned that VspP has a versatile function and could successively catalyze oxidation of C-13, C-16, and C-17 and desaturate the C-14/C-15 position.

Expression of the intact *asp* cluster led to the production of two major compounds 11a (*m*/*z* 287.2368 [M–H_2_O + H]^+^) and 11b (*m*/*z* 301.2168 [M–H_2_O + H]^+^). While co-expression of *aspP2* with *aspT1* and *aspT2* led to the yield of a hydroxylation product 11c (Table S27†), no new products were detected when *aspP1* was expressed with *aspT1* and *aspT2* (Fig. S5†). These results strongly suggested that AspP2 can hydroxylate the C-12 position, and then AspP1 oxidizes the C-17 methyl group to give 11a and 11b.

**Fig. 4:**
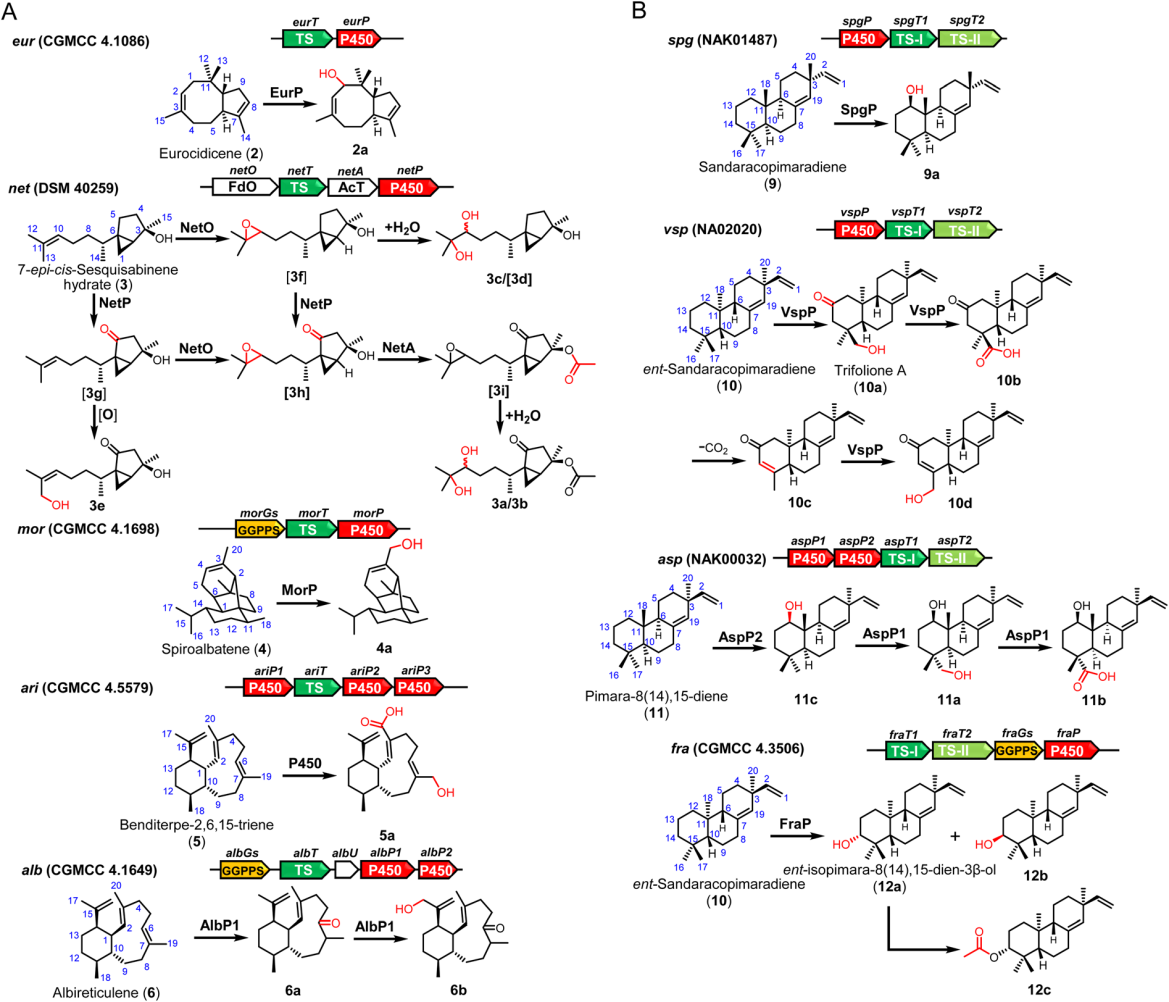
The structures of terpenoids produced by heterologous expression of terpenoid BGCs in *S. albus* J1074M.

Finally, the intact *asp* cluster produced a known compound 12a and two novel diterpenes 12b and 12c (Tables S28 and S29†). The biosynthetic role of FraP could be C-3 oxidase. As no acetyltransferase is present in the *fra* cluster, we surmised that the acetyl group in 12c could be introduced by endogenous enzymes in the chassis (Fig. 4).

## Conclusions

In summary, using the HMM searching method, we identified 756 potential TSs from 282 bacteria genomes and selected 16 terpenoid BGCs based on SSN analysis of TSs. We have successfully obtained 13 distinct terpenes from 13 gene clusters in our optimized *S. albus* J1074M expression system. Among these, we obtained 11 terpene products, including a new sesquiterpene (2) and two new diterpenes (6 and 8). In addition, 1 and 3 were the first reports of terpenes of bacterial origin.

Post-tailoring modification plays an important role in expanding the structural diversity of terpenoids. In our cases, after co-expression of TS genes with their corresponding tailoring-modification genes, 18 new terpenoids with different oxidation states were isolated and characterized. We can find that the *S. albus* chassis has a significant advantage not only in providing sufficient precursors for terpenoid biosynthesis, but also in efficiently expressing post-tailoring modification genes. So far, characterized terpenoids are still relatively rare in bacteria in comparison to plants and fungi. Overall, using the *S. albus* chassis combined with bioinformatics analysis will be an ideal way to explore novel terpenoids of bacterial origin.

## Data availability

The experimental datasets and spectra supporting this article have been uploaded as a part of ESI.†

## Author contributions

H. M. G. and R. H. J. conceived and designed the project. Y. L. H., Q. Z., S. H. L., J. L. S., F. Z. Y., Z. R. W. and J. S. performed the experiments and analysed the data. H. M. G. and Y. L. H. wrote the manuscript. All authors discussed the results and commented on the manuscript.

## Conflicts of interest

The authors declare no conflict of interest.

## Supplementary Material

SC-014-D2SC06033G-s001
