# Supplementary material for: Building Streptomyces albus as a chassis for synthesis of bacterial terpenoids
Source: Chem Sci. 2023 Mar 6;14(13):3661–7. doi: 10.1039/d2sc06033g (PMC10055836; doi:10.1039/d2sc06033g)
Supplement: SC-014-D2SC06033G-s001 [file SC-014-D2SC06033G-s001.pdf]

Electronic Supplementary Material (ESI) for Chemical Science.
This journal is © The Royal Society of Chemistry 2023

Supporting Information

Building *Streptomyces albus* as a Chassis for Synthesis of Diverse Bacterial Terpenoids

Yi Ling Hu‡, Qi Zhang‡, Shuang He Liu, Jia Li Sun, Fang Zhou Yin, Zi Ru Wang, Jing Shi, Rui Hua Jiao*, Hui Ming Ge*

State Key Laboratory of Pharmaceutical Biotechnology, Institute of Functional Biomolecules, Chemistry and Biomedicine Innovation Center (ChemBIC), School of Life Sciences, Nanjing University, Nanjing, 210023 China. rhjiao@nju.edu.cn, hmge@nju.edu.cn.

‡ These authors contribute equally to this work.

CONTANTS

I. Experimental Procedures: 6

DNA manipulation and plasmid construction. 6

Culture conditions of recombinant strains. 6

Extraction and HPLC analysis of lycopene. 7

Sequence similarity network (SSN) of terpene synthases. 7

Isolation and analysis of terpenoids. 7

II. Supplementary Tables: 8

Table S1 The titer of lycopene produced by recombinant strains in different conditions. 8

Table S2 Characterized bacterial terpene synthases and the e-value of HMM model search. 8

Table S3 Genbank number of terpenoids biosynthetic genes were mentioned in this study. 11

Table S4 Plasmids were achieved in this study. 12

Table S5 Primers and oligonucleotides were used in this study. 13

Table S6 ^{1}H NMR (400 MHz) and ^{13}C NMR (100 MHz) assignments for **2** in $CDCl_3$. 16

Table S7 ^{1}H NMR (400 MHz) and ^{13}C NMR (100 MHz) assignments for **5′** in $CDCl_3$. 17

Table S8 ^{1}H NMR (400 MHz) and ^{13}C NMR (100 MHz) assignments for **6′** in $CDCl_3$. 18

Table S9 ^{1}H NMR (400 MHz) and ^{13}C NMR (100 MHz) assignments for **6** in C_6D_6. 19

Table S10 ^{1}H NMR (400 MHz) and ^{13}C NMR (100 MHz) assignments for **8** in $CDCl_3$. 20

Table S11 ^{1}H NMR (400 MHz) and ^{13}C NMR (100 MHz) assignments for **2a** in $CDCl_3$. 21

Table S12 ^{1}H NMR (400 MHz) and ^{13}C NMR (100 MHz) assignments for **3a** in methanol-d_4. 22

Table S13 ^{1}H NMR (400 MHz) and ^{13}C NMR (100 MHz) assignments for **3b** in methanol-d_4. 23

Table S14 ^{1}H NMR (400 MHz) and ^{13}C NMR (100 MHz) assignments for **3c** in methanol-d_4. 24

Table S15 ^{1}H NMR (400 MHz) and ^{13}C NMR (100 MHz) assignments for **3e** in methanol-d_4. 25

Table S16 ^{1}H NMR (400 MHz) and ^{13}C NMR (100 MHz) assignments for **4a** in $CDCl_3$. 26

Table S17 ^{1}H NMR (400 MHz) and ^{13}C NMR (100 MHz) assignments for **5a** in $CDCl_3$. 27

Table S18 ^{1}H NMR (400 MHz) and ^{13}C NMR (100 MHz) assignments for **6a** in $CDCl_3$. 28

Table S19 ^{1}H NMR (400 MHz) and ^{13}C NMR (100 MHz) assignments for **6b** in $CDCl_3$. 29

Table S20 ^{1}H NMR (400 MHz) and ^{13}C NMR (100 MHz) assignments for **9a** in $CDCl_3$. 30

Table S21 ^{1}H NMR (400 MHz) and ^{13}C NMR (100 MHz) assignments for **10a** in $CDCl_3$. 31

Table S22 ^{1}H NMR (400 MHz) and ^{13}C NMR (100 MHz) assignments for **10b** in $CDCl_3$. 32
Table S23 ^{1}H NMR (400 MHz) and ^{13}C NMR (100 MHz) assignments for **10c** in $CDCl_3$. 33
Table S24 ^{1}H NMR (400 MHz) and ^{13}C NMR (100 MHz) assignments for **10d** in $CDCl_3$. 34
Table S25 ^{1}H NMR (400 MHz) and ^{13}C NMR (100 MHz) assignments for **11a** in methanol-d_4. 35
Table S26 ^{1}H NMR (400 MHz) and ^{13}C NMR (100 MHz) assignments for **11b** in methanol-d_4. 36
Table S27 ^{1}H NMR (400 MHz) and ^{13}C NMR (100 MHz) assignments for **11c** in $CDCl_3$. 37
Table S28 ^{1}H NMR (400 MHz) and ^{13}C NMR (100 MHz) assignments for **12b** in $CDCl_3$. 38
Table S29 ^{1}H NMR (400 MHz) and ^{13}C NMR (100 MHz) assignments for **12c** in acetone-d_6. 39
III. Physicochemical data of compounds. 40
IV. Supplementary Figures: 42
Figure S1 42
Figure S2 42
Figure S3 43
Figure S4 43
Figure S5 43
Figure S6-S7 NMR spectra of **1** in $CDCl_3$ (400 MHz). 44
Figure S8 EI mass spectrum of **1**. 45
Figure S9-S15 NMR spectra of **2** in $CDCl_3$ (400 MHz). 45
Figure S16 EI mass spectrum of **2**. 49
Figure S17-S18 NMR spectra of **3** in $CDCl_3$ (400 MHz). 49
Figure S19 EI mass spectrum of **3**. 50
Figure S20-S21 NMR spectra of **4** in $CDCl_3$ (400 MHz). 51
Figure S22 EI mass spectrum of **4**. 52
Figure S23-S24 NMR spectra of **5** in $CDCl_3$ (400 MHz). 52
Figure S25 EI mass spectrum of **5**. 53
Figure S26-S32 NMR spectra of **5′** in $CDCl_3$ (400 MHz). 54
Figure S33 EI mass spectrum of **5′**. 57
Figure S34-S40 NMR spectra of 6 in C_6D_6 (400 MHz). 58
Figure S41 EI mass spectrum of **6**. 61
Figure S42-S48 NMR spectra of **6′** in $CDCl_3$ (400 MHz). 62

Figure S49 EI mass spectrum of **6′**. 65

Figure S50-S51 NMR spectra of **7** in $CDCl_3$ (400 MHz). 66

Figure S52 EI mass spectrum of **7**. 67

Figure S53-S59 NMR spectra of **8** in $CDCl_3$ (400 MHz). 67

Figure S60 EI mass spectrum of **8**. 71

Figure S61-S62 NMR spectra of **9** in $CDCl_3$ (400 MHz). 71

Figure S63 EI mass spectrum of **9**. 72

Figure S64-S65 NMR spectra of **10** in $CDCl_3$ (400 MHz). 73

Figure S66 EI mass spectrum of **10**. 74

Figure S67-S68 NMR spectra of **11** in $CDCl_3$ (400 MHz). 74

Figure S69 EI mass spectrum of **11**. 75

Figure S70-S76 NMR spectra of **2a** in $CDCl_3$ (400 MHz). 76

Figure S77 EI mass spectrum of **2a**. 79

Figure S78-S84 NMR spectra of **3a** in methanol-d_4 (400 MHz). 80

Figure S85 EI mass spectrum of **3a**. 83

Figure S86-S92 NMR spectra of **3b** in methanol-d_4 (400 MHz). 84

Figure S93 EI mass spectrum of **3b**. 87

Figure S94-S100 NMR spectra of **3c** in methanol-d_4 (400 MHz). 88

Figure S101 EI mass spectrum of **3c**. 91

Figure S102-S108 NMR spectra of **3e** in methanol-d_4 (400 MHz). 92

Figure S109 EI mass spectrum of **3e**. 95

Figure S110-S116 NMR spectra of **4a** in $CDCl_3$ (400 MHz). 96

Figure S117 EI mass spectrum of **4a**. 99

Figure S118-S124 NMR spectra of **5a** in $CDCl_3$ (400 MHz). 100

Figure S125 EI mass spectrum of **5a**. 103

Figure S126-S132 NMR spectra of **6a** in $CDCl_3$ (400 MHz). 104

Figure S133 EI mass spectrum of **6a**. 107

Figure S134-S140 NMR spectra of **6b** in $CDCl_3$ (400 MHz). 108

Figure S141 EI mass spectrum of **6b**. 111

Figure S142-S148 NMR spectra of **9a** in $CDCl_3$ (400 MHz). 112

Figure S149 EI mass spectrum of **9a**. 115
Figure S150-S156 NMR spectra of **10a** in $CDCl_3$ (400 MHz). 116
Figure S157 EI mass spectrum of **10a**. 119
Figure S158-S164 NMR spectra of **10b** in $CDCl_3$ (400 MHz). 120
Figure S165 EI mass spectrum of **10b**. 123
Figure S166-S172 NMR spectra of **10c** in $CDCl_3$ (400 MHz). 124
Figure S173 EI mass spectrum of **10c**. 127
Figure S174-S180 NMR spectra of **10d** in $CDCl_3$ (400 MHz). 128
Figure S181 EI mass spectrum of **10d**. 131
Figure S182-S188 NMR spectra of **11a** in methanol-d_4 (400 MHz). 132
Figure S189 EI mass spectrum of **11a**. 135
Figure S190-S196 NMR spectra of **11b** in methanol-d_4 (400 MHz). 136
Figure S197 ^{13}C NMR spectrum of **11b** in $CDCl_3$ (100 MHz). 139
Figure S198 EI mass spectrum of **11b**. 140
Figure S199-S205 NMR spectra of **11c** in $CDCl_3$ (400 MHz). 140
Figure S206 EI mass spectrum of **11c**. 144
Figure S207-S2008 NMR spectra of **12a** in $CDCl_3$ (400 MHz). 144
Figure S209 EI mass spectrum of **12a**. 145
Figure S210-S216 NMR spectra of **12b** in $CDCl_3$ (400 MHz). 146
Figure S217 EI mass spectrum of **12b**. 149
Figure S218-S224 NMR spectra of **12c** in acetone-d_6 (400 MHz). 150
Figure S225 EI mass spectrum of **12c**. 153
References 154

I. Experimental Procedures:

DNA manipulation and plasmid construction.

The genomic DNA of actinobacteria was prepared according to the protocol described previously[1]. All plasmids were isolated from a 5 mL liquid culture of the corresponding *Escherichia coli* DH5α strains using the SanPrep Column Plasmid Mini-Preps Kit (Sangon Biotech (Shanghai) Co., Ltd., China). PCR amplifications were conducted on a Bio-Rad S1000™ Thermal Cycler using Phanta Max Super-Fidelity DNA Polymerase (Nanjing Vazyme Biotech Co., Ltd., China). ClonExpress MultiS One Step Cloning Kit (Nanjing Vazyme Biotech Co., Ltd., China) was used to clone DNA into plasmid. DNA concentrations were measured using the NanoDrop 2000c Microvolume UV–vis spectrophotometer (Thermo Fisher Scientific). Sequence identity was confirmed by sequencing (Tsingke Biotechnology Co., Ltd., China). The plasmids used in this study were shown in Table S3. Primers and oligonucleotides were also synthesized by Sangon Biotech (Shanghai, China) and were shown in Table S4.

A paired oligonucleotides SF14p-F, SF14p-R were degenerated, and ligated with linearized vector pSOK804[2] at *Hind*III and *EcoR*I endonucleases cleavage sites to generate plasmid pSOK-SF14p. The *crt* cluster containing *crtE, crtI* and *crtB* genes of *Streptomyces avermitilis* was amplified with primers crtEIB-F and crtEIB-R and cloned into *Bcu*I and *EcoR*I site of pSOK-SF14p to generate plasmid pSOK-SF14p-crt. The promoter *kasO*p* was amplified from plasmid pSET152-kasOp* with paired primers kasOp*-F/kasOp*-R, and clone into pSOK-SF14p-crt between *Hind*III and *Bcu*I site to generate plasmid pSOK-kasOp*-crt. The promoter ermEp was amplified from house-keeping plasmid pUW201 with paired primers ermEp-F/ermEp-R, and cloned into pSOK-SF14p-crt between *Hind*III and *Bcu*I site to generate plasmid pSOK-ermEp-crt. A SF14p-crtE gene fragment was amplified from pSOK-SF14p-crt using paired primers SF14p-F2/crtE-R, and a *ptlB* gene fragment was amplified from *S. avermitilis* genomic DNA using paired primers ptlB-F and ptlB-R, then both of the fragments were cloned into plasmid pSET152 at *Xba*I and *Xho*I site with hygromycin resistance gene to yield plasmid pSET-SF14p-EB. The identified terpene biosynthetic genes were amplified from genomic DNA and subsequently cloned into plasmid pSOK-SF14p between *Bcu*I and *EcoR*I sites. These plasmids were transferred into the *E. coli* ET12567 (pUZ8002) strain individually, and further introduced into *Streptomyces* by conjugation[3].

Culture conditions of recombinant strains.

ISP4 solid medium was used to display the color of strains, strains were cultured in plates for 3 days at 30 ℃. Recombinant strains containing lycopene BGC were cultured in four mediums: A, F, ISP2, TSB. Recombinant strains containing others terpenoids BGCs were just cultured in F medium. All of them were cultured for 6 days at 180 rpm shaker in 5 liters' scale using 1-liter shake flask containing 200 mL liquid medium. After fermentation, the culture was extracted with ethyl acetate directly, and ethyl acetate was removed under reduced pressure to obtain crude extract which can be used for terpenoids isolation.

ISP4 medium (1 liter): soluble starch 10 g, K_2HPO_4 1 g, $MgSO_4•7H_2O$ 1 g, NaCl 1 g, $(NH_4)_2SO_4$ 2 g, $CaCO_3$ 2 g, $FeSO_4•7H_2O$ 1 mg, $MnCl_2•4H_2O$ 1 mg, $ZnSO_4•7H_2O$ 1 mg, agar 20 g.

A medium (1 liter): soluble starch 10 g, yeast extract 4 g, peptone 2 g, $CaCO_3$ 1 g, $Fe_2(SO_4)_3•4H_2O$ 40 mg, KBr 100 mg.

F medium (1 liter): sucrose 20 g, glucose 10 g, casamino acids 0.1 g, yeast extract 5 g, MOPS 5 g, K_2SO_4 0.25 g, $MgCl_2•6H_2O$ 1 g, trace elements solution 1 mL. Trace elements solution: $ZnCl_2$ 40 mg/L, $FeCl_3•6H_2O$ 200 mg/L, $CuCl_2•2H_2O$ 10 mg/L, $MnCl_2•4H_2O$ 10 mg/L, $Na_2B_4O_7•10H_2O$ 10 mg/L, $(NH_4)_6Mo_7O_{24}•4H_2O$ 10 mg/L.

ISP2 medium (1 liter): glucose 4 g, yeast extract 4 g, malt extract 10 g.

TSB medium (1 liter): pancreatin digest of casein 17 g, papain digest of soybean meal 3 g, NaCl 5 g, K_2HPO_4 2.5 g, glucose 2.5 g.

Extraction and HPLC analysis of lycopene.

To extract lycopene from the *Streptomyces* strains, 2 mL aliquots of the cell cultures were centrifuged at 13,000 ×*g* for 10 min and washed twice with deionized water. The mycelia were lyophilized directly in dark, and grinded in liquid nitrogen using a grander Tissuelyser-GXF (Jingxin Industrial Development Co., Ltd., China), then the pigments were extracted with 1 mL organic reagents (acetone : ethyl acetate = 1 : 1), when necessary, several extraction cycles were performed to remove all visible colors from the cell pellets. The lycopene content in the extracts was quantified through absorbance at 470 nm by high performance liquid chromatography (HPLC) analyses on an Agilent 1200 series HPLC system, with a diode array detector (DAD) for UV/visible (Vis) spectrum. A Poroshell 120 EC-C18 column (4.6 × 50 mm, 2.7 μm, Agilent) was used and isocratic elution with 80% acetonitrile/methanol for 20 min during the analysis. The injection volume was 5 μL and the flow rate was kept constantly at 0.5 mL/min. And the concentrations of lycopene were calculated using a standard curve (Figure S3B) and appropriate dilutions. The lycopene (B20378, HPLC > 90 %) was purchased from Shanghai yuanye Bio-Technology, the UV-Vis spectrum of standard and extract lycopene were showed in Figure S3C.

Sequence similarity network (SSN) of terpene synthases.

A proteome database was established based on sequenced actionbacteria in our laboratory collection including obtained from public collections and isolated from nature directly. HMM model search was achieved by command hmmsearch of HMMER 3.1b2 with default parameters. The e-values of terpene synthases to each other were calculated using the ncbi-blast tool, and the SSN was visualized by CytoScape 3.5.1 software[4].

Isolation and analysis of terpenoids.

Terpene skeletons were purified from crude extracts by column chromatography on silica gel using petroleum ether as eluent. Low polarity terpenoids containing modification were also purified by silica-gel column, and eluted with the mixture of petroleum ether and ethyl acetate. Crude extracts of other terpenoids were fractionated by Medium Pressure Liquid Chromatography (MPLC) over ODS column eluted with a linear gradient MeOH-H_2O system from 10% MeOH to 100% MeOH. The target fractions were further purified by semi-preparative HPLC. LC-MS analysis was performed on an Agilent 6530 TOF LC/MS mass spectrometer with a Poroshell 120 EC-C18 column (4.6×50 mm, 2.7 μm, Agilent Techonologies). GC-MS analysis was performed on an Agilent (Santa Clara, CA, USA) 7890B GC fitted with a HP-5ms fused silica capillary column (30 m, 0.25 mm i. d., 0.25 μm film), which was connected to a 5977A mass detector was used to record GC/MS data. GC parameters were 1) inlet pressure: 77.1 kPa, He at 20 mL min^{-1}, 2) injection volume: 1 μL, 3) temperature program: 5 min at 80 ℃ increasing at 10 ℃ min^{-1} to 240 ℃ and then 5 ℃ min^{-1} to 280 ℃, 4) 60 s valve time, 5) carrier gas: He at 1.0 mL min^{-1}. MS parameters were 1) source: 230 ℃, 2) transfer line: 250 ℃, 3) quadrupole: 150 ℃ and 4) electron energy: 70 eV. The NMR spectra were collected on Bruker Avance 400 with 400 MHz for ^1H and 100 MHz for ^{13}C nuclei. Optical rotations were recorded on a S3 Rudolph Research Analytical Autopol VI automatic polarimeter using a 50 mm pathlength cell with $[\alpha]_D^{25}$ values reported in degrees; concentration (*c*) is in g/100mL.

II. Supplementary Tables:

Table S1 The titer (mg / L) of lycopene produced by recombinant strains in different conditions.

Medium/promoter	J1074-crt	1018-crt	M1154-crt	SBT18-crt
A/ SF14p	22.19 ±1.2	10.05 ±0.64	10.49 ±1.84	7.8 ±0.83
F/ SF14p	32.83 ±2.43	8.72 ±0.22	8.37 ±1.77	3.25 ±0.27
F/ kasOp*	8.55 ±0.99			
F/ ermEp	5.21 ±0.64			
ISP2/ SF14p	15.92 ±0.67	19.16 ±3.14	2.76 ±0.3	6.34 ±0.38
TSB/ SF14p	12.8 ±0.14	5.17 ±0.51	2.85 ±0.08	4.41 ±0.46

Table S2 Characterized bacterial terpene synthases and the e-value of HMM model search.

Entry	PF03936	PF19086	Product / annotation	Cn	Ref.
A0A0H5BN57	2.7E-05	1E-32	(12E)-Labda-8(17),12,14-triene	20	(5,6)
A0A0H5BN61	4.1E-05	8E-36	(12E)-Labda-8(17),12,14-triene	20	(5,6)
A0A291SJC7	0.00051	3E-36	(+)-Isoafricanol	15	(7)
A3KI17	3.8E-05	1E-29	2-Methylisoborneol	11	(8)
A4FG19		4E-31	2-Methylisoborneol	11	(8)
A7NH01	0.00024	2E-49	(+)-T-Muurolol	15	(9)
A9FZ87	2E-06	3E-44	(+)-Eremophilene	15	(9)
A9GK58	6.7E-05	9E-43	10-*epi*-Cubebol	15	(9)
ADU79148		2E-26	2-Methylisoborneol	11	(9)
ADU79149		9E-26	2-Methylisoborneol	11	(10)
AEK21533		6E-25	2-Methylisoborneol	11	(10)
AGO55049	8.5E-06	2E-24	Sodorifen	15	(11)
ALO06273	7.2E-06	6E-32	Venezuelaenes, VenA	20	(12)
B1W019	5.6E-06	1E-39	(+)-Caryolan-1-ol	15	(9)
B2J4A4	1.9E-10	1E-49	5-*epi*-α-Selinene	15	(9)
B5GMG2	7.2E-07	4E-46	1,8-Cineole	10	(9)
B5GRC8		2E-21	Labda-7,13(16),14-triene	20	(6)
B5GS26		4E-36	(-)-δ-Cadinene	15	(9)
B5GW45		4E-31	(+)-T-Muurolol	15	(9)
B5H7H3	1.7E-08	3E-47	Pristinol	15	(13)
B5HDJ6		8E-32	Selina-4(15),7(11)-diene	15	(9)

BAL14866	6E-06	4E-41	(-)-Germacradien-4-ol	15	(9)
BAL14867		8E-31	(−)-*epi*-*α*-Bisabolol	15	(9)
BAP82203	8.6E-07	4E-32	Cyclooctat-7(8),10(14)-diene	20	(9)
BAP82213	0.00037	3E-39	(−)-Germacradien-4-ol	15	(9)
BAP82216		4E-34	(−)-Isohirsut-4-ene	15	(9)
BAP82223	0.00021	9E-35	Selina-3,7(11)-diene	15	(9)
BAP82229	4.6E-07	1E-28	Odyverdiene-A	20	(9)
C7PLV2	2.1E-06	9E-49	γ-Cadinene	15	(9)
C9K1X5		2E-08	Cyclooctat-9-en-7-ol, CotB2	20	(9)
CCA53839	4.2E-07	1E-33	(+)-Dauca-8,11-diene	15	(9)
D2B747	6.4E-08	2E-43	4-epi-Cubebol	15	(9)
D3KYU2	3.2E-05	2E-29	2-Methylisoborneol	11	(8)
D5SL78		2E-37	(3R)-Linalool	10	(9)
D9XD61	1.5E-05	3E-42	7-*epi*-α-Eudesmol	15	(9)
D9XDR8		2E-23	α-Amorphene	15	(9)
E3VWJ0	1.3E-09	3E-44	Pentalenene	15	(14)
E4MYY0	3.1E-05	1E-37	(2Z,6E)-Hedycaryol	15	(9)
E4N7E5		3E-30	Corvol-ether A and B	15	(9)
E8W6C7	3.6E-09	6E-38	(+)-(1(10)E,4E,6S,7R)-Germacradien-6-ol	15	(9)
EFG04655		2E-28	Clavulatriene A	20	(9)
EFG04889		1E-29	(-)-Isohirsut-1-ene	15	(9)
G2P5T1	8.8E-05	6E-39	Isoafricanol	15	(9)
I2N045		2E-26	Tsukubadiene, TdS	20	(9)
K0K750	1.2E-08	8E-41	(E)-β-Caryophyllene	15	(9)
M1V9Q0		4E-24	Cembrene C, DtcycA	20	(15)
M1VDX3		9E-18	Nephthenol, DtcycB	20	(15)
P0DPK6	9E-06	3E-36	Spiroviolene_SvS	20	(16)
Q45222	8.8E-08	3E-12	*ent*-kaurene, blr2150	20	(17)
Q55012	6.5E-08	4E-44	Pentalenene	15	(9)
Q5KSN4	0.0012	1E-05	*ent*-Pimara-9(11)-15-diene	20	(18)
Q82IY4	4E-09	5E-43	Pentalenene	15	(19)
Q82RR7		7E-41	Avermitilol	15	(9)
Q9AJE3	0.00093	1E-23	Terpentetriene, CYC2	20	(20)
Q9F1V8	3.3E-06	6E-31	2-Methylisoborneol	11	(10)

Q9F1Y6	0.00035	1E-26	2-Methylisoborneol	11	(9)
Q9K499	5.8E-05	2E-38	*epi*-Isozizaene	15	(9)
Q9X839	1.4E-07	2E-88	Germacradienol and Geosmin	15	(21)
TWH66842	0.0011	5E-25	2-Methylisoborneol	11	(10)
WP_003955204	3.2E-05	6E-34	Intermedeol	15	(9)
WP_003963279		2E-27	Hydropyrene	20	(9)
WP_003963391	5.1E-06	4E-34	African-2-ene	15	(9)
WP_004941320		6E-19	Sestermobaraene	25	(22)
WP_006348376	0.00058	3E-33	Selina-3,7(11)-diene	15	(9)
WP_010314578	4.7E-06	8E-35	Spinodiene, SoS	20	(9)
WP_010998816	7.1E-07	2E-41	Germacrene A	15	(9)
WP_011318775	2.5E-08	7E-42	Germacrene A	15	(9)
WP_011333305	0.0002	8E-31	2-Methylenebornane	11	(9)
WP_011958209	2.7E-05	3E-49	(+)-T-Muurolol	15	(9)
WP_012190524	2.3E-07	6E-41	Obscuronatin	20	(9)
WP_012190525	8.4E-06	4E-36	α-Selinene	15	(9)
WP_012381690	2.8E-08	6E-47	(+)-*epi*-Cubenol	15	(9)
WP_012394883		1E-36	(+)-Allohedycaryol	15	(9)
WP_012789469	1.5E-09	3E-40	18-Hydroxydolabella-3,7-diene	20	(9)
WP_013004899	6.3E-05	3E-31	Neomeranol B	15	(23)
WP_014150548		7E-32	Cattleyene, CyS	20	(24)
WP_028183010	2.9E-11	8E-18	Isopimara-8,15-diene, SaDTS	20	(25)
WP_030426588		2E-35	Spiroalbatene, SaS	20	(26)
WP_030430753		1E-35	Cembrene A, CAS	20	(26)
WP_030431358		9E-21	Allokutznerene and Phomopsene, PmS	20	(27)
WP_030432512		8E-30	Bonnadiene, BdS	20	(27)
WP_035852539		6E-29	β-Himachalene	15	(28)
WP_035857999		1E-32	γ-Bisabolene	15	(29)
WP_039829446		5E-26	Phomopsene	20	(26)
WP_041328593	9.9E-06	2E-44	Cembrene C	20	(26)
WP_051714436		9E-24	Phomopsene	20	(25)
WP_052407688		9E-21	Phomopsene	20	(25)
WP_054468580		2E-25	2-Methylisoborneol	11	(10)
WP_073290622		8E-31	Chryseodiene, CpCS	20	(30)

WP_089795910	5.2E-08	5E-35	Wanjudiene, CwWS	20	(30)
WP_091046421	0.00055	1E-38	Micromonocyclol	20	(31)
WP_095757924	1.5E-05	2E-45	Spata-13,17-diene, SpS	20	(32)
WP_100105659	0.00015	1E-86	Germacradienol and Geosmin	15	(21)
WP_190371453		7E-90	Germacradienol and Geosmin	15	(21)
WP_239771469	0.00029	2E-35	Benditerpe-2,6,15-triene, Bnd4	20	(33)
YP_001866236	2.2E-06	1E-88	Geosmin	15	(9)

Table S3 Genbank number of terpenoids biosynthetic genes were mentioned in this study.

Symbol of gene	Genbank No.	Symbol of gene	Genbank No.
crtE	BAB69143	*euoG*	PNE32286
crtI	BAB69144	*euoT*	PNE32120
crtB	BAB69145	*euoD*	PNE32119
ptlB	BAC70708	*sspM*	OLZ59235
argF	UYP65652	*sspG*	OLZ59235
argT	UYP65651	*sspT*	OLZ59233
eurT	PNE30129	*aliG*	PAU49966
eurP	PNE30127	*aliT*	PAU49965
netO	GGR09481	*aliT'*	PAU49964
netT	GGR09488	*aliP*	PAU49973
netA	GGR09494	*xylT*	SDY90229
netP	GGR09501	*xylP*	SDY90207
forF	ATL29994	*natG*	KIZ16993
forD1	ATL29995	*natT1*	KIZ16994
forD2	ATL29996	*natT2*	KIZ16995
forT	ATL29997	*spgP*	UYP65655
morG	GHF23893	*spgT1*	UYP65653
morT	GHF23885	*spgT2*	UYP65654
morP	GHF23877	*vspP*	QKW17372
ariP1	SFP28037	*vspT1*	QKW17371
ariT	SFP28068	*vspT2*	QKW17370
ariP2	SFP28100	*aspP1*	QKW39411
ariP3	SFP28132	*aspP2*	QKW39412
albG	PAU47878	*aspT1*	QKW39413
albT	PAU47876	*aspT2*	QKW40963
albU	PAU47875	*fraT1*	SDG51259
albP1	PAU47874	*fraT2*	SDG51286
albP2	PAU47873	*fraG*	SDG51315
		fraP	SDG51324

Table S4 Plasmids were achieved in this study.

Plasmid	Genes were cloned	Product	Discription
pSOK-SF14p	-	-	a SF14p promoter was inserted into pSOK804 between *Hind*III and *EcoR*I site.
pSOK-SF14p-crt	*crtE*, *crtB*,*crtI*	lycopene	a crt cluster containing crtE, crtB, crtI was inserted into pSOK-SF14p between *Bcu*I and *EcoR*I site.
pSOK-kasOp*-crt	*crtE*, *crtB*,*crtI*	lycopene	promoter SF14p was deleted and promoter kasOp* was inserted into pSOK-SF14p-crt between *Hind*III and *Bcu*I site.
pSOK-ermEp-crt	*crtE*, *crtB*,*crtI*	lycopene	promoter SF14p was deleted and promoter ermEp was inserted into pSOK-SF14p-crt between *Hind*III and *Bcu*I site.
pSET-SF14p-EB	*crtE*, *crtB*,*crtI*	lycopene	crtE and ptlB under control of SF14p promoter inserted into pSET152 between *Xba*I and *Xho*I.
pSOK-argT	*argT*	**1**	gene argT was amplified by paired primers argT-F / argT-R and cloned into pSOK-SF14p between *Bcu*I and *EcoR*I site.
pSOK-eurT	*eurT*	**2**	gene eurT was amplified by paired primers eurT-F / eurT-R and cloned into pSOK-SF14p between *Bcu*I and *EcoR*I site.
pSOK-netT	*netT*	**3**	gene netT was amplified by paired primers netT-F / netT-R and cloned into pSOK-SF14p between *Bcu*I and *EcoR*I site.
pSOK-forT	*forT*	-	gene forT was amplified by paired primers forT-F / forT-R and cloned into pSOK-SF14p between *Bcu*I and *EcoR*I site.
pSOK-morT	*morG, morT*	**4**	fragment morG-morT was amplified by paired primers morG-F / morT-R and cloned into pSOK-SF14p between *Bcu*I and *EcoR*I site.
pSOK-ariT	*ariT*	**5**, **5'**	gene ariT was amplified by paired primers ariT-F / ariT-R and cloned into pSOK-SF14p between *Bcu*I and *EcoR*I site.
pSOK-albGT	*albG, albT*	**6**, **6'**	fragment albG-albT was amplified by paired primers albG-F / albT-R and cloned into pSOK-SF14p between *Bcu*I and *EcoR*I site.
pSOK-euoGT	*euoG, euoT*	**7**	fragment euoG-euoT was amplified by paired primers euoG-F / euoT-R and cloned into pSOK-SF14p between *Bcu*I and *EcoR*I site.
pSOK-sspT	*sspT*	**7**	gene sspT was amplified by paired primers sspT-F / sspT-R and cloned into pSOK-SF14p between *Bcu*I and *EcoR*I site.
pSOK-aliGTT'	*aliG, aliT, aliT'*	-	fragment aliG-aliT-aliT' was amplified by paired primers aliG-F / aliT'-R and cloned into pSOK-SF14p between *Bcu*I and *EcoR*I site.
pSOK-xylT	*xylT*	-	gene xylT was amplified by paired primers xylT-F / xylT-R and cloned into pSOK-SF14p between *Bcu*I and *EcoR*I site.
pSOK-natGT1T2	*natG, natT1, natT2*	**8**	fragment natG-natT1-natT2 was amplified by paired primers natG-F / natT2-R and cloned into pSOK-SF14p between *Bcu*I and *EcoR*I site.
pSOK-spgT1T2	*spgT1, spgT2*	**9**	fragment spgT1-spgT2 was amplified by paired primers spgT1-F / spgT2-R and cloned into pSOK-SF14p between *Bcu*I and *EcoR*I site.
pSOK-vspT1T2	*vspT1, vspT2*	**10**	fragment vspT1-vspT2 was amplified by paired primers vspT1-F / vspT2-R and cloned into pSOK-SF14p between *Bcu*I and *EcoR*I site.
pSOK-aspT1T2	*aspT1, aspT2*	**11**	fragment aspT1-aspT2 was amplified by paired primers aspT1-F / aspT2-R and cloned into pSOK-SF14p between *Bcu*I and *EcoR*I site.
pSOK-fraT1T2G	*fraT1, fraT2, fraG*	**10**	fragment fraT1-fraT2-fraG was amplified by paired primers fraT1-F / fraG-R and cloned into pSOK-SF14p between *Bcu*I and *EcoR*I site.
pSOK-eurTP	*eurT, eurP*	**2a**	fragment eurT-eurP was amplified by paired primers eurT-F / eurP-R and cloned into pSOK-SF14p between *Bcu*I and *EcoR*I site.
pSOK-netOTAP	*netO, netT, netA, netP*	**3a**, **3b**	fragment netOTAP was amplified by paired primers netO-F / netP-R and cloned into pSOK-SF14p between *Bcu*I and *EcoR*I site.
pSOK-netOT	*netO, netT*	**3c, 3d**	fragment netOT was amplified by paired primers netO-F / netT-R and cloned into pSOK-SF14p between *Bcu*I and *EcoR*I site.

pSOK-netTP	*netT, netP*	**3e**	fragment netT was amplified by paired primers netT-F / netT-R2, fragment netP was amplified by paired primers netP-F / netP-R, both of them were cloned into pSOK-SF14p between *Bcu*I and *EcoR*I site.
pSOK-netTA	*netT, netA*	-	fragment netTA was amplified by paired primers netT-F / netA-R and cloned into pSOK-SF14p between *Bcu*I and *EcoR*I site.
pSOK-morGTP	*morG, morT, morP*	**4a**	fragment morG-morT-morP was amplified by paired primers morG-F / morP-R and cloned into pSOK-SF14p between *Bcu*I and *EcoR*I site.
pSOK-ariP1TP2P3	*ariP1, ariT, ariP2, ariP3*	**5a**	fragment ariP1TP2P3 was amplified by paired primers ariP1-F / ariP3-R and cloned into pSOK-SF14p between *Bcu*I and *EcoR*I site.
pSOK-albGTUP1P2	*albG, albT, albU, albP1, albP2*	**6a, 6b**	fragment albGTUP1P2 was amplified by paired primers albG-F / ariP2-R and cloned into pSOK-SF14p between *Bcu*I and *EcoR*I site.
pSOK-albGTUP1	*albG, albT, albU, albP1*	**6a, 6b**	fragment albGTUP1 was amplified by paired primers albG-F / albP1-R and cloned into pSOK-SF14p between *Bcu*I and *EcoR*I site.
pSOK-albGTUP2	*albG, albT, albU, albP2*	-	fragment albGTU was amplified by paired primers albG-F / albU-R, fragment albP was amplified by paired primers albP2-F / albP2-R, both of them were cloned into pSOK-SF14p between *Bcu*I and *EcoR*I site.
pSOK-euoGTD	*euoG, euoT, euoD*	**7**	fragment euoG-euoT-euoD was amplified by paired primers euoG-F / euoD-R and cloned into pSOK-SF14p between *Bcu*I and *EcoR*I site.
pSOK-sspGTM	*sspG, sspT, sspM*	**7**	fragment sspG-sspT-sspM was amplified by paired primers sspG-F / sspM-R and cloned into pSOK-SF14p between *Bcu*I and *EcoR*I site.
pSOK-spgPT1T2	*spgP, spgT1, spgT2*	**9a**	fragment spgP-spgT1-spgT2 was amplified by paired primers spgP-F / spgT2-R and cloned into pSOK-SF14p between *Bcu*I and *EcoR*I site.
pSOK-vspPT1T2	*vspP, vspT1, vspT2*	**10a**, **10b**, **10c**, **10d**	fragment vspP-vspT1-vspT2 was amplified by paired primers vspP-F / vspT2-R and cloned into pSOK-SF14p between *Bcu*I and *EcoR*I site.
pSOK-aspP1P2T1T2	*aspP1, aspP2, aspT1, aspT2*	**11a**, **11b**	fragment aspP1P2T1T2 was amplified by paired primers aspP1-F / aspT2-R and cloned into pSOK-SF14p between *Bcu*I and *EcoR*I site.
pSOK-aspP2T1T2	*aspP2, aspT1, aspT2*	**11c**	fragment aspP2T1T2 was amplified by paired primers aspP2-F / aspT2-R and cloned into pSOK-SF14p between *Bcu*I and *EcoR*I site.
pSOK-aspP1T1T2	*aspP1, aspT1, aspT2*	-	fragment aspP1 was amplified by paired primers aspP1-F / aspP1-R, fragment aspT1T2 was amplified by paired primers aspT1-F2 / aspP2-R, both of them were cloned into pSOK-SF14p between *Bcu*I and *EcoR*I site.
pSOK-fraT1T2GP	*fraT1, fraT2, fraG, fraP*	**12a**, **12b**, **12c**	fragment fraT1T2GP was amplified by paired primers fraT1-F / fraP-R and cloned into pSOK-SF14p between *Bcu*I and *EcoR*I site.

Table S5 Primers and oligonucleotides were used in this study.

Primer name	Sequence (5' to 3')
SF14p-F	AGCTTGGGCTGCAGGTCGACTCTAGAGCCTTGACCTTGATGAGGCGGCGTGAGCTACAATCAATACT CGATTAGGGATCCACGTCATATGACGTA
SF14p-R	CTAGTACGTCATATGACGTGGATCCCTAATCGAGTATTGATTGTAGCTCACGCCGCCTCATCAAGGTC AAGGCTCTAGAGTCGACCTGCAGCCCA
crtEIB-F	GATCCACGTCATATGACGTACTAGTACGACGAGAGGAACCGGGAT
crtEIB-R	CAGCTATGACATGATTACGAATTCGGGTCATGCGACCTCCTCATGTG
kasOp*-F	ACGCCTCATGGAGGGCGCGAAGCTTGGGCTGCAGGTCGACTCTAGA
kasOp*-R	ATCCCGGTTCCTCTCGTCGTACTAGTACGTATGCATGCAGCATCG
ermEp-F	TACGCCTCATGGAGGGCGCGAAGCTTGAAGCAGCTCCAGCCTACA
ermEp-R	TCCCGGTTCCTCTCGTCGTACTAGTGGATCGATCCTACCAACCGG
SF14p-F2	AGCTTGGGCTGCAGGTCGACTCTAGA
crtE-R	GCGTATCCCCTTTCAGATACTCATCGGGAGGCCCCCTC

ptlB-F	GTATCTGAAAGGGGATACGCAATGACCGTGACCCCGGAGTC
ptlB-R	CAGCTATGACATGATTACCTCGAGTCAGACCTCCCGGTCCACGACGA
argT-F	GATCCACGTCATATGACGTACTAGTCTCGGAGGAGTTCTTTCGGC
argT-R	CAGCTATGACATGATTACGAATTCCACTCAGATGAAGTCCCACCACC
eurT-F	GGATCCACGTCATATGACGTACTAGTTACCCCTGTTTCTGCGCATC
eurT-R	CAGCTATGACATGATTACGAATTCATTTCTTCACTGAGGCCCCC
eurP-R	CAGCTATGACATGATTACGAATTCATCAACCGTGCGCCGAAGCT
netT-F	CGATGCTGCATGCATACGTACTAGTACTTCGCTGAGGACGTGCGT
netT-R	CAGCTATGACATGATTACGAATTCCGGGCATGACGTGTTCCCTT
netO-F	CGATGCTGCATGCATACGTACTAGTGTGTTTCACCGCATACGCCG
netT-R2	TGAGTGGTGCCTCGCAGTACCGGGCATGACGTGTTCCCTT
netA-R	CAGCTATGACATGATTACGAATTCGAGGTGATGAAGGGCAGTGG
netP-F	AAGGGAACACGTCATGCCCGGTACTGCGAGGCACCACTCA
netP-R	CAGCTATGACATGATTACGAATTCACATCGCGAACAGCCAGGCA
forT-F	GATCCACGTCATATGACGTACTAGTCATCCACTGTTCAGGAGGAC
forT-R	CAGCTATGACATGATTACGAATTCAAGTCCAACTCGCCTTCAGA
morG-F	GATCCACGTCATATGACGTACTAGTAACGATGCGAAGGGGATCGA
morT-R	CAGCTATGACATGATTACGAATTCTACGGATCGGTGGGTTCAGT
morP-R	CAGCTATGACATGATTACGAATTCTCTCGACAGGGGCCTGTCAT
ariT-F	GATCCACGTCATATGACGTACTAGTGAGGACCGTTGCCATGTCGA
ariT-R	CAGCTATGACATGATTACGAATTCGGTGAGGAACAGCGTGGACAT
ariP1-F	GATCCACGTCATATGACGTACTAGTAACGACGACCGCAAGGAGTC
ariP3-R	CAGCTATGACATGATTACGAATTCATGGGTTCCATCCCGGTGTC
albG-F	GGATCCACGTCATATGACGTACTAGTCCGCAGGCAGGTGACGTC
albT-R	CAGCTATGACATGATTACGAATTCGACCCATCGGAATCCCCTTC
albU-R	AGGGGTCAGTGGACCGTTTCTCAATTCCGTGCTGCCTGGC
albP1-R	CAGCTATGACATGATTACGAATTCTCAGTGGACCGTTTCGAGGG
albP2-F	GCCAGGCAGCACGGAATTGAGAAACGGTCCACTGACCCCT
albP2-R	CAGCTATGACATGATTACGAATTCTCACCAAAAGGTGGGGCGCC
euoG-F	GATCCACGTCATATGACGTACTAGTAAGGACCTCTTCCGCCGA
euoT-R	CAGCTATGACATGATTACGAATTCCTCATGTGCTTCCCGGTACG
euoD-R	CAGCTATGACATGATTACGAATTCCCCCAACGAGGGCTTAGCTT
sspM-F	GATCCACGTCATATGACGTACTAGTCCCACCCGCGTATCAAGGAG
sspT-F	GATCCACGTCATATGACGTACTAGTACTACCTGTTCCTCCTCGCC
sspT-R	CAGCTATGACATGATTACGAATTCCTAGTCGGCGGACTTCCAGT
aliG-F	GATCCACGTCATATGACGTACTAGTACAGACGCGAGGTGCTGCCA
aliT'-R	CAGCTATGACATGATTACGAATTCGGAGGGTATGTGGGTGCC
xylT-F	GATCCACGTCATATGACGTACTAGTACGTTGCGGATGGTGAGGGT
xylT-R	CAGCTATGACATGATTACGAATTCCTTGACGGTGTGGCCCACCA
natG-F	GATCCACGTCATATGACGTACTAGTTCCGCAGGCAGGTGACAACC
natT2-R	CAGCTATGACATGATTACGAATTCTCAGGCTCCTGGGTGTGGTT
spgT1-F	GATCCACGTCATATGACGTACTAGTAAGATCGAGCTGGAACTCCG

spgT2-R	CAGCTATGACATGATTACGAATTCCGGTGAAAATCCGGCGAATG
spgP-F	GATCCACGTCATATGACGTACTAGTTCACGTCGCGCAGATTCACG
vspT1-F	GATCCACGTCATATGACGTACTAGTGGTCGAGGTGGTCCTGCG
vspT2-R	CAGCTATGACATGATTACGAATTCGCACGCTAACAGCGTCACC
vspP-F	GATCCACGTCATATGACGTACTAGTCGAAGAGTAACGCCCCTCAC
aspT1-F	GATCCACGTCATATGACGTACTAGTGAGCACGAGCTGCGGAT
aspT2-R	CAGCTATGACATGATTACGAATTCGTTTGCCGCTTTACGTCCTT
aspP1-F	GATCCACGTCATATGACGTACTAGTCGCAAGCCCGGCAACGCGAT
aspP2-F	GATCCACGTCATATGACGTACTAGTATCGAGCGCAAGCCCGGCAA
aspT1-F2	GCTGAATGTCCGTCCTCACCATGCGCCTCGTCCCGATCGA
aspP1-R	GGATCCACGTCATATGACGTACTAGTAAGGGATCTCCGTCCGATGGCC
fraT1-F	GATCCACGTCATATGACGTACTAGTGAGCACACCGACTGGAGTTC
fraG-R	CAGCTATGACATGATTACGAATTCTCGTCCCACCTTCTCGTCGA
fraP-R	CAGCTATGACATGATTACGAATTCTTTCGTCCGGACGTCGGGTC

Table S6 ^{1}H NMR (400 MHz) and ^{13}C NMR (100 MHz) assignments for **2** in $CDCl_3$.

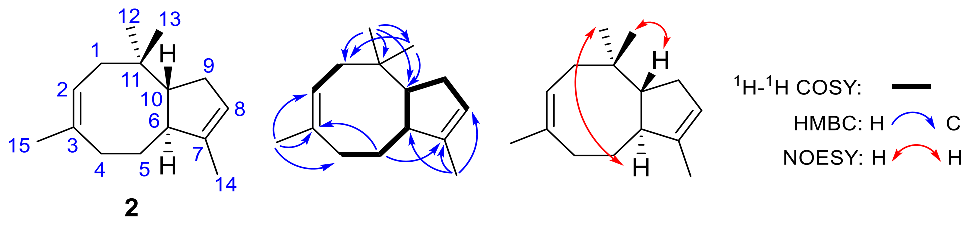

Position	$\delta_{C,}$ type	δ_H (mult, J in Hz)
1	37.2, CH_2	2.22 (1H, m)
		2.05 (1H, m)
2	122.3, CH	5.35 (1H, t, J = 5.4)
3	138.6, C	
4	32.4, CH_2	2.34 (1H, m)
		1.96 (1H, m)
5	31.7, CH_2	1.98 (1H, m)
		0.99 (1H, m)
6	52.4, CH	2.35 (1H, m)
7	142.6, C	
8	123.5, CH	5.18 (1H, m)
9	33.9, CH_2	2.21 (1H, m)
		2.10 (1H, m)
10	49.9, CH	2.13 (1H, m)
11	41.1, C	
12	23.6, CH_3	0.77 (3H, s)
13	19.9, CH_3	0.84 (3H, s)
14	15.2, CH_3	1.61 (3H, s)
15	25.0, CH_3	1.75 (3H, s)

Table S7 ^{1}H NMR (400 MHz) and ^{13}C NMR (100 MHz) assignments for **5′** in $CDCl_3$.

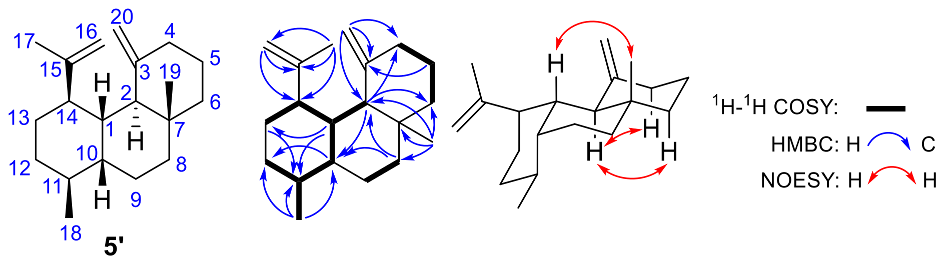

Position	δ_C, type	δ_H (mult, *J* in Hz)
1	34.1, CH	2.16 (1H, m)
2	49.1, CH	2.32 (1H, d, *J* = 12.1)
3	148.9, C	
4	39.4, CH_2	2.28 (1H, m)
		1.97 (1H, m)
5	25.2, CH_2	1.64 (2H, m)
6	42.9, CH_2	1.38 (2H, m)
7	37.9, C	
8	35.5, CH_2	1.43 (1H, m)
		1.15 (1H, m)
9	23.0, CH_2	1.54 (2H, m)
10	39.2, CH	1.55 (2H, m)
11	28.6, CH	1.82 (1H, m)
12	31.3, CH_2	1.41 (1H, m)
		1.10 (1H, m)
13	24.0, CH_2	1.67 (2H, m)
14	39.7, CH	2.68 (1H, m)
15	148.2, C	
16	110.3, CH_2	4.91 (1H, br)
		4.85 (1H, br)
17	23.4, CH_3	1.74 (3H, s)
18	20.5, CH_3	0.82 (3H, d, *J* = 6.3)
19	18.1, CH_3	0.82 (3H, s)
20	107.0, CH_2	4.87 (1H, br)
		4.32 (1H, br)

Table S8 ^{1}H NMR (400 MHz) and ^{13}C NMR (100 MHz) assignments for **6′** in $CDCl_3$.

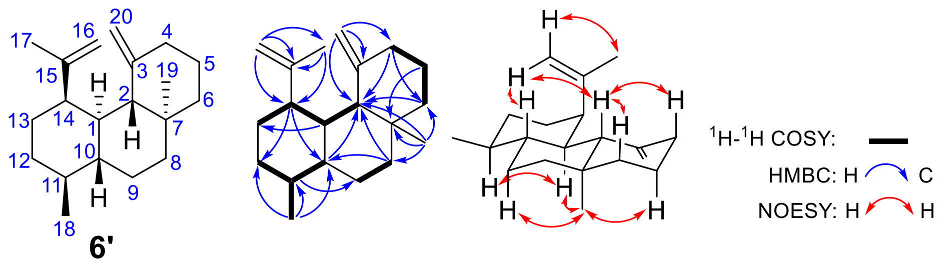

Position	δ_{C}, type	δ_{H} (mult, *J* in Hz)
1	40.9, CH	1.50 (1H, m)
2	52.3, CH	1.89 (1H, d, *J* = 11.1)
3	149.3, C	
4	39.2, CH_2	2.22 (1H, m)
		1.89 (1H, m)
5	25.3, CH_2	1.61 (2H, m)
6	42.7, C	1.34 (1H, m)
		1.27 (1H, m)
7	36.9, C	2.84 (1H, m)
8	41.4, CH_2	1.46 (1H, m)
		1.16 (1H, m)
9	25.6, CH_2	1.01 (2H, m)
10	43.0, CH	1.34 (1H, m)
11	38.4, CH	1.18 (1H, m)
12	31.6, CH_2	1.47 (1H, m)
		1.36 (1H, m)
13	30.7, CH_2	1.70 (1H, m)
		1.61 (1H, m)
14	39.6, CH	2.92 (1H, m)
15	148.5, C	
16	113.1, CH_2	4.83 (1H, brs)
		4.79 (1H, brs)
17	26.9, CH_3	1.73 (3H, s)
18	20.4, CH_3	0.91 (3H, d, *J* = 6.3)
19	17.9, CH_3	0.74 (3H, s)
20	106.7, CH_2	4.88 (1H, brs)
		4.40 (1H, brs)

Table S9 ^{1}H NMR (400 MHz) and ^{13}C NMR (100 MHz) assignments for **6** in C_6D_6.

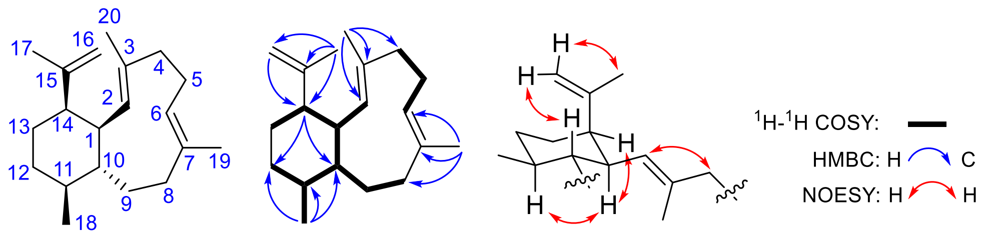

6

Position	δ_C, type	δ_H (mult, *J* in Hz)
1	46.6, CH	2.08 (1H, m)
2	135.8, CH	4.82 (1H, br)
3	132.4, C	
4	40.0, CH_2	2.07 (1H, m)
		1.94 (1H, m)
5	26.3, CH_2	2.20 (1H, m)
		2.02 (1H, m)
6	126.8, CH	4.80 (1H, m)
7	130.8, C	
8	41.7, CH_2	2.35 (1H, m)
		1.99 (1H, m)
9	35.0, CH_2	1.82 (2H, m)
10	49.8, CH	1.14 (1H, m)
11	36.9, CH	1.11 (1H, m)
12	30.2, CH_2	1.64 (1H, m)
		1.31 (1H, m)
13	28.0, CH_2	1.65 (1H, m)
		1.45 (1H, m)
14	45.9, CH	2.30 (1H, m)
15	147.2, C	
16	112.5, CH	4.99 (1H, br)
		4.87 (1H, br)
17	25.8, CH_3	1.68 (3H, s)
18	21.8, CH_3	0.98 (3H, d, *J* = 5.5)
19	16.5, CH_3	1.45 (3H, s)
20	30.5, CH_3	1.45 (3H, s)

Table S10 ^{1}H NMR (400 MHz) and ^{13}C NMR (100 MHz) assignments for **8** in $CDCl_3$.

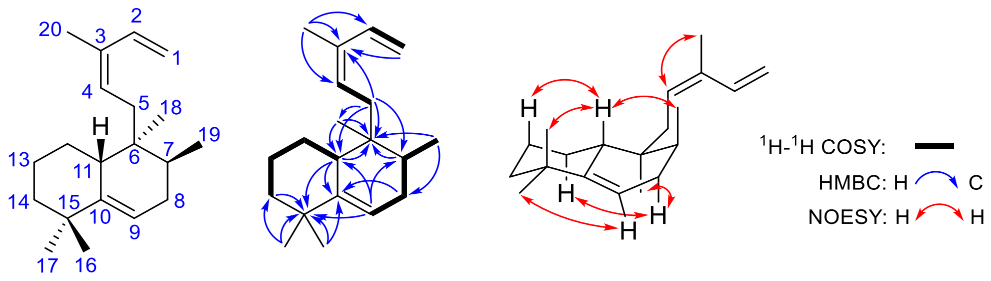

8

Position	$\delta_{C,}$ type	δ_H (mult, *J* in Hz)
1	113.0, CH_2	5.20 (1H, dd, *J* = 17.2, 1.0)
		5.07 (1H, dd, *J* = 10.8, 1.0)
2	134.4, CH	6.79 (1H, dd, *J* = 10.8, 17.2)
3	133.7, C	
4	128.4, CH	5.50 (1H, t, *J* = 7.9)
5	40.0, CH_2	2.34 (1H, dd, *J* = 7.9, 14.1)
		1.97 (1H, m)
6	37.5, C	
7	33.2, CH	1.64 (1H, m)
8	31.5, CH_2	2.08 (1H, m)
		1.79 (1H, m)
9	114.9, CH	5.39 (1H, t, *J* = 3.5)
10	147.2, C	
11	41.4, CH	2.04 (1H, m)
12	29.9, CH_2	1.80 (2H, m)
13	23.1, CH_2	1.64 (1H, m)
		0.92 (1H, m)
14	42.7, CH_2	1.48 (1H, m)
		1.21 (1H, m)
15	36.9, C	
16	29.7, CH_3	1.09 (3H, s)
17	26.6, CH_3	1.03 (3H, s)
18	22.8, CH_3	0.90 (3H, s)
19	15.0, CH_3	0.87 (3H, d, *J* = 6.8)
20	20.5, CH_3	1.89 (3H, s)

Table S11 ^{1}H NMR (400 MHz) and ^{13}C NMR (100 MHz) assignments for **2a** in $CDCl_3$.

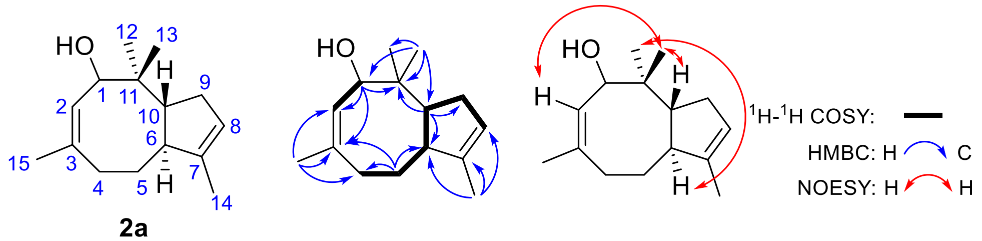

Position	$\delta_{C,}$ type	δ_H (mult, *J* in Hz)
1	75.7, CH	4.26 (1H, d, *J* = 8.7)
2	126.4, CH	5.22 (1H, d, *J* = 8.7)
3	138.3, C	
4	33.3, CH_2	2.35 (1H, m)
		2.00 (1H, m)
5	32.0, CH_2	0.94 (1H, m)
		2.00 (1H, m)
6	51.3, CH	2.33 (1H, m)
7	142.1, C	
8	123.6, CH	5.21 (1H, m)
9	34.1, CH_2	2.13 (1H, m)
		2.26 (1H, m)
10	50.6, CH	2.06 (1H, dt, *J* = 10.0, 6.1)
11	41.3, C	
12	18.3, CH_3	0.89 (3H, s)
13	19.4, CH_3	0.79 (3H, s)
14	15.2, CH_3	1.62 (3H, s)
15	25.1, CH_3	1.78 (3H, s)

Table S12 ^{1}H NMR (400 MHz) and ^{13}C NMR (100 MHz) assignments for **3a** in methanol-d_4.

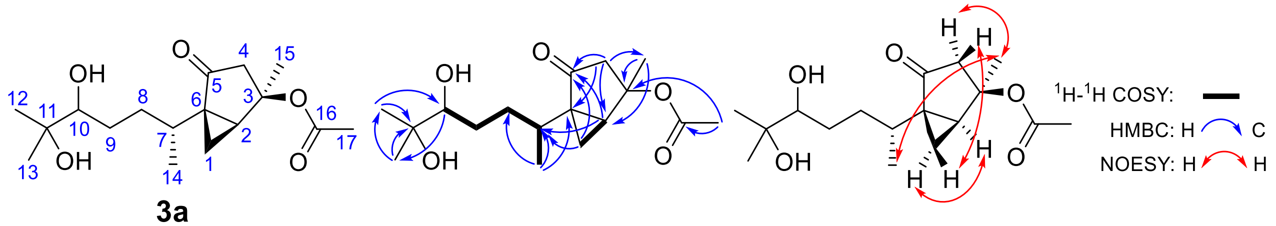

3a

Position	δ_C, type	δ_H (mult, J in Hz)
1	18.5, CH_2	1.39 (1H, dd, J = 5.3, 4.4)
		1.26 (1H, m)
2	37.6, CH	2.43 (1H, dd, J = 7.7, 4.4)
3	81.6, C	
4	48.5, CH_2	2.62(1H, d, J = 18.2)
		2.30(1H, d, J = 18.2)
5	211.6, C	
6	46.1, C	
7	33.9, CH	1.58 (1H, m)
8	32.5, CH_2	1.67 (1H, m)
		1.56 (1H, m)
9	30.4, CH_2	1.56 (1H, m)
		1.31 (1H, m)
10	79.4, CH	3.21 (1H, dd, J = 10.5, 1.4)
11	73.8, C	
12	25.7, CH_3	1.15 (3H, s)
13	24.9, CH_3	1.12 (3H, s)
14	17.3, CH_3	0.97 (3H, d, J = 6.6)
15	25.9, CH_3	1.65 (3H, s)
16	172.3, C	
17	21.6, CH_3	2.00 (3H, s)

Table S13 ^{1}H NMR (400 MHz) and ^{13}C NMR (100 MHz) assignments for **3b** in methanol-d_4.

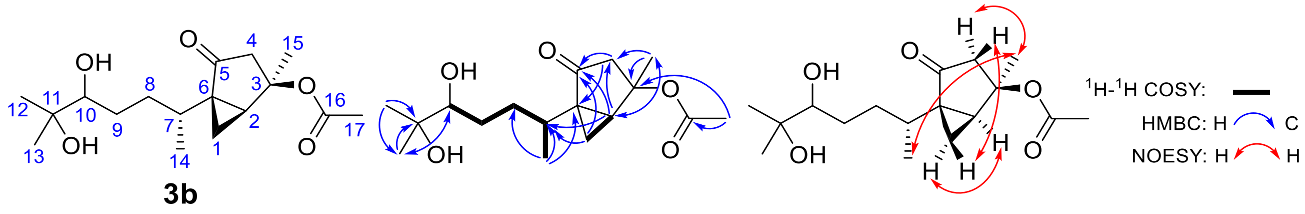

Position	δ_{C}, type	δ_{H} (mult, J in Hz)
1	18.5, CH_2	1.40 (1H, dd, J =5.1, 4.3)
		1.28 (1H, m)
2	37.8, CH	2.45 (1H, dd, J = 7.7, 4.3)
3	81.6, C	
4	48.5, CH_2	2.62 (1H, d, J = 18.2),
		2.30 (1H, d, J = 18.2)
5	211.5, C	
6	46.1, C	
7	34.3, CH	1.57 (1H, m)
8	32.6, CH_2	1.84 (1H, m)
		1.38 (1H, m)
9	30.7, CH_2	1.70 (1H, m)
		1.20 (1H, m)
10	79.9, CH	3.20 (1H, dd, J = 10.5, 1.8)
11	73.8, C	
12	25.8, CH_3	1.15 (3H, s)
13	24.8, CH_3	1.12 (3H, s)
14	17.7, CH_3	0.99 (3H, d, J = 6.9)
15	25.9, CH_3	1.65 (3H, s)
16	172.3, C	
17	21.6, CH_3	2.00 (3H, s)

Table S14 ^{1}H NMR (400 MHz) and ^{13}C NMR (100 MHz) assignments for **3c** in methanol-d_4.

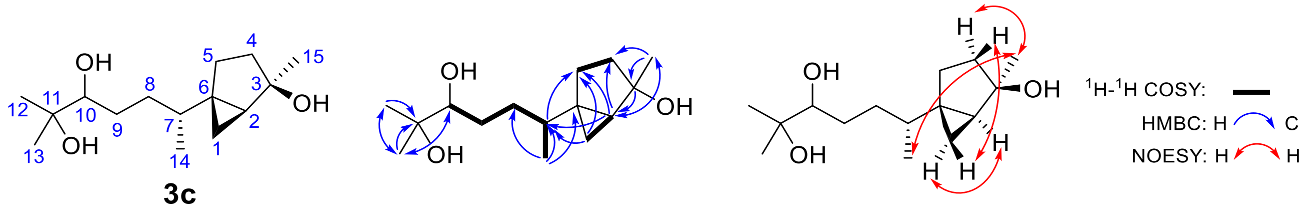

Position	δ_{C}, type	δ_{H} (mult, J in Hz)
1	14.3, CH_2	0.80 (1H, m)
		0.36 (1H, dd, J = 5.2, 7.7)
2	33.9, CH	0.97 (1H, m)
3	79.8, C	
4	36.4, CH_2	1.42 (2H, m),
	25.5, CH_2	1.67 (1H, m)
5		1.58 (1H, m)
6	33.3, C	
7	39.7, CH	1.08 (1H, m)
8	33.2, CH_2	1.46 (2H, m)
9	26.9, CH_2	1.68 (2H, m)
10	79.8, CH	3.21 (1H, dd, J = 1.4, 10.3)
11	73.8, C	
12	25.7, CH_3	1.16 (3H, s)
13	24.9, CH_3	1.13 (3H, s)
14	17.8, CH_3	0.97 (3H, d, J = 6.7)
15	28.6, CH_3	1.30 (3H, s)

Table S15 ^{1}H NMR (400 MHz) and ^{13}C NMR (100 MHz) assignments for **3e** in methanol-d_4.

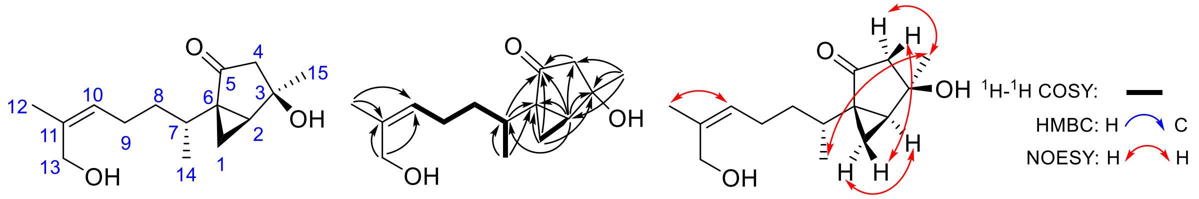

Position	$\delta_{C,}$ type	δ_H (mult, J in Hz)
1	18.9, CH_2	1.42 (1H, m)
		1.20 (1H, ddd, J = 1.5, 5.3, 7.1)
2	39.3, CH	1.99 (1H, m)
3	72.3, C	
4	49.3, CH_2	2.39 (1H, d, J = 17.6),
		1.97 (1H, m)
5	213.0, C	
6	46.3, C	
7	33.9, CH	1.51 (1H, m)
8	35.7, CH_2	1.58 (1H, m)
		1.43 (1H, m)
9	26.9, CH_2	2.07 (2H, m)
10	128.7, CH	5.25 (1H, t, J = 7.2)
11	135.8, C	
12	21.5, CH_3	1.76 (3H, s)
13	61.3, CH_2	4.06 (2H, s)
14	17.4, CH_3	0.96 (3H, d, J = 6.7)
15	29.7, CH_3	1.40 (3H, s)

Table S16 ^{1}H NMR (400 MHz) and ^{13}C NMR (100 MHz) assignments for **4a** in $CDCl_3$.

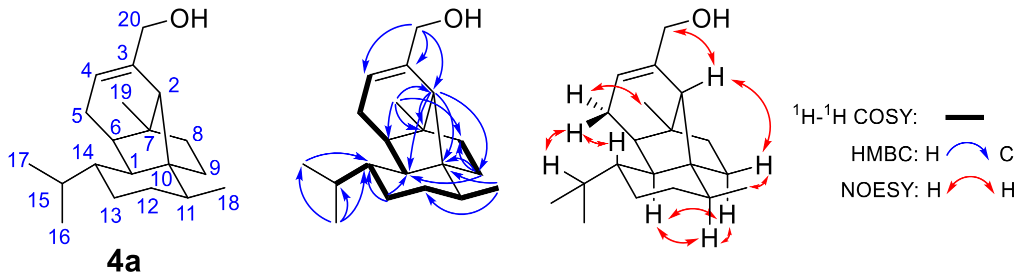

4a

Position	$\delta_{C,}$ type	δ_H (mult, *J* in Hz)
1	47.9, CH	1.38 (1H, m)
2	49.1, CH	1.60 (1H, s)
3	139.9, C	
4	122.2, CH	5.56 (1H, m)
5	27.4, CH_2	2.14 (1H, m)
		1.85 (1H, m)
6	45.2, CH	1.75 (1H, m)
7	43.5, C	
8	34.8, CH_2	1.46 (2H, m)
9	40.5, CH_2	0.90 (1H, m)
		1.85 (1H, m)
10	58.5, C	
11	37.6, CH	1.41 (1H, m)
12	31.7, CH_2	0.94 (1H, m)
		1.41 (1H, m)
13	24.9, CH_2	1.55(1H, m)
		0.81 (1H, m)
14	41.7, CH	1.39 (1H, m)
15	30.5, CH	1.44 (1H, m)
16	21.3, CH_3	0.87 (3H, d, *J* = 6.8)
17	15.6, CH_3	0.72 (3H, d, *J* = 6.8)
18	18.1, CH_3	1.02 (3H, d, *J* = 6.8)
19	18.5, CH_3	0.99 (3H, s)
20	67.1, CH_2	4.14 (1H, dd, *J* = 13.3, 1.2)
		4.06 (1H, dd, *J* = 13.3, 1.2)

Table S17 ^{1}H NMR (400 MHz) and ^{13}C NMR (100 MHz) assignments for **5a** in $CDCl_3$.

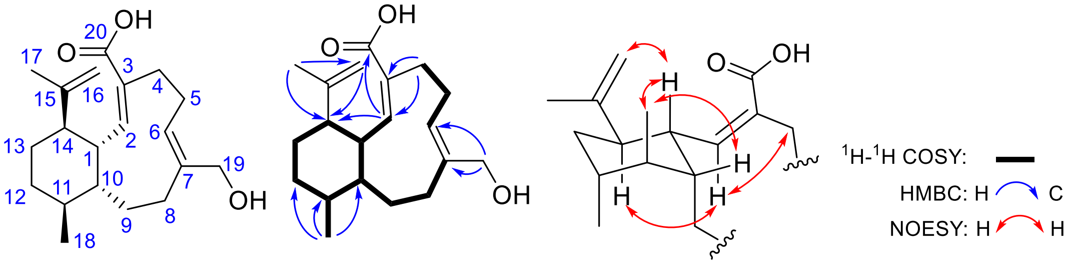

5a

Position	δ_C, type	δ_H (mult, J in Hz)
1	36.8, CH	3.47 (1H, m)
2	158.8, CH	5.78 (1H, d, J = 11.0)
3	127.0, C	
4	34.6, CH_2	1.98 (1H, m)
		2.78 (1H, m)
5	25.2, CH_2	2.56 (1H, m)
		2.19 (1H, m)
6	131.8, CH	5.15 (1H, m)
7	140.8, C	
8	30.4, CH_2	2.24 (1H, m)
		1.97 (1H, m)
9	33.0, CH_2	1.71 (1H, m)
		1.44 (1H, m)
10	45.3, CH	1.56 (1H, m)
11	34.4, CH	1.72 (1H, m)
12	26.9, CH_2	1.71 (1H, m)
		1.38 (1H, m)
13	25.9, CH_2	1.37 (1H, m)
		1.38 (1H, m)
14	46.7, CH	1.96 (1H, m)
15	149.4, C	
16	110.8, CH_2	4.64 (1H, br)
		4.68 (1H, br)
17	19.1, CH_3	1.60 (3H, s)
18	18.6, CH_3	1.11 (3H, d, J = 7.1)
19	63.3, CH_2	4.08 (1H, d, J = 10.8)
		3.84 (1H, d, J = 11.7)
20	172.1, C	

Table S18 ^{1}H NMR (400 MHz) and ^{13}C NMR (100 MHz) assignments for **6a** in $CDCl_3$.

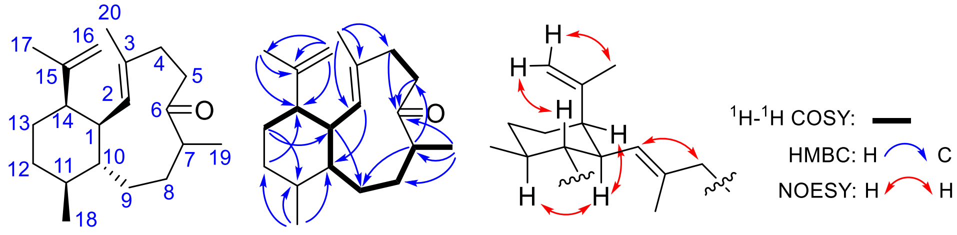

6a

Position	δ_C, type	δ_H (mult, J in Hz)
1	46.3, CH	2.04 (1H, td, J = 11.1, 5.5)
2	130.8, CH	5.39 (1H, d, J = 11.1)
3	133.4, C	
4	33.6, CH_2	2.50 (1H, m)
		2.28 (1H, m)
5	39.5, CH_2	2.94 (1H, m)
		2.26 (1H, m)
6	217.2, C	
7	46.0, CH	2.84 (1H, m)
8	34.2, CH_2	1.84 (1H, m)
		1.25 (1H, m)
9	29.6, CH_2	1.25 (1H, m)
		0.67 (1H, m)
10	45.0,CH	0.95 (1H, m)
11	39.0, CH	1.11 (1H, m)
12	30.4, CH_2	1.41 (1H, dq, J = 13.4, 4.1)
		1.26 (1H, m)
13	29.7, CH_2	1.67 (1H, m)
		1.54 (1H, m)
14	44.3, CH	2.24 (1H, m)
15	147.4, C	
16	112.6, CH_2	4.91 (1H, br)
		4.83 (1H, br)
17	26.6, CH_3	1.72 (3H, s)
18	20.8, CH_3	0.91 (3H, d, J = 6.3)
19	17.3, CH_3	0.93 (3H, d, J = 7.0)
20	18.9, CH_3	1.56 (3H, d, J = 1.0)

Table S19 ^{1}H NMR (400 MHz) and ^{13}C NMR (100 MHz) assignments for **6b** in $CDCl_3$.

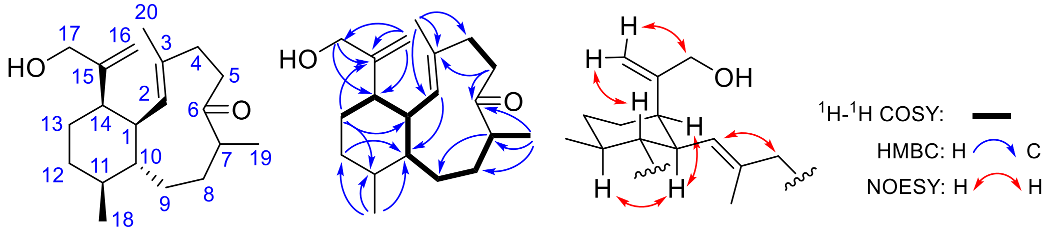

6b

Position	δ_C, type	δ_H (mult, J in Hz)
1	45.9, CH	2.06 (1H, td, J = 11.0, 5.5)
2	130.5, CH	5.29 (1H, d, J = 11.0)
3	134.2, C	
4	33.6, CH_2	2.47 (1H, m)
		2.28 (1H, m)
5	39.5, CH_2	2.93 (1H, m)
		2.24 (1H, m)
6	217.2, C	
7	45.9, CH	2.84 (1H, m)
8	34.3, CH_2	1.86 (1H, m)
		1.25 (1H, m)
9	29.7, CH_2	1.25 (1H, m)
		0.69 (1H, m)
10	45.5,CH	0.92 (1H, m)
11	38.9, CH	1.11 (1H, m)
12	30.2, CH_2	1.45 (1H, m)
		1.25 (1H, m)
13	30.1, CH_2	1.61 (2H, m)
14	39.6, CH	2.21 (1H, m)
15	150.5, C	
16	110.9, CH_2	5.26 (1H, br)
		5.16 (1H, br)
17	67.9, CH_2	3.94 (2H, m)
18	20.8, CH_3	0.94 (3H, d, J = 6.2)
19	17.4, CH_3	0.93 (3H, d, J = 7.0)
20	19.0, CH_3	1.57 (3H, d, J = 1.2)

Table S20 ^{1}H NMR (400 MHz) and ^{13}C NMR (100 MHz) assignments for **9a** in $CDCl_3$.

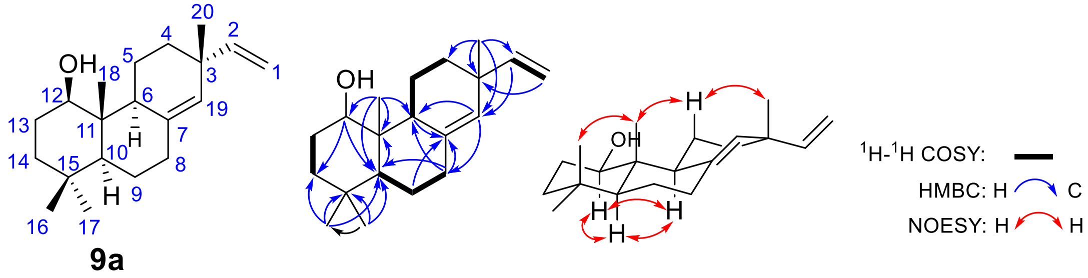

Position	δ_C, type	δ_H (mult, J in Hz)
1	110.7, CH_2	4.93 (1H, dd, J = 1.5, 10.2)
		4.87 (1H, dd, J = 1.5, 17.8)
2	148.5 CH	5.77 (1H, dd, J = 10.2, 17.8)
3	37.9, C	
4	34.1, CH_2	1.45 (2H, m)
5	20.8, CH_2	1.79 (1H, m)
		1.61 (1H, m)
6	48.7, CH	2.16 (1H, m)
7	135.7, C	
8	35.3, CH_2	2.38 (1H, ddd, J =1.7, 4.6, 14.7)
		2.20 (1H, m)
9	20.8, CH_2	1.76 (1H, m)
		1.49 (1H, m)
10	52.4, CH	1.74 (1H, dd, J = 3.3, 13.0)
11	46.4, C	
12	83.4, CH	4.13 (1H, m)
13	29.6 CH_2	1.34 (1H, m)
		1.27 (1H, m)
14	31.7, CH_2	1.30 (1H, m)
15	42.1, C	
16	28.7, CH_3	1.10 (3H, s)
17	20.9, CH_3	1.03 (3H, s),
18	9.4, CH_3	0.65 (3H, s)
19	131.2, CH	5.32 (1H, s)
20	26.6, CH_3	1.05 (3H, s)

Table S21 ^{1}H NMR (400 MHz) and ^{13}C NMR (100 MHz) assignments for **10a** in $CDCl_3$.

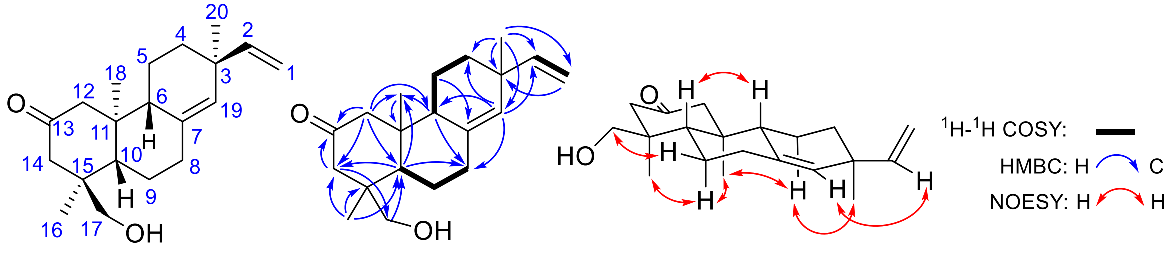

10a

Position	δ_C, type	δ_H (mult, J in Hz)
1	110.6, CH_2	4.92 (1H, dd, J = 1.2, 10.2)
		4.89 (1H, br)
2	148.5 CH	5.76 (1H, dd, J = 10.2, 17.8)
3	37.5, C	
4	34.3, CH_2	1.46 (1H, m)
		1.40 (1H, m)
5	18.9, CH_2	1.58 (1H, m)
		1.52 (1H, m)
6	50.1, CH	2.02 (1H, m)
7	135.8, C	
8	35.4, CH_2	2.33 (1H, m)
		2.16 (1H, m)
9	22.7, CH_2	1.62 (1H, m)
		1.42 (1H, m)
10	46.7, CH	2.00 (1H, m)
11	43.9, C	
12	53.7, CH_2	2.38 (1H, m)
		2.24 (1H, m)
13	212.7, C	
14	50.5, CH_2	2.74 (1H, d, J = 13.1)
		1.98 (1H, m)
15	43.6, C	
16	19.7, CH_3	0.82 (3H, s)
17	70.2, CH_2	3.58 (1H, d, J = 10.8),
		3.07 (1H, d, J = 10.8)
18	16.2, CH_3	0.87 (3H, s)
19	130.1, CH	5.31 (1H, s)
20	26.4, CH_3	1.03 (3H, s)

Table S22 ^{1}H NMR (400 MHz) and ^{13}C NMR (100 MHz) assignments for **10b** in $CDCl_3$.

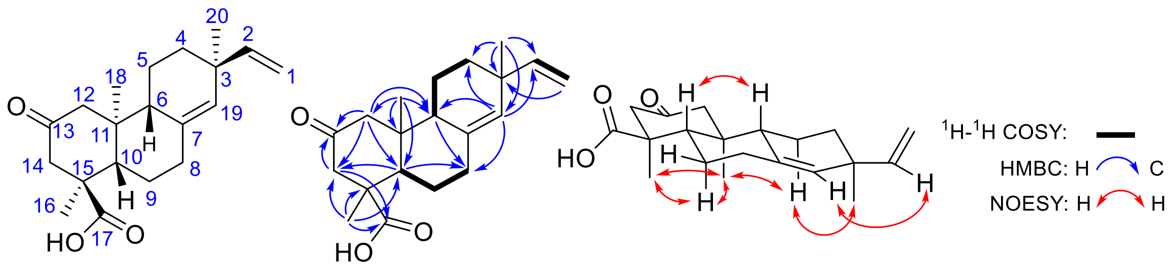

10b

Position	δ_C, type	δ_H (mult, J in Hz)
1	110.7, CH_2	4.93 (1H, dd, J = 1.4, 10.2)
		4.89 (1H, dd, J = 1.4, 17.8)
2	148.5, CH	5.76 (1H, dd, J = 10.2, 17.8)
3	37.6, C	
4	34.2, CH_2	1.47 (2H, m)
5	18.8, CH_2	1.57 (1H, m)
		1.43 (1H, m)
6	50.4, CH	2.07. (1H, m)
7	134.7, C	
8	35.1, CH_2	2.32 (1H, m)
		2.20 (1H, m)
9	24.3, CH_2	1.53 (2H, m)
10	49.4, CH	2.42 (1H, m)
11	43.9, C	
12	53.4, CH_2	2.45 (1H, m)
		2.35 (1H, m)
13	209.3, C	
14	51.2, CH_2	3.00 (1H, d, J = 13.2)
		2.34 (1H, m)
15	51.1, C	
16	18.6, CH_3	1.22(3H, s)
17	180.7, C	
18	15.9, CH_3	0.88 (3H, s)
19	130.7, CH	5.32 (1H, s)
20	26.4, CH_3	1.04 (3H, s)

Table S23 ^{1}H NMR (400 MHz) and ^{13}C NMR (100 MHz) assignments for **10c** in $CDCl_3$.

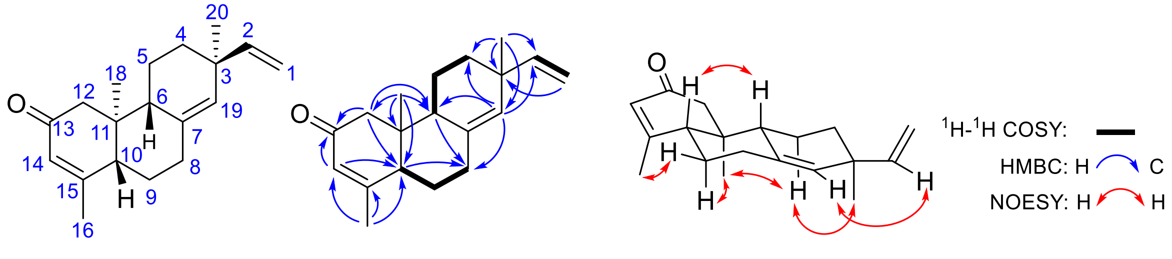

10c

Position	$\delta_{C,}$ type	δ_H (mult, J in Hz)
1	110.7, CH_2	4.94 (1H, dd, J = 1.3, 10.7)
		4.90 (1H, dd, J = 1.3, 17.0)
2	148.5, CH	5.77 (1H, dd, J = 10.7, 17.0)
3	37.6, C	
4	34.3, CH_2	1.46 (2H, m)
5	19.1, CH_2	1.67 (1H, m)
		1.53 (1H, m)
6	47.3, CH	2.04 (1H, t, J = 7.5)
7	134.7, C	
8	35.2, CH_2	2.37 (2H, m)
9	24.1, CH_2	1.41 (2H, m)
10	49.5, CH	2.45 (H, d, J = 12.2)
11	41.9, C	
12	51.2, CH_2	2.22 (1H, d, J = 15.9)
		2.57 (1H, d, J = 15.9)
13	199.3, C	
14	126.8, CH	5.91 (1H, s)
15	163.2, C	
16	22.5, CH_3	1.90 (3H, s)
18	14.3, CH_3	0.80 (3H, s)
19	130.7, CH	5.34 (1H, s)
20	26.1, CH_3	1.05 (3H, s)

Table S24 ^{1}H NMR (400 MHz) and ^{13}C NMR (100 MHz) assignments for **10d** in $CDCl_3$.

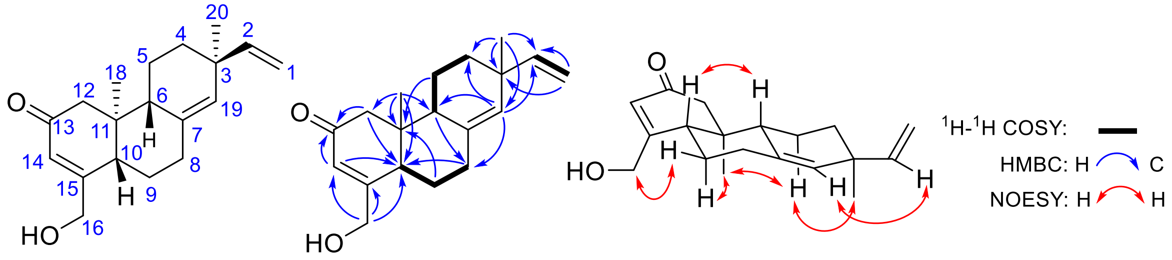

10d

Position	δ_{C}, type	δ_{H} (mult, J in Hz)
1	110.8, CH_2	4.93 (1H, dd, J = 1.3, 10.2)
		4.89 (1H, dd, J = 1.3, 17.9)
2	148.3, CH	5.77 (1H, dd, J = 10.2, 17.9)
3	37.6, C	
4	34.2, CH_2	1.46 (2H, m)
5	19.2, CH_2	1.67 (1H, m)
		1.51 (1H, m)
6	47.2, CH	2.06. (1H, m)
7	134.3, C	
8	35.0, CH_2	2.34 (1H, m)
		2.18 (1H, m)
9	23.0, CH_2	1.84 (1H, m)
		1.42 (1H, m)
10	47.6, CH	2.56 (1H, m)
11	42.1, C	
12	51.1, CH_2	2.58 (1H, d, J = 15.9)
		2.22 (1H, d, J = 15.9)
13	199.5, C	
14	123.0, CH	6.20 (1H, s)
15	164.0, C	
16	63,3, CH_2	4.29 (2H, m)
18	14.3, CH_3	0.81 (3H, s)
19	130.9, CH	5.34 (1H, s)
20	26.1, CH_3	1.04 (3H, s)

Table S25 ^{1}H NMR (400 MHz) and ^{13}C NMR (100 MHz) assignments for **11a** in methanol-d_4.

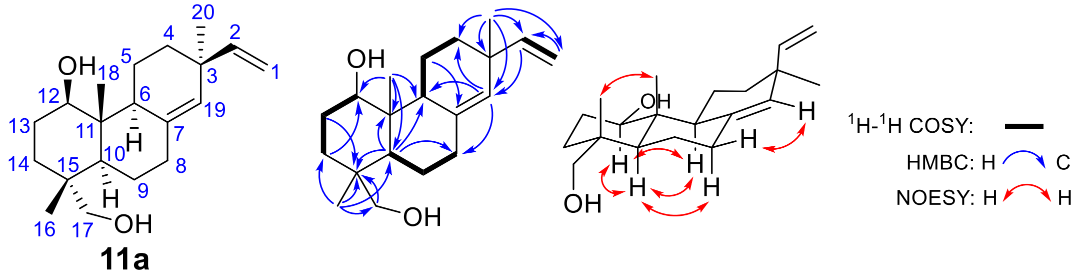

Position	δ_C, type	δ_H (mult, *J* in Hz)
1	113.4, CH_2	4.95 (1H, dd, *J* = 10.5, 2.0)
		4.91 (1H, dd, *J* = 17.2, 2.0)
2	148.5, CH	5.72 (1H, dd *J* = 10.5, 17.2)
3	39.4, C	
4	37.3, CH_2	1.54 (1H, m)
		1.19 (1H, m)
5	23.7, CH_2	1.92 (1H, m)
		1.63 (1H, m)
6	53.6, CH	1.93. (1H, m)
7	140.0, C	
8	37.1, CH_2	2.29 (1H, m)
		2.12 (1H, m)
9	23.2, CH_2	1.53 (1H, m)
		1.47 (1H, m)
10	48.1, CH	1.44 (1H, m)
11	45.1, C	
12	79.7, CH	3.36 (1H, m)
13	34.7, CH_2	1.65 (1H, m)
		1.25 (1H, m)
14	30.0, CH_2	1.61 (2H, m)
15	38.6, C	
16	18.1, CH_3	0.77 (3H, s)
17	71.7, CH_2	3.35 (1H, d, *J* = 11.0)
		3.01 (1H, d, *J* = 11.0)
18	10.1, CH_3	0.82 (3H, s)
19	129.8, CH	5.15 (1H, s)
20	30.1, CH_3	0.95 (3H, s)

Table S26 ^{1}H NMR (400 MHz) and ^{13}C NMR (100 MHz) assignments for **11b** in methanol-d_4.

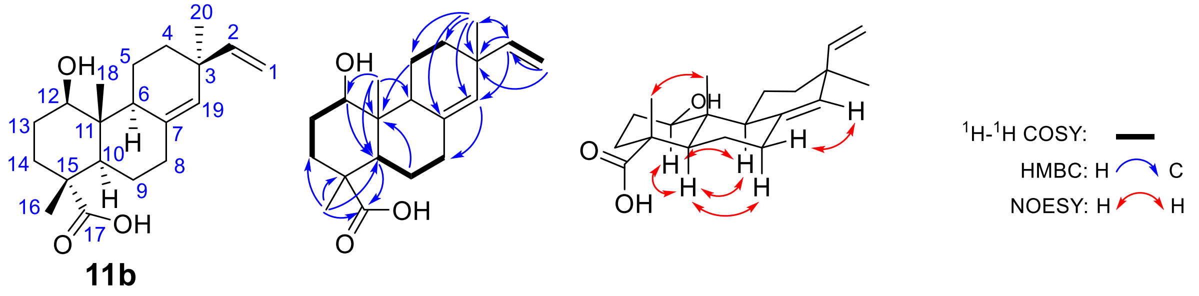

Position	δ_{C}, type	δ_{H} (mult, *J* in Hz)
1	113.6, CH_2	4.95 (1H, dd, *J* = 2.1, 10.4)
		4.90 (1H, dd, *J* = 2.1, 17.2)
2	148.4, CH	5.73 (1H, dd, *J* = 10.4, 17.2)
3	39.4, C	
4	37.2, CH_2	1.55 (1H, m)
		1.21 (1H, m)
5	23.7, CH_2	1.93 (1H, m)
		1.67 (1H, m)
6	53.8, CH	1.97. (1H, m)
7	139.5, C	
8	37.1, CH_2	2.29 (1H, m)
		2.11 (1H, m)
9	25.9, CH_2	1.62 (1H, m)
		1.26 (1H, m)
10	50.4, CH	1.92 (1H, m)
11	45.0, C	
12	79.2, CH	3.48 (1H, dd, *J* = 6.2, 9.6)
13	29.7, CH_2	1.64, (2H, m)
14	36.3, CH_2	1.90 (1H, m)
		1.55 (1H, m)
15	48.4, C	
16	17.2, CH_3	1.17 (3H, s)
17	182.3, C	
18	9.9, CH_3	0.82 (3H, s)
19	130.3, CH	5.17 (1H, s)
20	30.1, CH_3	0.96 (3H, s)

Table S27 ^{1}H NMR (400 MHz) and ^{13}C NMR (100 MHz) assignments for **11c** in $CDCl_3$.

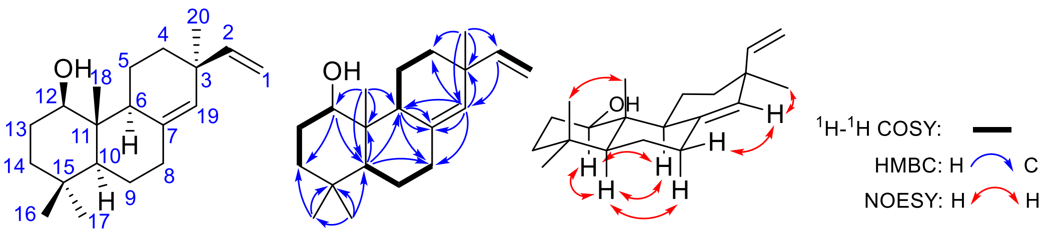

11c

Position	δ_{C}, type	δ_{H} (mult, *J* in Hz)
1	113.2, CH_2	4.96 (1H, dd, *J* = 2.0, 10.4)
		4.92 (1H, dd, *J* = 2.0, 17.3)
2	147.3, CH	5.72 (1H, dd, *J* = 10.4, 17.3)
3	38.5, C	
4	36.1, CH_2	1.57 (1H, m)
		1.22 (1H, m)
5	23.0, CH_2	1.81 (1H, m)
		1.60 (1H, m)
6	52.1, CH	1.90. (1H, m)
7	138.3, C	
8	36.3, CH_2	2.32 (1H, ddd, *J* =1.9, 4.5, 14.2)
		2.04 (1H, ddd, *J* =1.9, 4.5, 14.2)
9	22.6, CH_2	1.59 (1H, m)
		1.46 (1H, m)
10	54.3, CH	1.01 (1H, dd, *J* =2.6, 12.4)
11	44.4, C	
12	79.3, CH	3.45 (1H, dd, *J* = 5.8, 10.0)
13	29.8, CH_2	1.58, (2H, m)
14	39.9, CH_2	1.38 (1H, m)
		1.33 (1H, m)
15	33.4, C	
16	33.3, CH_3	0.87 (3H, s)
17	21.8, CH_3	0.83 (3H, s)
18	8.9, CH_3	0.77 (3H, s)
19	129.0, CH	5.17 (1H, s)
20	29.6, CH_3	0.98 (3H, s)

Table S28 ^{1}H NMR (400 MHz) and ^{13}C NMR (100 MHz) assignments for **12b** in $CDCl_3$.

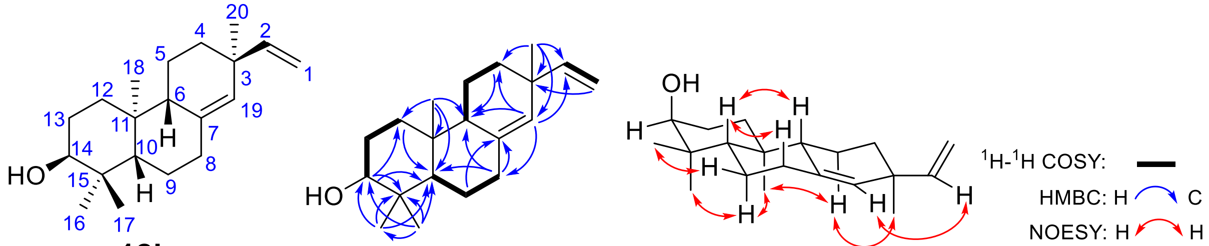

12b

Position	δ_{C}, type	δ_{H} (mult, J in Hz)
1	110.1, CH_2	4.90 (1H, dd, J = 1.4, 17.1)
		4.86 (1H, dd, J = 1.4, 10.5)
2	149.1, CH	5.76 (1H, dd J = 10.5, 17.1)
3	37.5, C	
4	34.7, CH_2	1.44 (1H, m)
		1.34 (1H, m)
5	18.8, CH_2	1.61 (1H, m)
		1.45 (1H, m)
6	50.4, CH	1.79 (1H, t, J = 7.8)
7	137.1, C	
8	36.0, CH_2	2.24 (1H, m)
		2.07 (1H, m)
9	22.3, CH_2	1.48 (1H, m)
		1.34 (1H, m)
10	48.3, CH	1.46 (1H, m)
11	37.6, C	
12	31.7, CH_2	1.53 (1H, m)
		1.45 (1H, m)
13	25.5, CH_2	1.89 (1H, m)
		1.60 (1H, m)
14	76.3, CH	3.46 (1H, s)
15	38.0, C	
16	28.6, CH_3	0.95 (3H, m)
17	22.8, CH_3	0.86 (3H, s)
18	15.0, CH_3	0.81 (3H, s)
19	128.8, CH	5.21 (1H, s)
20	26.1, CH_3	1.03 (3H, s)

Table S29 ^{1}H NMR (400 MHz) and ^{13}C NMR (100 MHz) assignments for **12c** in acetone-d_6.

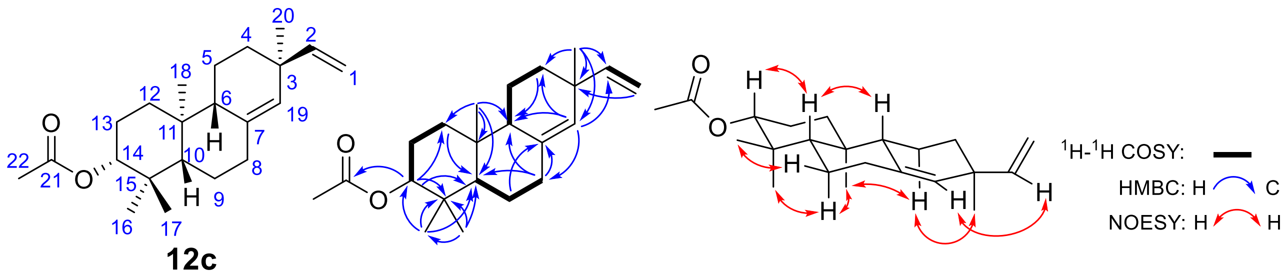

Position	δ_C, type	δ_H (mult, J in Hz)
1	110.6, CH_2	4.91 (1H, dd J = 1.5, 17.5)
		4.86 (1H, dd, J = 1.5, 10.6)
2	149.6, CH	5.77 (1H, dd J = 10.6, 17.5)
3	38.1, C	
4	35.3, CH_2	1.48, 1.38 (2H, m)
5	19.5, CH_2	1.62 (2H, m)
6	51.0, CH	1.78. (1H, m)
7	137.4, C	
8	36.4, CH_2	2.31 (1H, m)
		2.10 (1H, m)
9	22.8, CH_2	1.64 (1H, m)
		1.40 (1H, m)
10	54.9, CH	1.22 (1H, m)
11	38.6, C	
12	37.6, CH_2	1.80 (1H, m)
		1.30 (1H, m)
13	25.8, CH_2	1.63 (2H, m)
14	80.9, CH	4.52 (H, dd, J = 5.1, 10.9)
15	38.7, C	
16	28.8, CH_3	0.90 (3H, s)
17	17.4, CH_3	0.93 (3H, s)
18	15.4, CH_3	0.86 (3H, s)
19	137.4, C	
20	26.4, CH_3	1.04 (3H, s)
21	170.2, C	
22	23.0, CH_3	0.88 (3H, s)

III. Physicochemical data of compounds.

Compound 1: colorless oil, $[\alpha]_D^{25}$= +32.0 (*c* 1.2, $CHCl_3$); EI mass spectrum is showed in Figure S8. ^1H NMR (400 MHz), ^{13}C NMR (100 MHz) data and optical rotation of **1** are in agreement with the reported guaia-1(10),11-diene[34].

Compound 2: colorless oil, $[\alpha]_D^{25}$= + 25.2 (*c* 2.5, $CHCl_3$); NMR data in $CDCl_3$ is showed in Table S6; EI mass spectrum is showed in Figure S16.

Compound 3: colorless oil, $[\alpha]_D^{25}$= + 85.4 (*c* 1.5, $CHCl_3$); EI mass spectrum is showed in Figure S19. ^1H NMR (400 MHz), ^{13}C NMR (100 MHz) data and optical rotation of **3** are in agreement with the reported 7-*epi*-*cis*-sesquisabinene hydrate[35].

Compound 4: colorless oil, $[\alpha]_D^{25}$= + 10.2 (*c* 1.2, $CHCl_3$); EI mass spectrum is showed in Figure S22. ^1H NMR (400 MHz), ^{13}C NMR (100 MHz) data and optical rotation of **4** are in agreement with the reported spiroalbatene[26].

Compound 5: colorless oil, $[\alpha]_D^{25}$= + 38.9 (*c* 1.1, n-hexane); EI mass spectrum is showed in Figure S25, ^1H NMR (400 MHz), ^{13}C NMR (100 MHz) data and optical rotation of **5** are in agreement with the reported benditerpe-2,6,15-triene[33].

Compound 5′: colorless oil, $[\alpha]_D^{25}$= + 51.7 (*c* 1.6, n-hexane); NMR data in $CDCl_3$ is showed in Table S7; EI mass spectrum is showed in Figure S33.

Compound 6′: colorless oil, $[\alpha]_D^{25}$= – 66.8 (*c* 2.1, n-hexane); NMR data in $CDCl_3$ is showed in Table S8; EI mass spectrum is showed in Figure S49.

Compound 6: colorless oil, $[\alpha]_D^{25}$= – 22.8 (*c* 2.5, n-hexane); NMR data in $CDCl_3$ is showed in Table S9; EI mass spectrum is showed in Figure S41.

Compound 7: colorless oil, $[\alpha]_D^{25}$= + 10.5 (*c* 1.3, $CHCl_3$); EI mass spectrum is showed in Figure S52. ^1H NMR (400 MHz), ^{13}C NMR (100 MHz) data and optical rotation of **7** are in agreement with the reported (*S*)-(+)-cembrene A[36].

Compound 8: colorless oil, $[\alpha]_D^{25}$= – 78.2 (*c* 0.9, $CHCl_3$); NMR data in $CDCl_3$ is showed in Table S10; EI mass spectrum is showed in Figure S60.

Compound 9: colorless oil, $[\alpha]_D^{25}$= – 20.5 (*c* 0.7, $CHCl_3$); EI mass spectrum is showed in Figure S63 ^1H NMR (400 MHz), ^{13}C NMR (100 MHz) data and optical rotation of **9** are in agreement with the reported sandaracopimaradiene[37].

Compound 10: colorless oil, $[\alpha]_D^{25}$= + 16.1 (*c* 0.6, $CHCl_3$); EI mass spectrum is showed in Figure S66. ^1H NMR (400 MHz), ^{13}C NMR (100 MHz) data and optical rotation of **10** are in agreement with the reported *ent*-sandaracopimaradiene[38].

Compound 11: colorless oil, $[\alpha]_D^{25}$= + 91.2 (*c* 1.3, $CHCl_3$); EI mass spectrum is showed in Figure S69. ^1H NMR (400 MHz), ^{13}C NMR (100 MHz) data and optical rotation of **11** are in agreement with the reported pimara-8(14),15-diene[39].

Compound 2a: colorless oil, $[\alpha]_D^{25}$= + 150.6 (*c* 0.9, $CHCl_3$); NMR data in $CDCl_3$ is showed in Table S11; EI mass spectrum is showed in Figure S77.

Compound 3a: yellow powder, $[\alpha]_D^{25}$= + 35.3 (*c* 1.3, methanol); NMR data in methanol-d_4 is showed in Table S12; EI mass spectrum is showed in Figure S85; HRESIMS *m/z* 313.2012 $[M+H]^+$ (calcd. for $C_{17}H_{28}O_5$, 313.2010).

Compound 3b: yellow powder, $[\alpha]_D^{25}$= + 16.2 (*c* 1.4, methanol); NMR data in methanol-d_4 is showed in Table S13; EI mass spectrum is showed in Figure S93; HRESIMS *m/z* 313.2010 $[M+H]^+$ (calcd. for $C_{17}H_{28}O_5$, 313.2010).

Compound 3c: yellow oil, $[\alpha]_D^{25}$= + 130.1 (*c* 0.8, $CHCl_3$); NMR data in $CDCl_3$ is showed in Table S14; EI mass spectrum is showed in Figure S101.

Compound 3e: yellow oil, $[\alpha]_D^{25}$= – 17.5 (*c* 0.5, $CHCl_3$); NMR data in $CDCl_3$ is showed in Table S15; EI mass spectrum is showed in Figure S109; HRESIMS *m/z* 235.1715 $[M-H_2O+H]^+$ (calcd. for $C_{17}H_{28}O_5$, 235.1693).

Compound 4a: colorless oil, $[\alpha]_D^{25}$= + 30.1 (*c* 1.5, $CHCl_3$); NMR data in $CDCl_3$ is showed in Table S16; EI mass spectrum is showed in Figure S117.

Compound 5a: colorless waxy solid, $[\alpha]_D^{25}$= + 6.8 (*c* 1.2, $CHCl_3$); NMR data in $CDCl_3$ is showed in Table S17; EI mass spectrum is showed in Figure S125; HRESIMS *m/z* 301.2164 $[M - H_2O + H]^+$ (calcd for $C_{20}H_{30}O_3$, 301.2162).

Compound 6a: colorless waxy solid, $[\alpha]_D^{25}$= – 13.8 (*c* 1.1, $CHCl_3$); NMR data in $CDCl_3$ is showed in Table S18; EI mass spectrum is showed in Figure S133.

Compound 6b: colorless waxy solid, $[\alpha]_D^{25}$= – 10.7 (*c* 1.5, $CHCl_3$); NMR data in $CDCl_3$ is showed in Table S19; EI mass spectrum is showed in Figure S141.

Compound 9a: colorless waxy solid, $[\alpha]_D^{25}$= – 4.5 (*c* 1.9, $CHCl_3$); NMR data in $CDCl_3$ is showed in Table S20; EI mass spectrum is showed in Figure S149.

Compound 10a: white powder, $[\alpha]_D^{25}$= + 10.8 (*c* 1.3, $CHCl_3$); NMR data in $CDCl_3$ is showed in Table S21; EI mass spectrum is showed in Figure S157.

Compound 10b: white powder, $[\alpha]_D^{25}$= + 22.6 (*c* 0.5, methanol); NMR data in $CDCl_3$ is showed in Table S22; EI mass spectrum is showed in Figure S165; HRESIMS *m/z* 339.1938 [M + Na]$^+$ (calcd. for $C_{20}H_{28}O_3Na$, 339.1931).

Compound 10c: white powder, $[\alpha]_D^{25}$= + 38.8 (*c* 1.0, $CHCl_3$); NMR data in $CDCl_3$ is showed in Table S23; EI mass spectrum is showed in Figure S173.

Compound 10d: white powder, $[\alpha]_D^{25}$= + 59.5 (*c* 1.1, $CHCl_3$); NMR data in $CDCl_3$ is showed in Table S24; EI mass spectrum is showed in Figure S181.

Compound 11a: white powder, $[\alpha]_D^{25}$= + 100.1 (*c* 0.9, methanol); NMR data in methanol-d_4 is showed in Table S25; EI mass spectrum is showed in Figure S189.

Compound 11b: white powder, $[\alpha]_D^{25}$= + 16.0 (*c* 0.2, methanol); NMR data in methanol-d_4 is showed in Table S26; EI mass spectrum is showed in Figure S198; HRESIMS *m/z* 301.2168 [M - H_2O + H]$^+$ (calcd. for $C_{20}H_{30}O_3$, 301.2162).

Compound 11c: white powder, $[\alpha]_D^{25}$= + 92.0 (*c* 0.8, $CHCl_3$); NMR data in methanol-d_4 is showed in Table S27; EI mass spectrum is showed in Figure S206.

Compound 12a: colorless oil, $[\alpha]_D^{25}$= + 52.0 (*c* 0.2, $CHCl_3$); EI mass spectrum is showed in Figure S207. ^1H NMR (400 MHz), ^{13}C NMR (100 MHz) data and optical rotation of **12a** are in agreement with the reported *ent*-isopimara-8(14),15-dien-3*β*-ol[40].

Compound 12b: colorless oil, $[\alpha]_D^{25}$= + 28.0 (*c* 0.2, $CHCl_3$); NMR data in methanol-d_4 is showed in Table S28; EI mass spectrum is showed in Figure S217.

Compound 12c: colorless waxy solid, $[\alpha]_D^{25}$= – 6.4 (*c* 1.7, methanol); NMR data in methanol-d_4 is showed in Table S29; EI mass spectrum is showed in Figure S225; HRESIMS *m/z* 331.2631 [M + H]$^+$ (calcd. for $C_{22}H_{34}O_2$, 331.2632).

IV. Supplementary Figures:

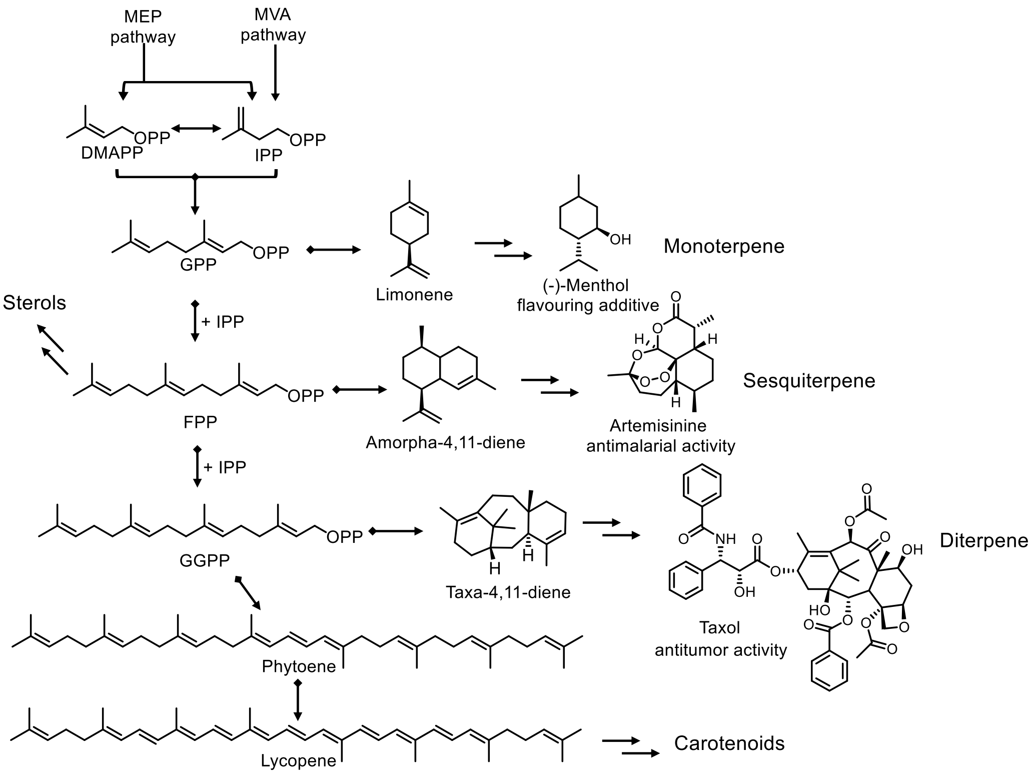

Figure S1 Biosynthesis of terpenoids and the activity of classic compounds containing multistep modifications. In these cases of menthol, artemisinine and taxol, the activity of terpene skeletons is not detectable.

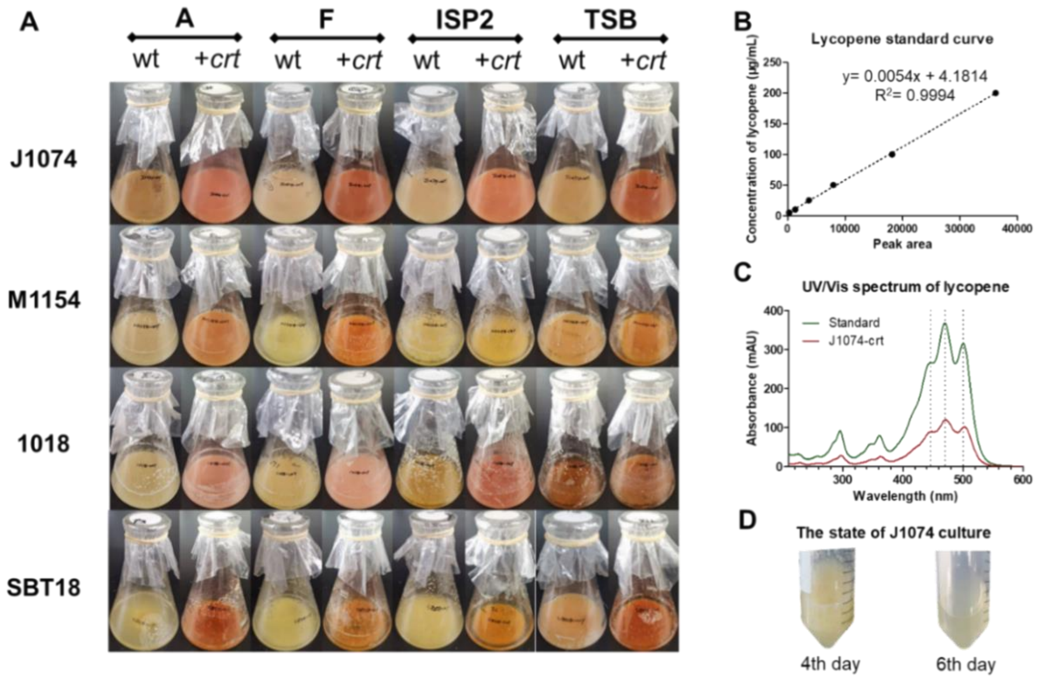

Figure S2 Evaluation of *Streptomyces* expression system. (A) the figures of strains were cultured in liquid mediums with or without lycopene gene cluster. (B) the lycopene standard curve. (C) the UV-Vis spectrum of lycopene, the λ of dotted lines was 444 nm, 470 nm, 500 nm, respectively. (D) the state of *S. albus* J1074 strain was cultured for 4 days and 6 days in medium F.

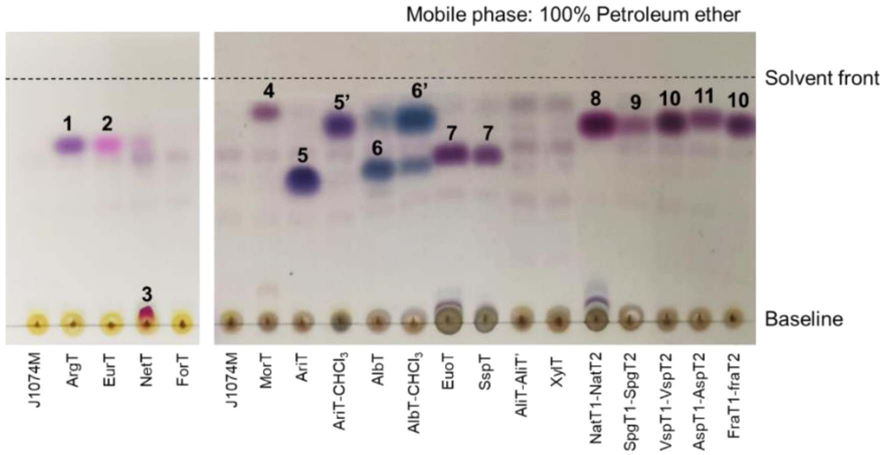

Figure S3 The TLC analysis of organic extracts were produced by *S. albus* J1074M and recombinant strains containing terpene synthases.

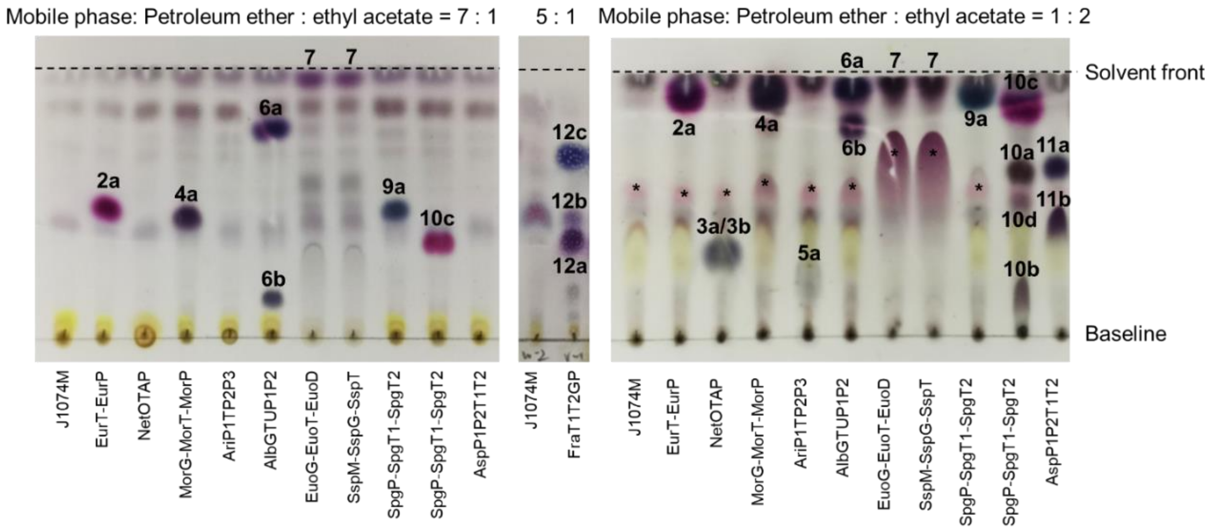

Figure S4 The TLC analysis of organic extracts were produced by *S. albus* J1074M and recombinant strains containing intact terpene BGCs. *: An oily unknown substance produced by chassis cell.

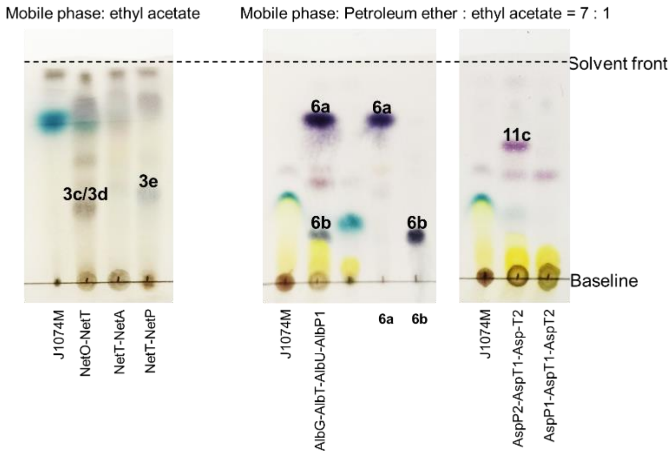

Figure S5 The TLC analysis of organic extracts were produced by *S. albus* J1074M and recombinant strains. The tailoring enzymes in cluster *net*, *alb* and *asp* were expressed individually.

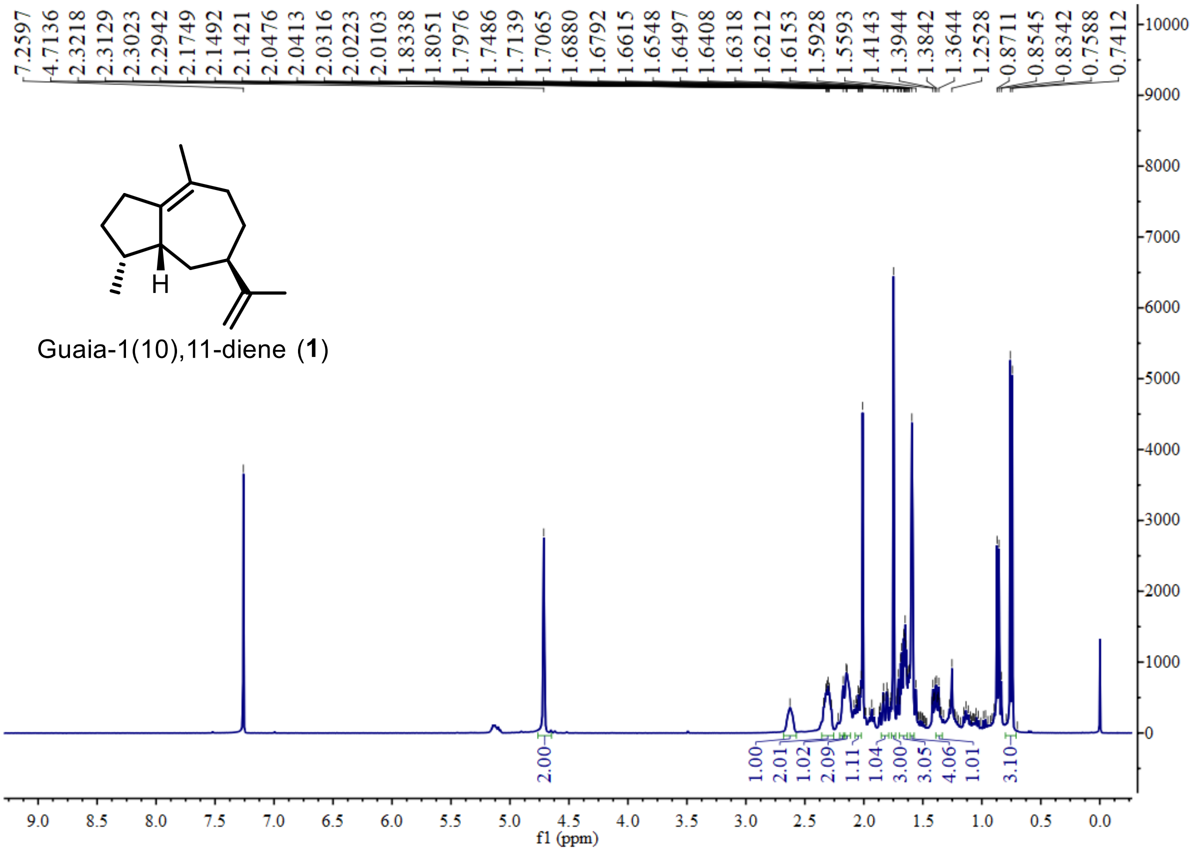

Figure S6 ^{1}H NMR spectrum of **1** in $CDCl_3$ (400 MHz).

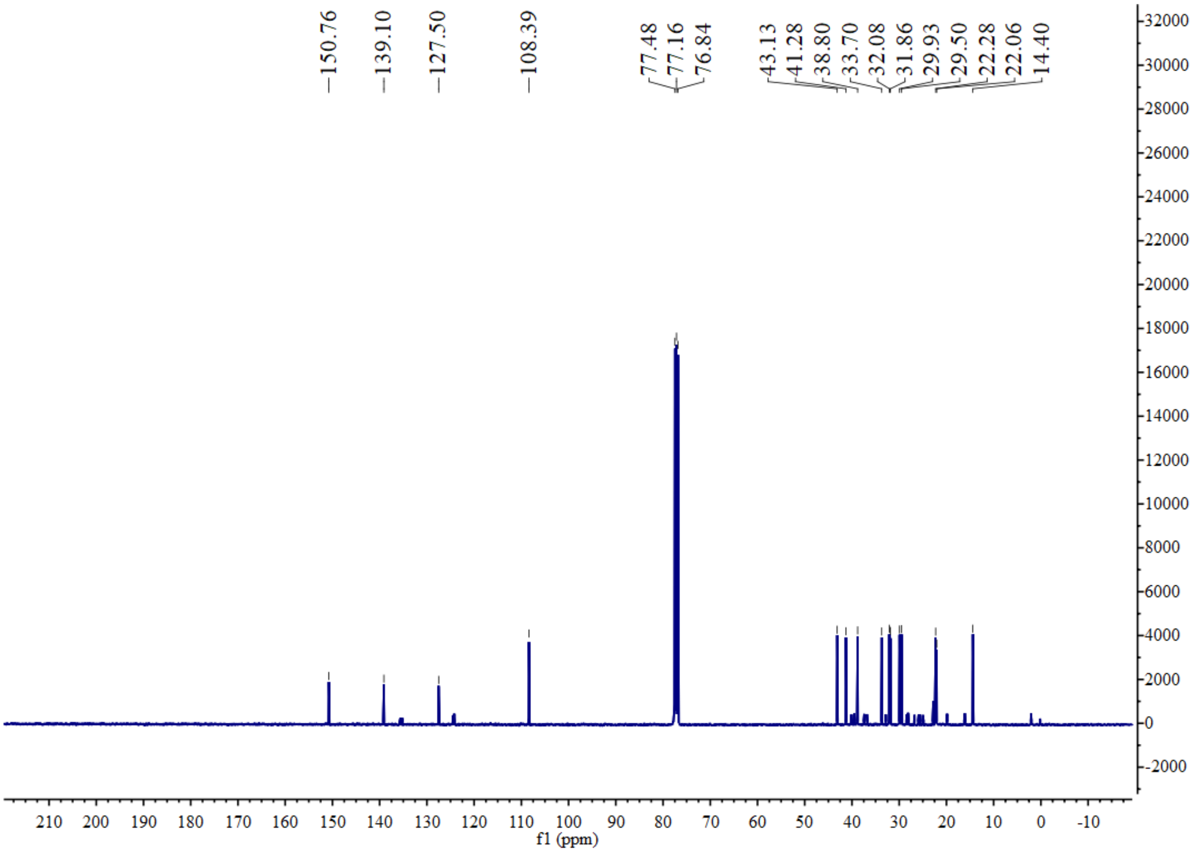

Figure S7 ^{13}C NMR spectrum of **1** in $CDCl_3$ (100 MHz).

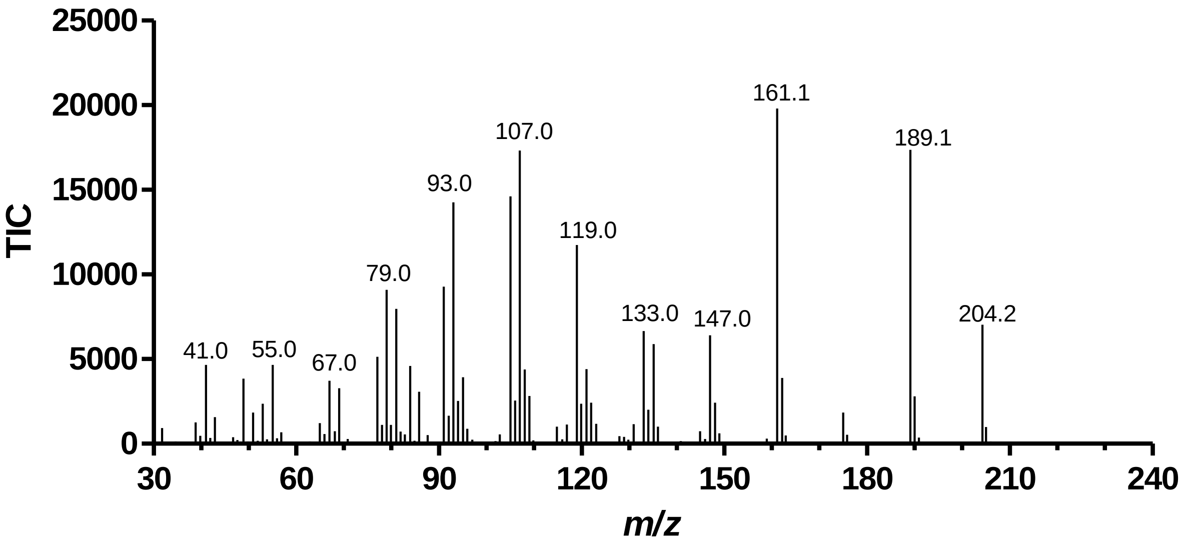

Figure S8 EI mass spectrum of **1**.

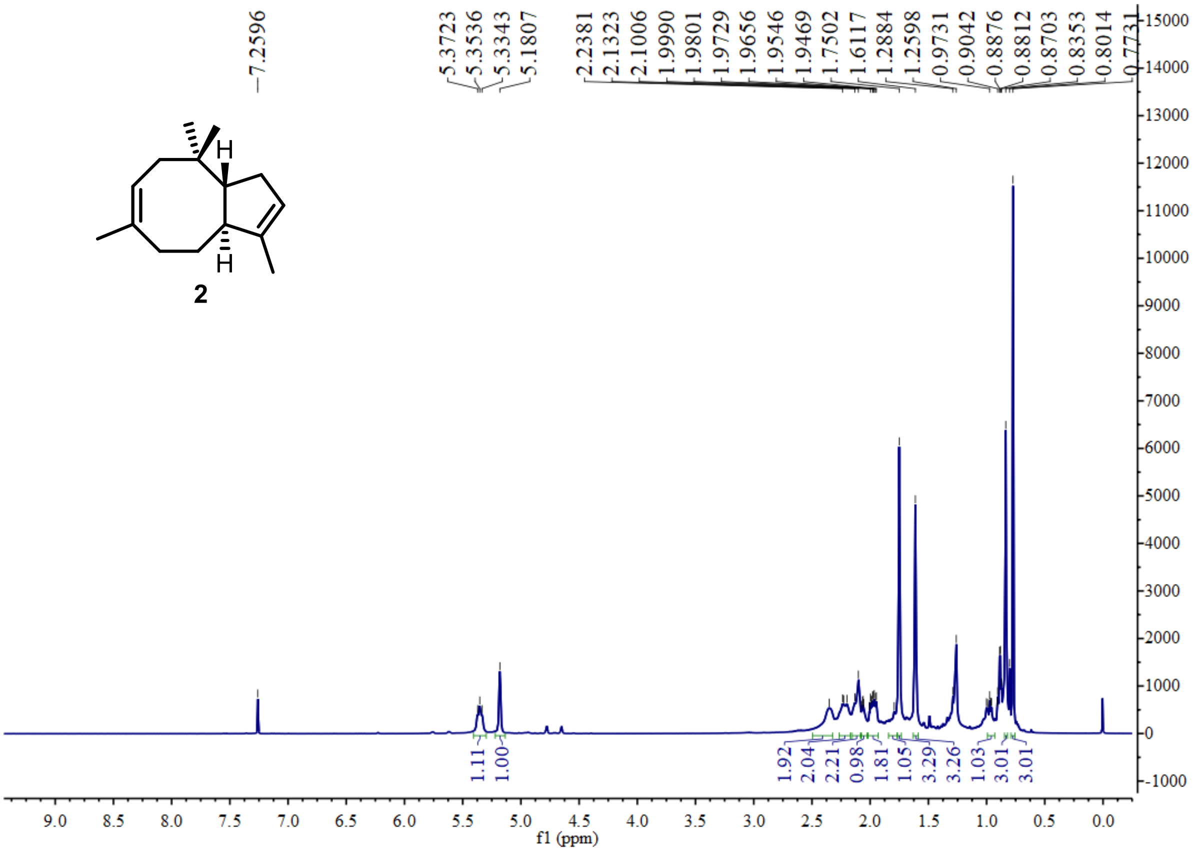

Figure S9 ^{1}H NMR spectrum of **2** in $CDCl_3$ (400 MHz).

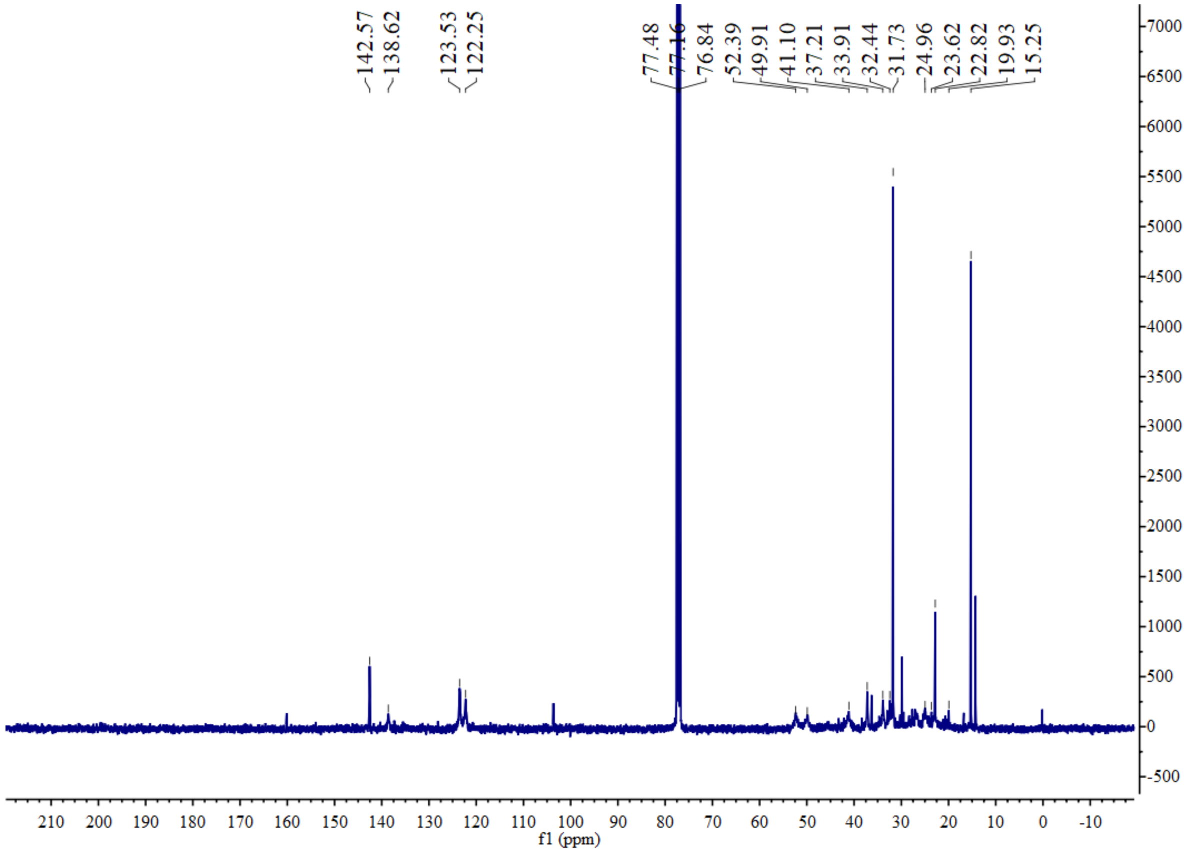

Figure S10 ^{13}C NMR spectrum of **2** in $CDCl_3$ (100 MHz)

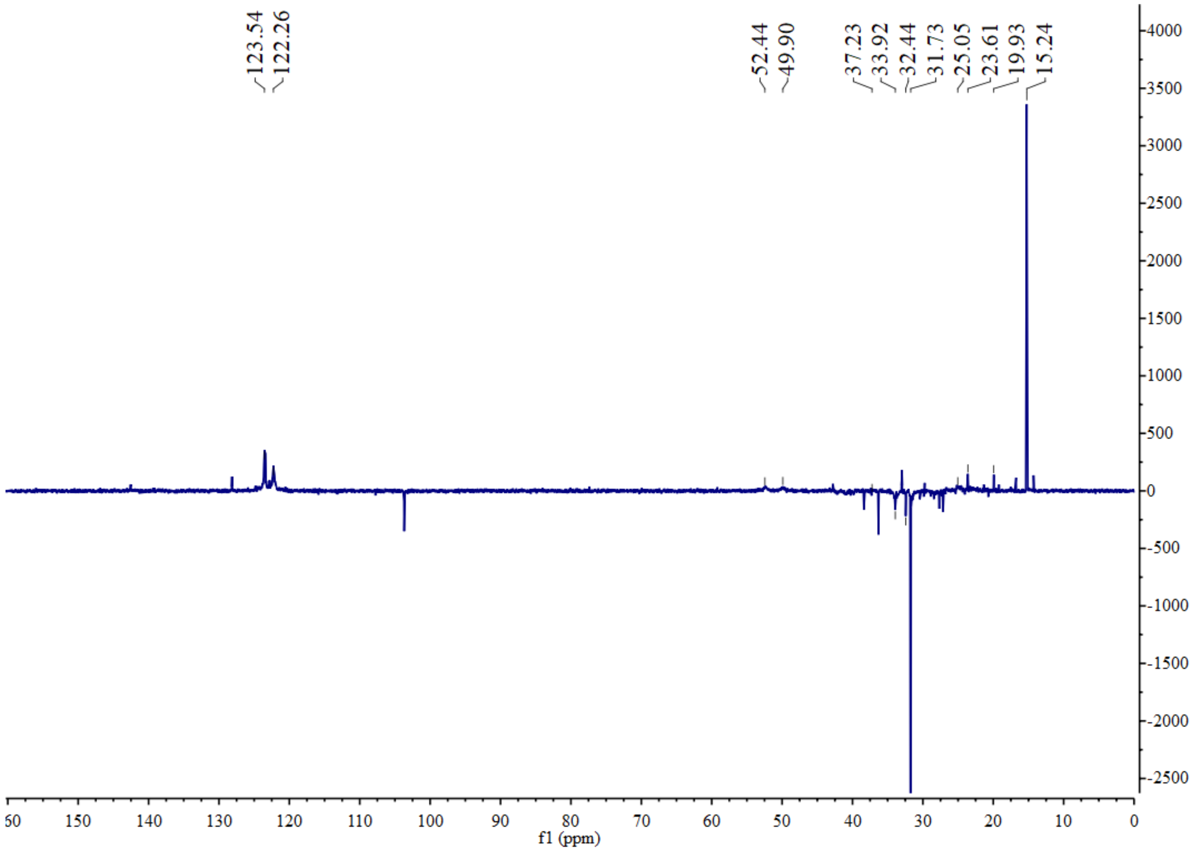

Figure S11 DEPT-135 NMR spectrum of **2** in $CDCl_3$ (100 MHz).

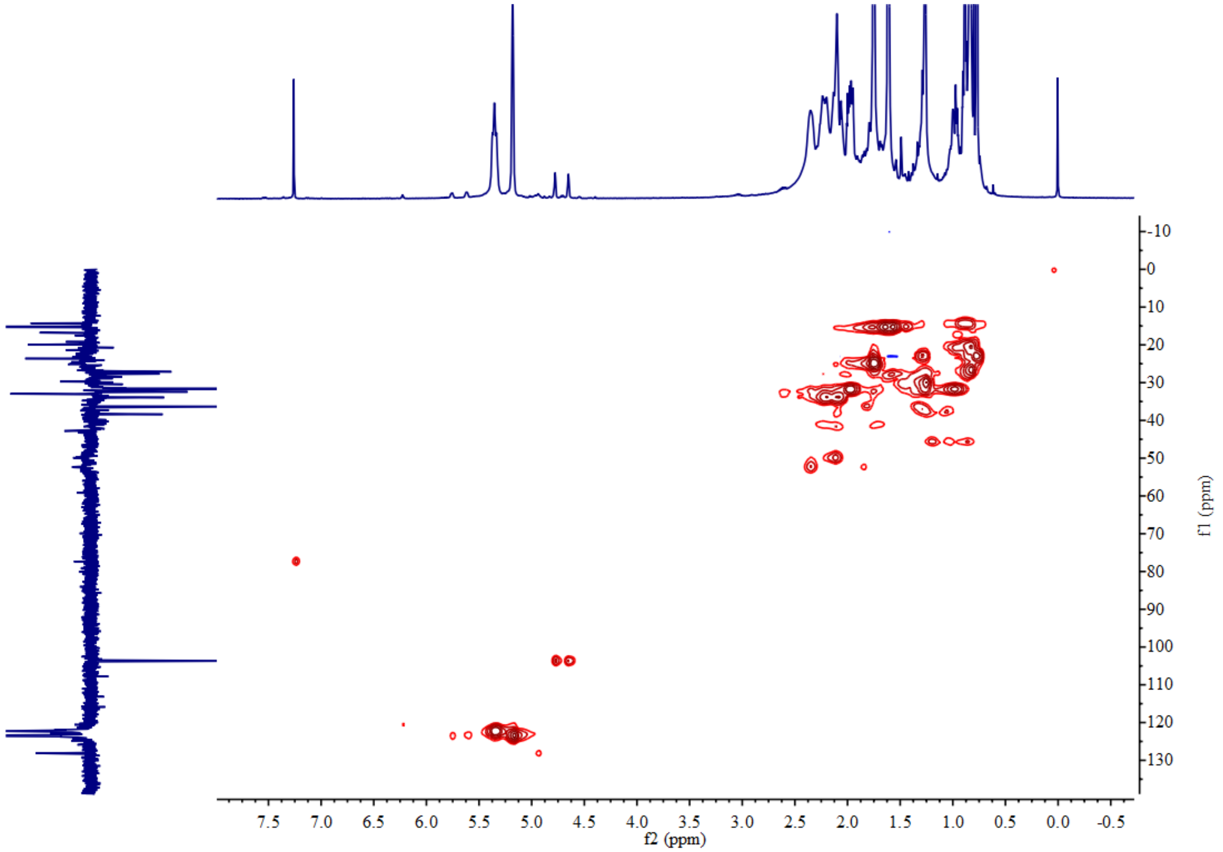

Figure S12 HSQC spectrum of **2** in $CDCl_3$ (400 MHz).

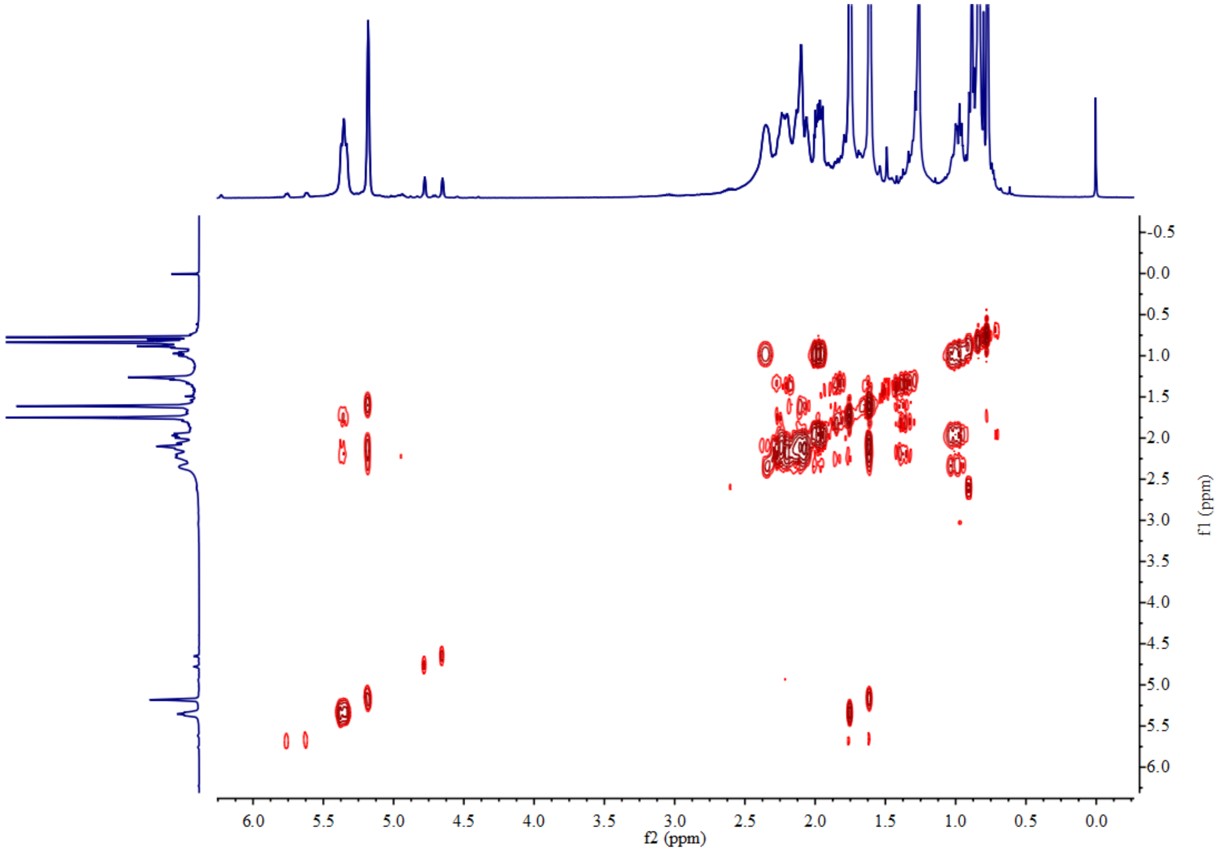

Figure S13 ^1H-^1H COSY spectrum of **2** in $CDCl_3$ (400 MHz).

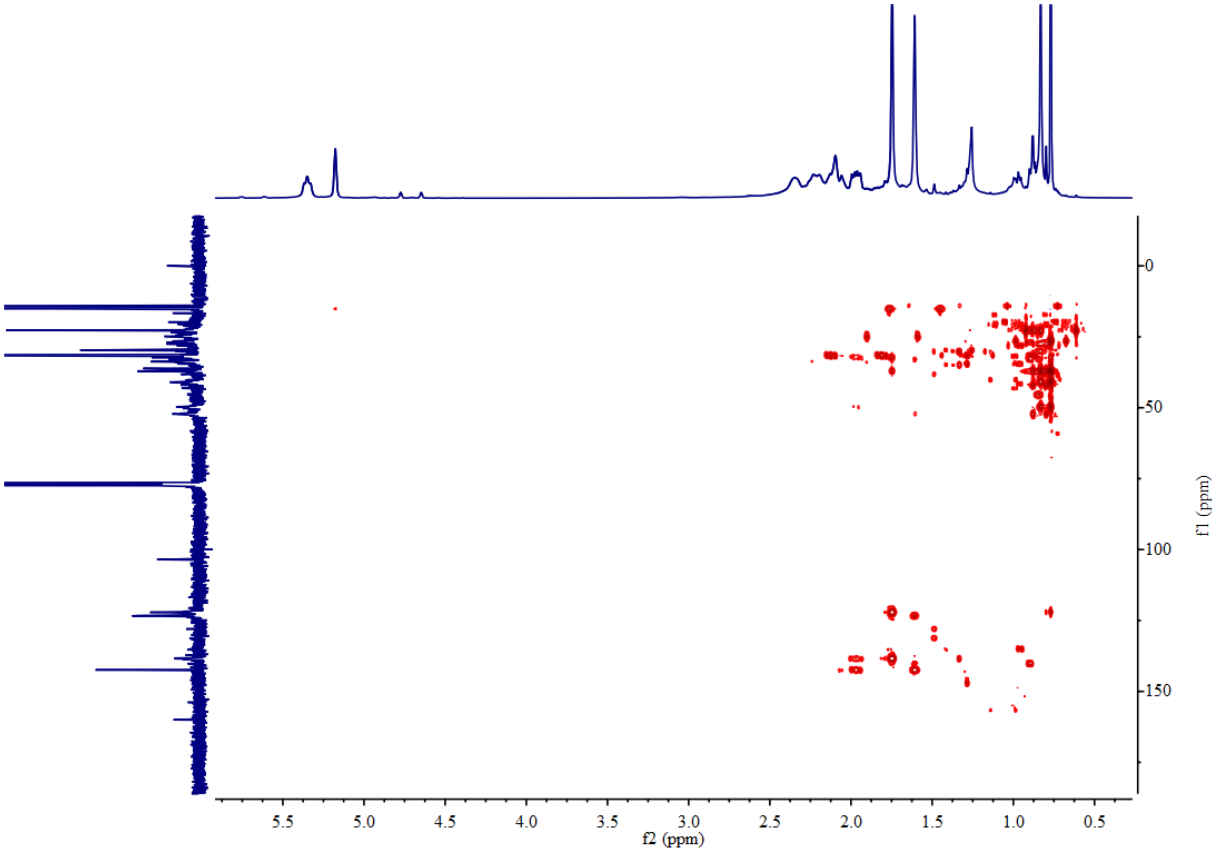

Figure S14 HMBC spectrum of **2** in $CDCl_3$ (400 MHz).

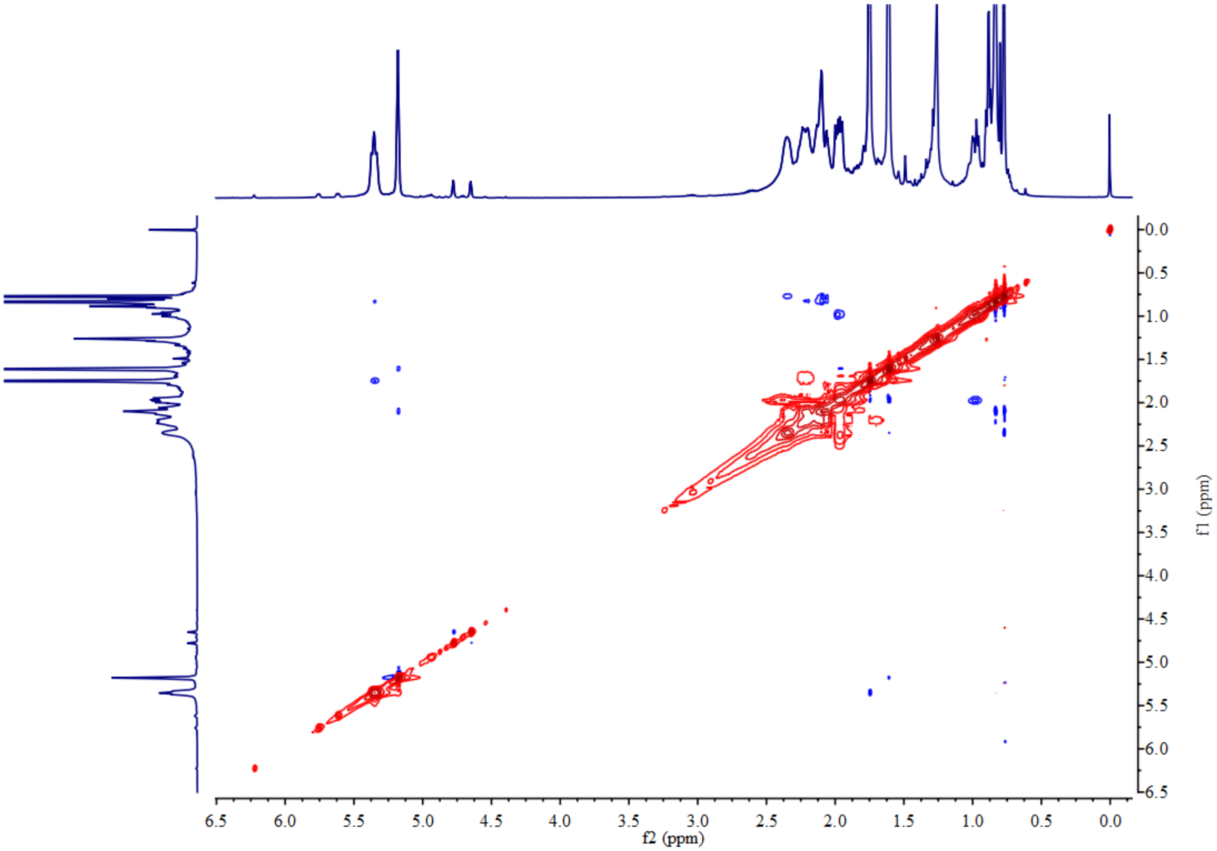

Figure S15 NOESY spectrum of **2** in $CDCl_3$ (400 MHz).

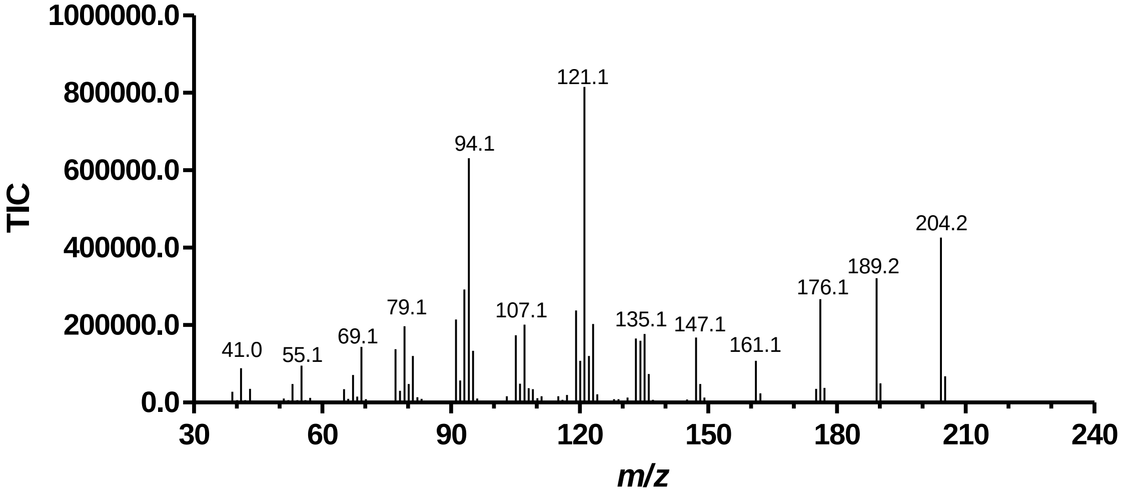

Figure S16 EI mass spectrum of **2**.

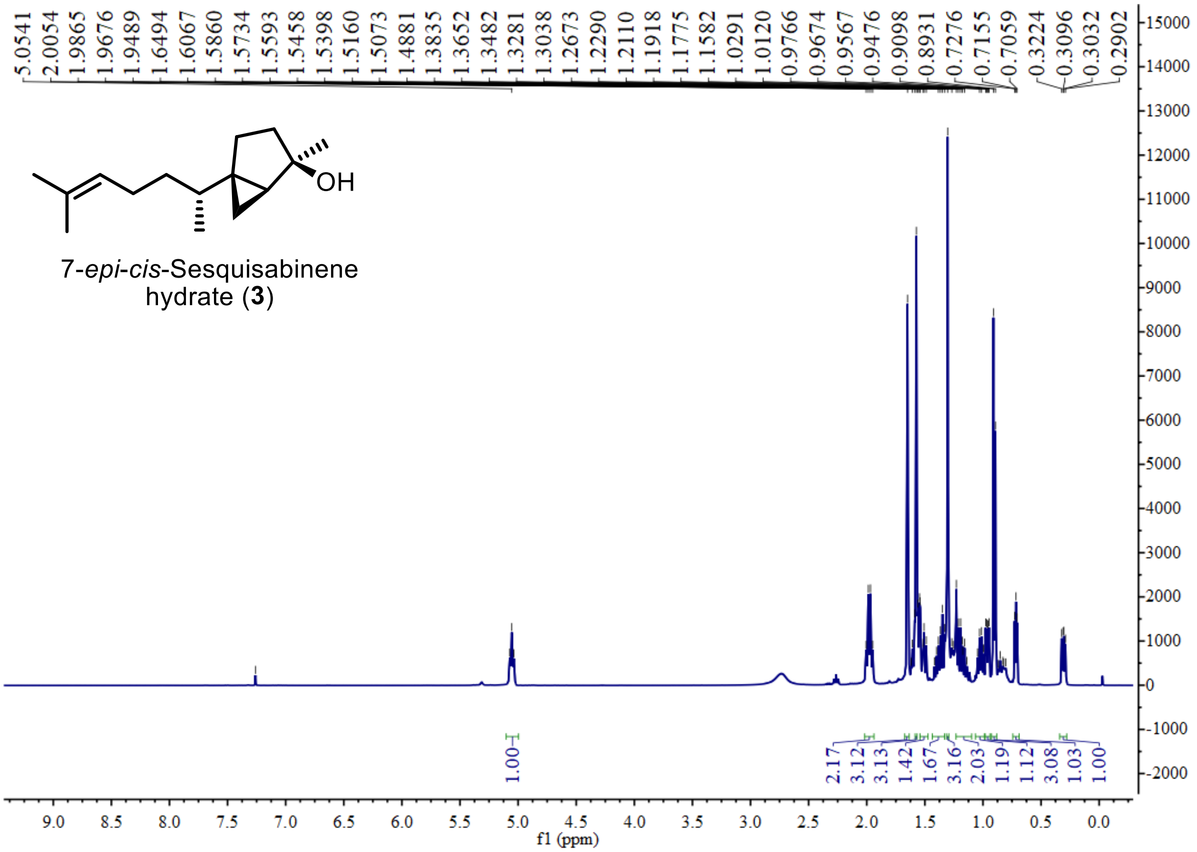

Figure S17 ^{1}H NMR spectrum of **3** in $CDCl_3$ (400 MHz).

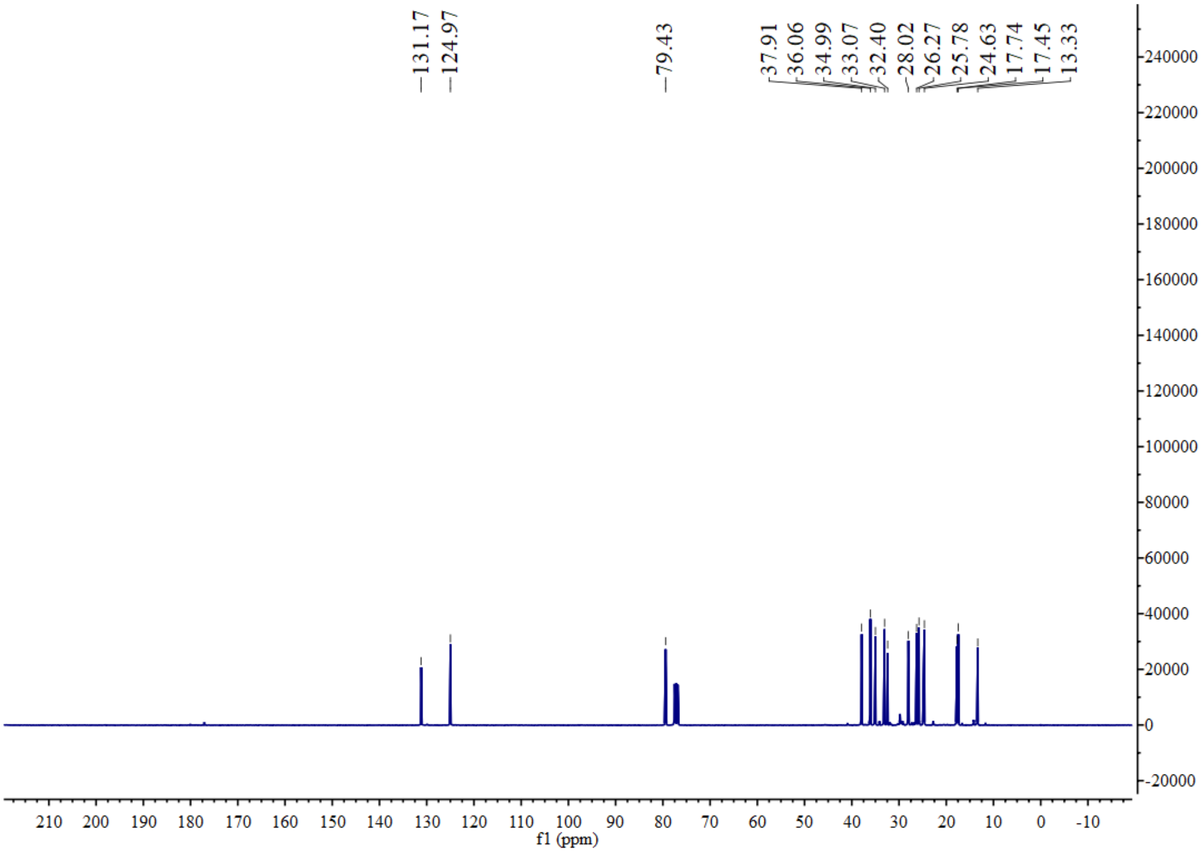

Figure S18 ^{13}C NMR spectrum of **3** in $CDCl_3$ (100 MHz).

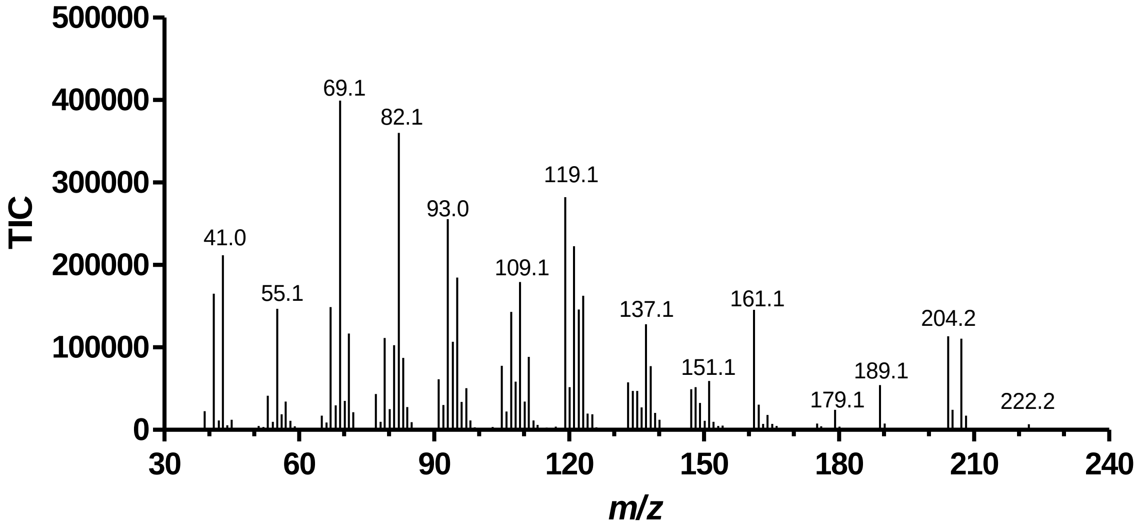

Figure S19 EI mass spectrum of **3**.

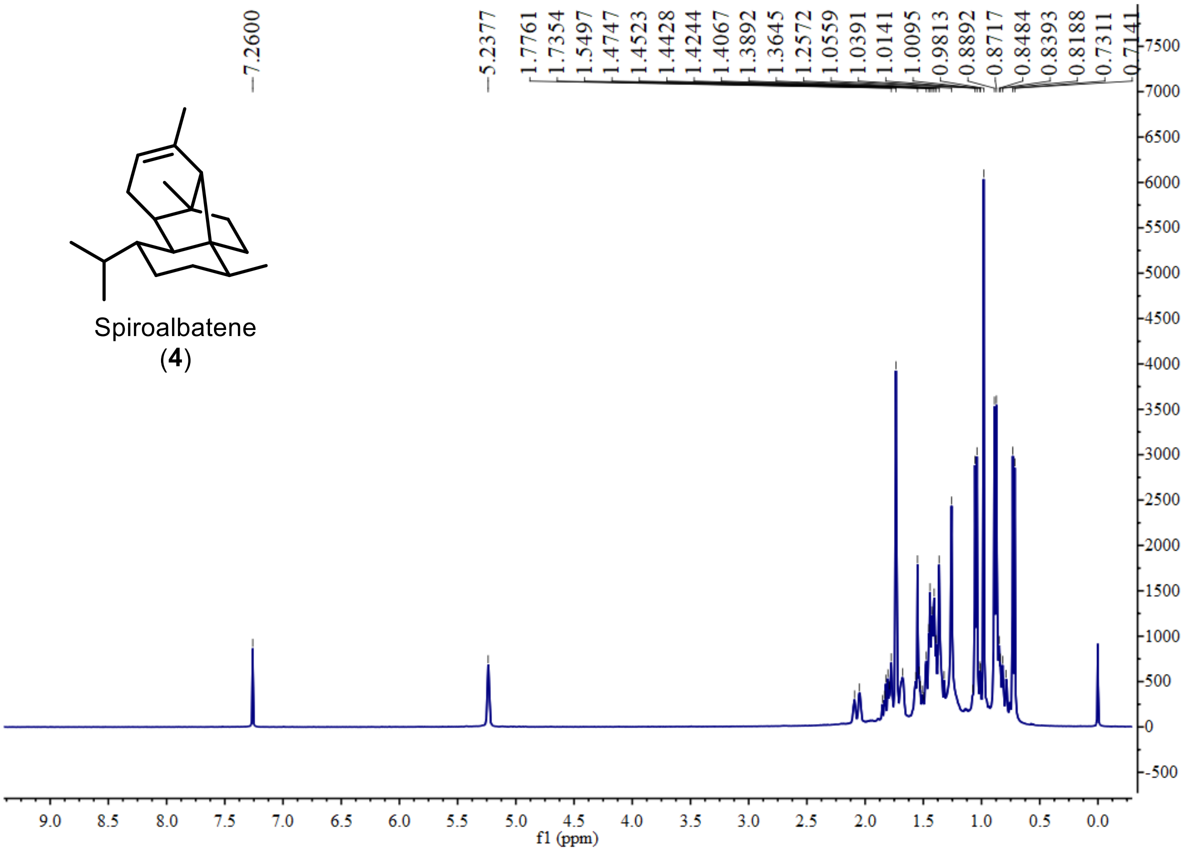

Figure S20 ^{1}H NMR spectrum of **4** in $CDCl_3$ (400 MHz).

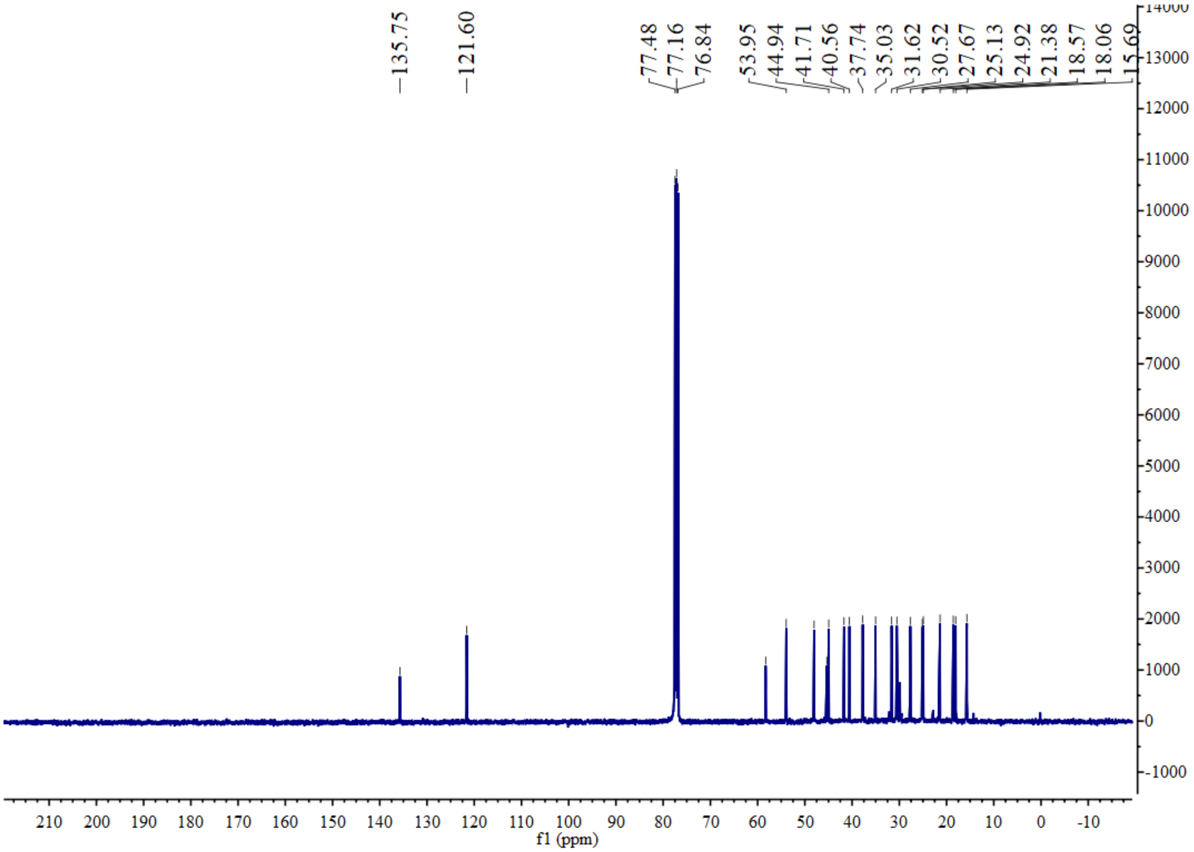

Figure S21 ^{13}C NMR spectrum of **4** in $CDCl_3$ (100 MHz).

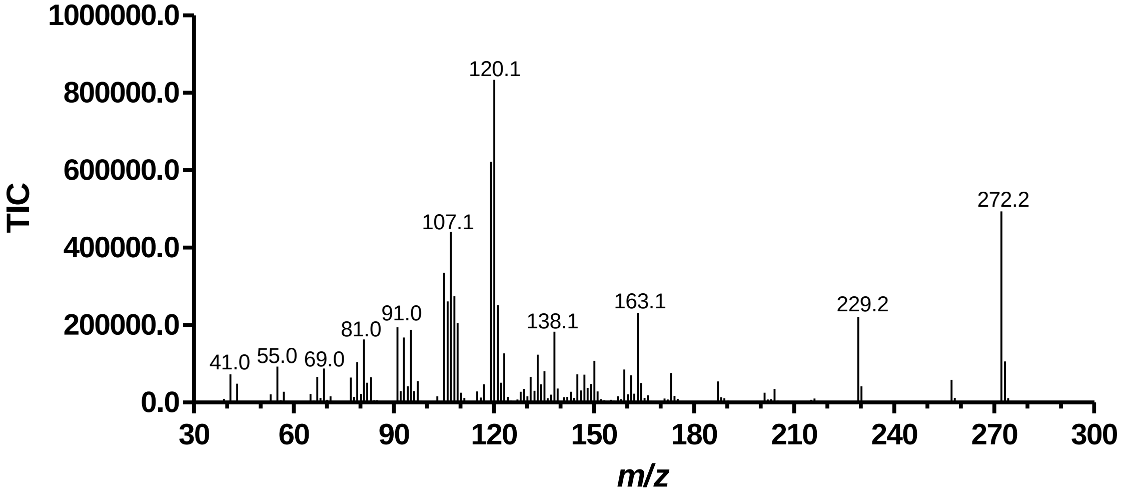

Figure S22 EI mass spectrum of **4**.

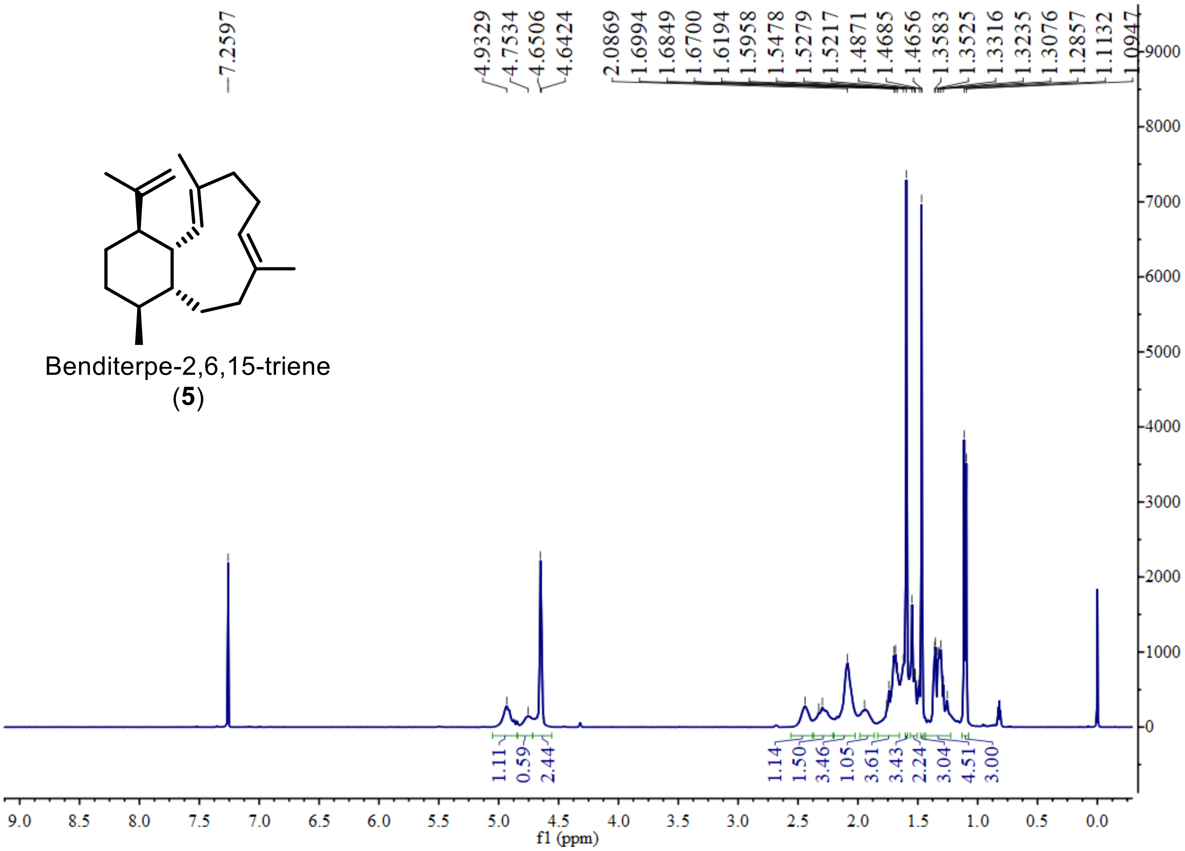

Figure S23 ^{1}H NMR spectrum of **5** in $CDCl_3$ (400 MHz).

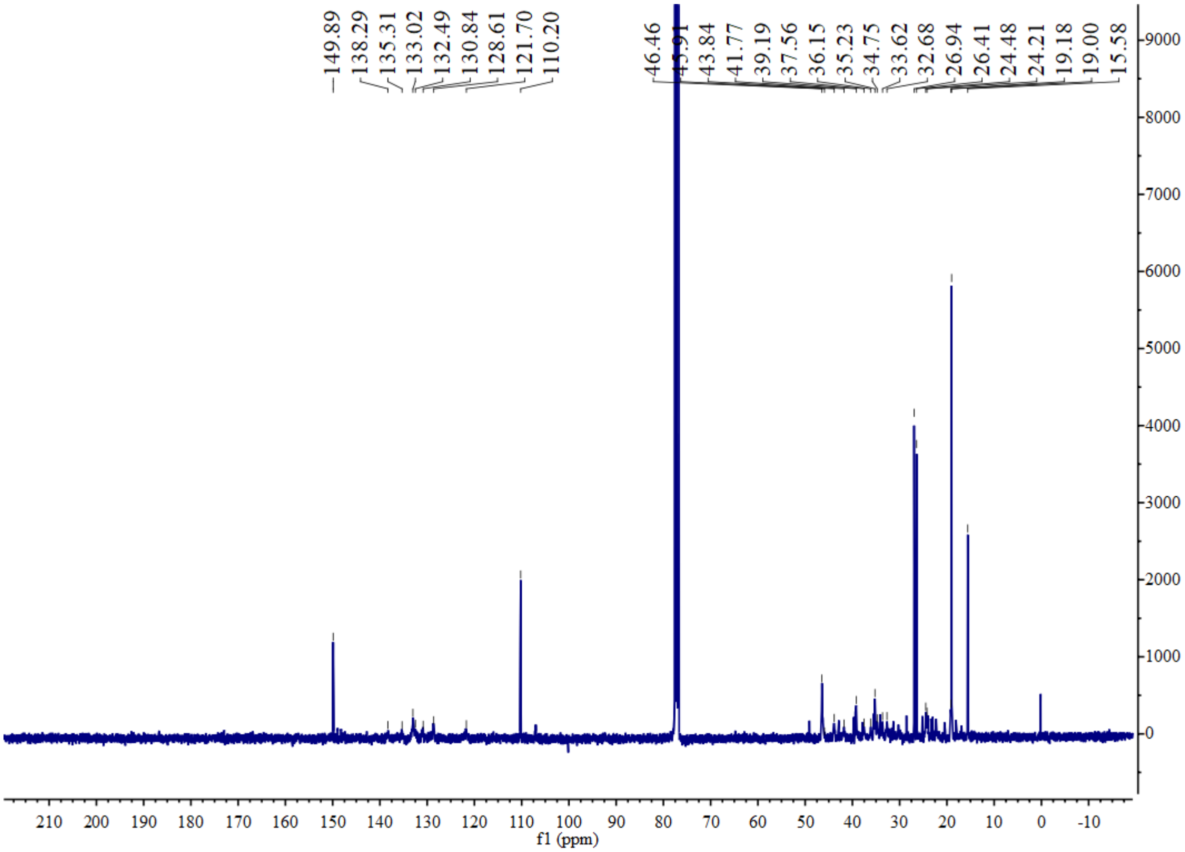

Figure S24 ^{13}C NMR spectrum of **5** in $CDCl_3$ (100 MHz).

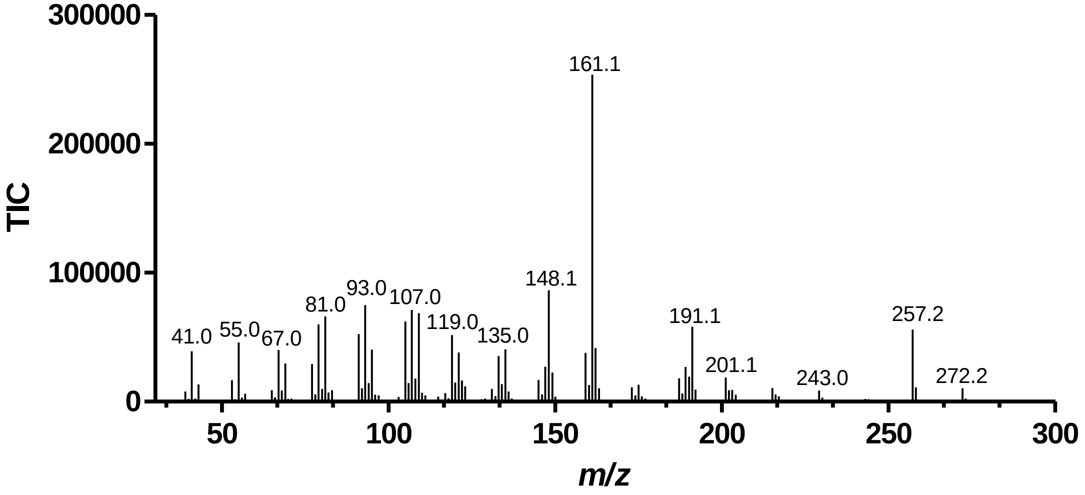

Figure S25 EI mass spectrum of **5**.

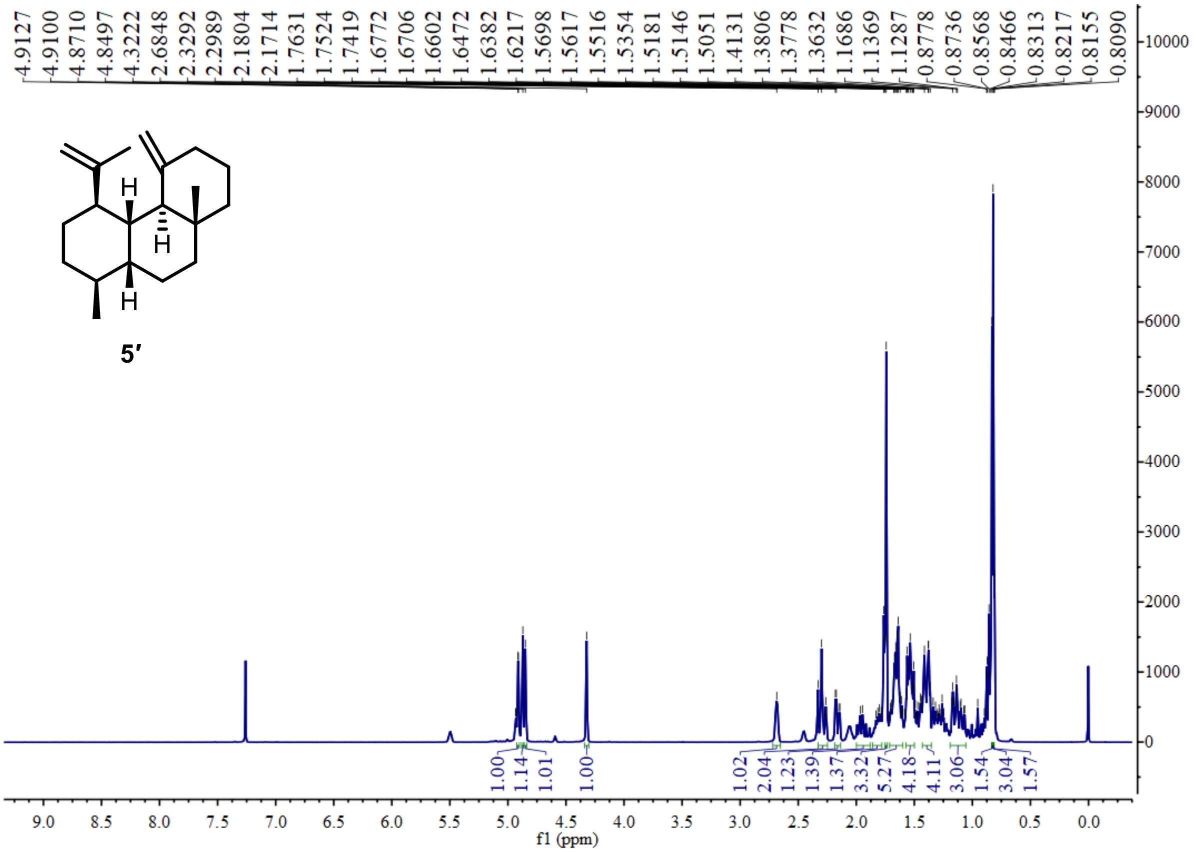

Figure S26 ^{1}H NMR spectrum of **5′** in $CDCl_3$ (400 MHz).

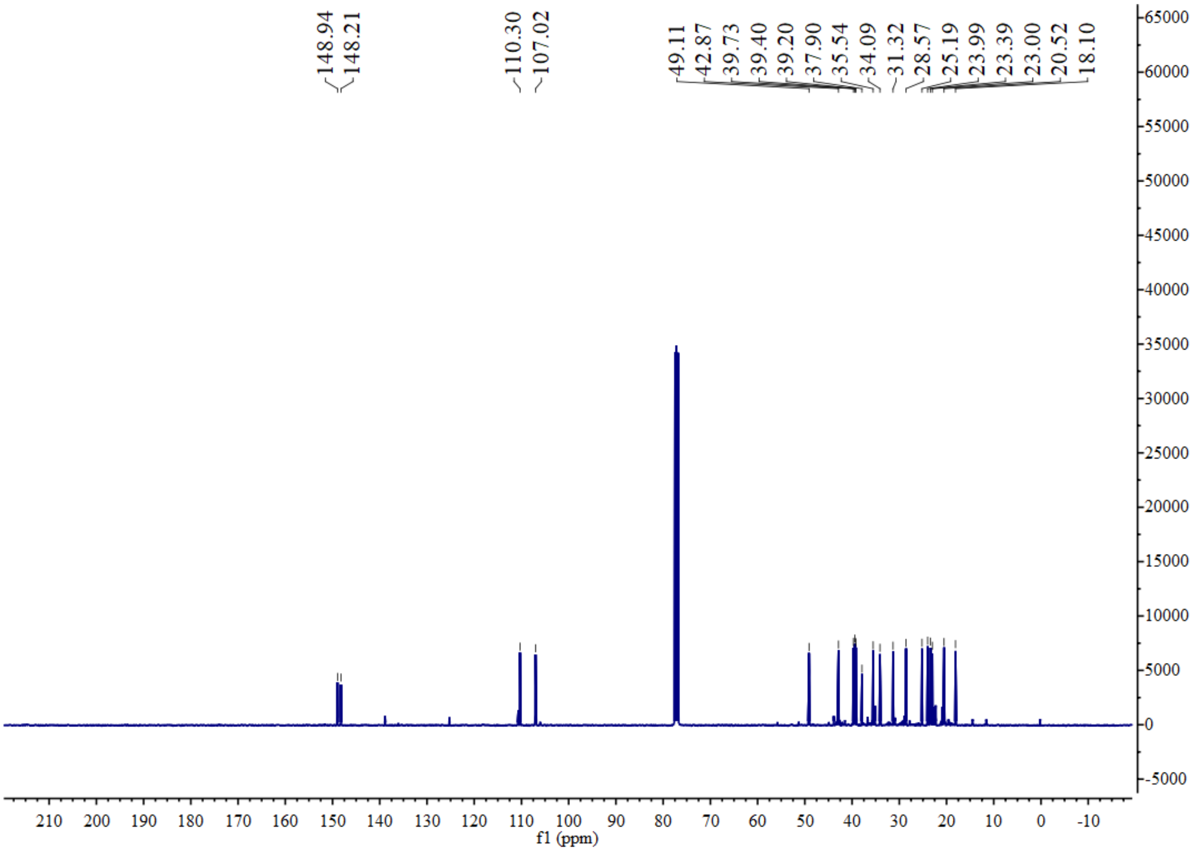

Figure S27 ^{13}C NMR spectrum of **5′** in $CDCl_3$ (100 MHz).

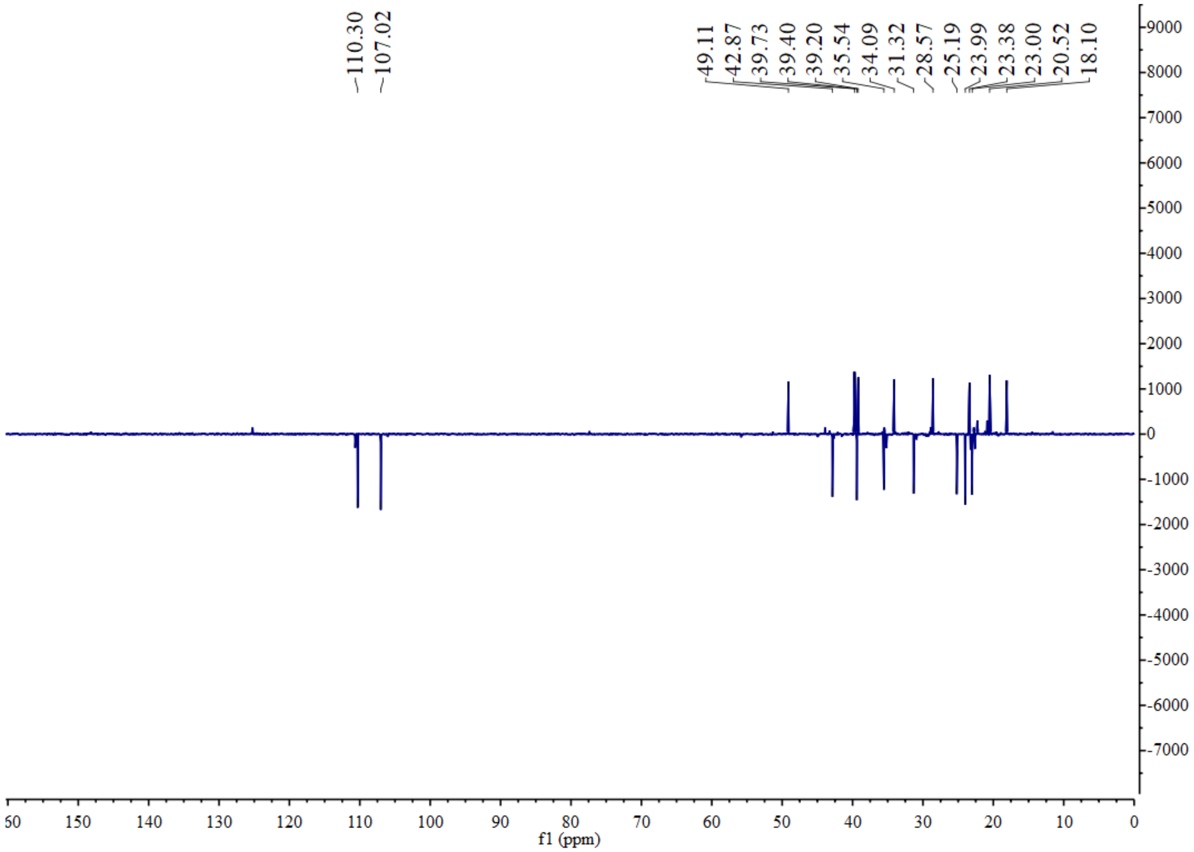

Figure S28 DEPT-135 NMR spectrum of **5′** in $CDCl_3$ (100 MHz).

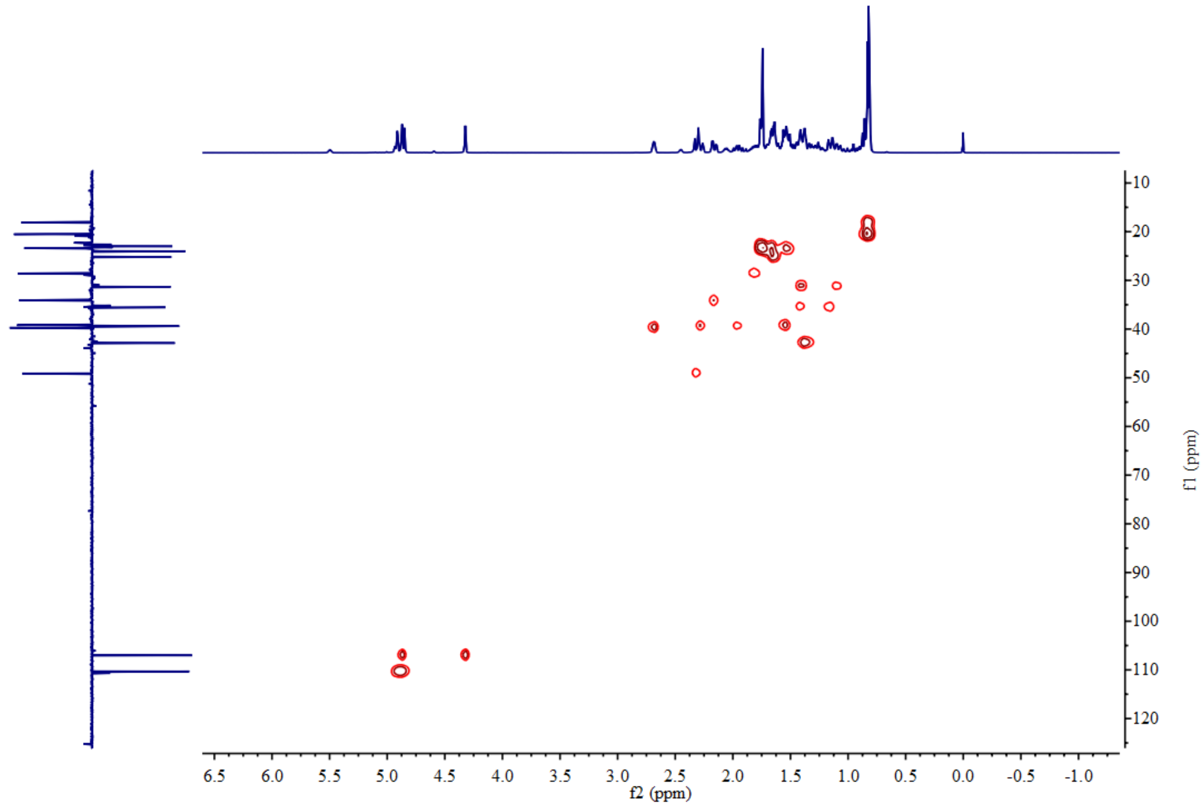

Figure S29 HSQC spectrum of **5′** in $CDCl_3$ (400 MHz).

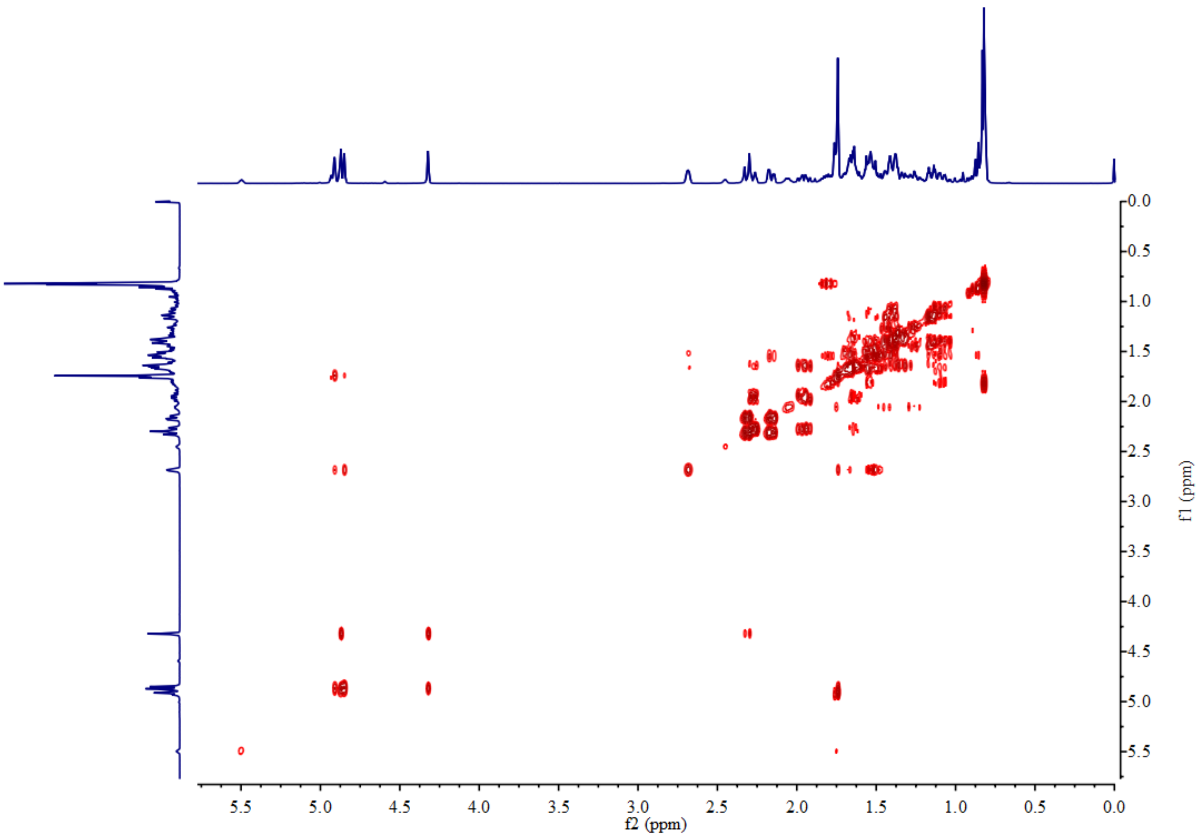

Figure S30 ^{1}H-^{1}H COSY spectrum of **5′** in $CDCl_3$ (400 MHz).

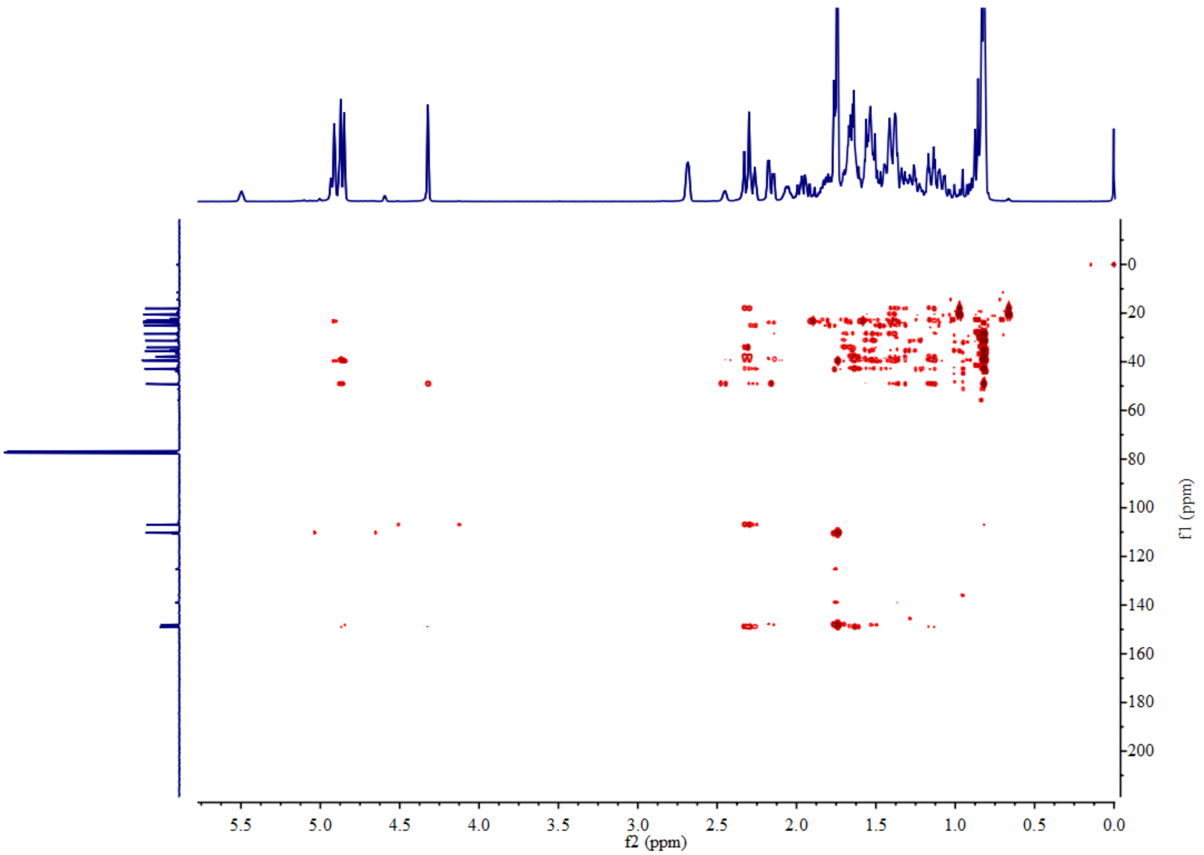

Figure S31 HMBC spectrum of **5′** in $CDCl_3$ (400 MHz).

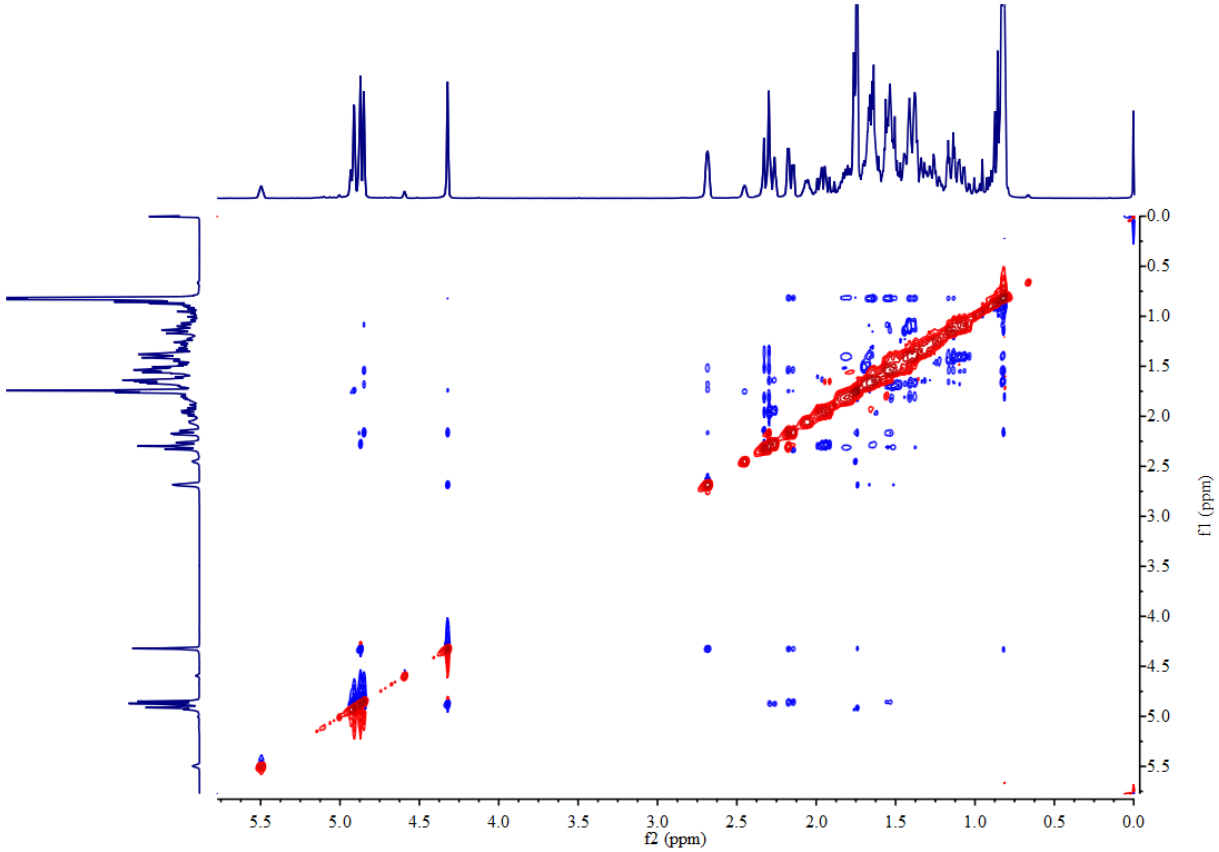

Figure S32 NOESY spectrum of **5′** in $CDCl_3$ (400 MHz).

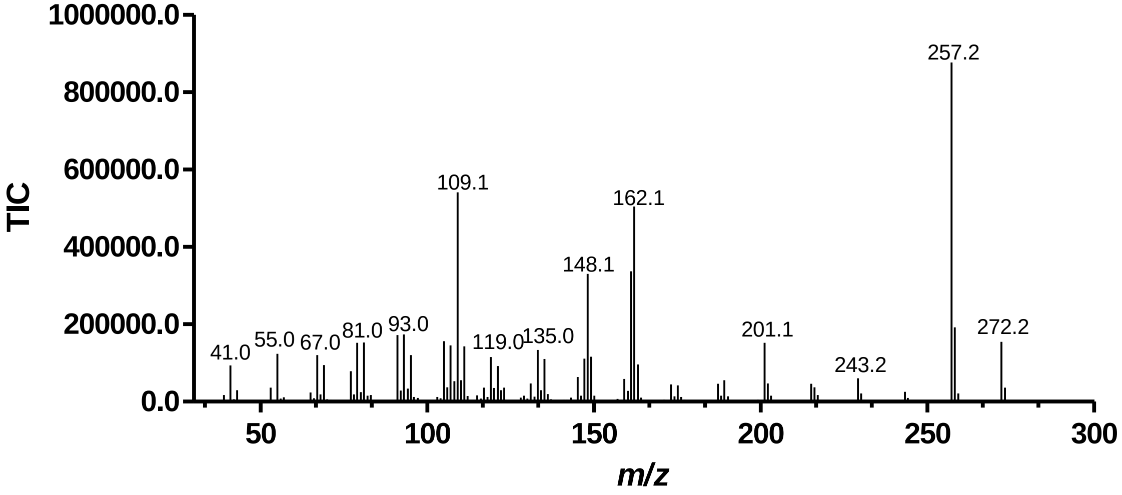

Figure S33 EI mass spectrum of **5′**.

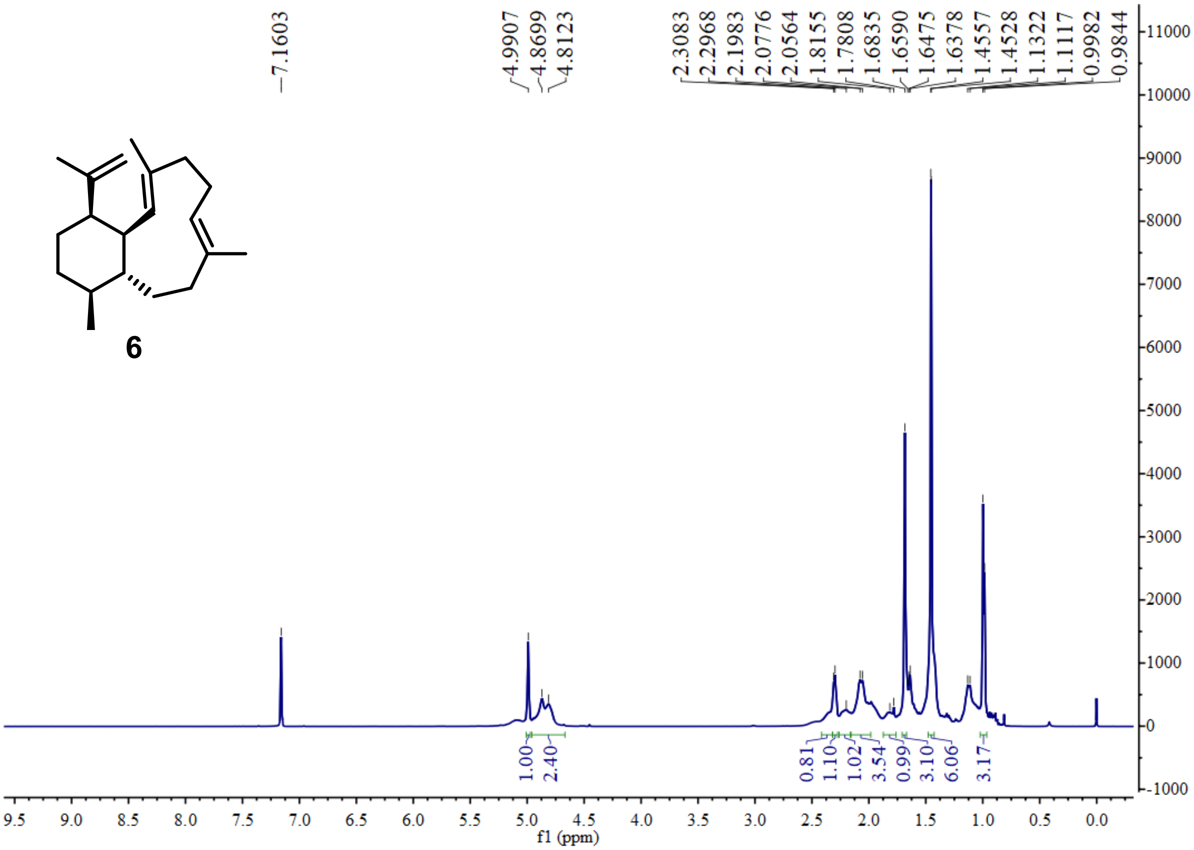

Figure S34 ^{1}H NMR spectrum of **6** in C_6D_6 (400 MHz).

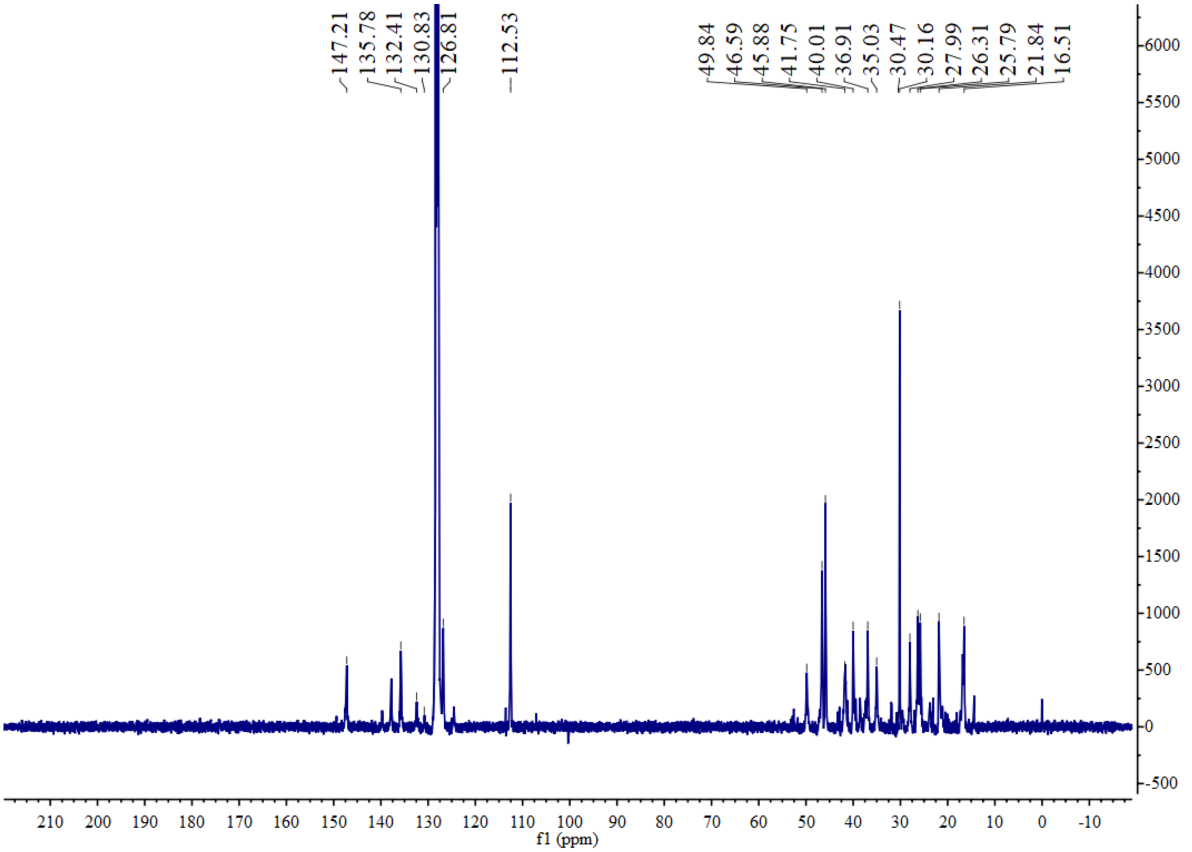

Figure S35 ^{13}C NMR spectrum of **6** in C_6D_6 (100 MHz).

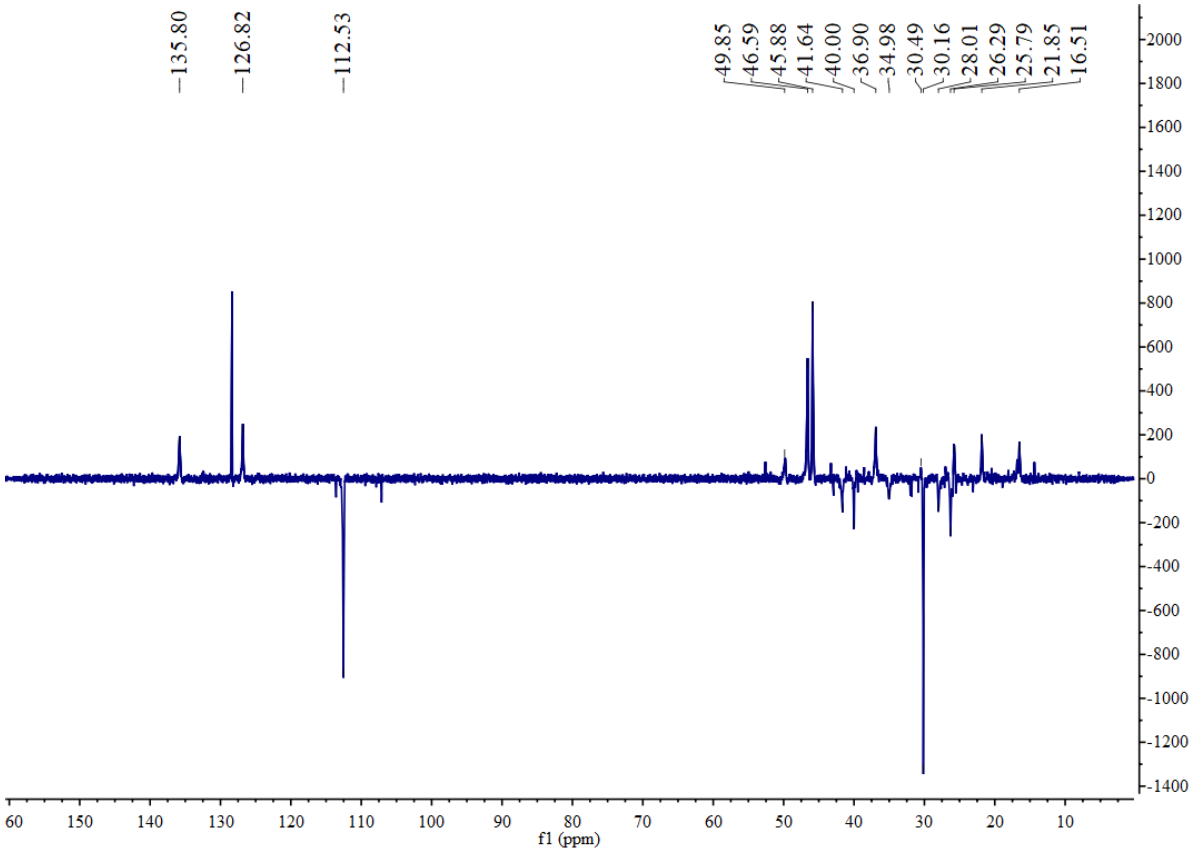

Figure S36 DEPT-135 NMR spectrum of **6** in C_6D_6 (100 MHz).

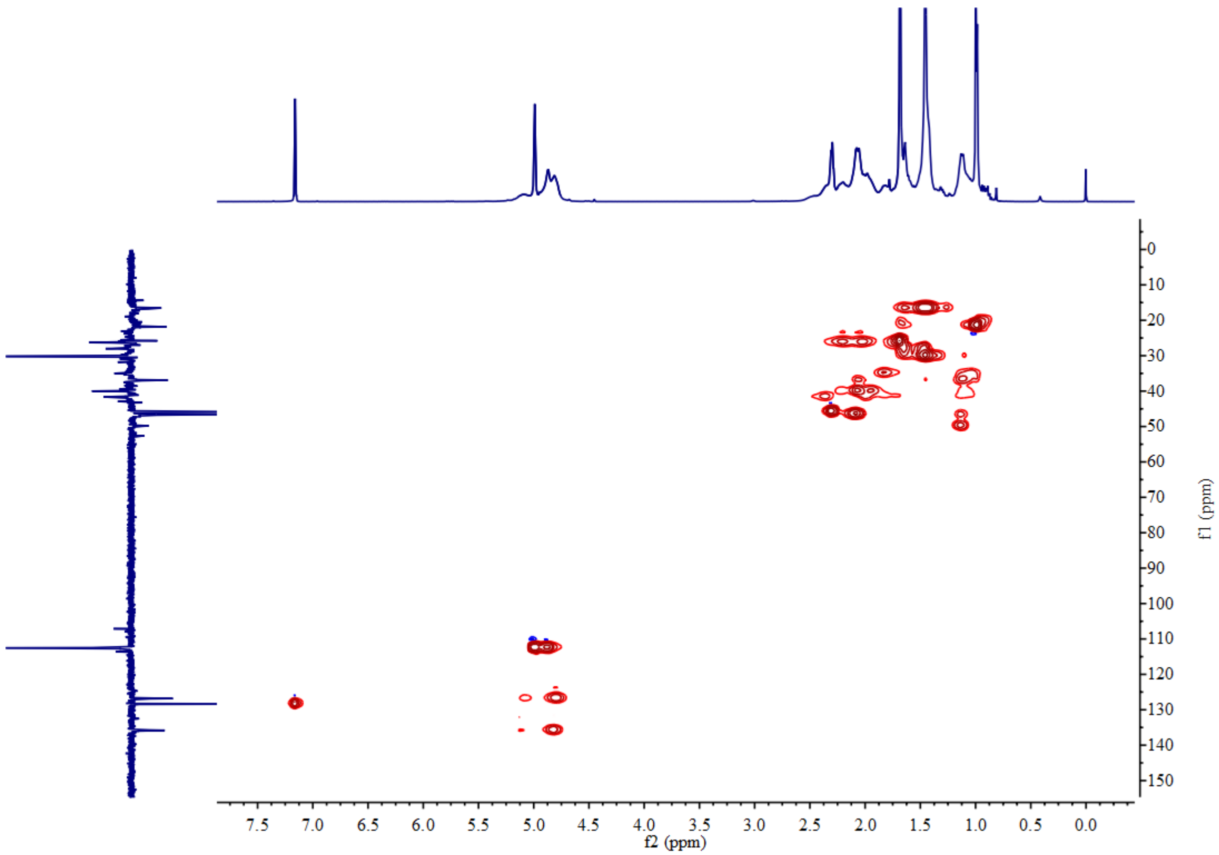

Figure S37 HSQC spectrum of **6** in C_6D_6 (400 MHz).

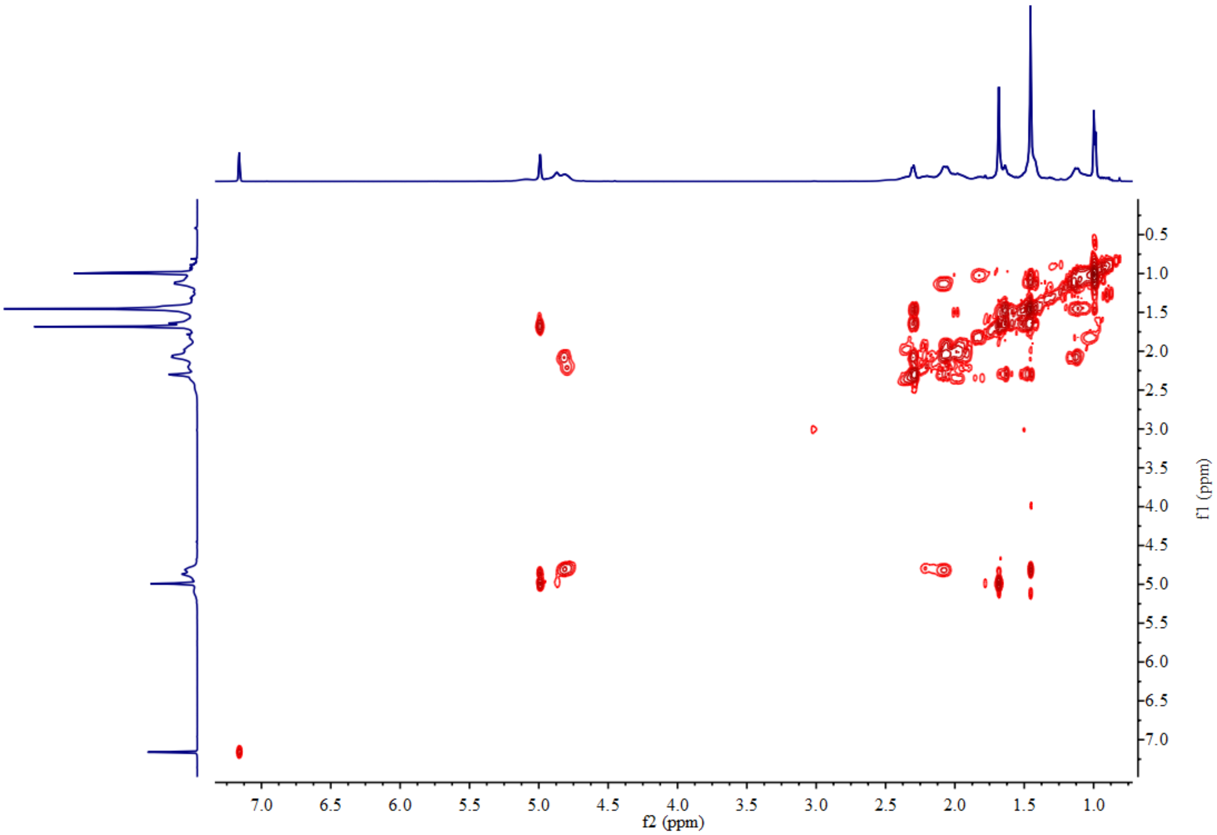

Figure S38 ^1H-^1H COSY spectrum of **6** in C_6D_6 (400 MHz).

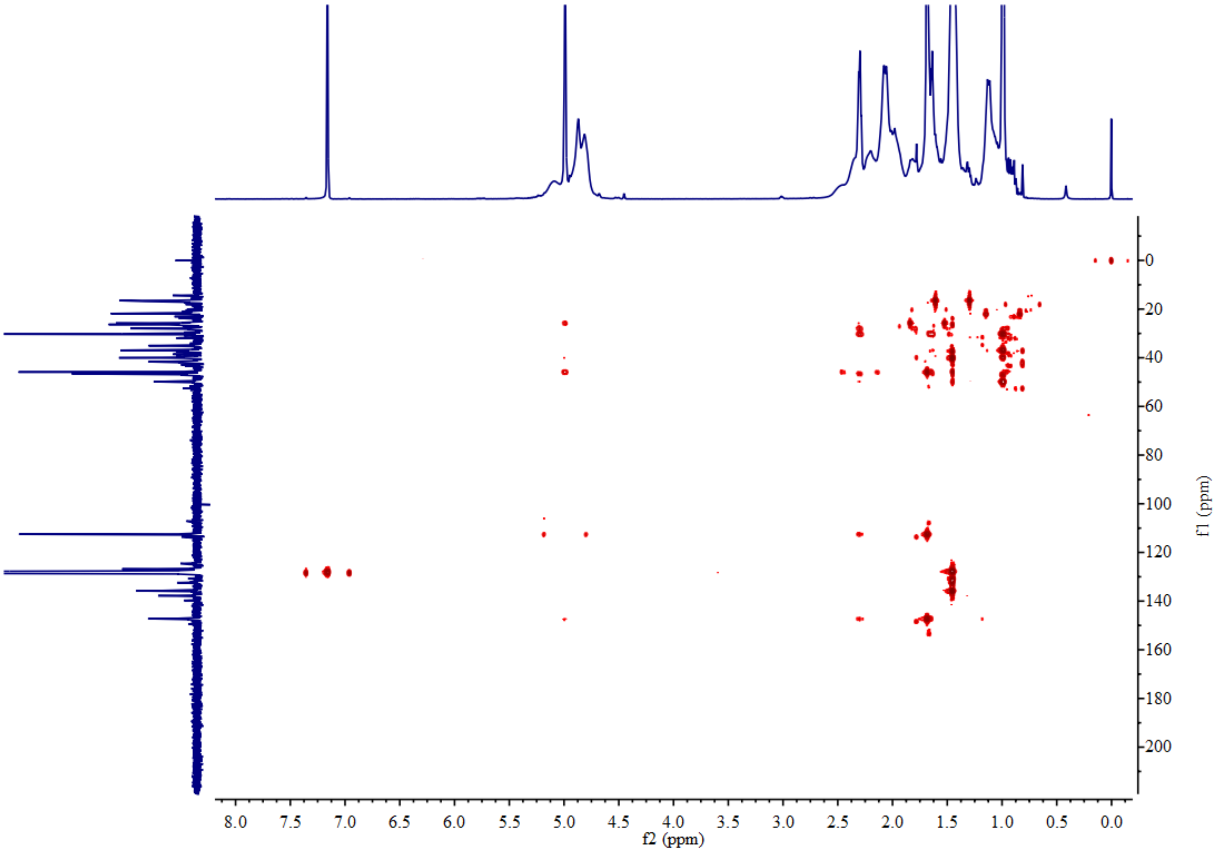

Figure S39 HMBC spectrum of **6** in C_6D_6 (400 MHz).

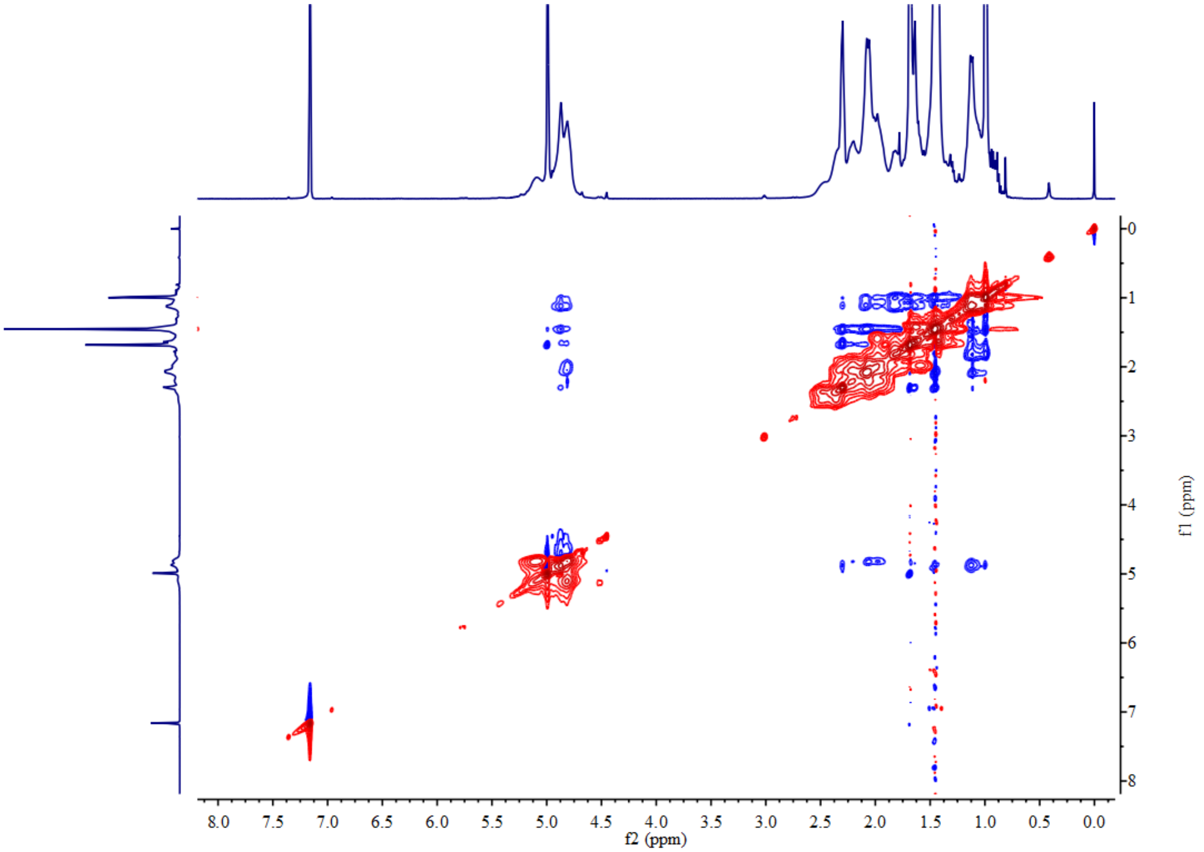

Figure S40 NOESY spectrum of **6** in C_6D_6 (400 MHz).

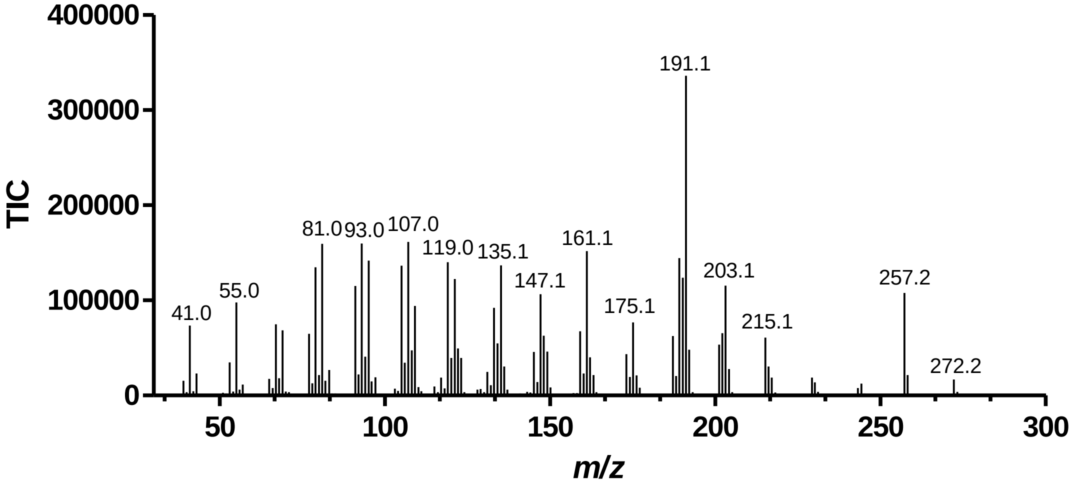

Figure S41 EI mass spectrum of **6**.

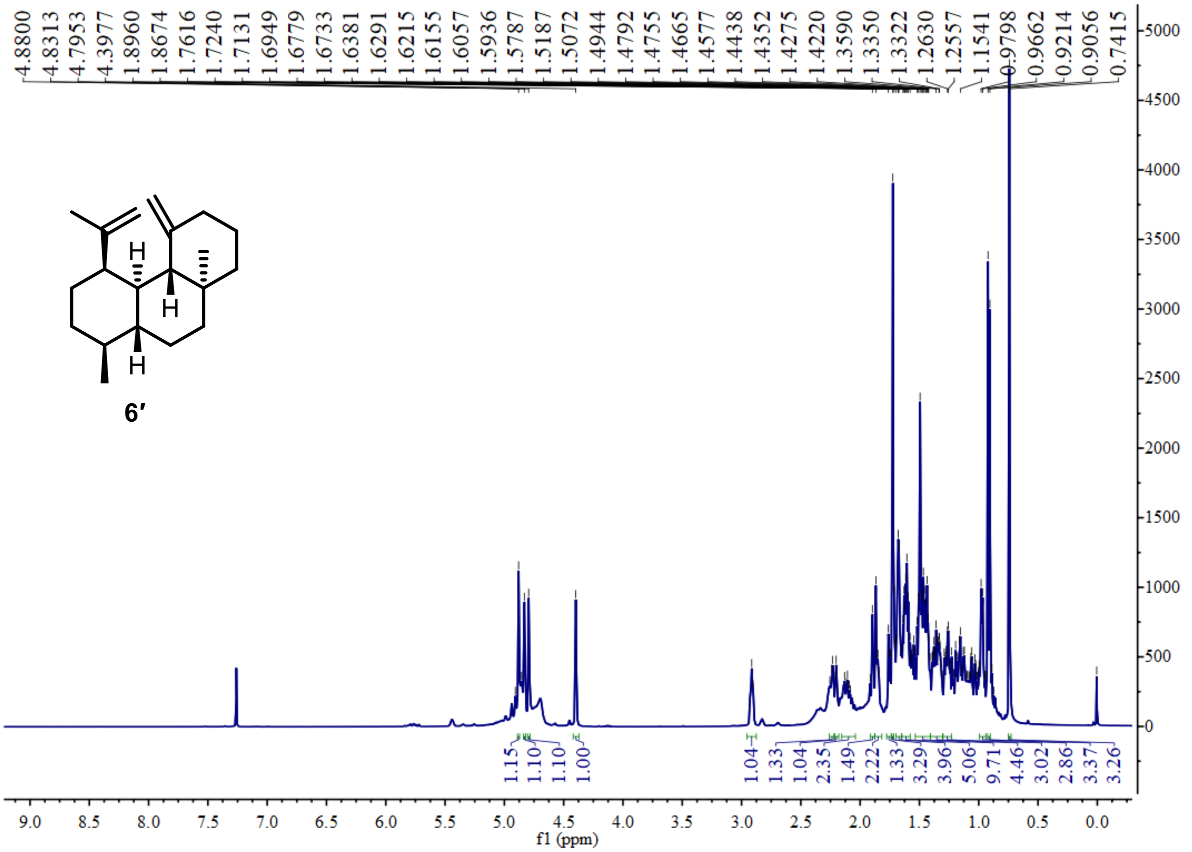

Figure S42 ^{1}H NMR spectrum of **6′** in $CDCl_3$ (400 MHz).

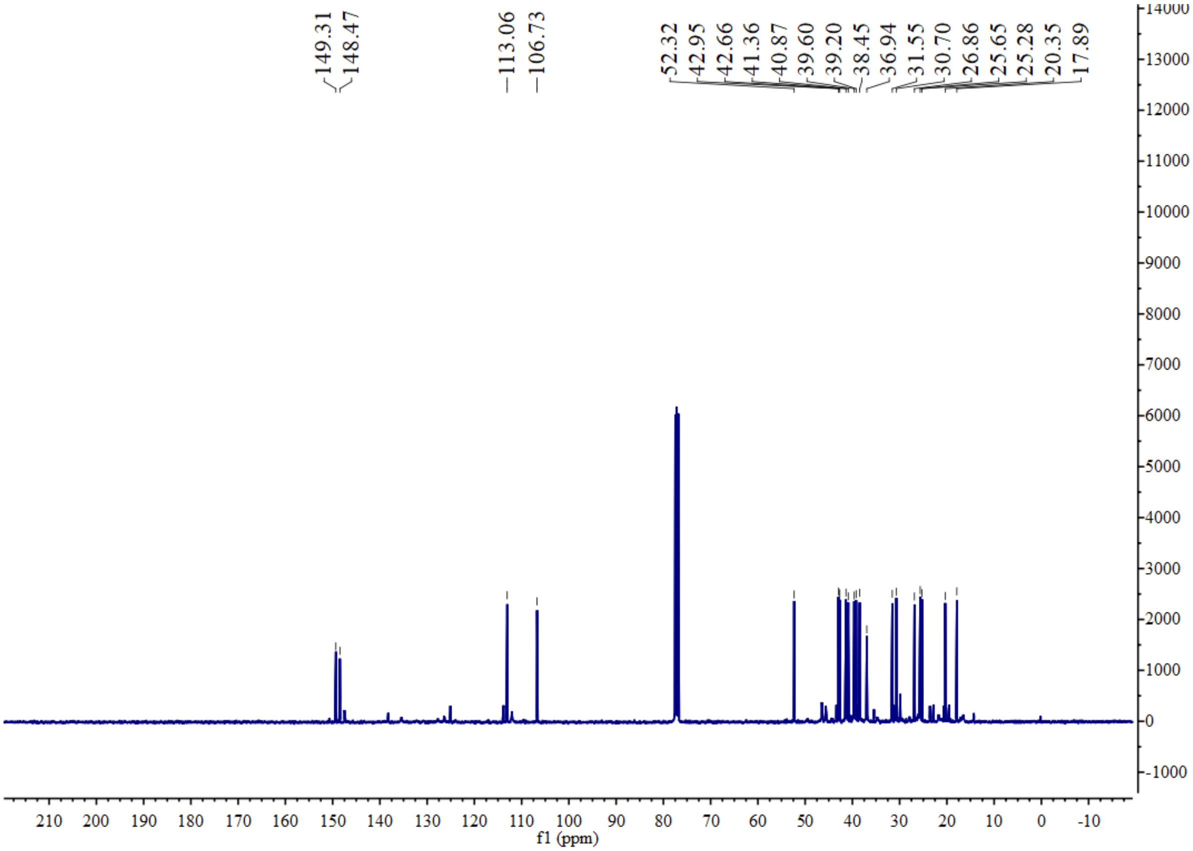

Figure S43 ^{13}C NMR spectrum of **6′** in $CDCl_3$ (100 MHz).

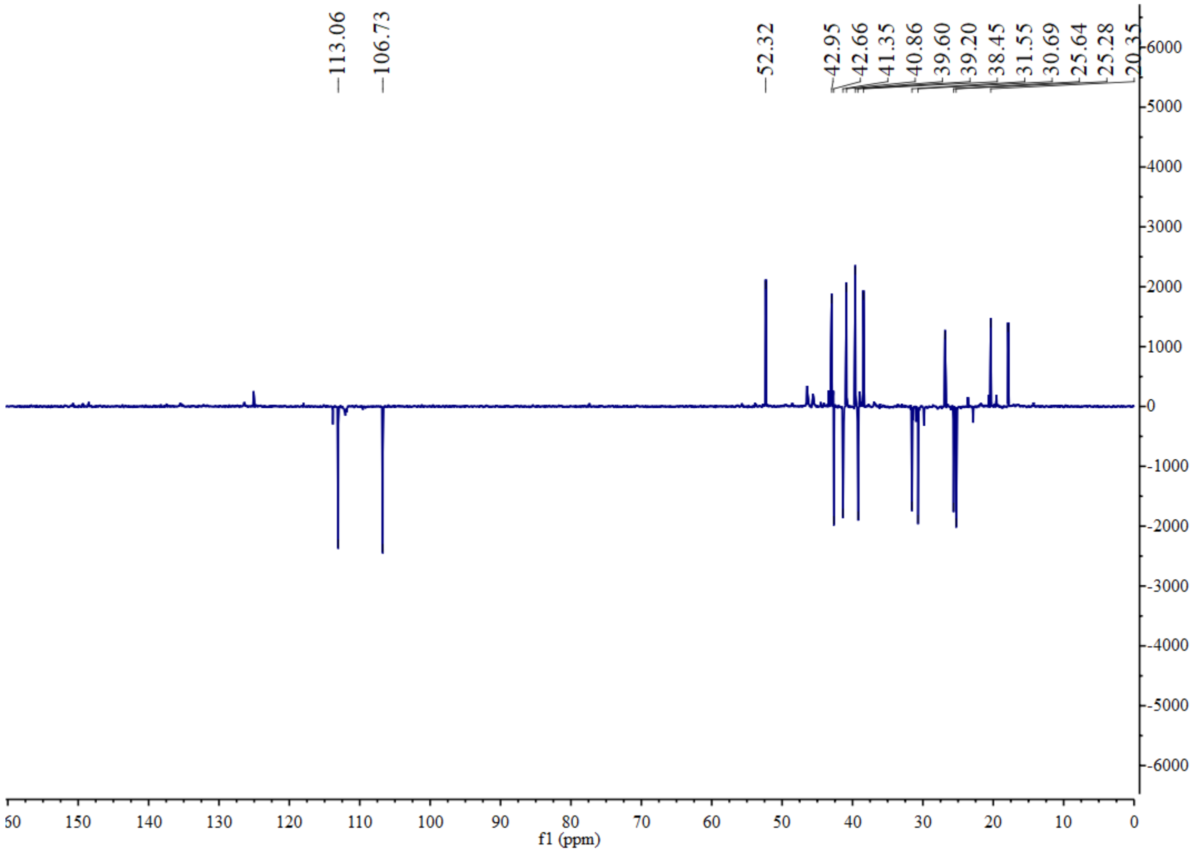

Figure S44 DEPT-135 NMR spectrum of **6′** in $CDCl_3$ (100 MHz).

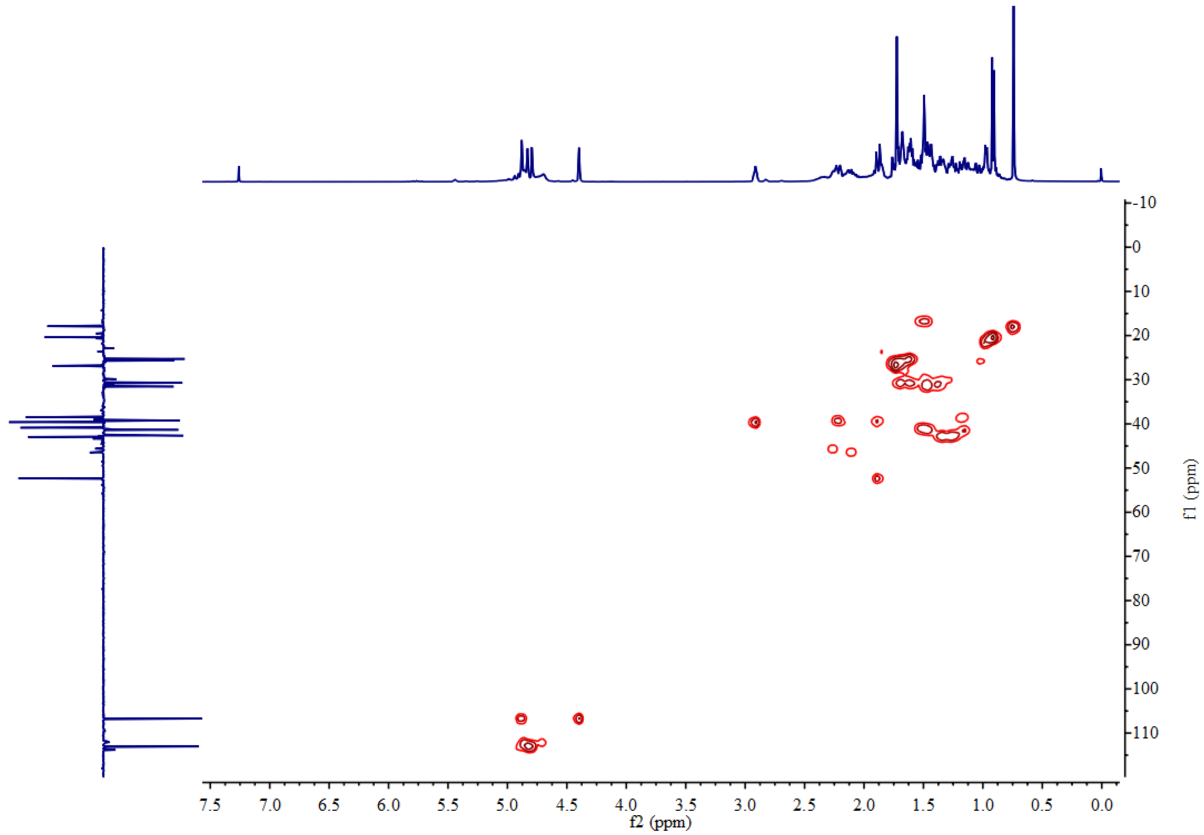

Figure S45 HSQC spectrum of **6′** in $CDCl_3$ (400 MHz).

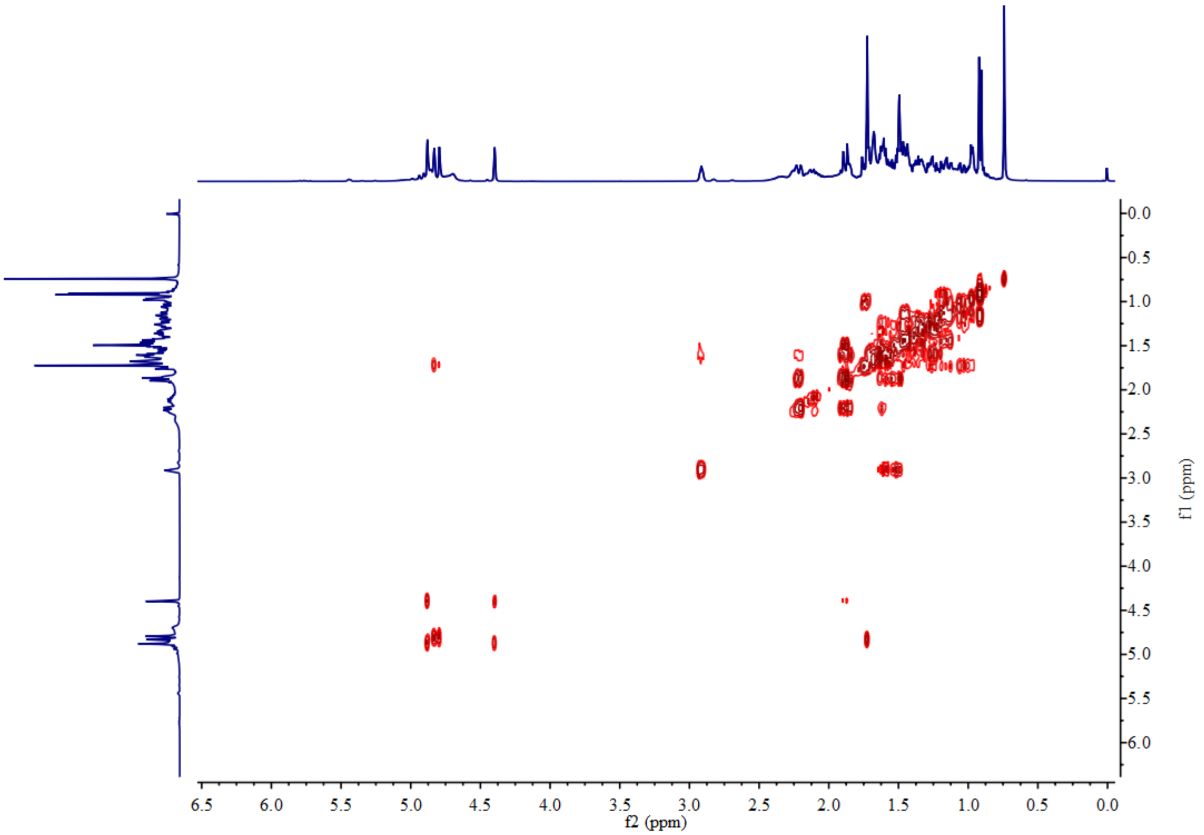

Figure S46 ^{1}H-^{1}H COSY spectrum of **6′** in $CDCl_3$ (400 MHz).

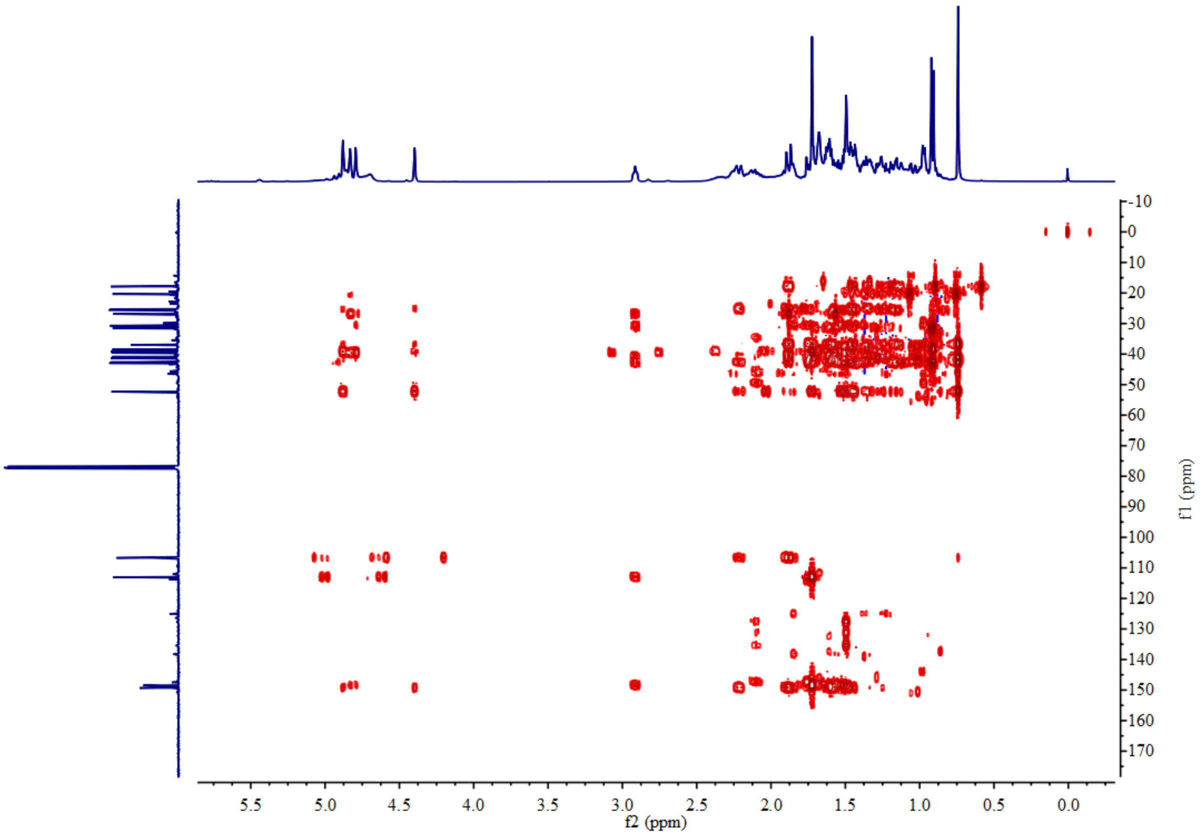

Figure S47 HMBC spectrum of **6′** in $CDCl_3$ (400 MHz).

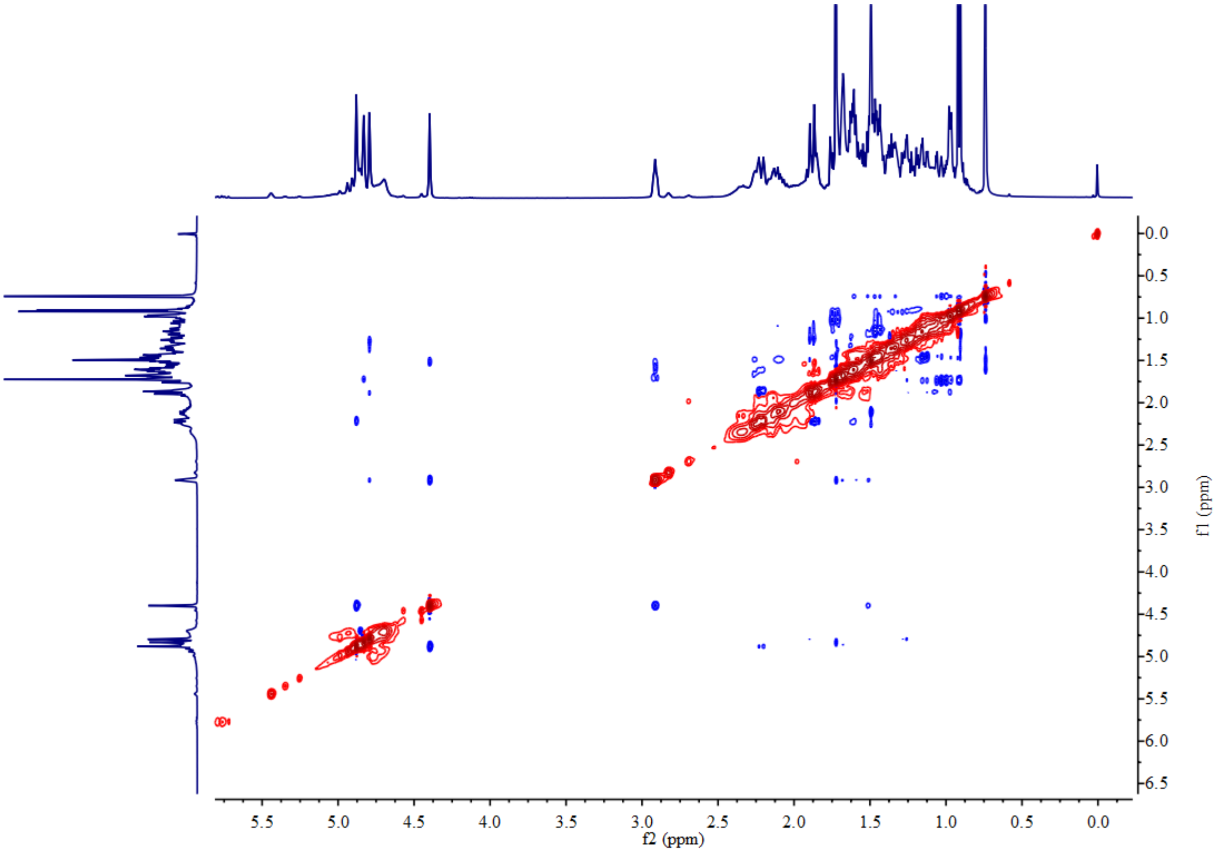

Figure S48 NOESY spectrum of **6′** in $CDCl_3$ (400 MHz).

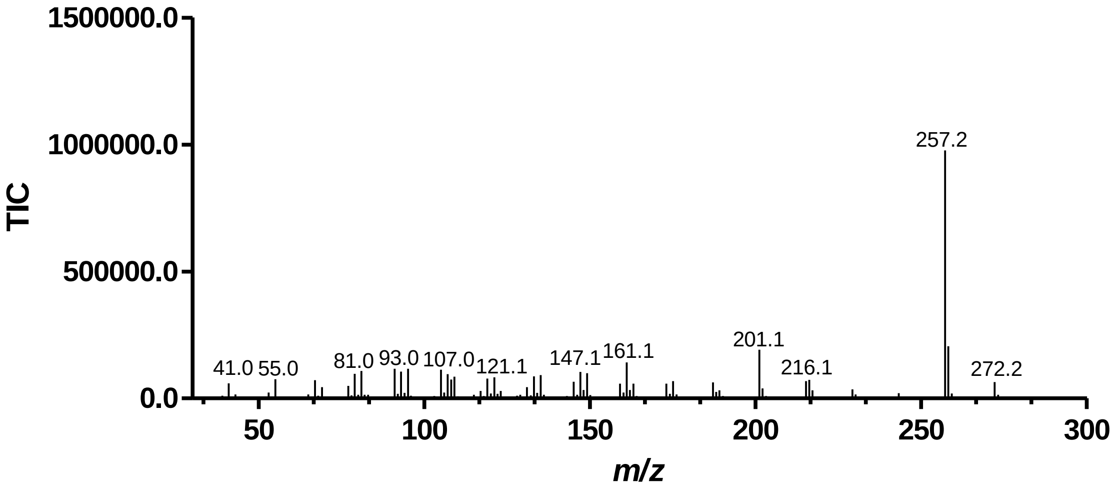

Figure S49 EI mass spectrum of **6′**.

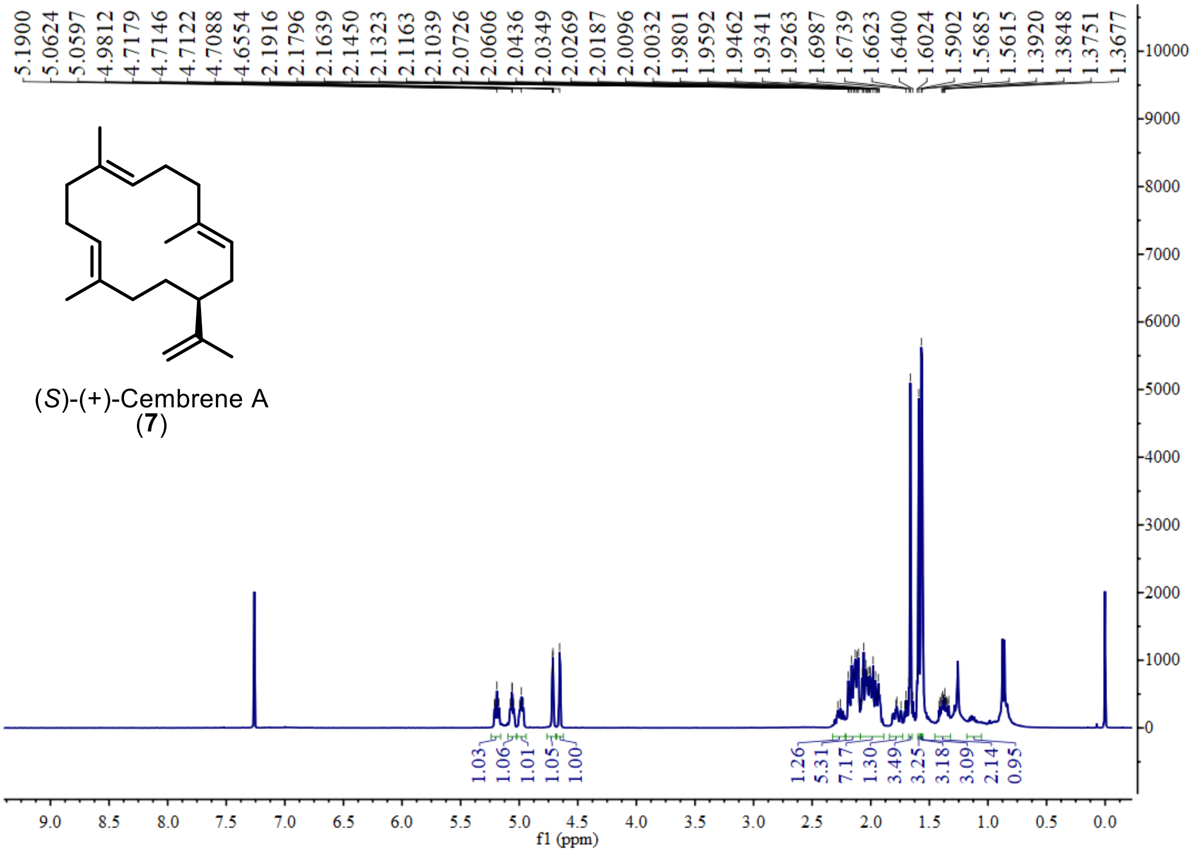

Figure S50 ^{1}H NMR spectrum of **7** in $CDCl_3$ (400 MHz).

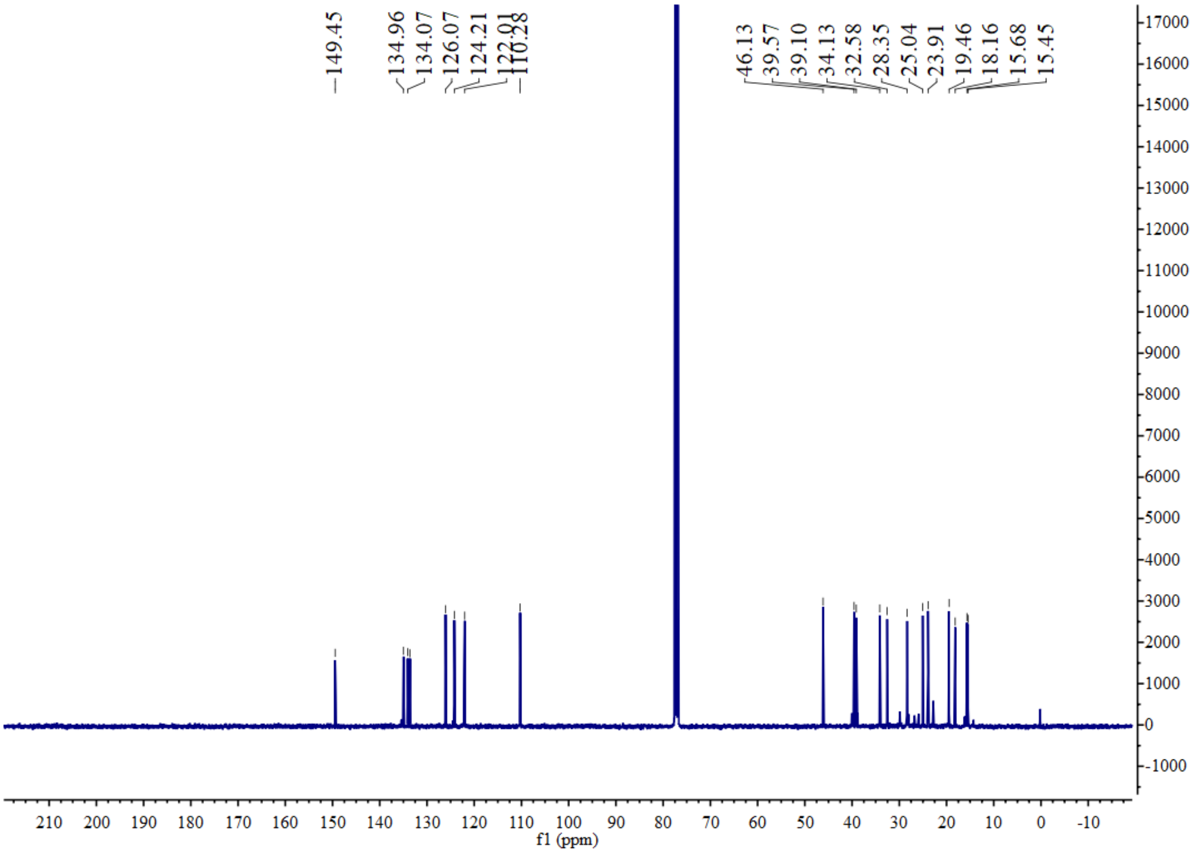

Figure S51 ^{13}C NMR spectrum of **7** in $CDCl_3$ (100 MHz).

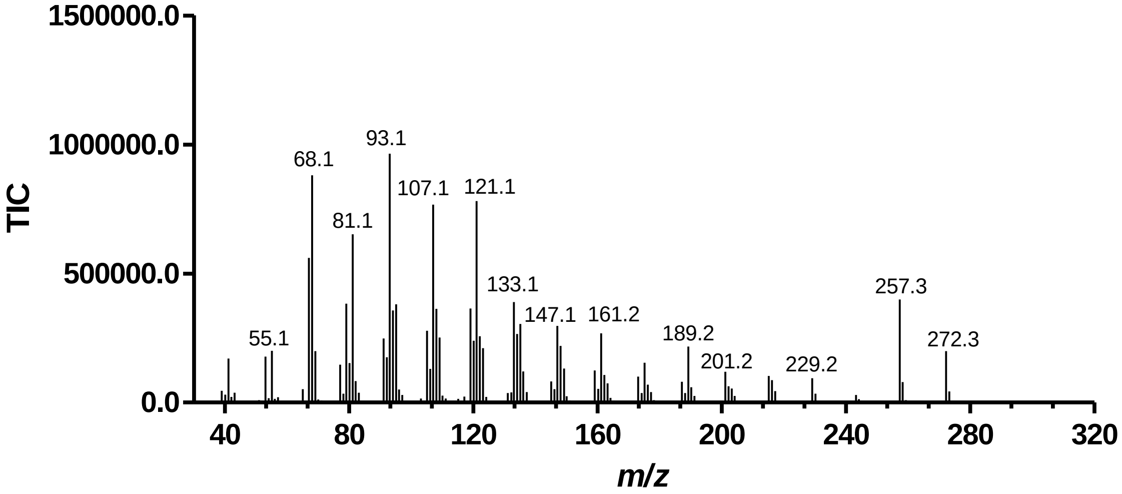

Figure S52 EI mass spectrum of **7**.

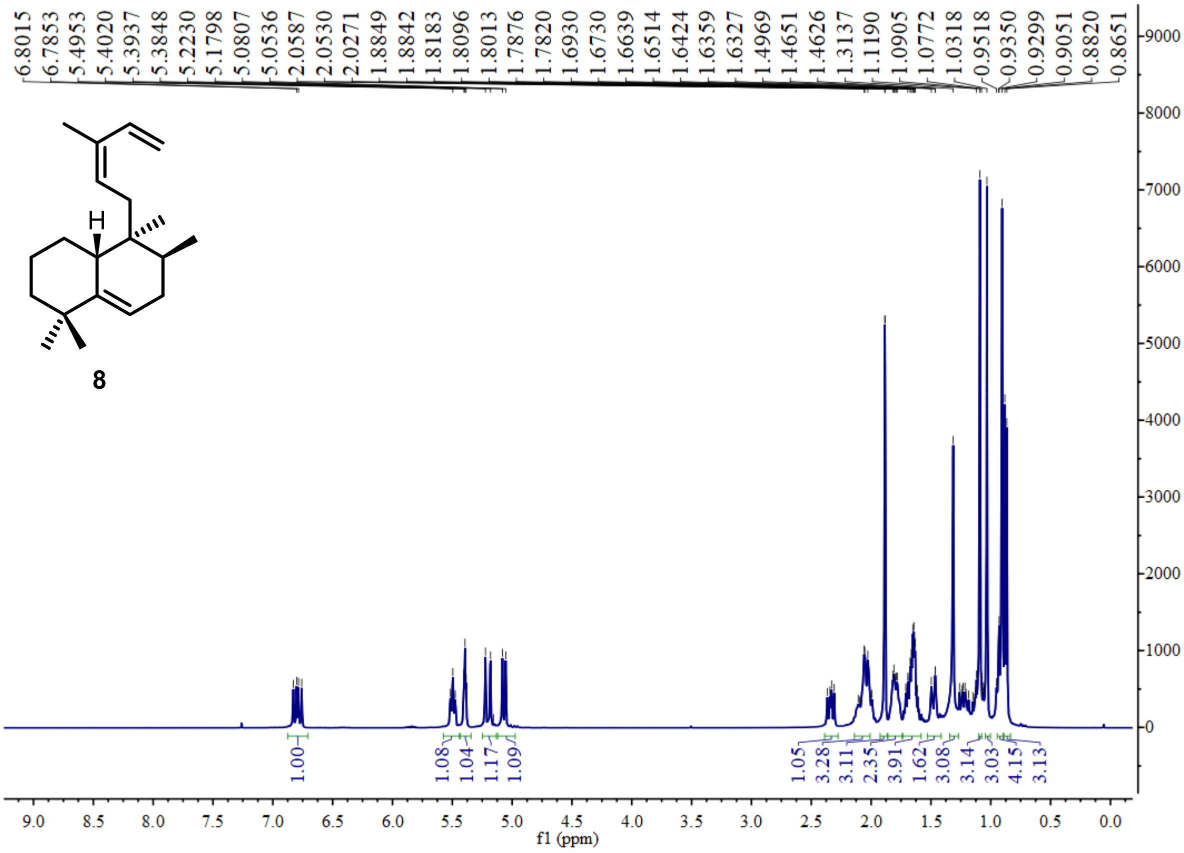

Figure S53 ^{1}H NMR spectrum of **8** in $CDCl_3$ (400 MHz).

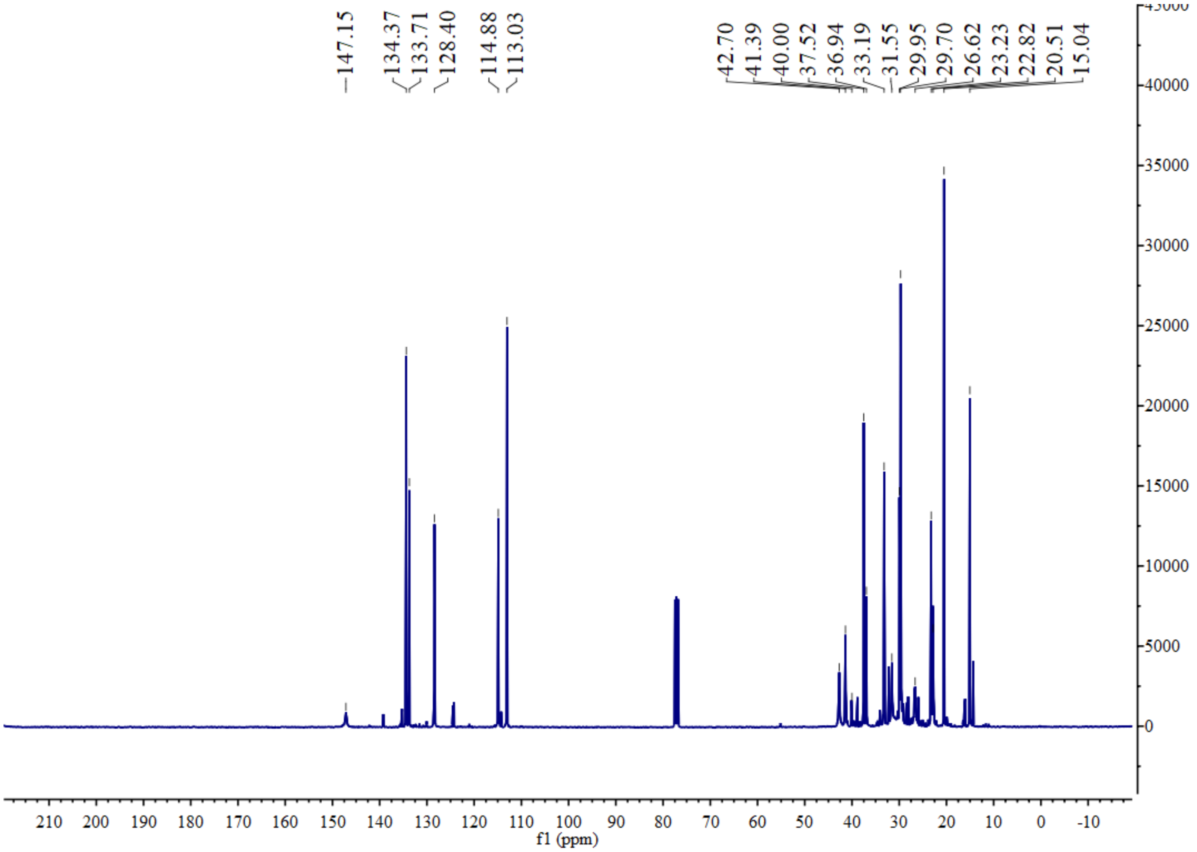

Figure S54 ${}^{13}C$ NMR spectrum of **8** in $CDCl_3$ (100 MHz).

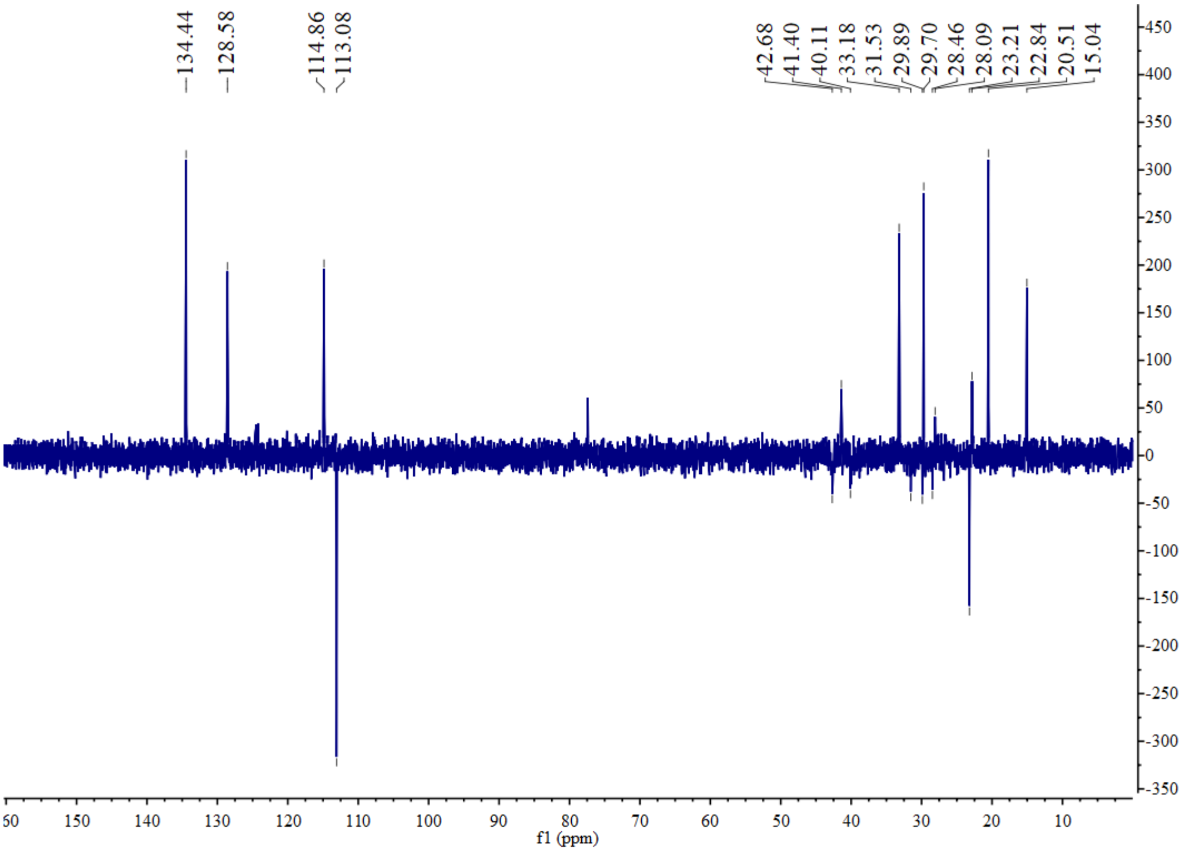

Figure S55 DEPT-135 NMR spectrum of **8** in $CDCl_3$ (100 MHz).

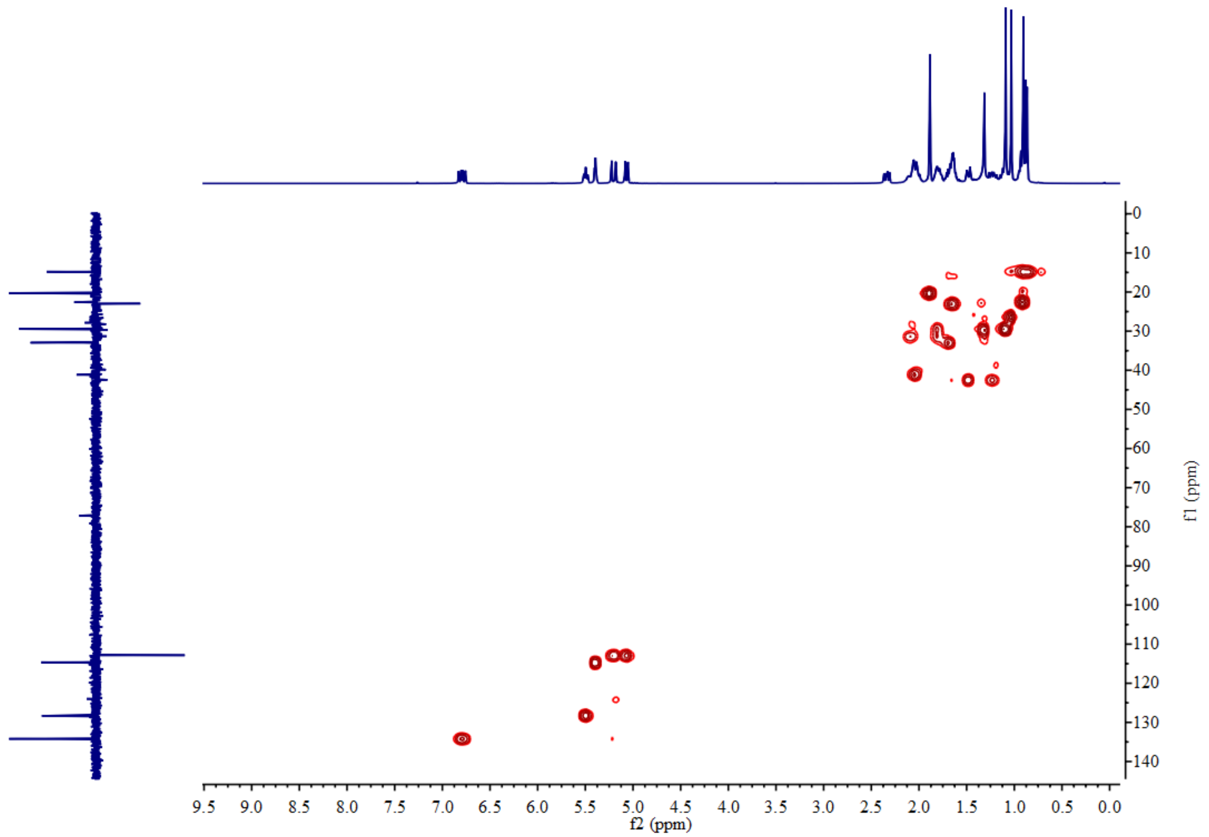

Figure S56 HSQC spectrum of **8** in $CDCl_3$ (400 MHz).

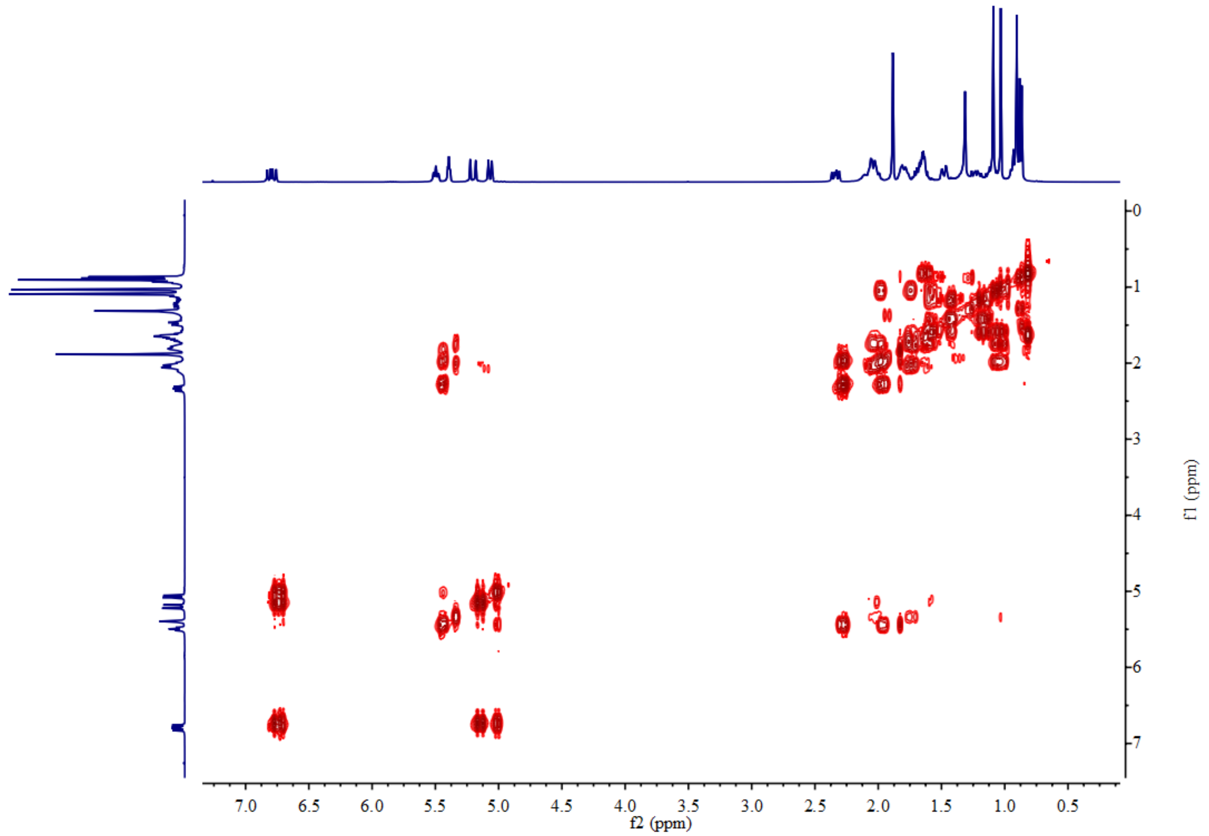

Figure S57 ^1H-^1H COSY spectrum of **8** in $CDCl_3$ (400 MHz).

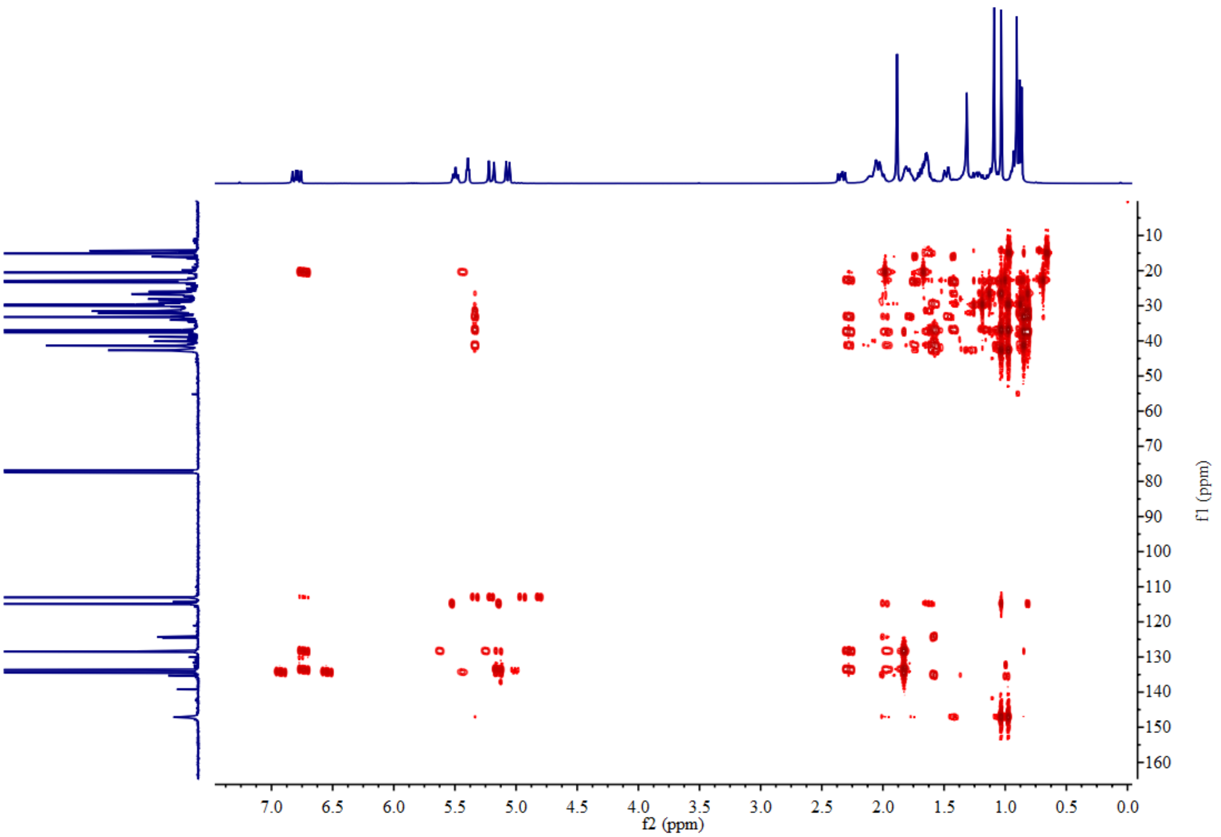

Figure S58 HMBC spectrum of **8** in $CDCl_3$ (400 MHz).

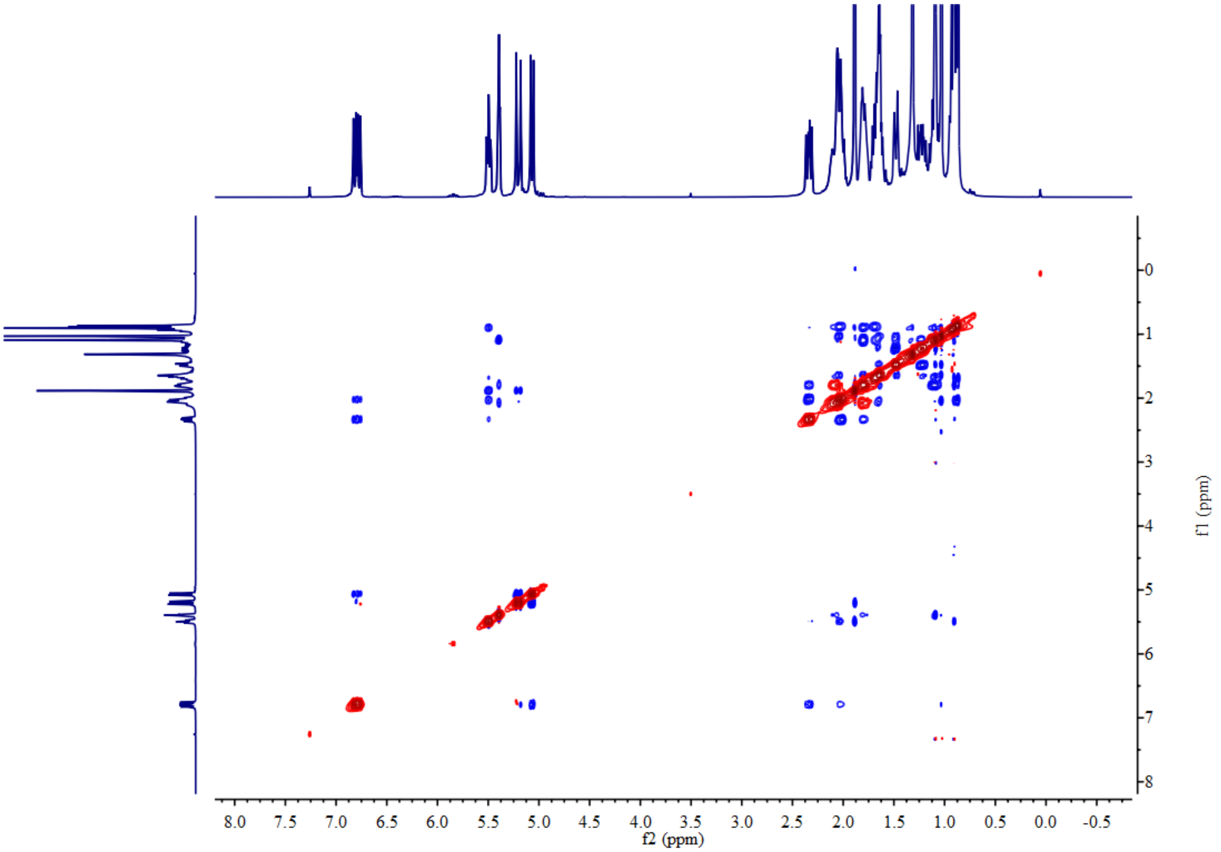

Figure S59 NOESY spectrum of **8** in $CDCl_3$ (400 MHz).

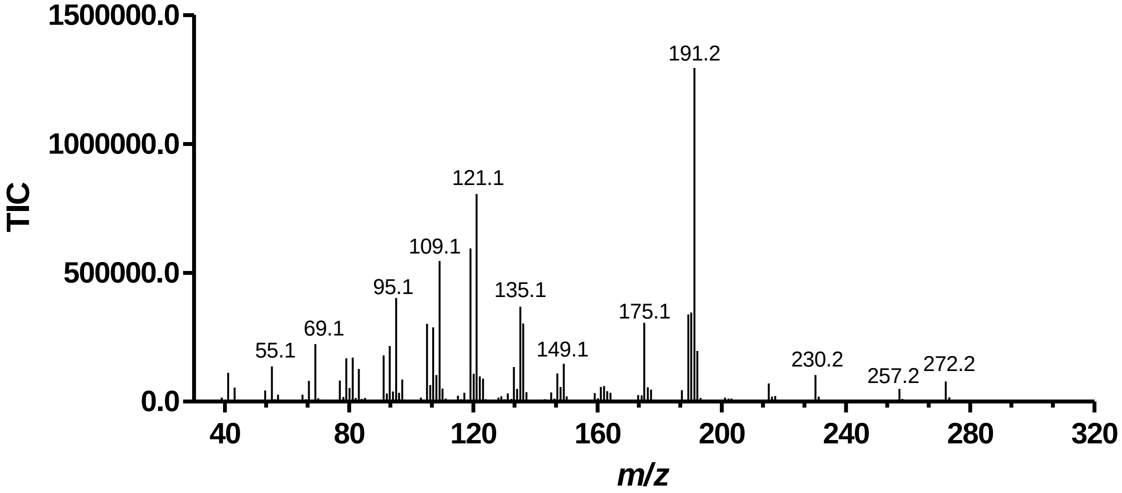

Figure S60 EI mass spectrum of **8**.

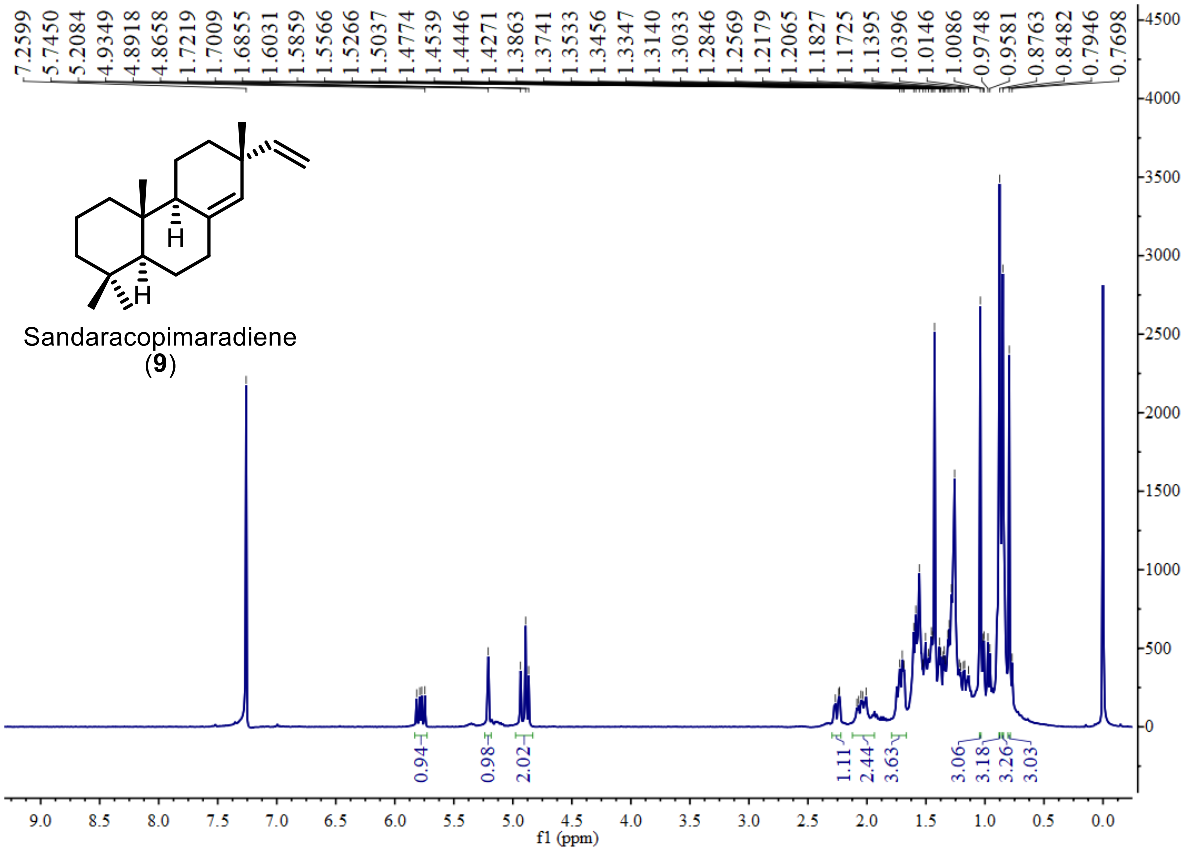

Figure S61 ^{1}H NMR spectrum of **9** in $CDCl_3$ (400 MHz).

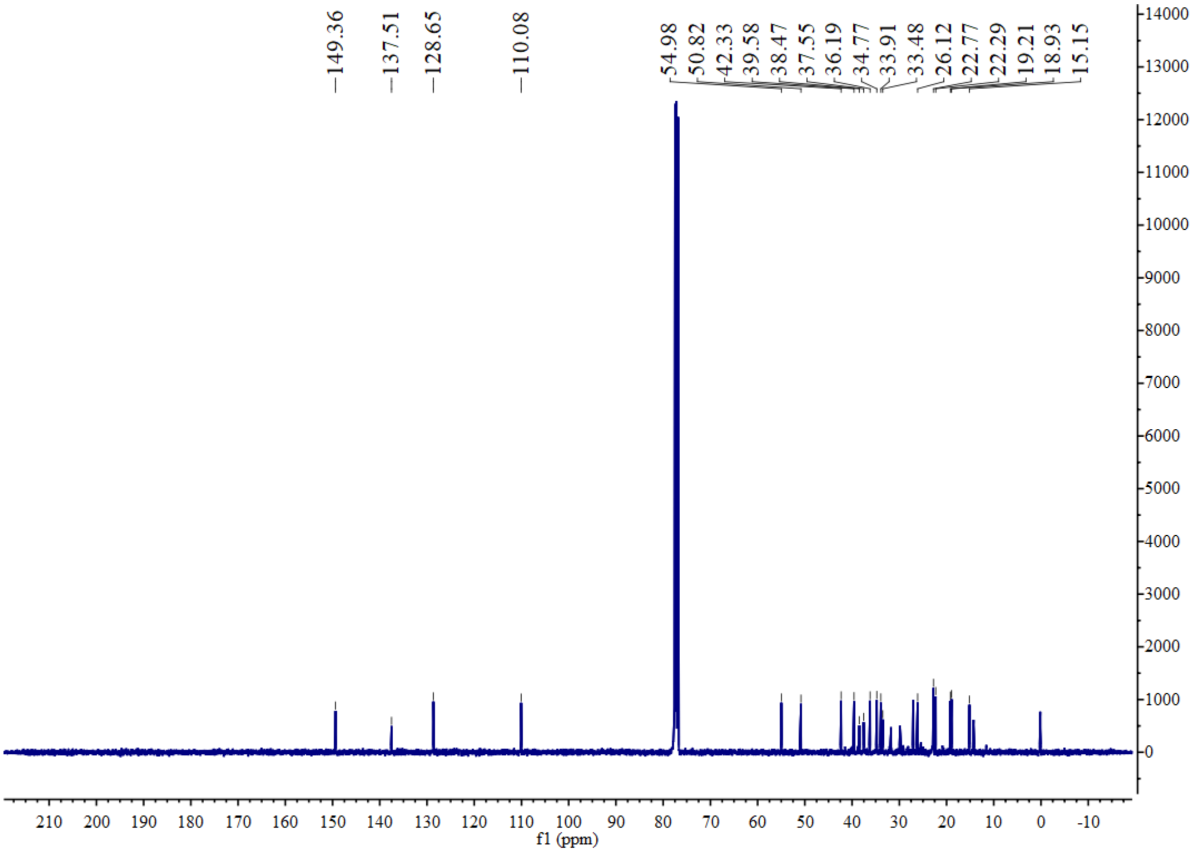

Figure S62 ^{13}C NMR spectrum of **9** in $CDCl_3$ (100 MHz).

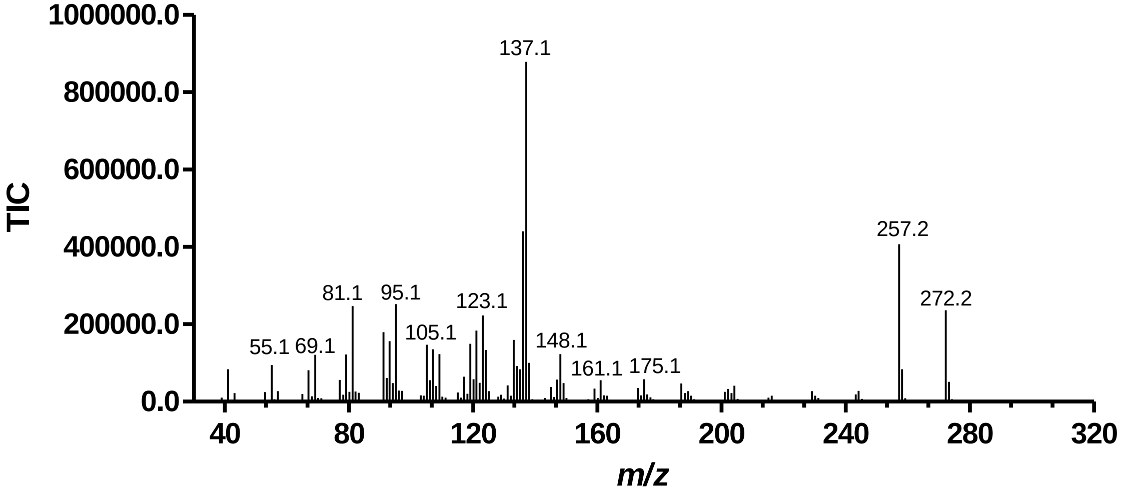

Figure S63 EI mass spectrum of **9**.

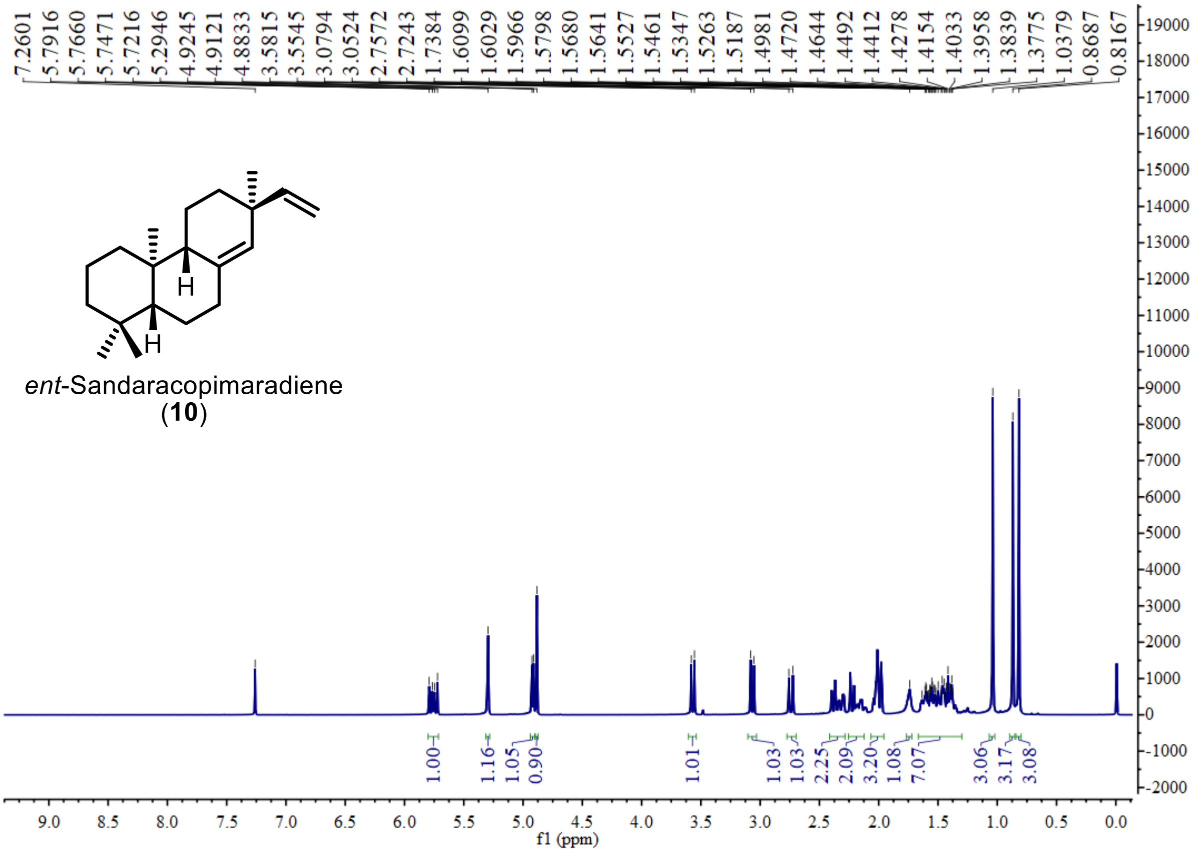

Figure S64 ^{1}H NMR spectrum of **10** in $CDCl_3$ (400 MHz).

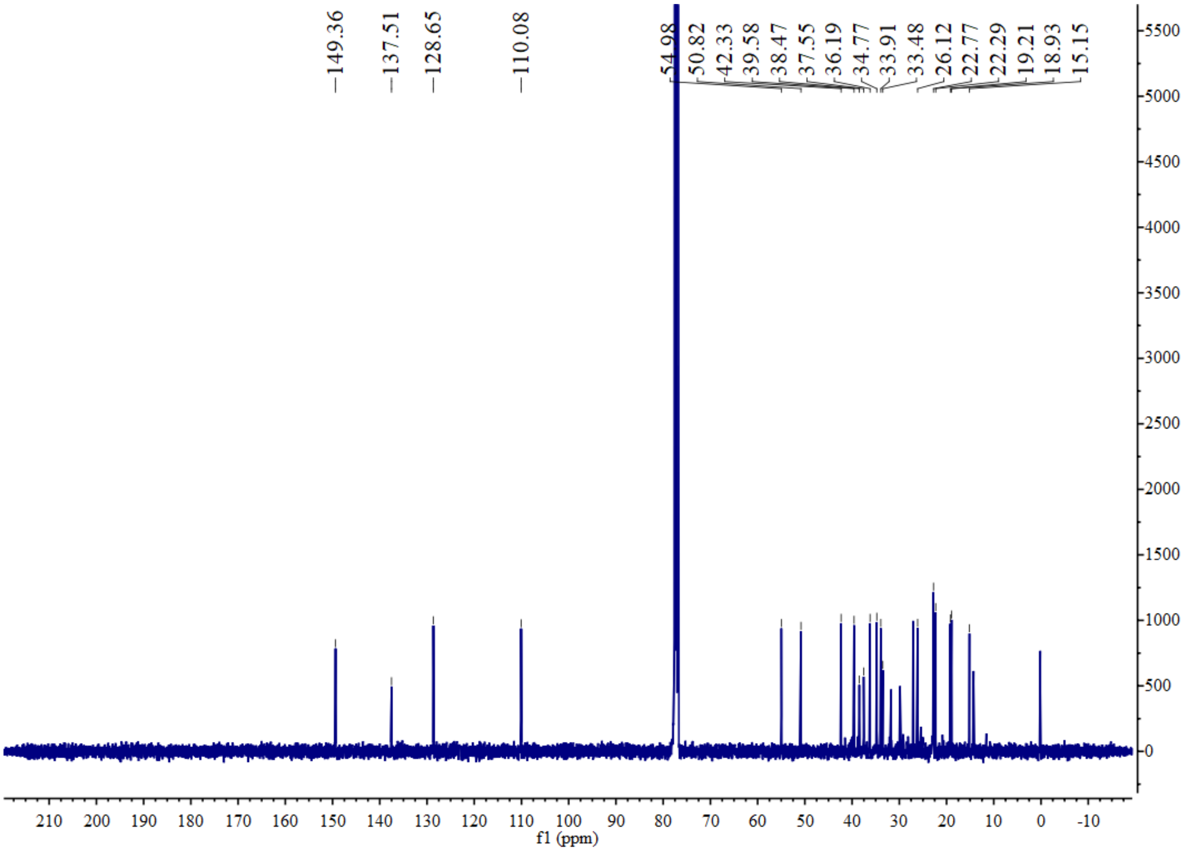

Figure S65 ^{13}C NMR spectrum of **10** in $CDCl_3$ (100 MHz).

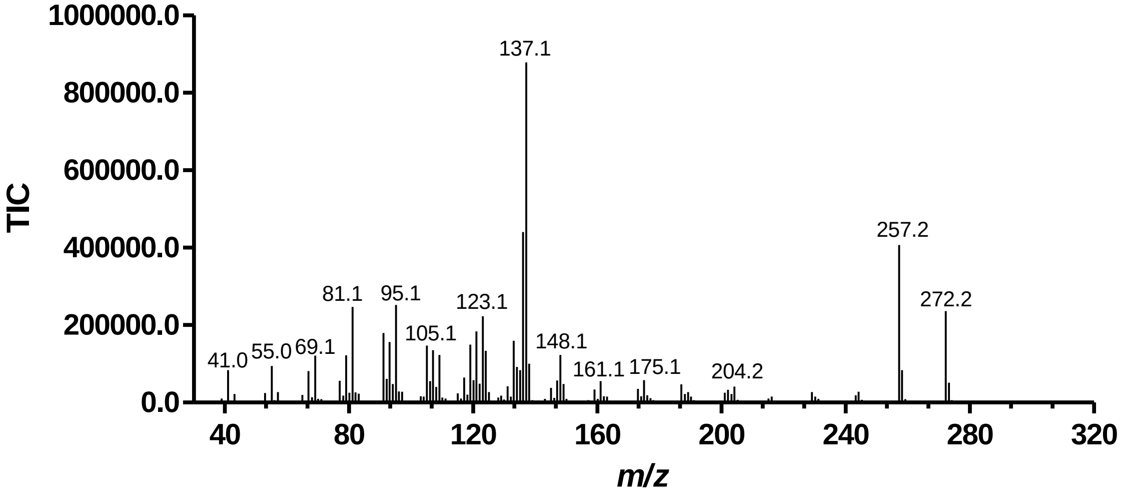

Figure S66 EI mass spectrum of **10**.

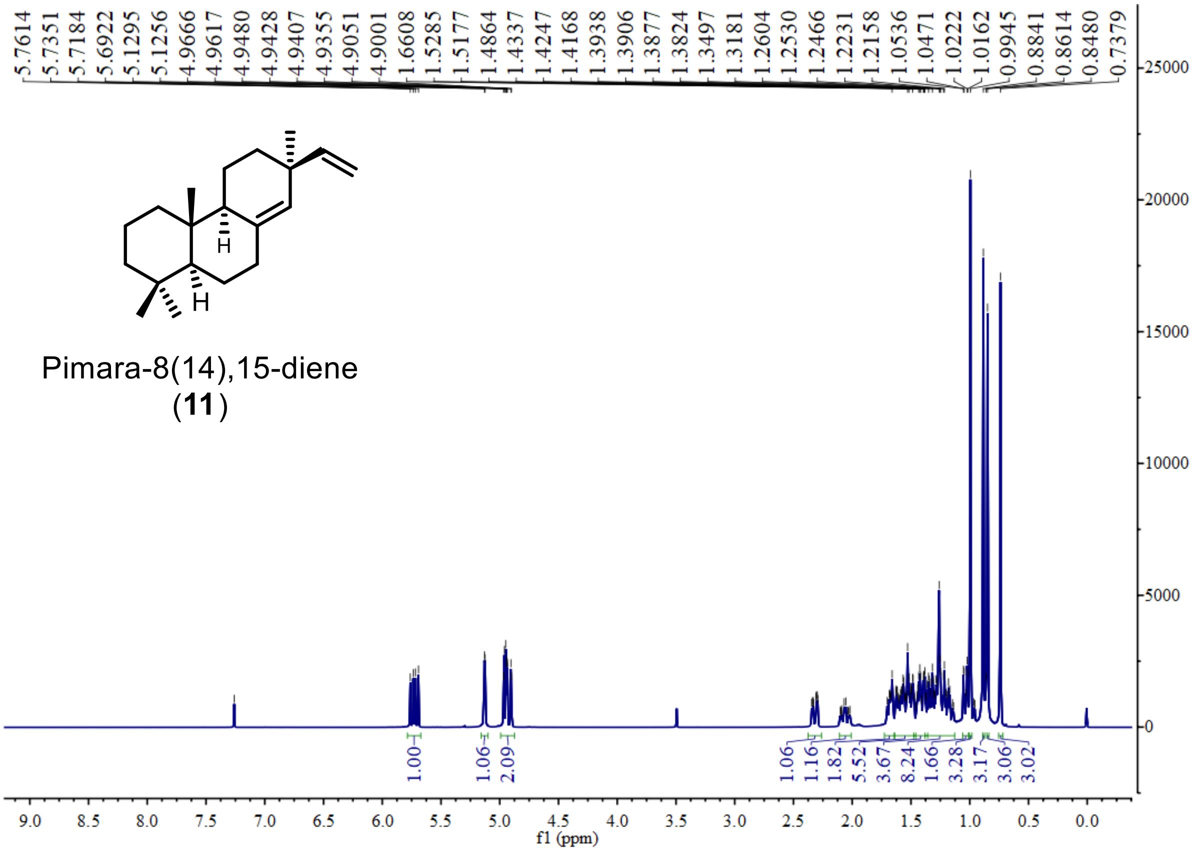

Figure S67 ^{1}H NMR spectrum of **11** in $CDCl_3$ (400 MHz).

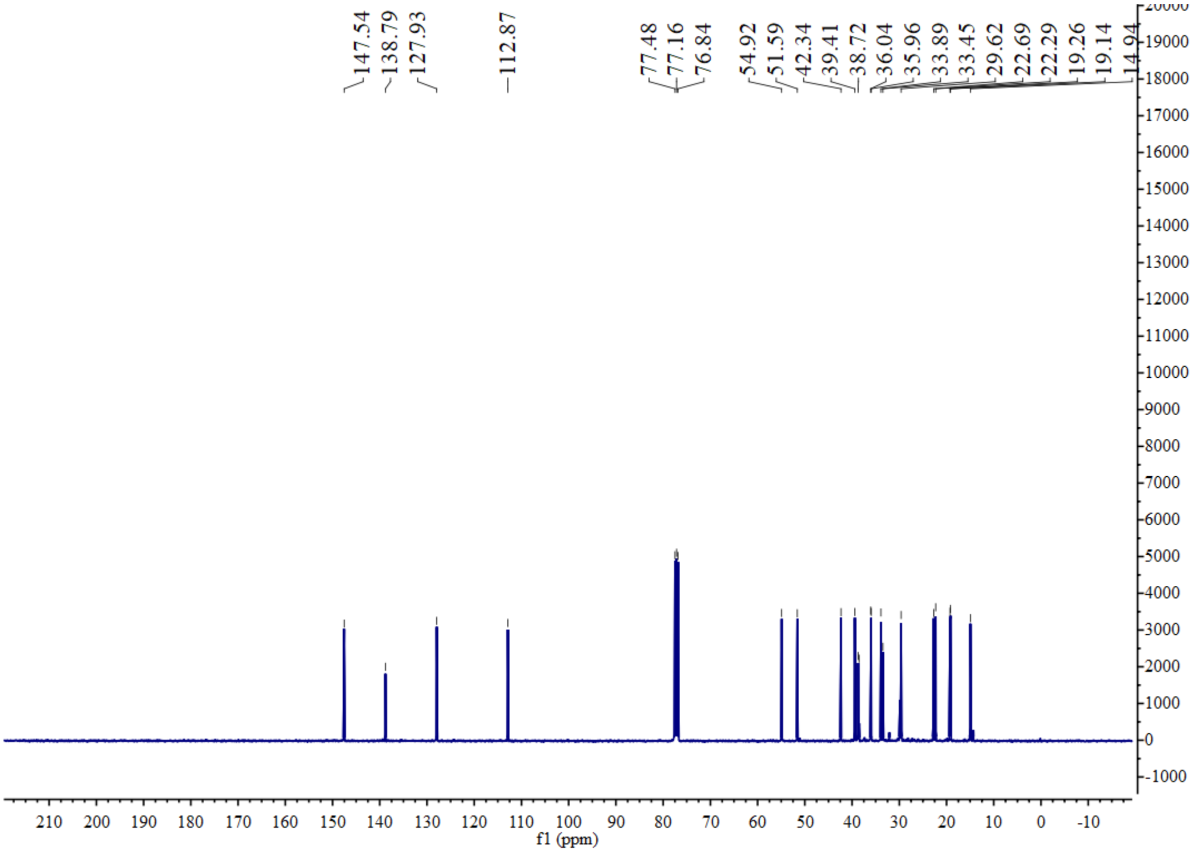

Figure S68 ^{13}C NMR spectrum of **11** in $CDCl_3$ (100 MHz).

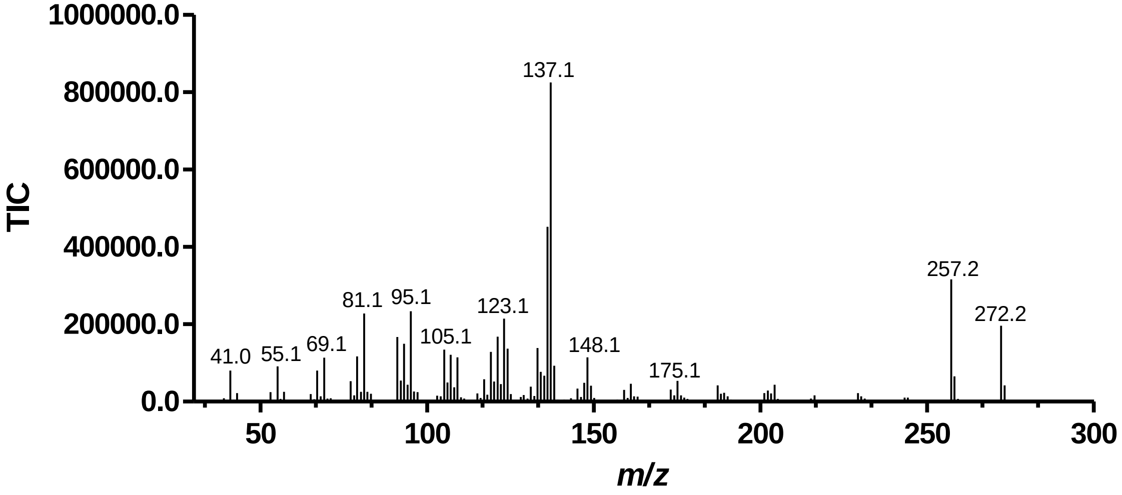

Figure S69 EI mass spectrum of **11**.

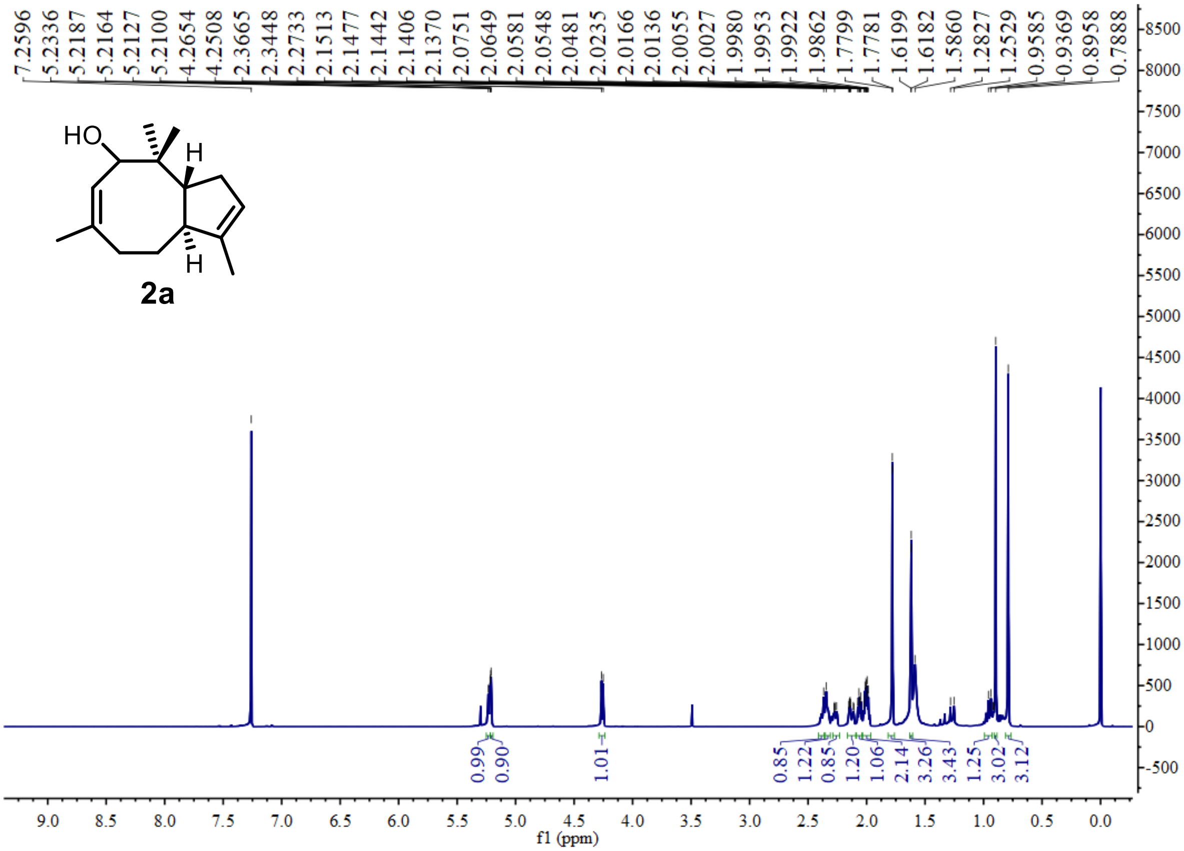

Figure S70 ^{1}H NMR spectrum of **2a** in $CDCl_3$ (400 MHz).

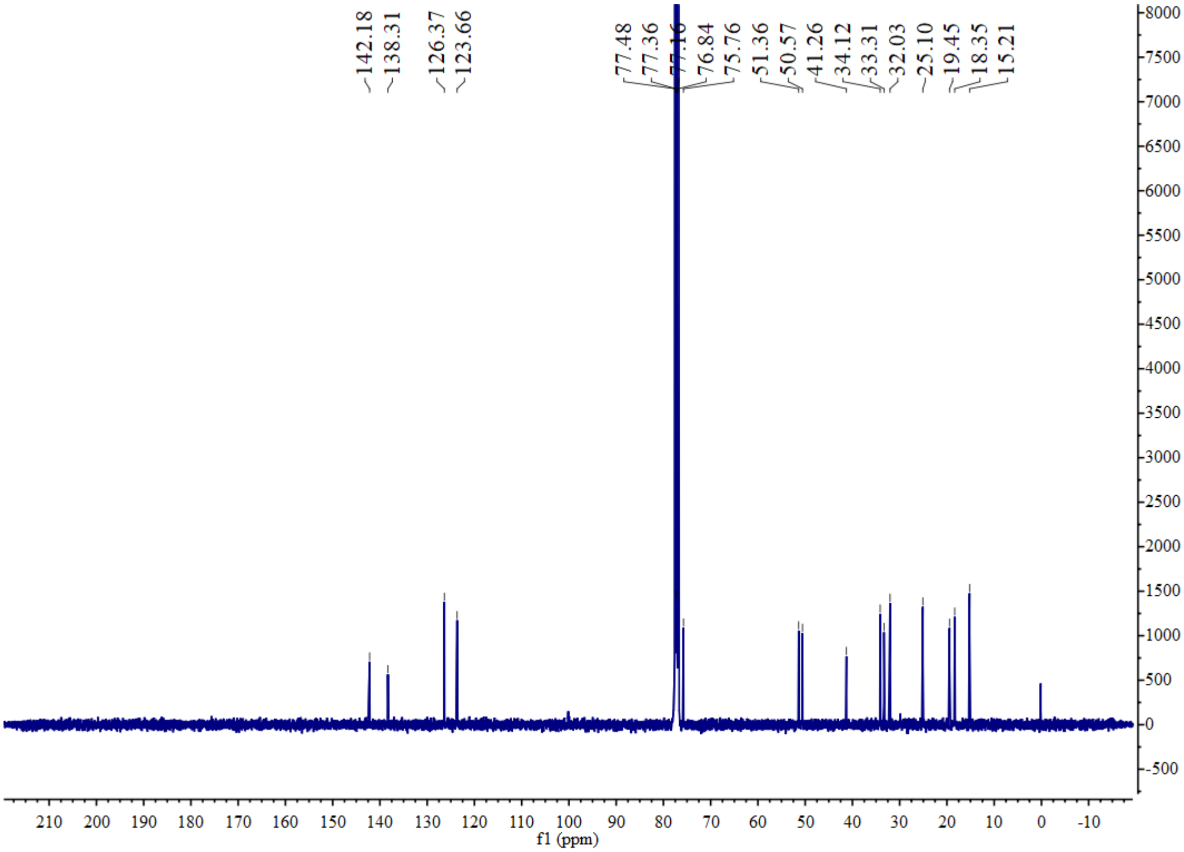

Figure S71 ^{13}C NMR spectrum of **2a** in $CDCl_3$ (100 MHz).

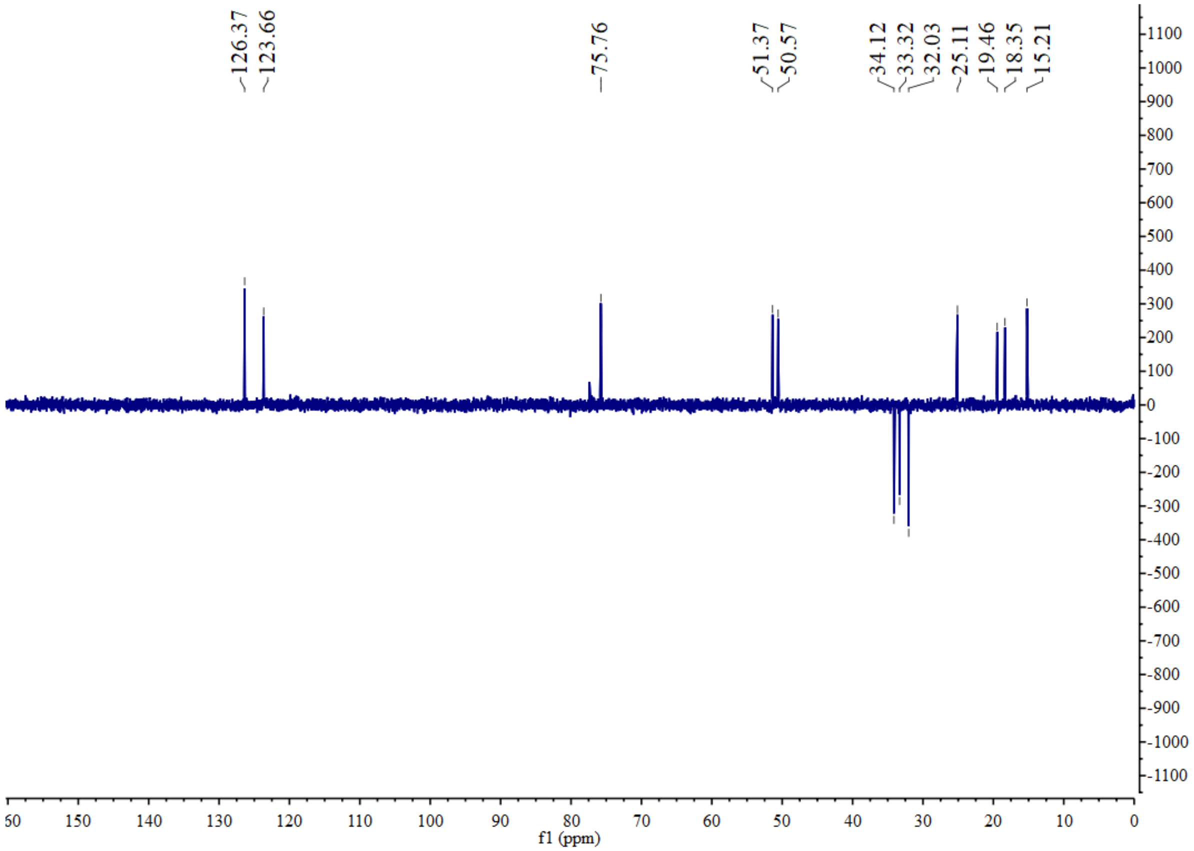

Figure S72 DEPT-135 NMR spectrum of **2a** in $CDCl_3$ (100 MHz).

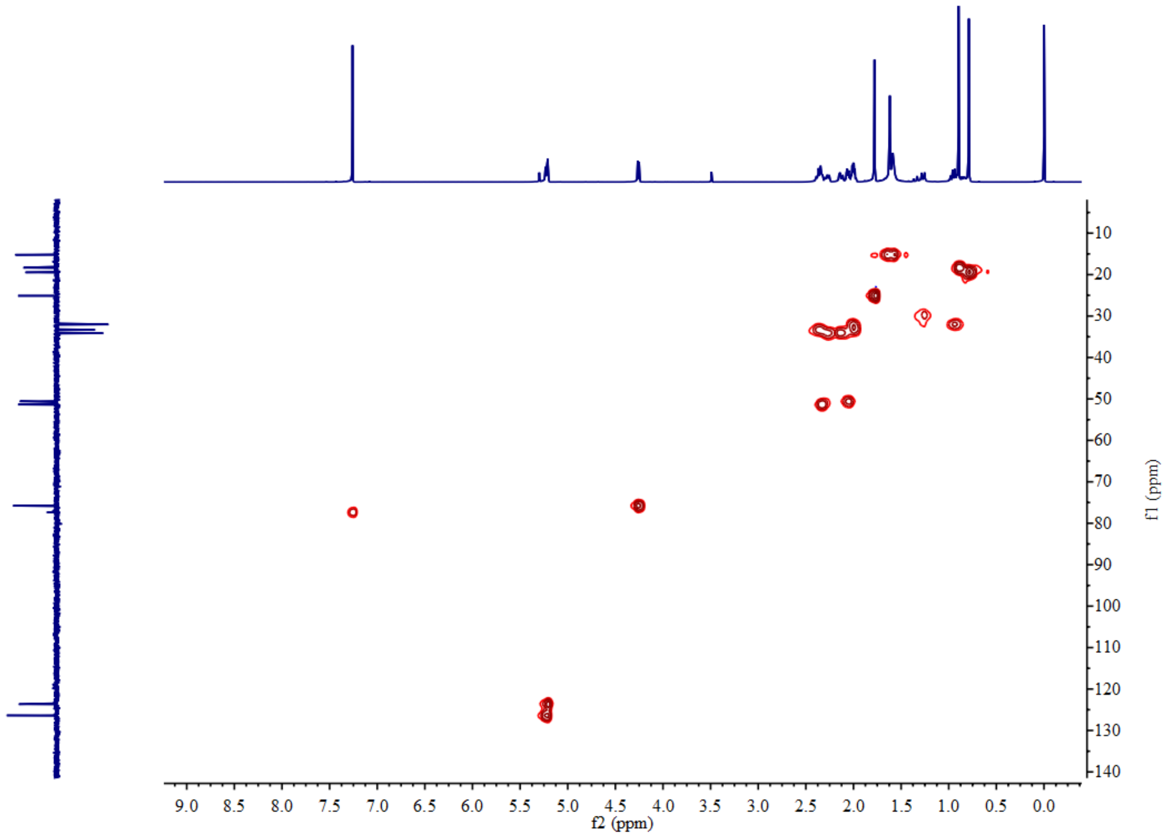

Figure S73 HSQC spectrum of **2a** in $CDCl_3$ (400 MHz).

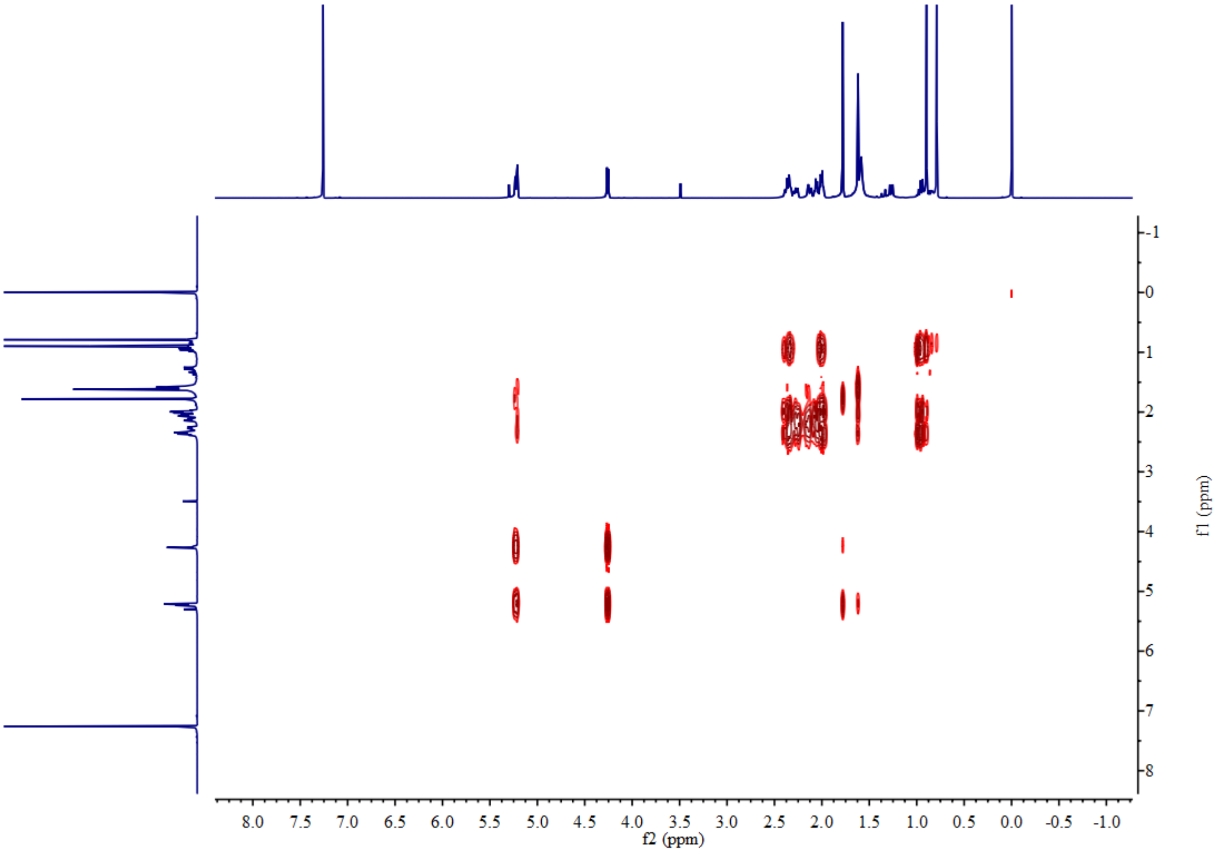

Figure S74 ^{1}H-^{1}H COSY spectrum of **2a** in $CDCl_3$ (400 MHz).

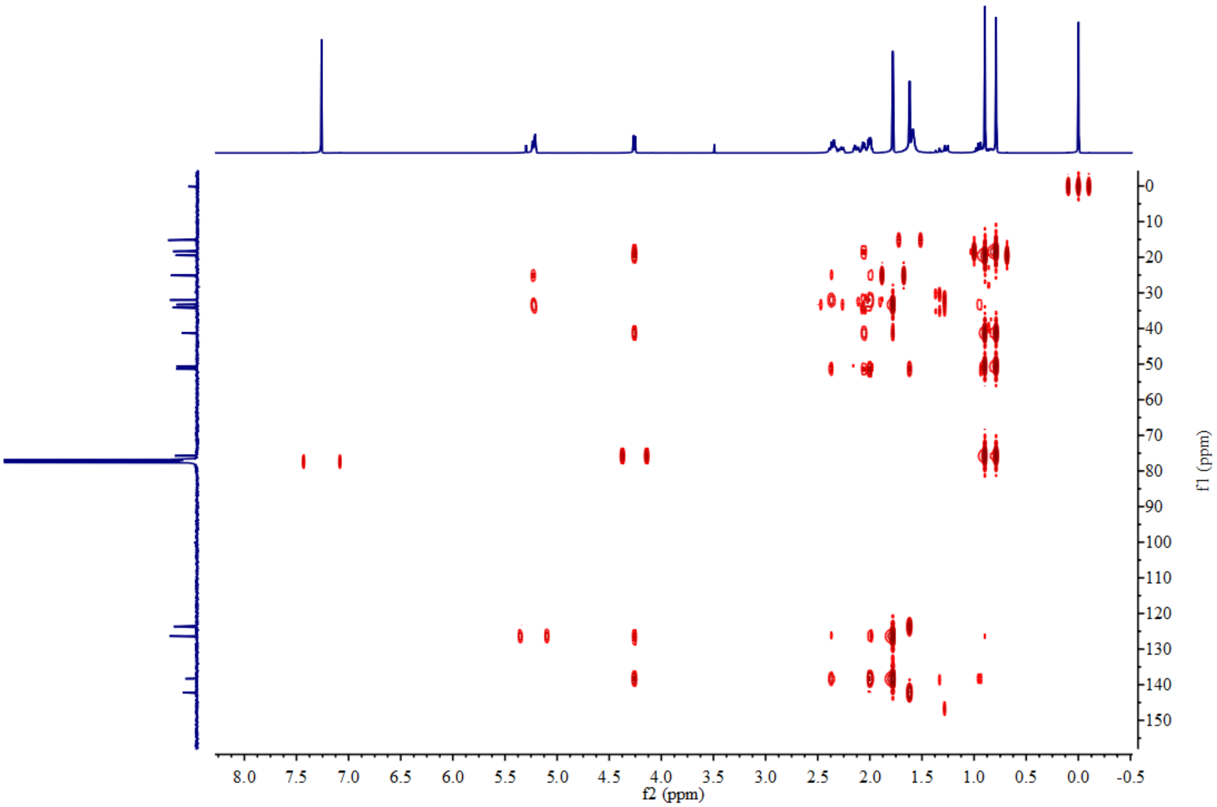

Figure S75 HMBC spectrum of **2a** in $CDCl_3$ (400 MHz).

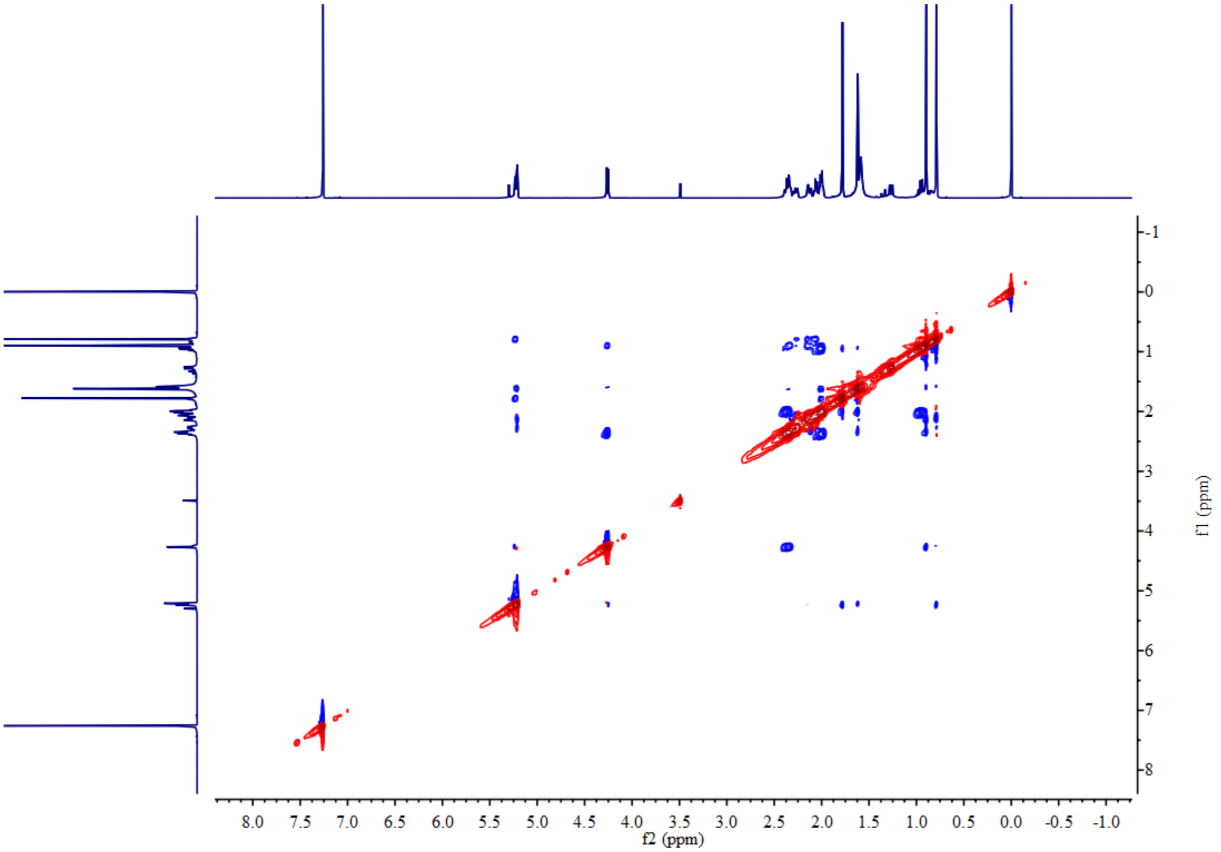

Figure S76 NOESY spectrum of **2a** in $CDCl_3$ (400 MHz).

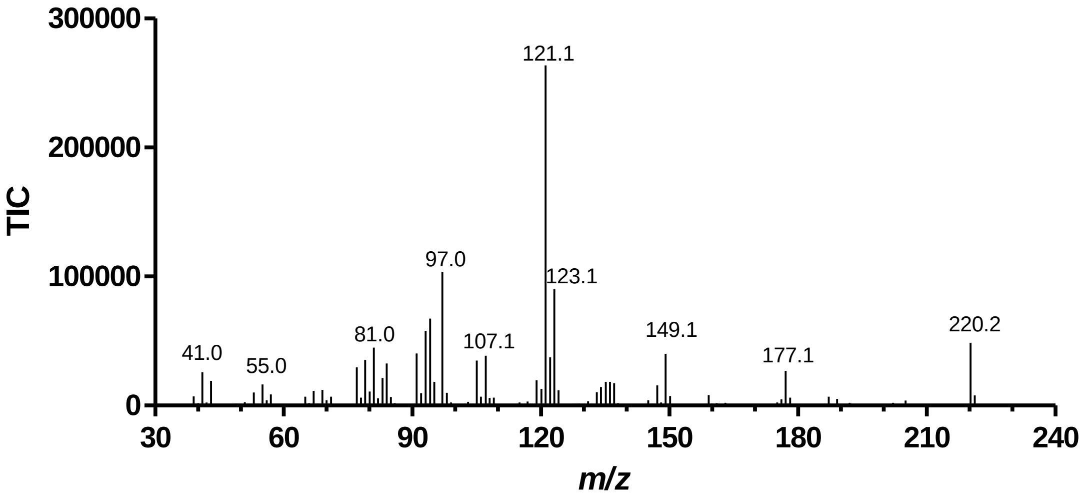

Figure S77 EI mass spectrum of **2a**.

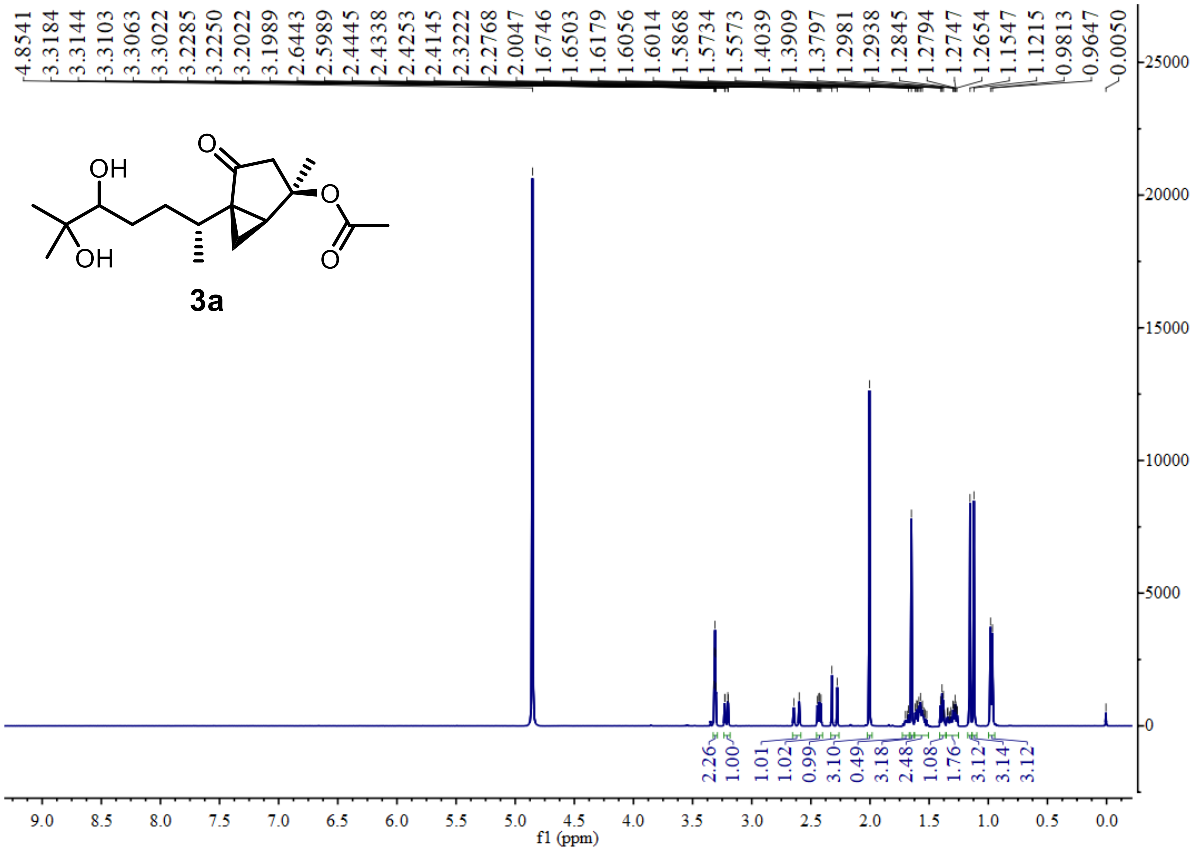

Figure S78 ^{1}H NMR spectrum of **3a** in methanol-d_4 (400 MHz).

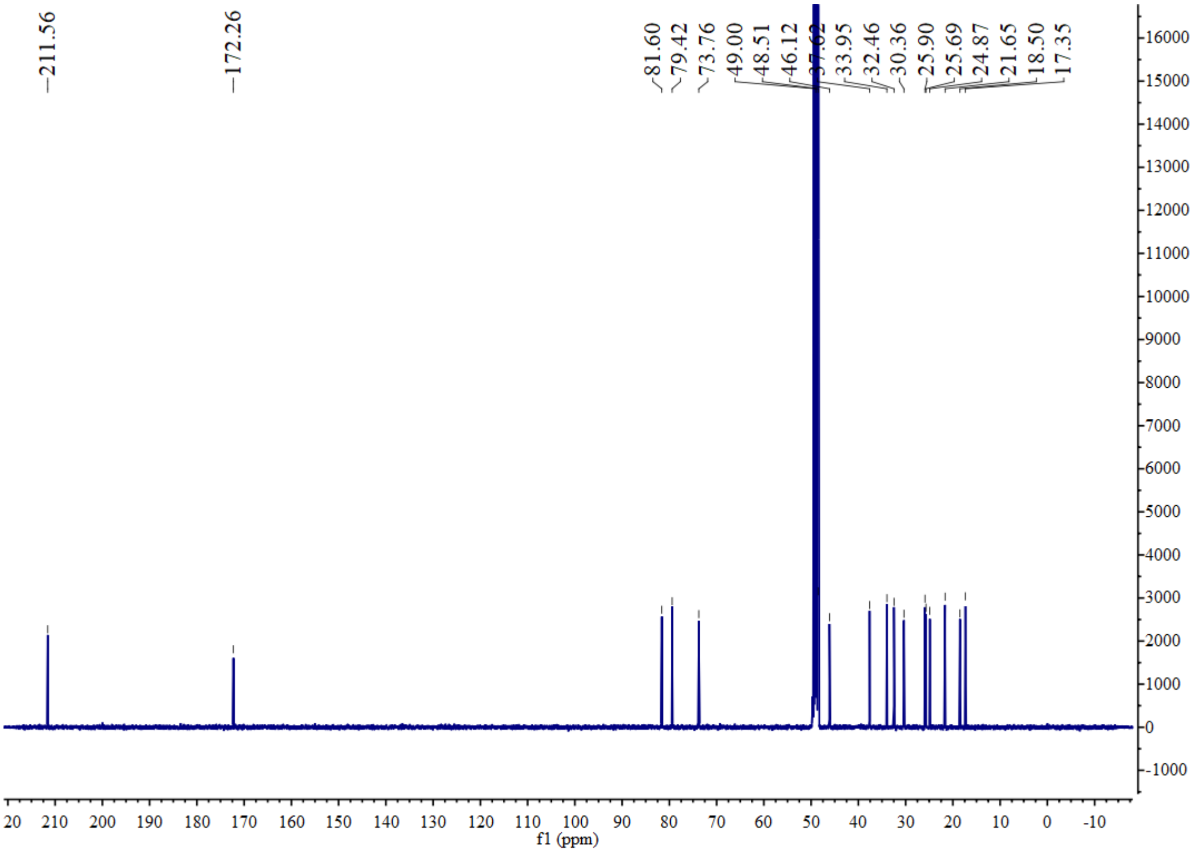

Figure S79 ^{13}C NMR spectrum of **3a** in methanol-d_4 (100 MHz).

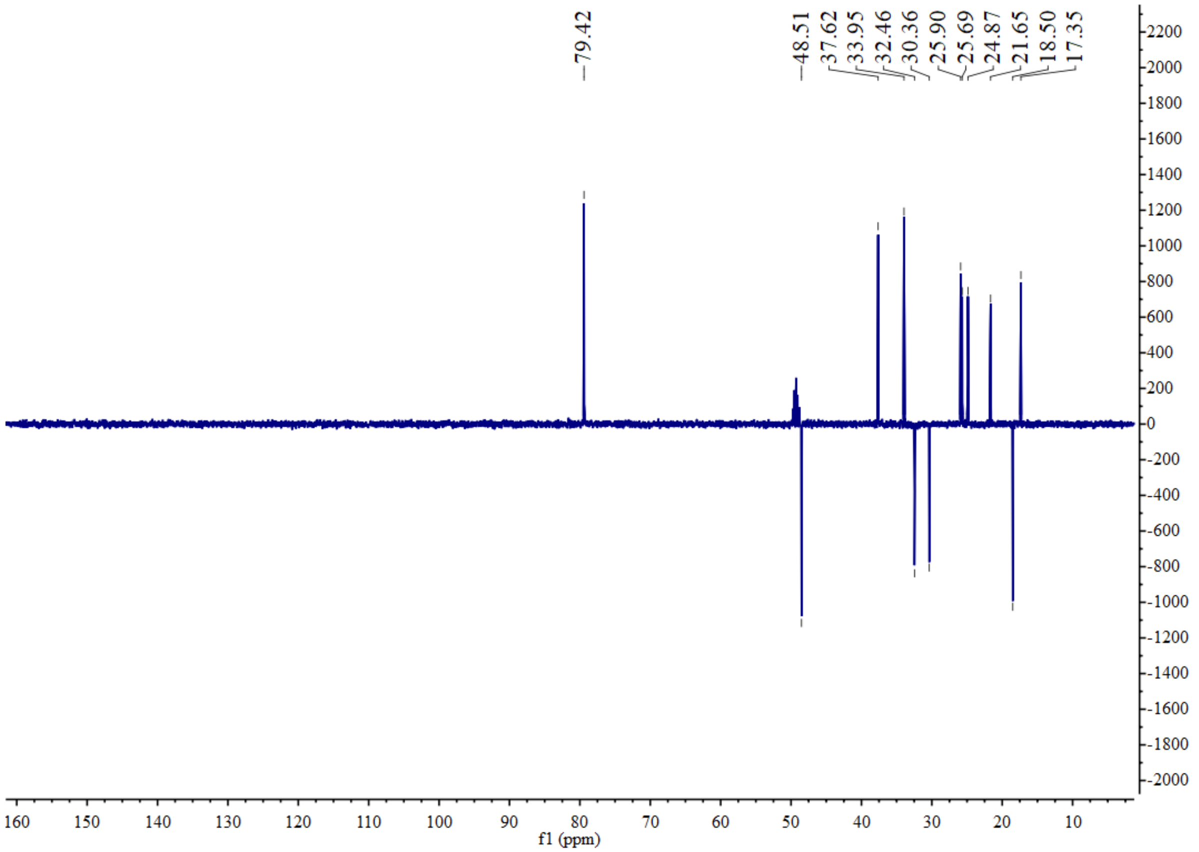

Figure S80 DEPT-135 NMR spectrum of **3a** in methanol-d_4 (100 MHz).

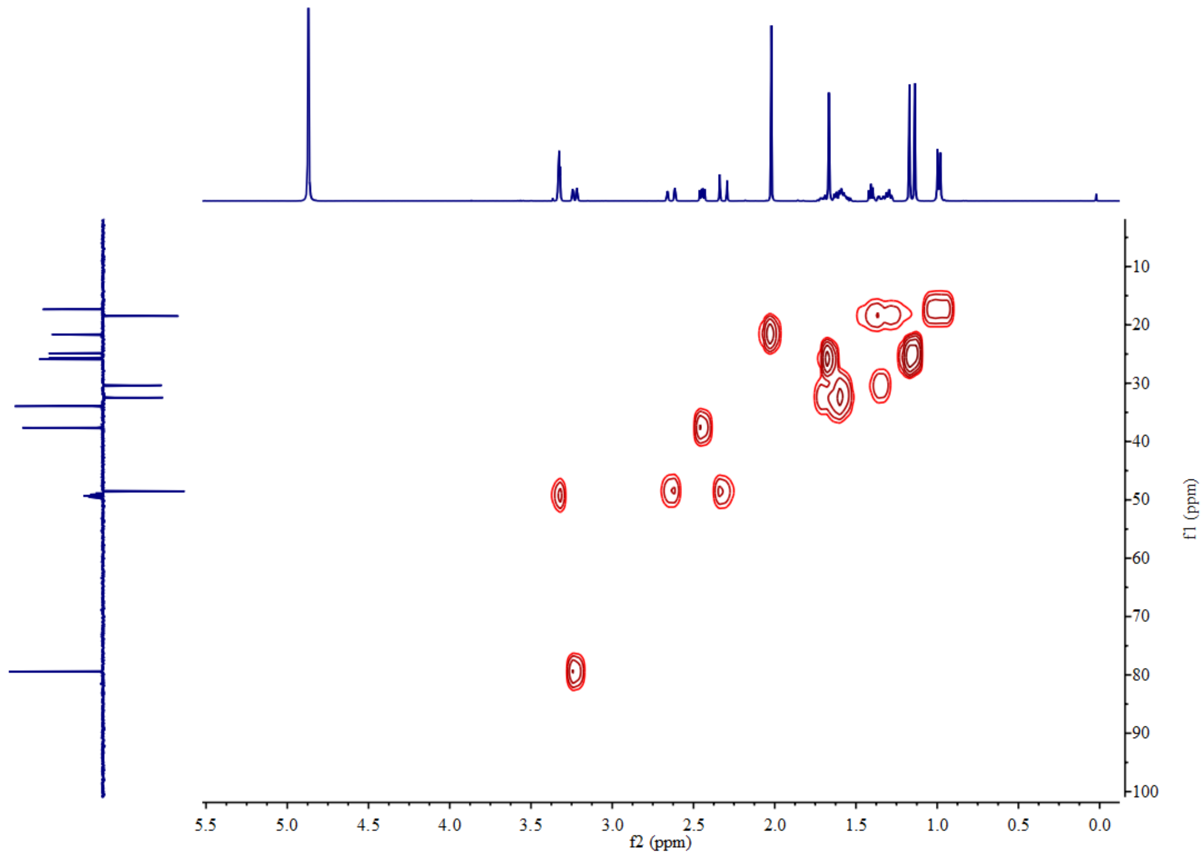

Figure S81 HSQC spectrum of **3a** in methanol-d_4 (400 MHz).

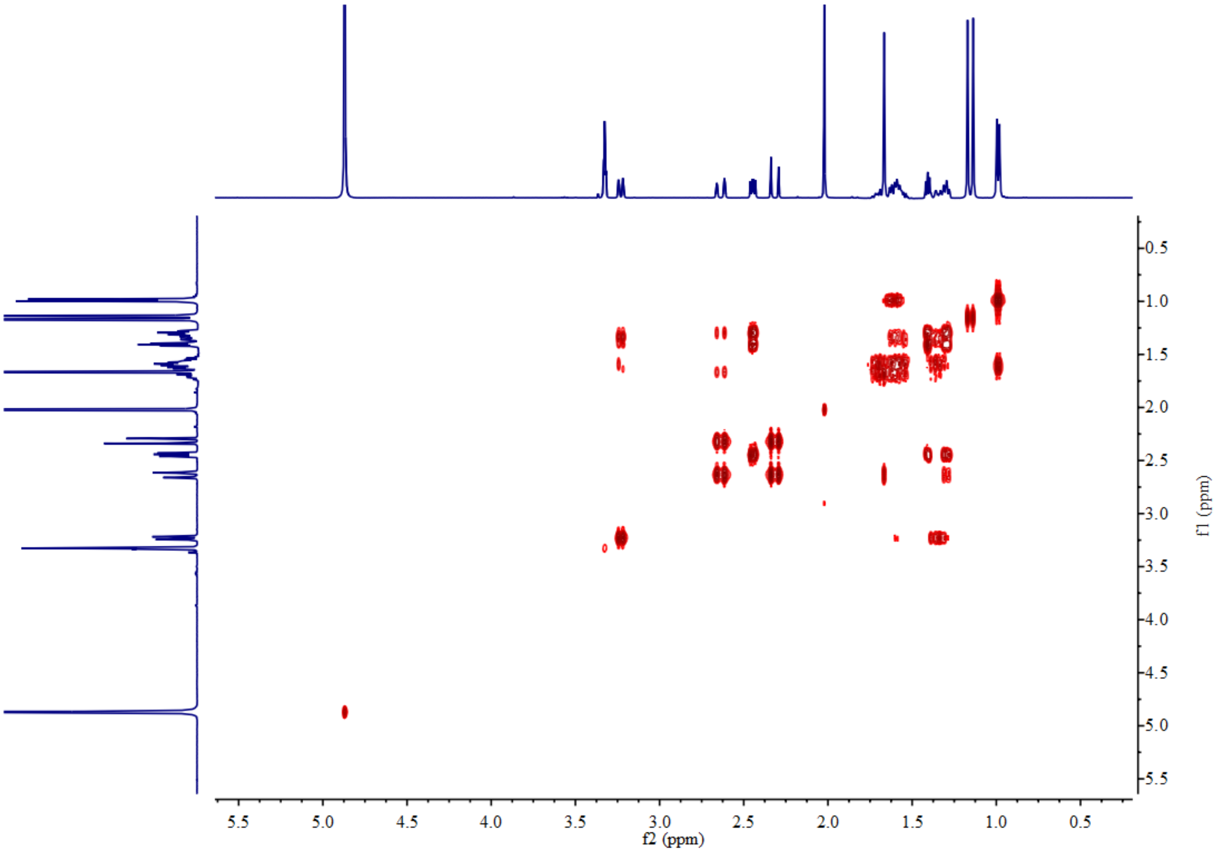

Figure S82 ^{1}H-^{1}H COSY spectrum of **3a** in methanol-d_4 (400 MHz).

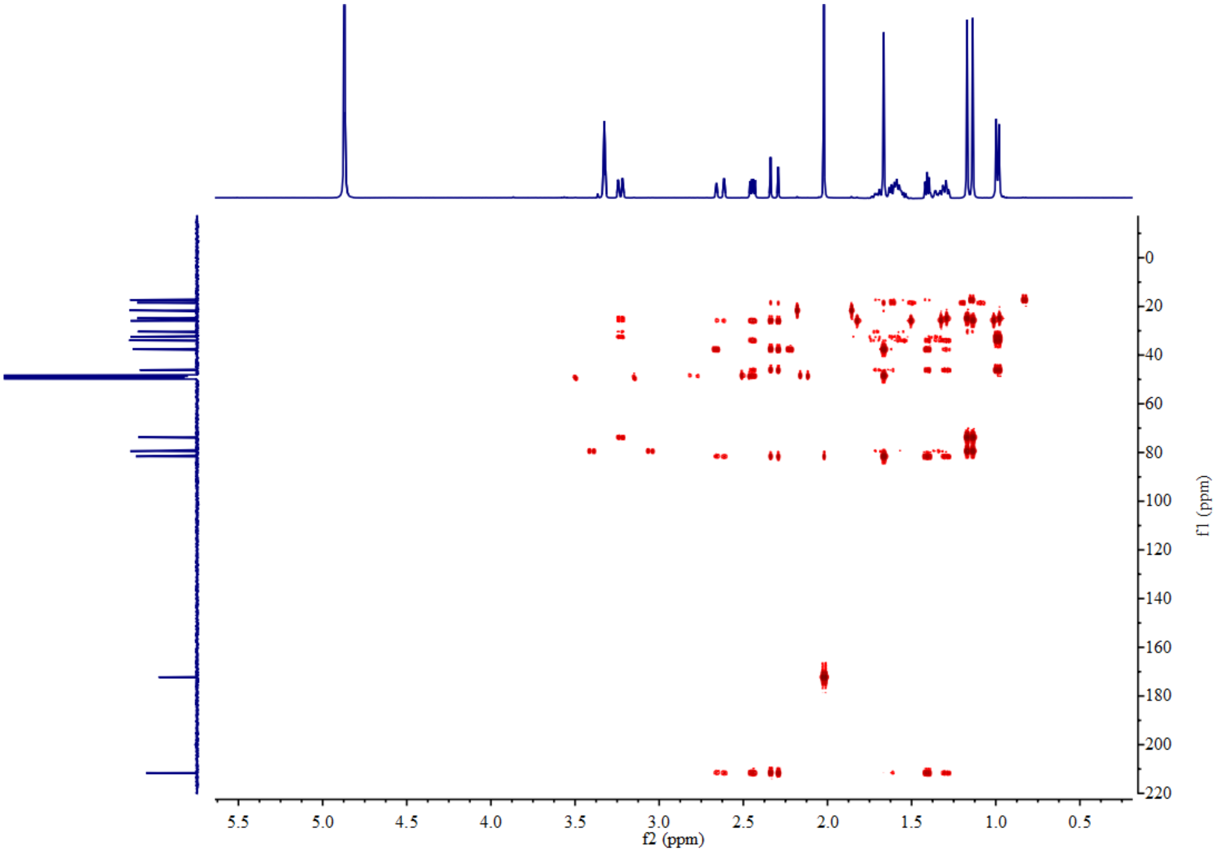

Figure S83 HMBC spectrum of **3a** in methanol-d_4 (400 MHz).

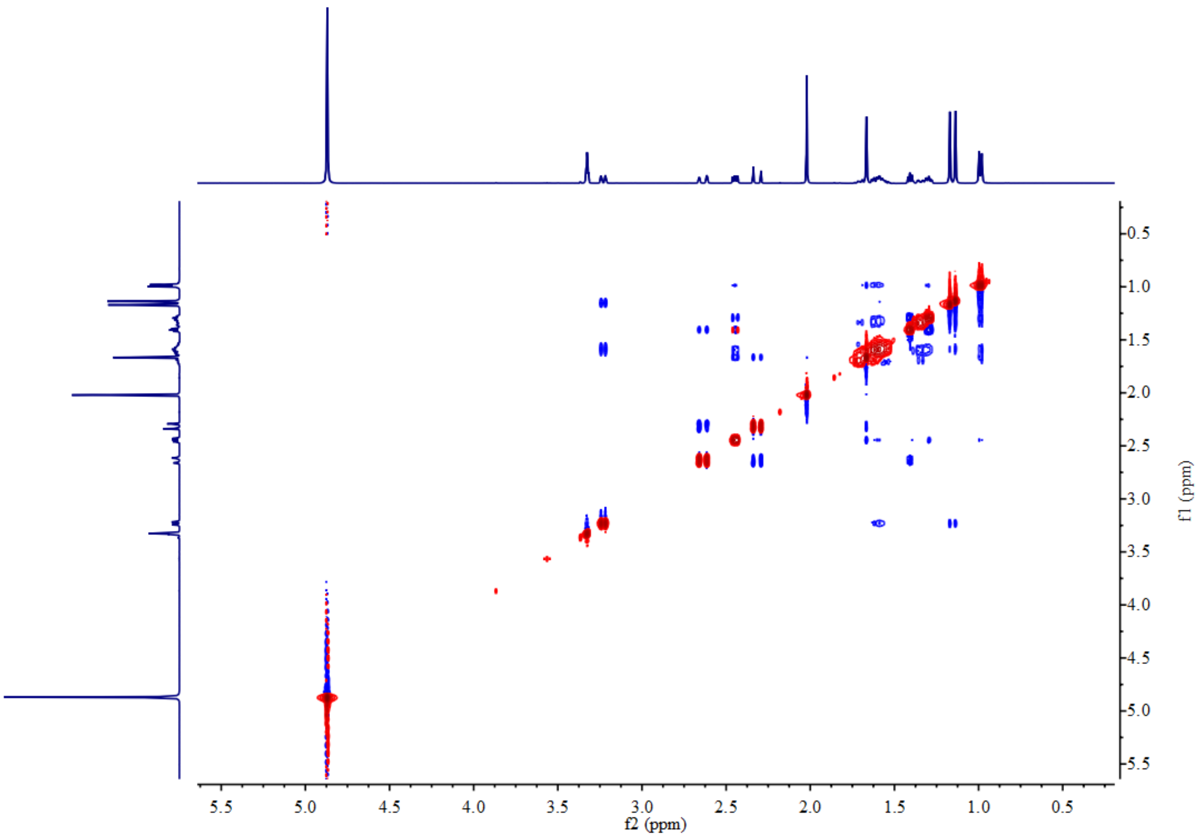

Figure S84 NOESY spectrum of **3a** in methanol-d_4 (400 MHz).

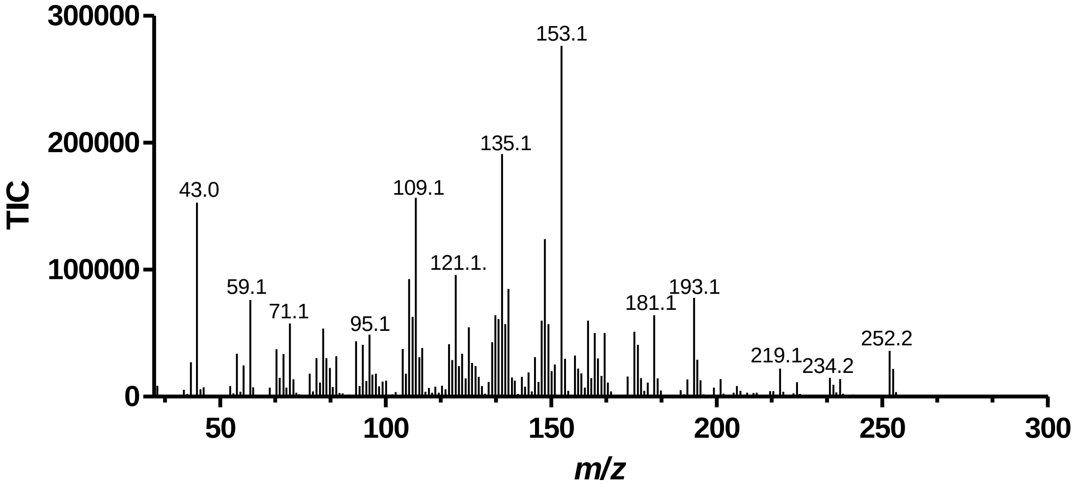

Figure S85 EI mass spectrum of **3a**.

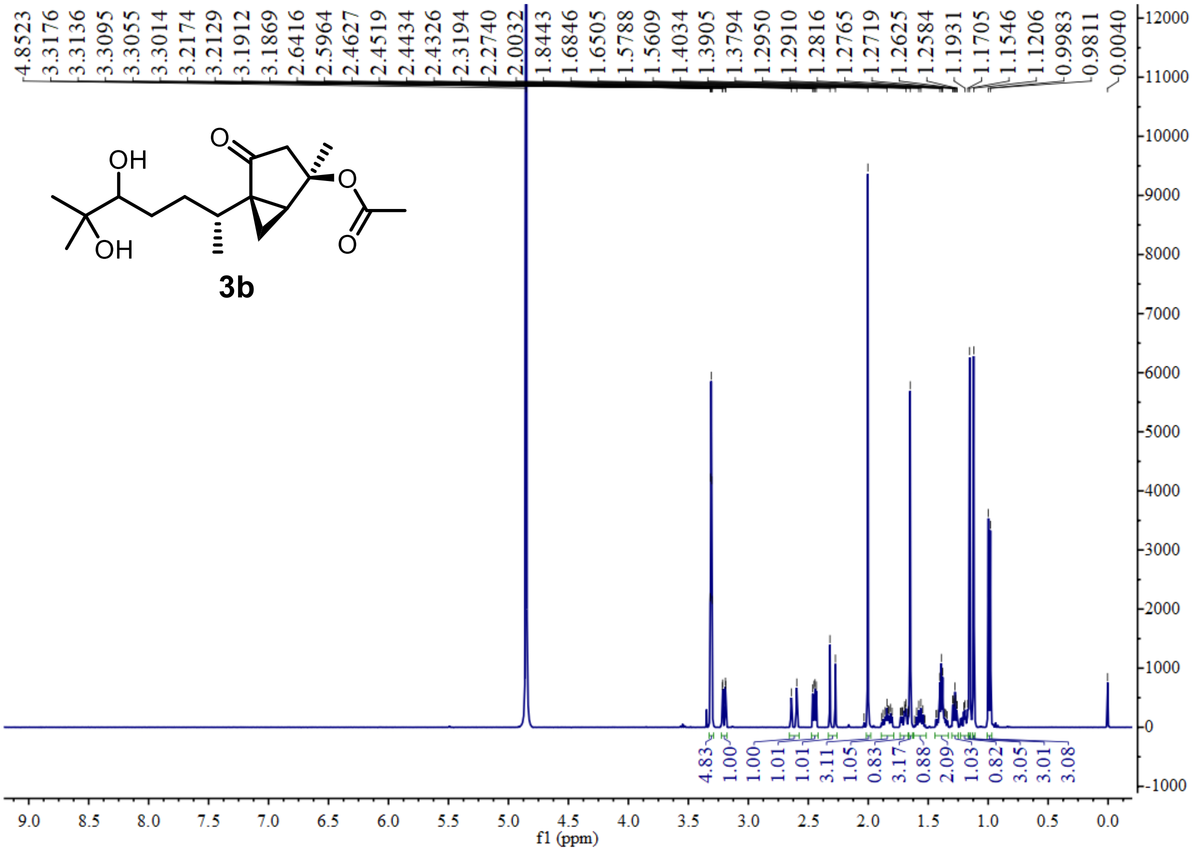

Figure S86 ^{1}H NMR spectrum of **3b** in methanol-d_4 (400 MHz).

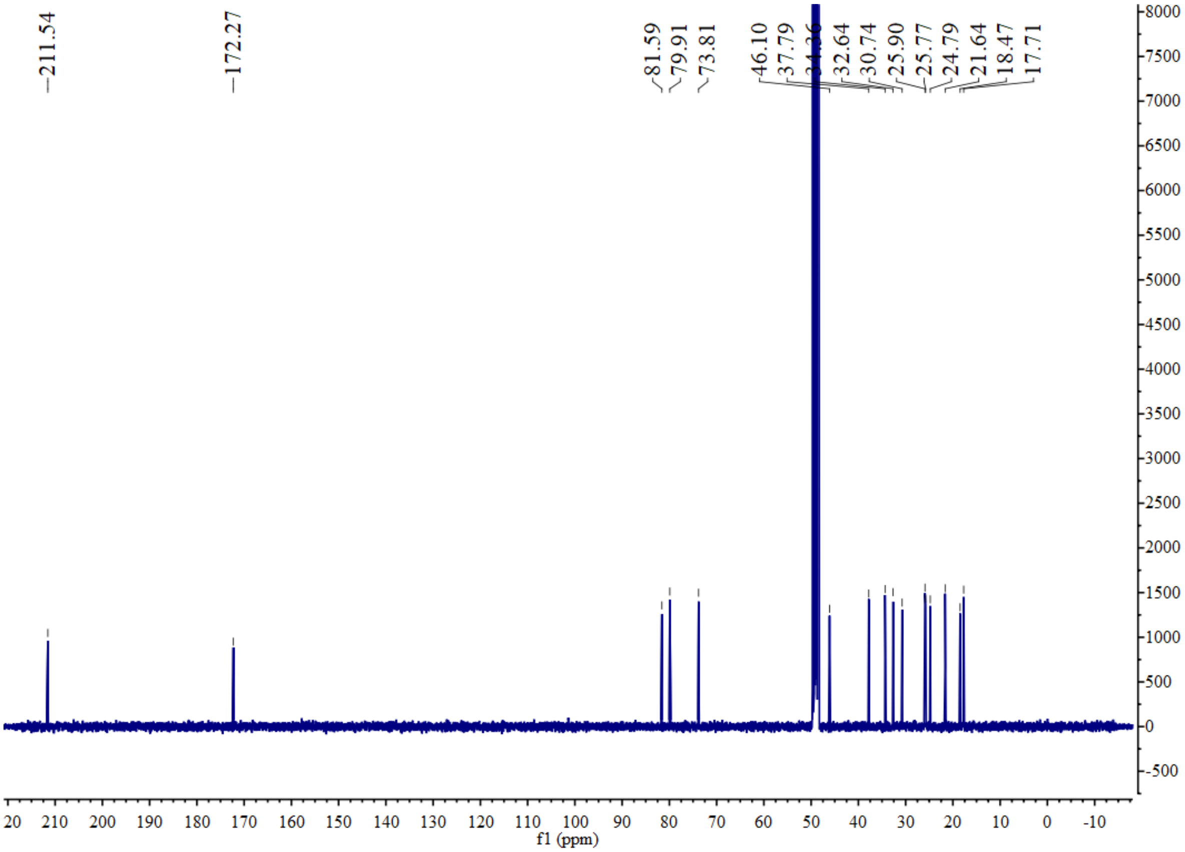

Figure S87 ^{13}C NMR spectrum of **3b** in methanol-d_4 (100 MHz).

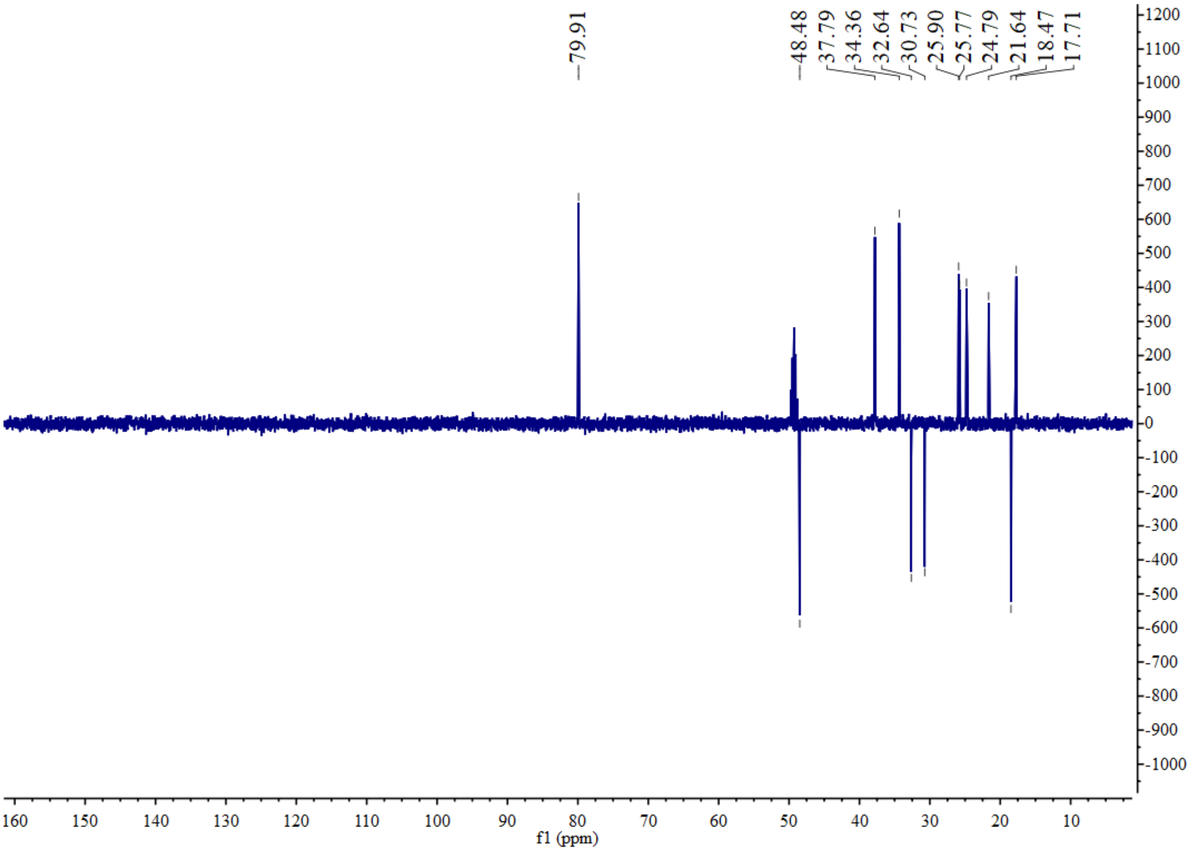

Figure S88 DEPT-135 NMR spectrum of **3b** in methanol-d_4 (100 MHz).

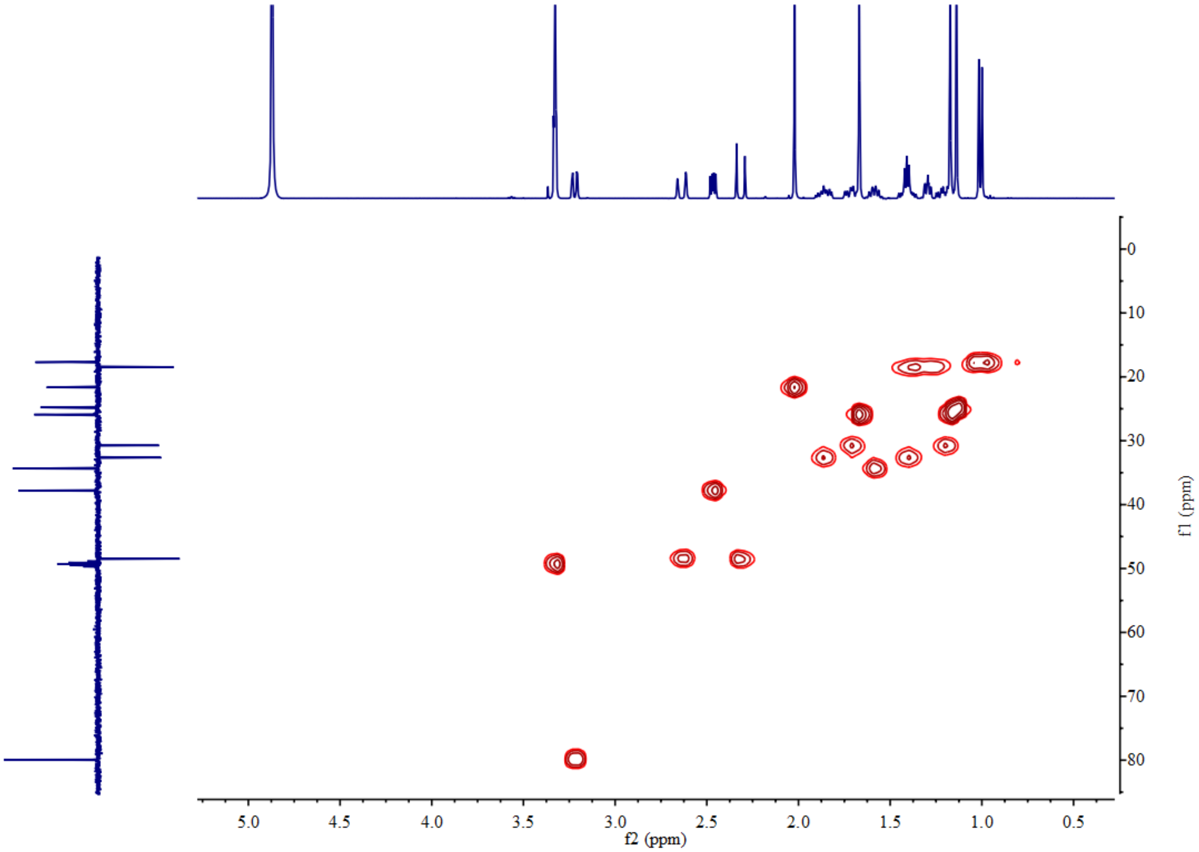

Figure S89 HSQC spectrum of **3b** in methanol-d_4 (400 MHz).

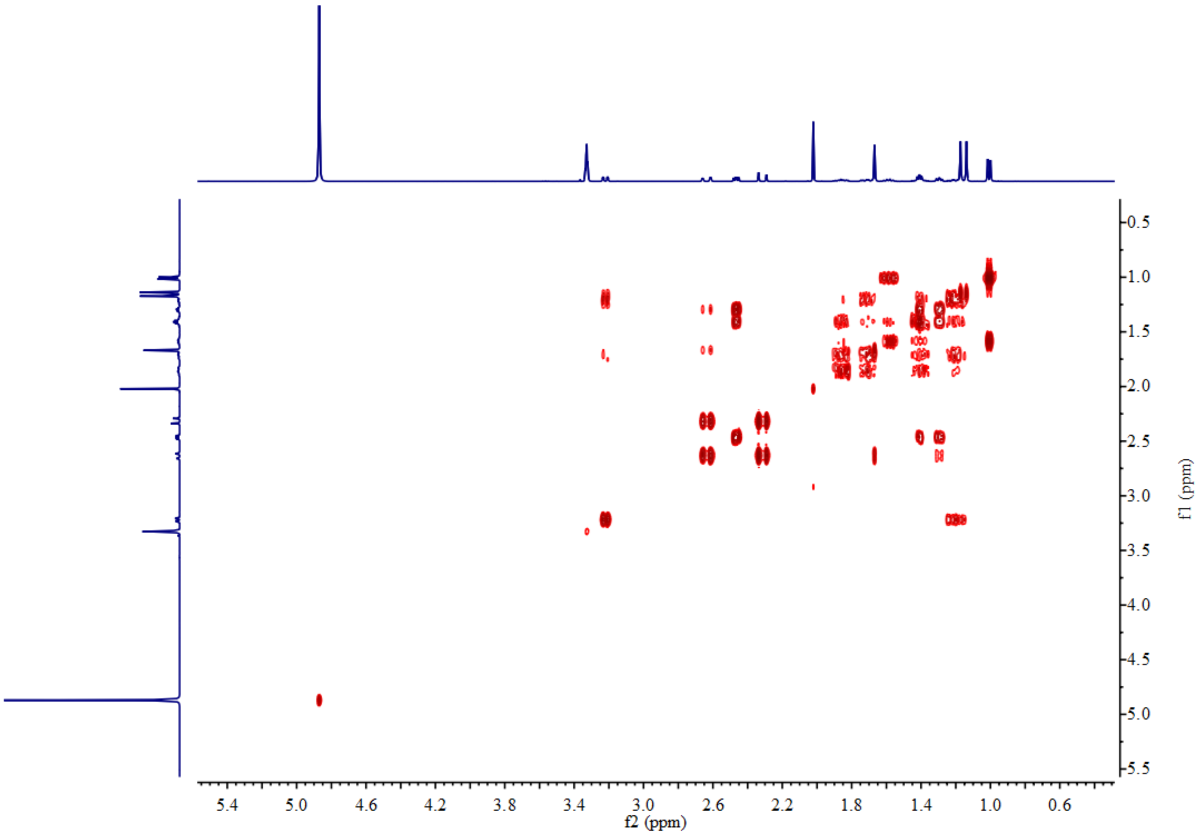

Figure S90 ^{1}H-^{1}H COSY spectrum of **3b** in methanol-d_4 (400 MHz).

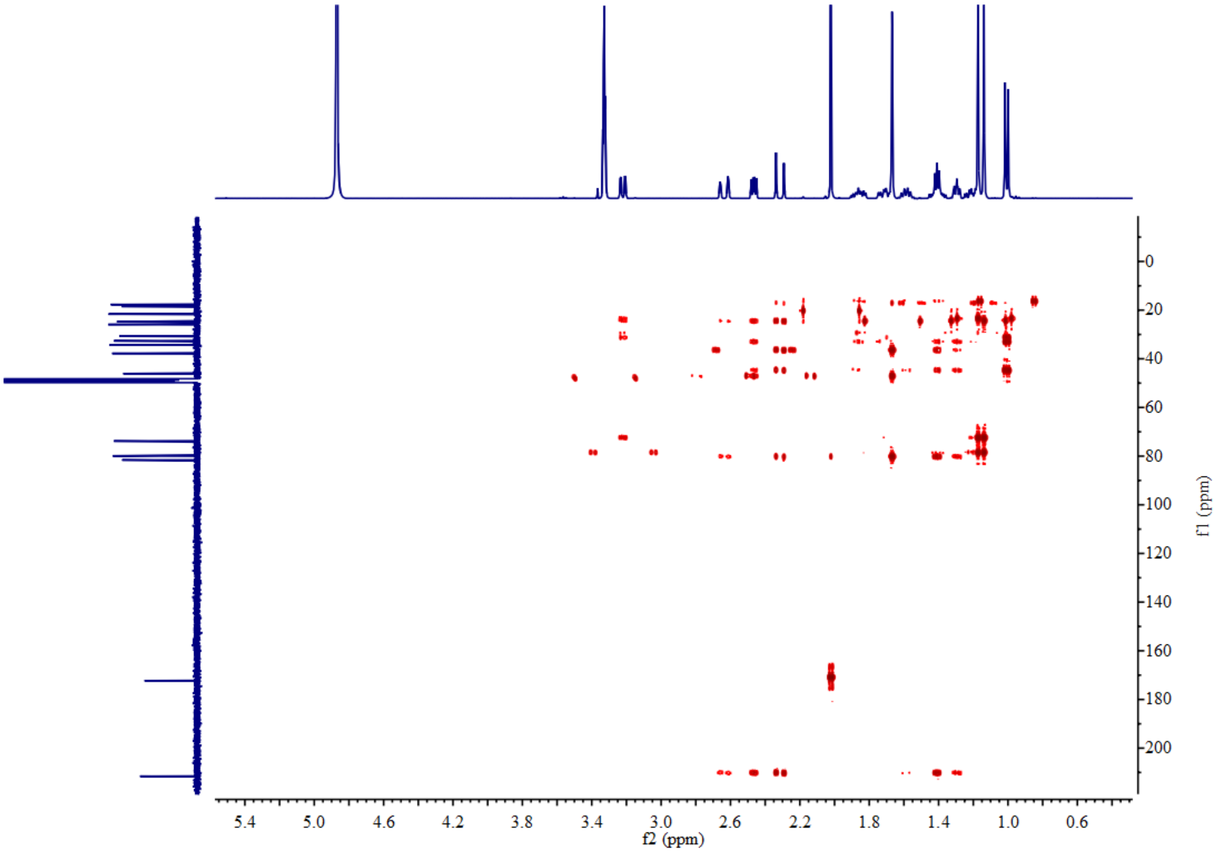

Figure S91 HMBC spectrum of **3b** in methanol-d_4 (400 MHz).

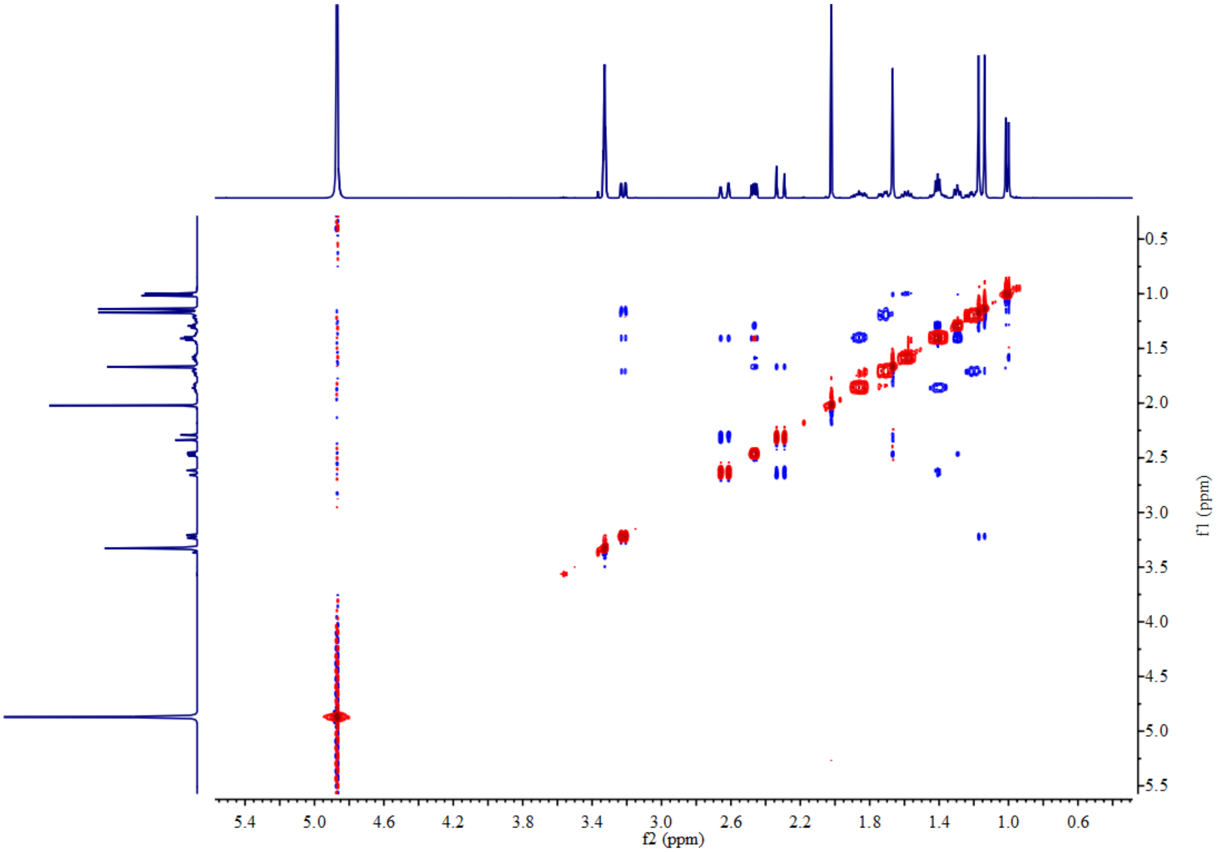

Figure S92 NOESY spectrum of **3b** in methanol-d_4 (400 MHz).

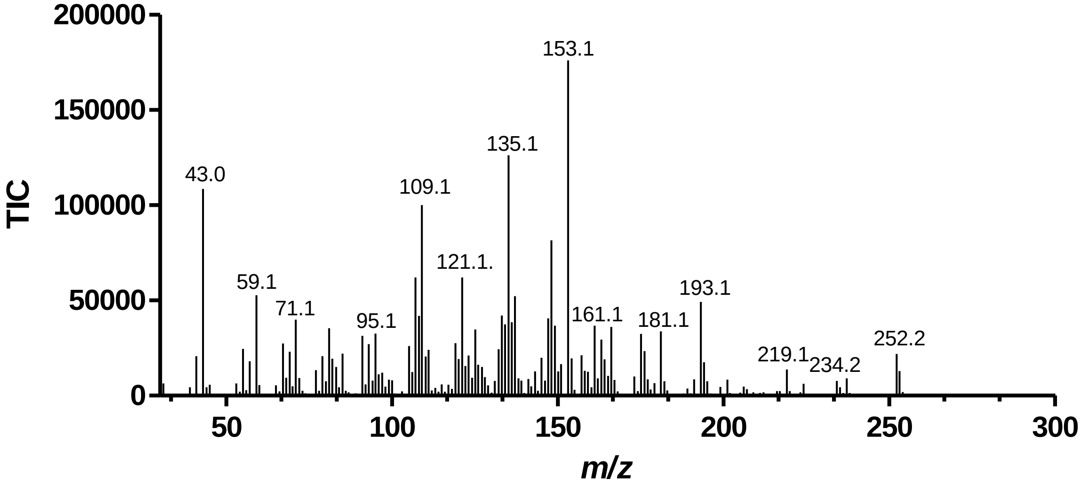

Figure S93 EI mass spectrum of **3b**.

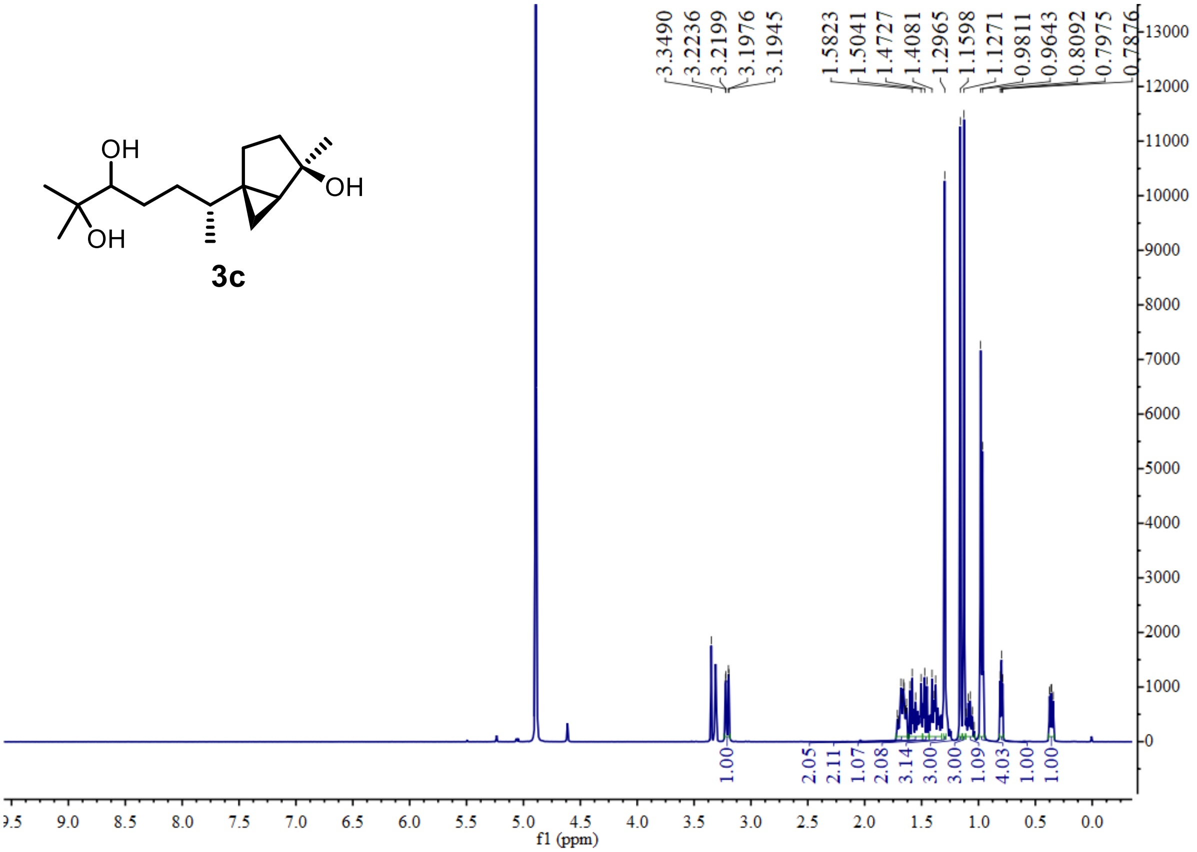

Figure S94 ^{1}H NMR spectrum of **3c** in methanol-d_4 (400 MHz).

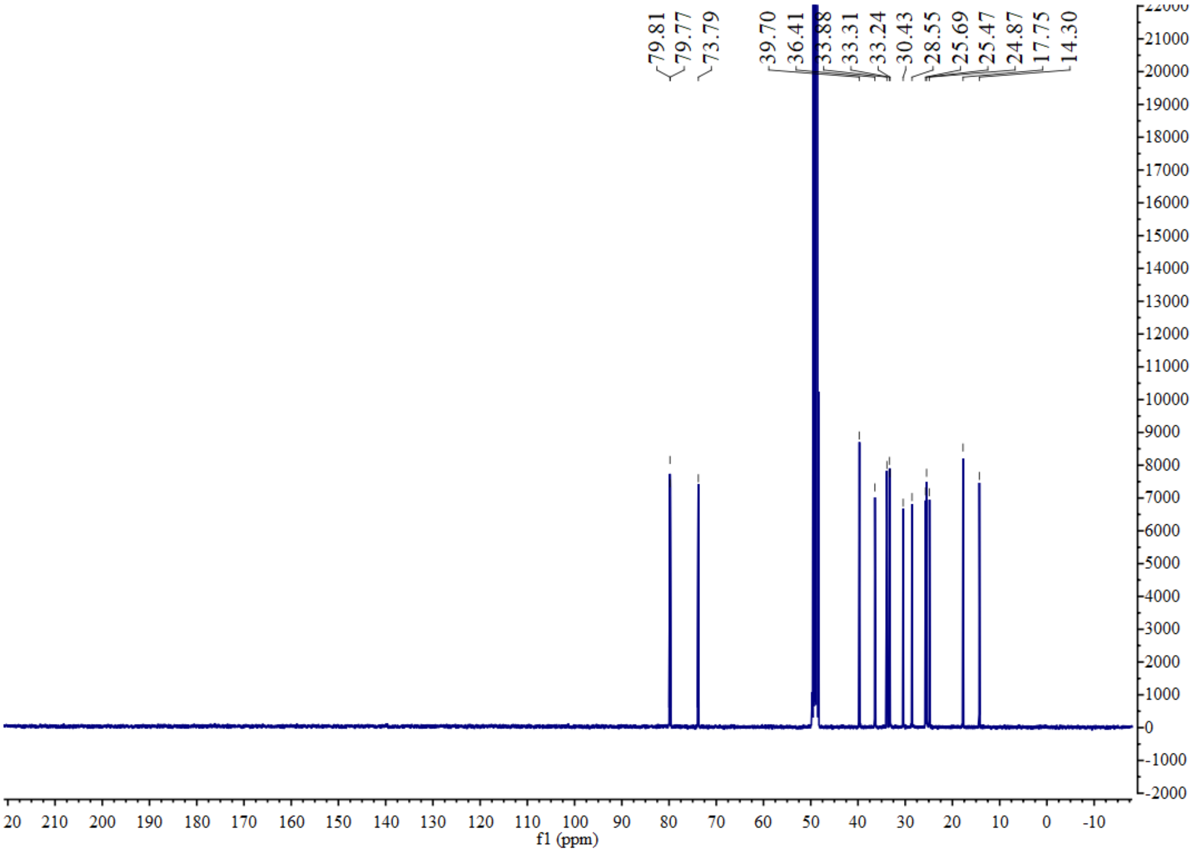

Figure S95 ^{13}C NMR spectrum of **3c** in methanol-d_4 (100 MHz).

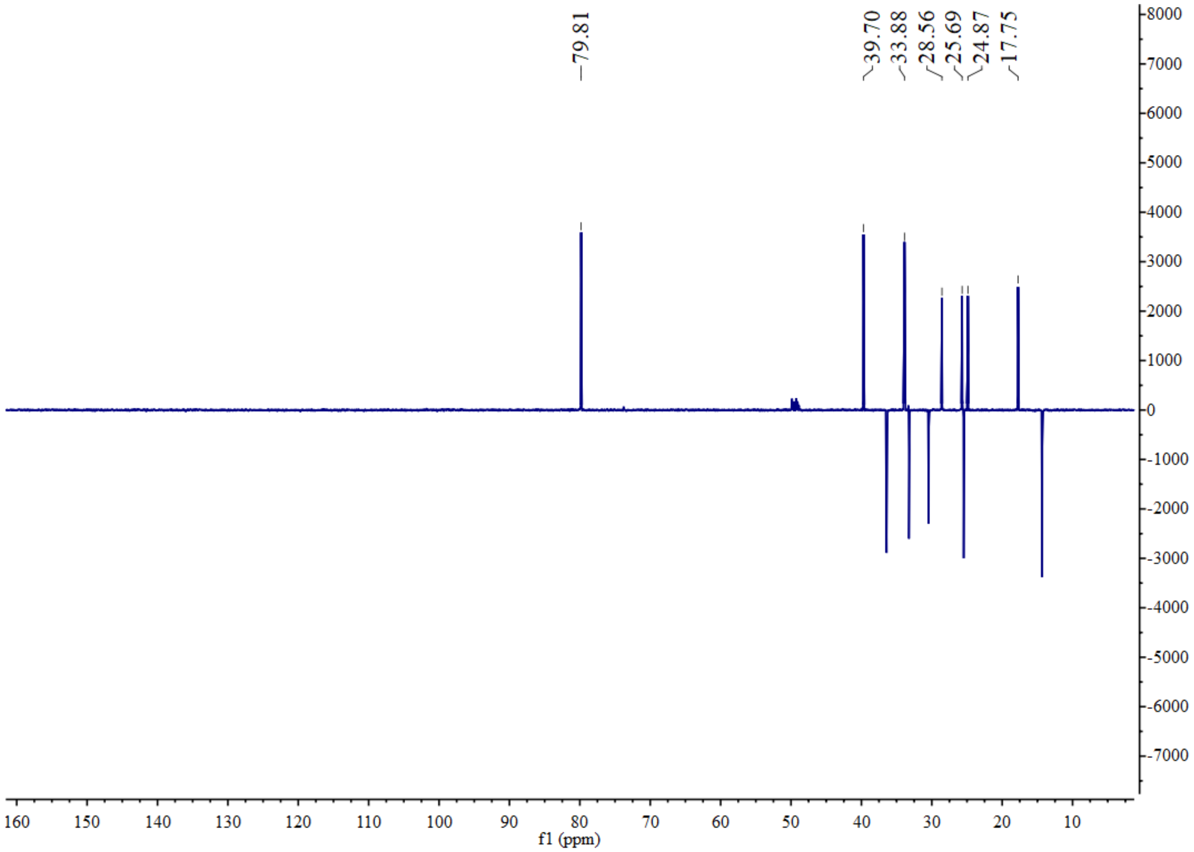

Figure S96 DEPT-135 NMR spectrum of **3c** in methanol-d_4 (100 MHz).

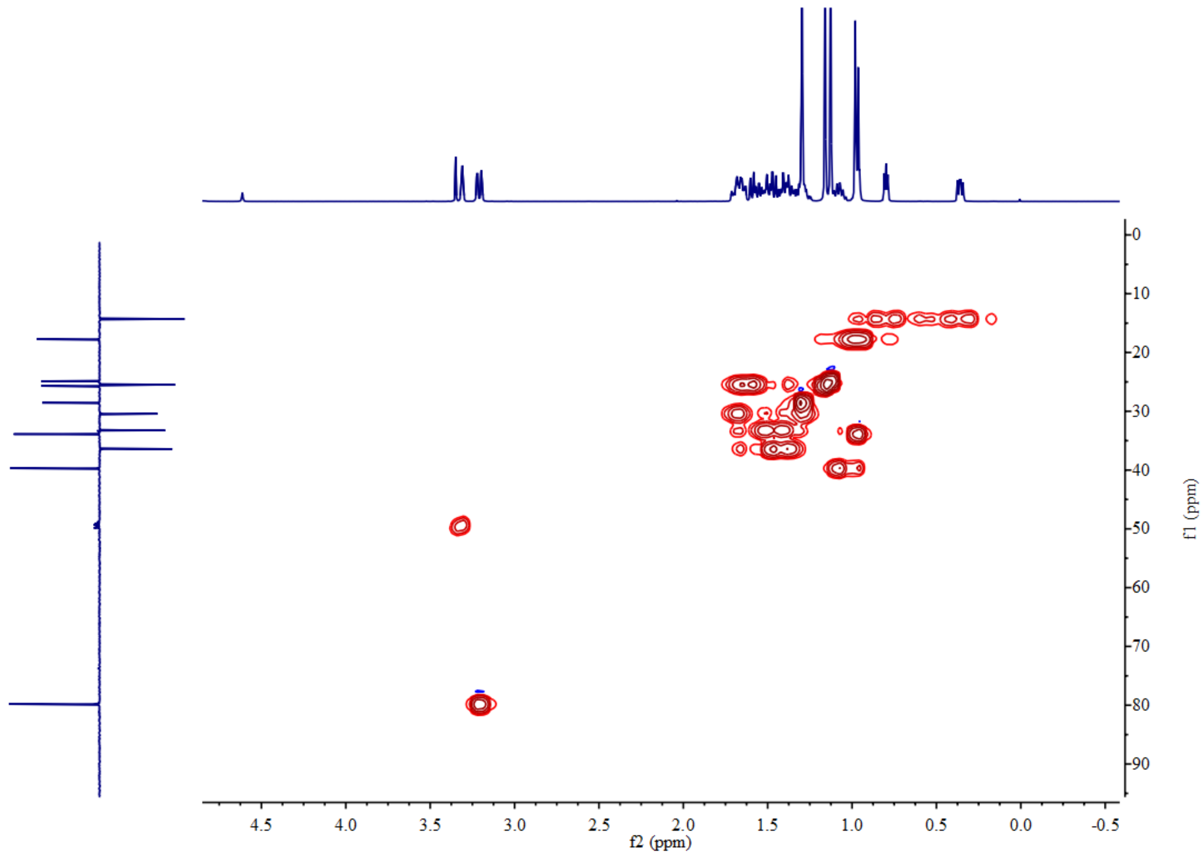

Figure S97 HSQC spectrum of **3c** in methanol-d_4 (400 MHz).

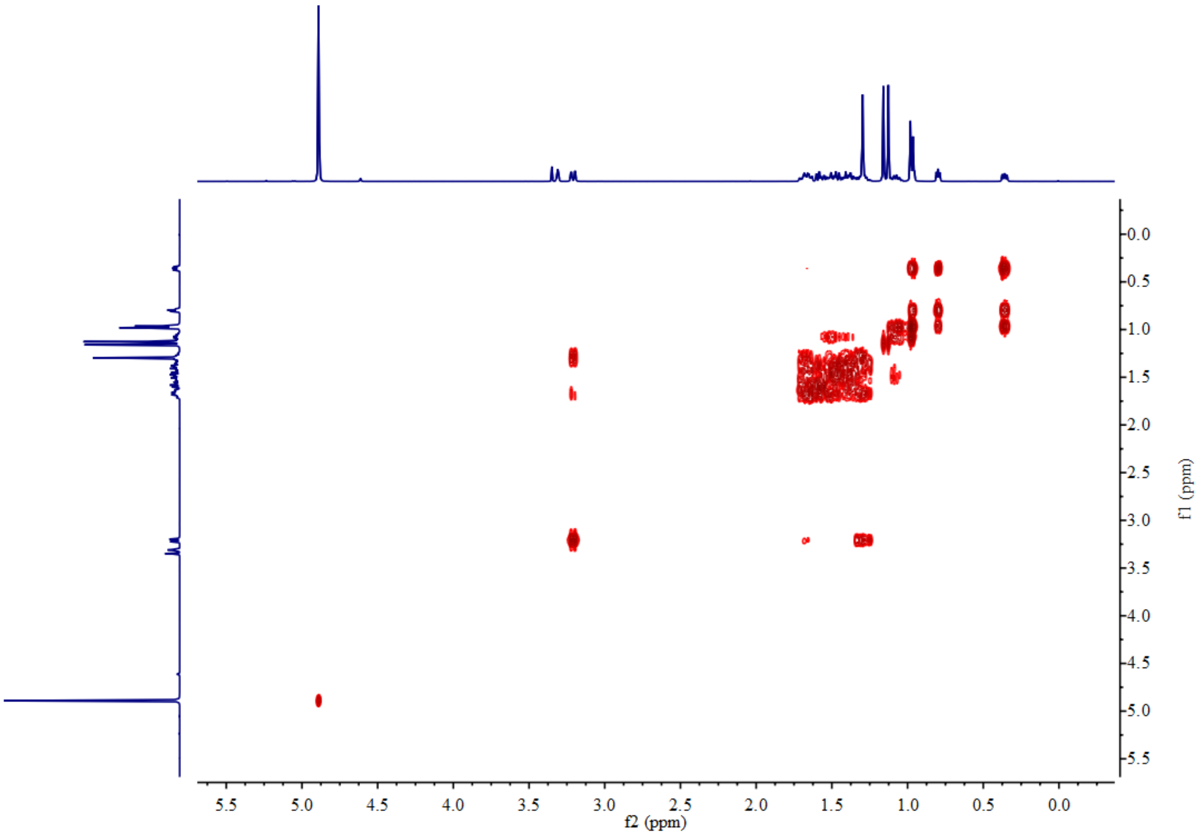

Figure S98 ^{1}H-^{1}H COSY spectrum of **3c** in methanol-d_4 (400 MHz).

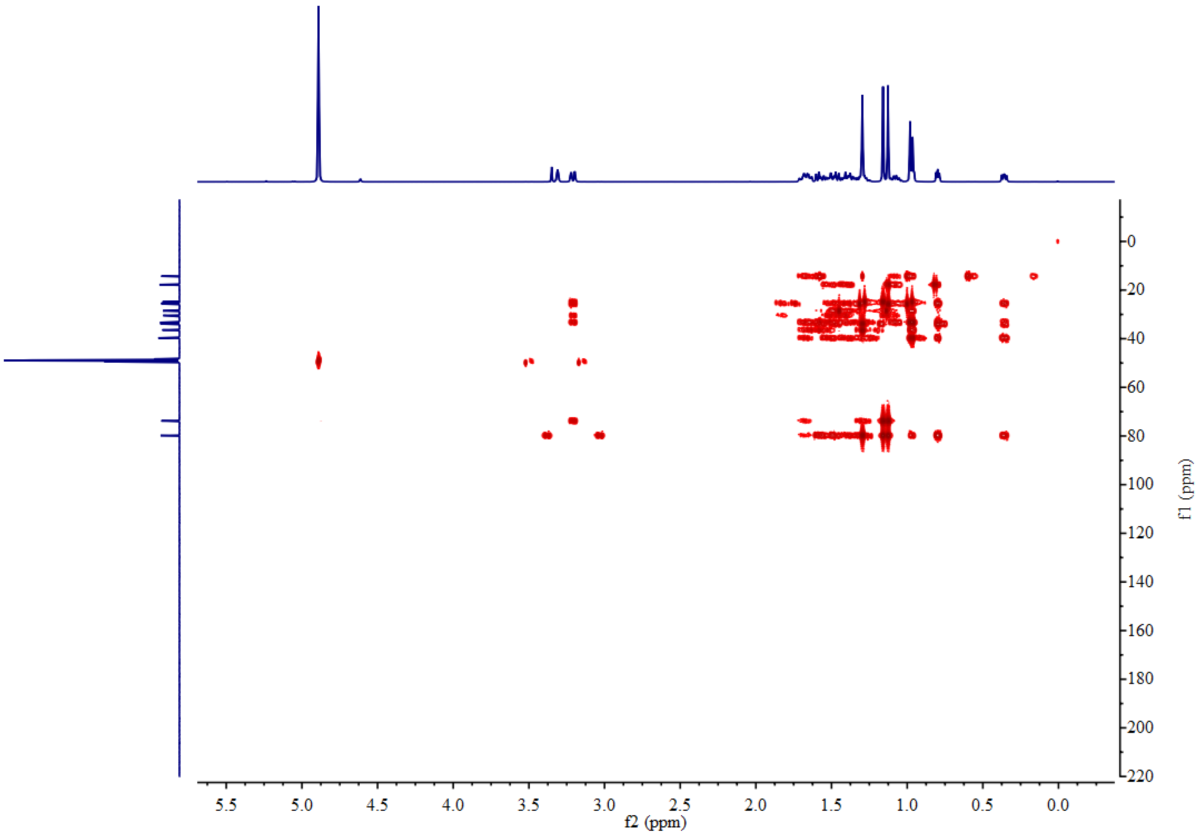

Figure S99 HMBC spectrum of **3c** in methanol-d_4 (400 MHz).

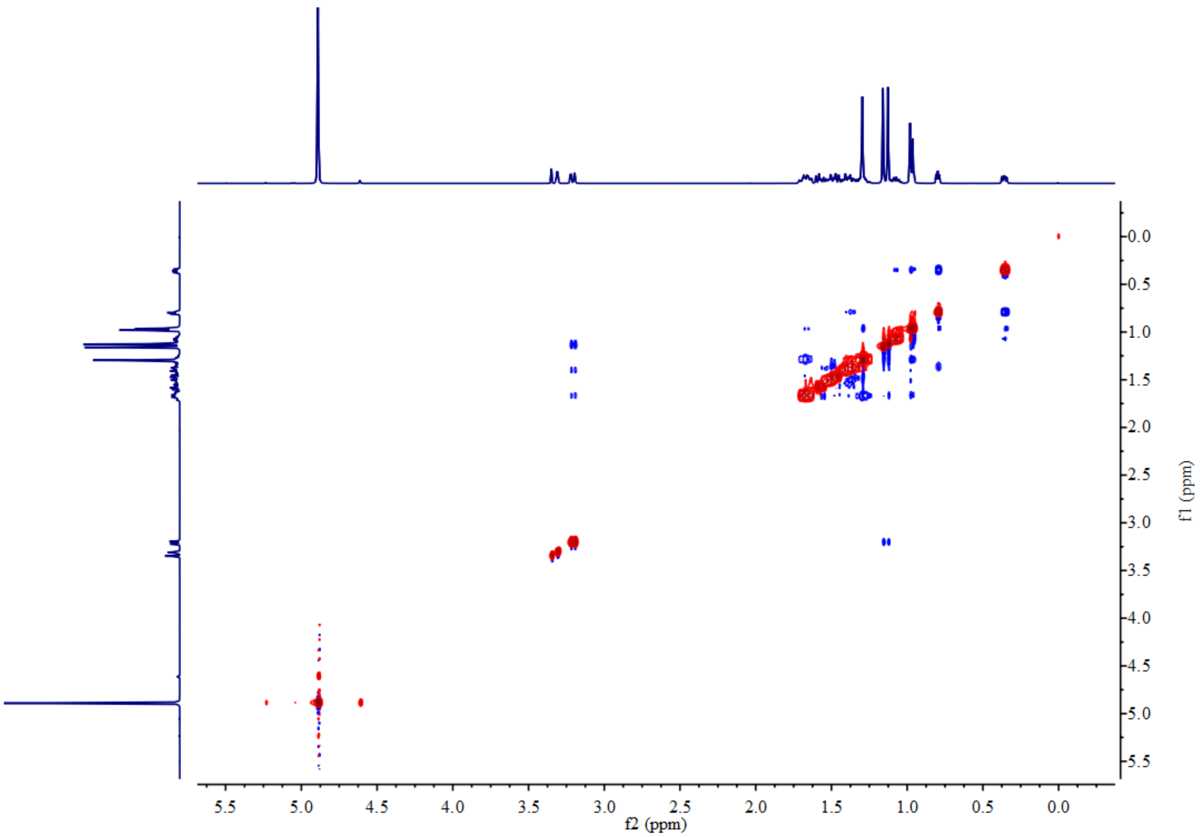

Figure S100 NOESY spectrum of **3c** in methanol-d_4 (400 MHz).

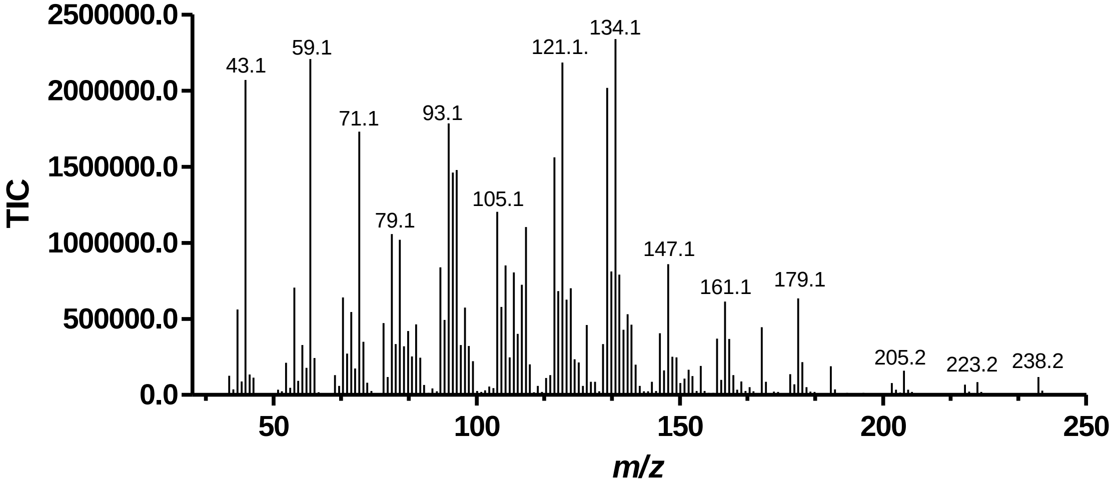

Figure S101 EI mass spectrum of **3c**.

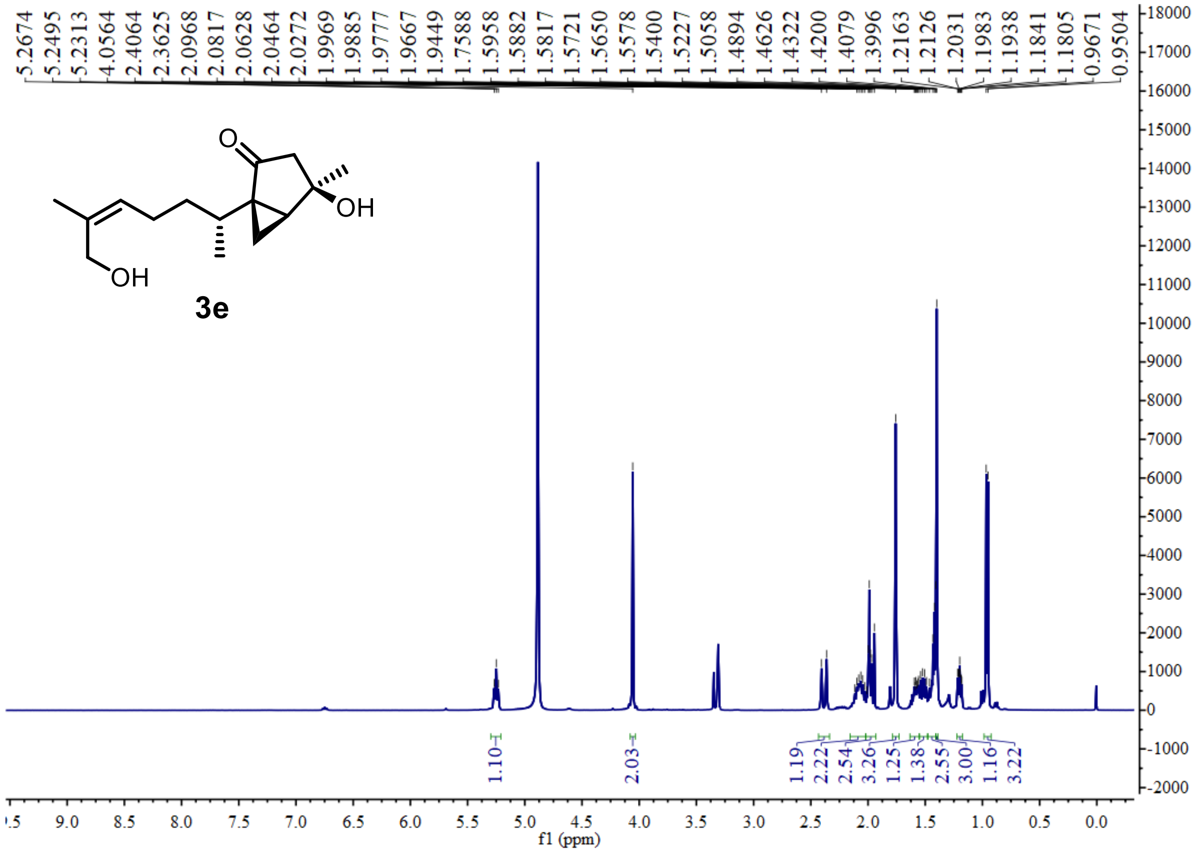

Figure S102 ^{1}H NMR spectrum of **3e** in methanol-d_4 (400 MHz).

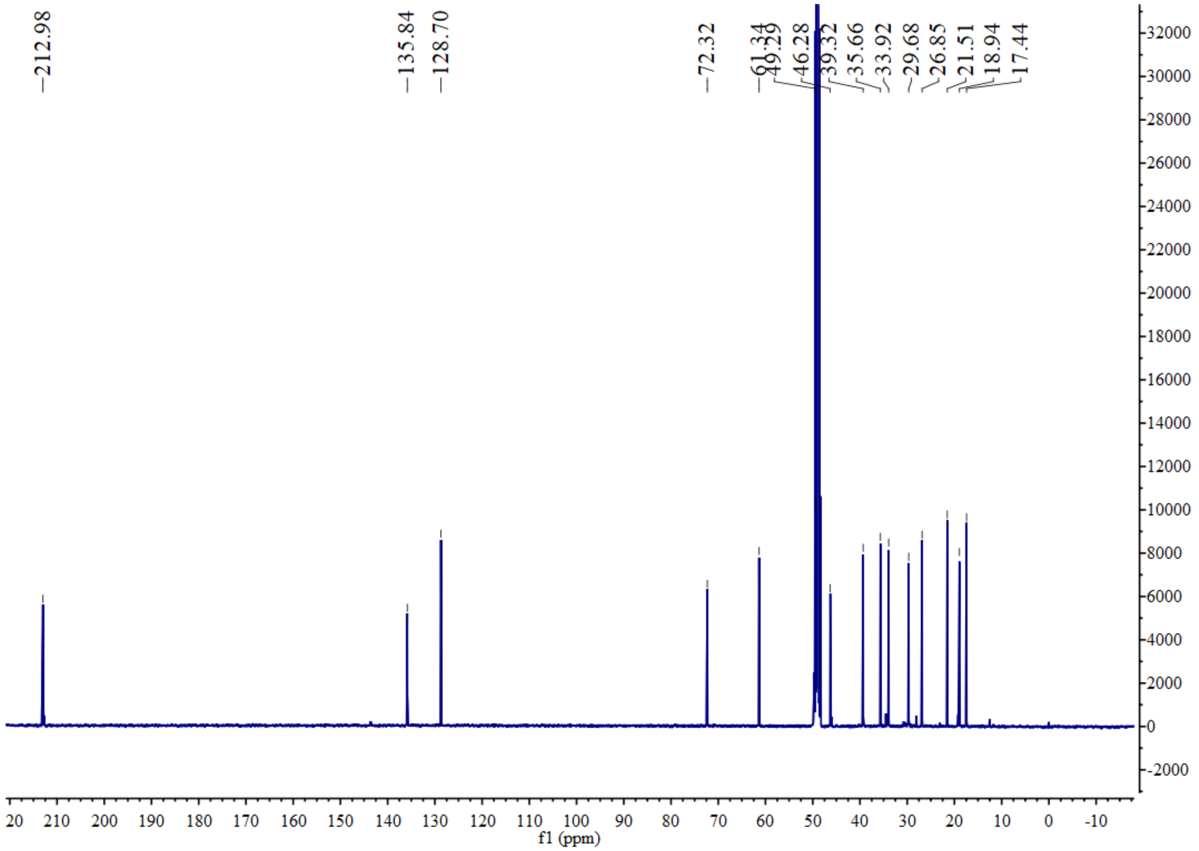

Figure S103 ^{13}C NMR spectrum of **3e** in methanol-d_4 (100 MHz).

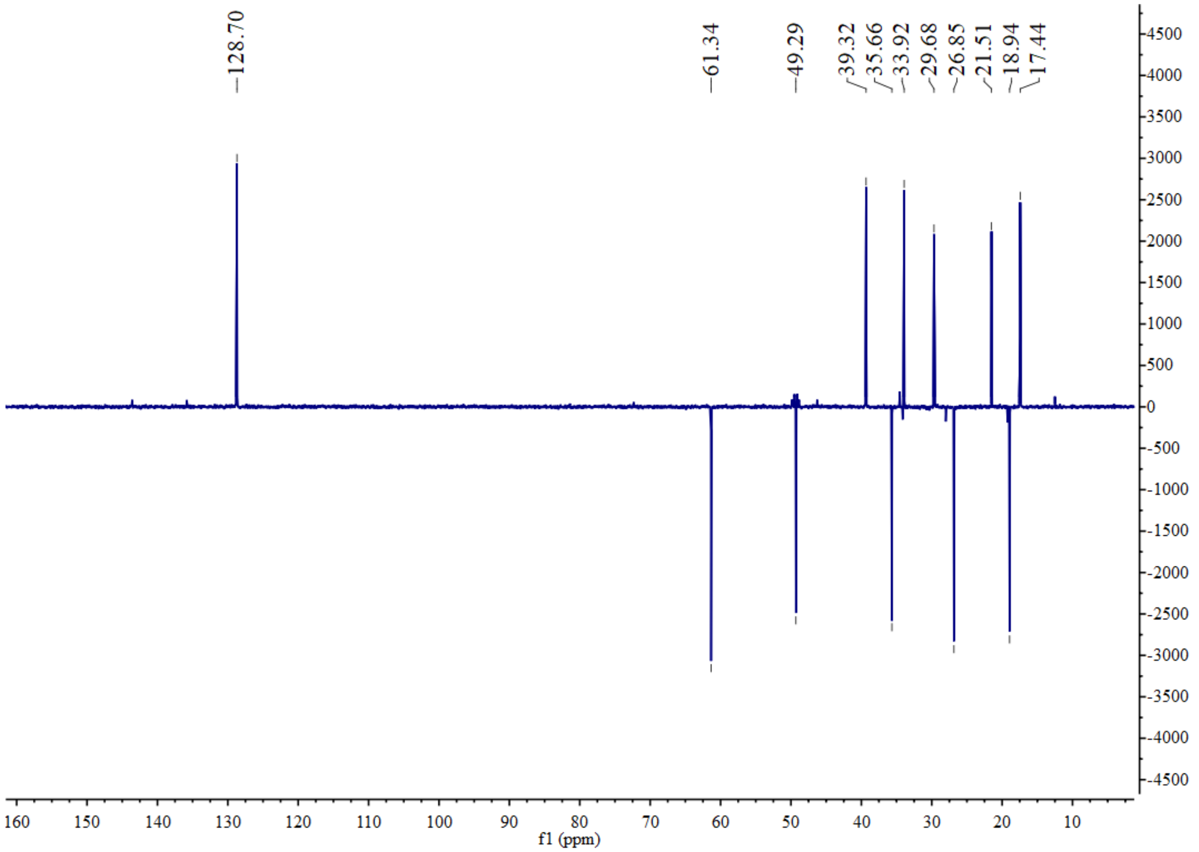

Figure S104 DEPT-135 NMR spectrum of **3e** in methanol-d_4 (100 MHz).

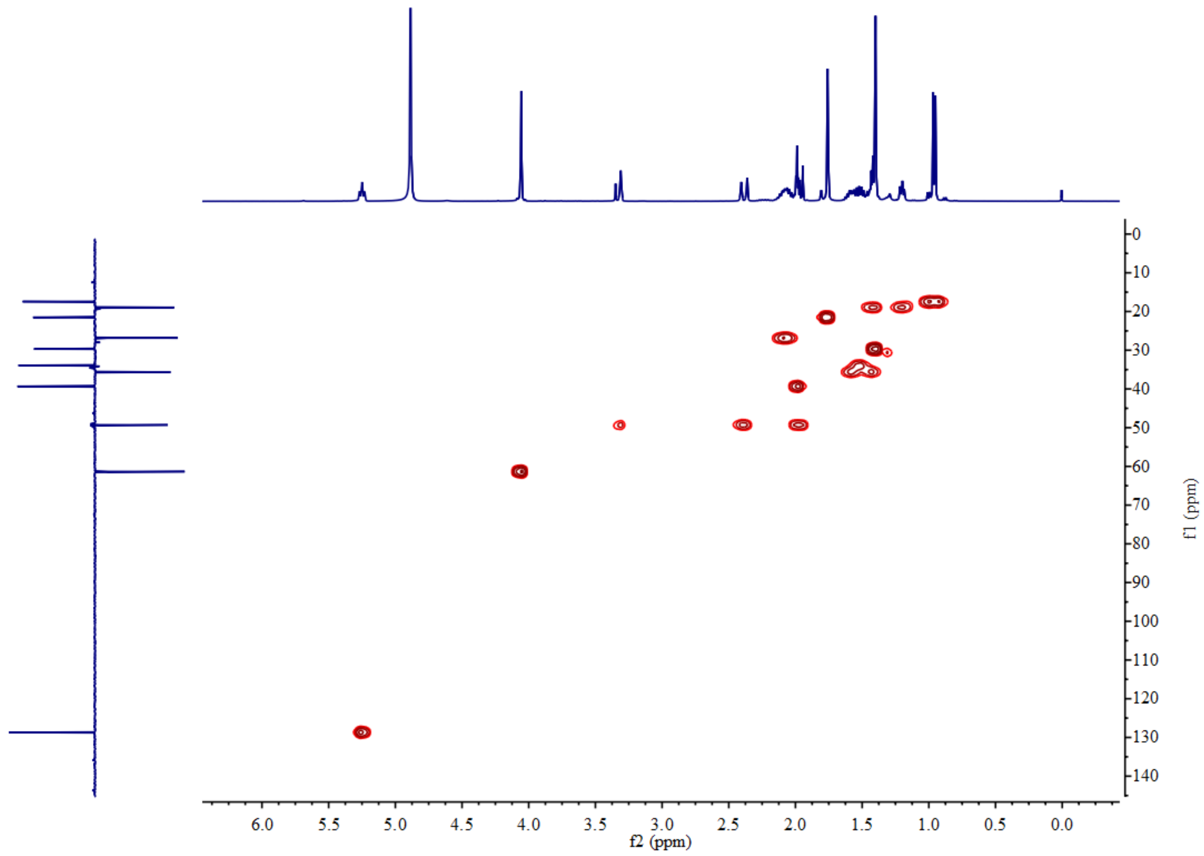

Figure S105 HSQC spectrum of **3e** in methanol-d_4 (400 MHz).

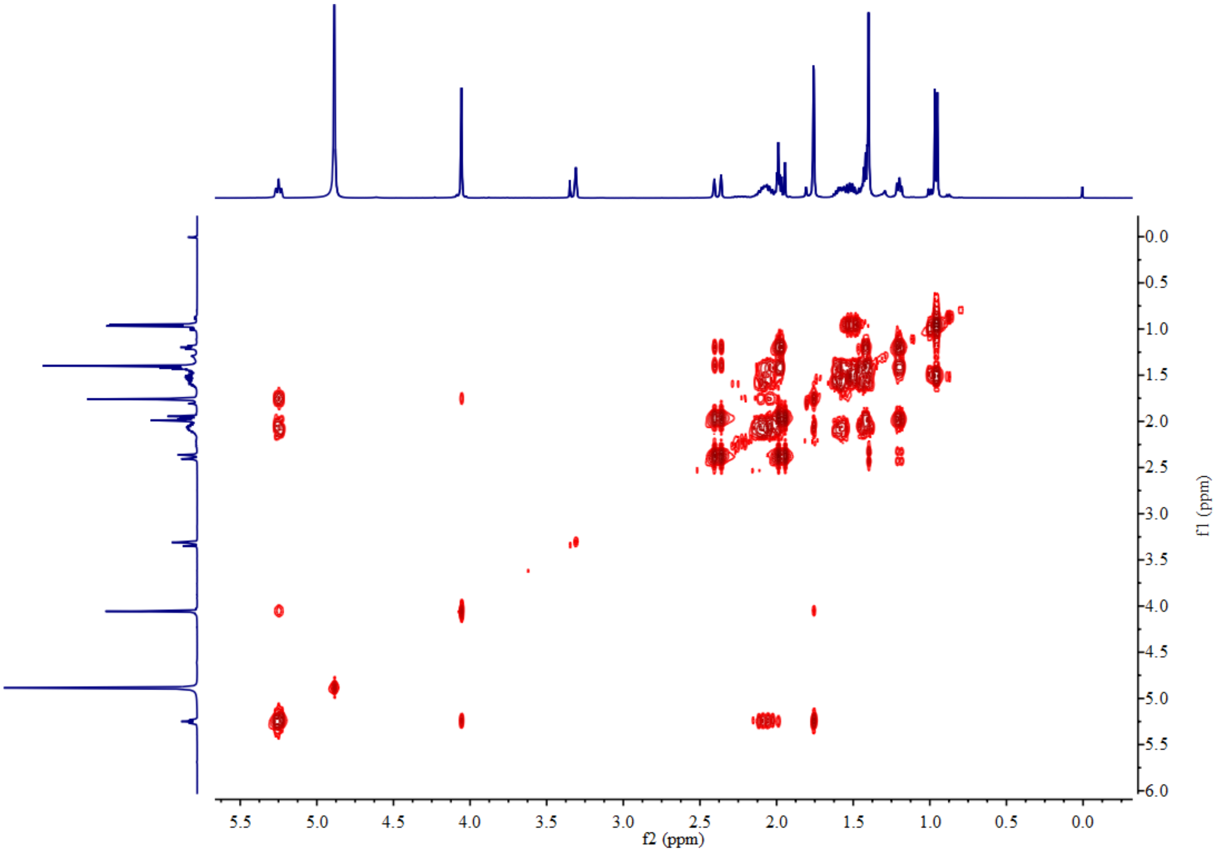

Figure S106 ^{1}H-^{1}H COSY spectrum of **3e** in methanol-d_4 (400 MHz).

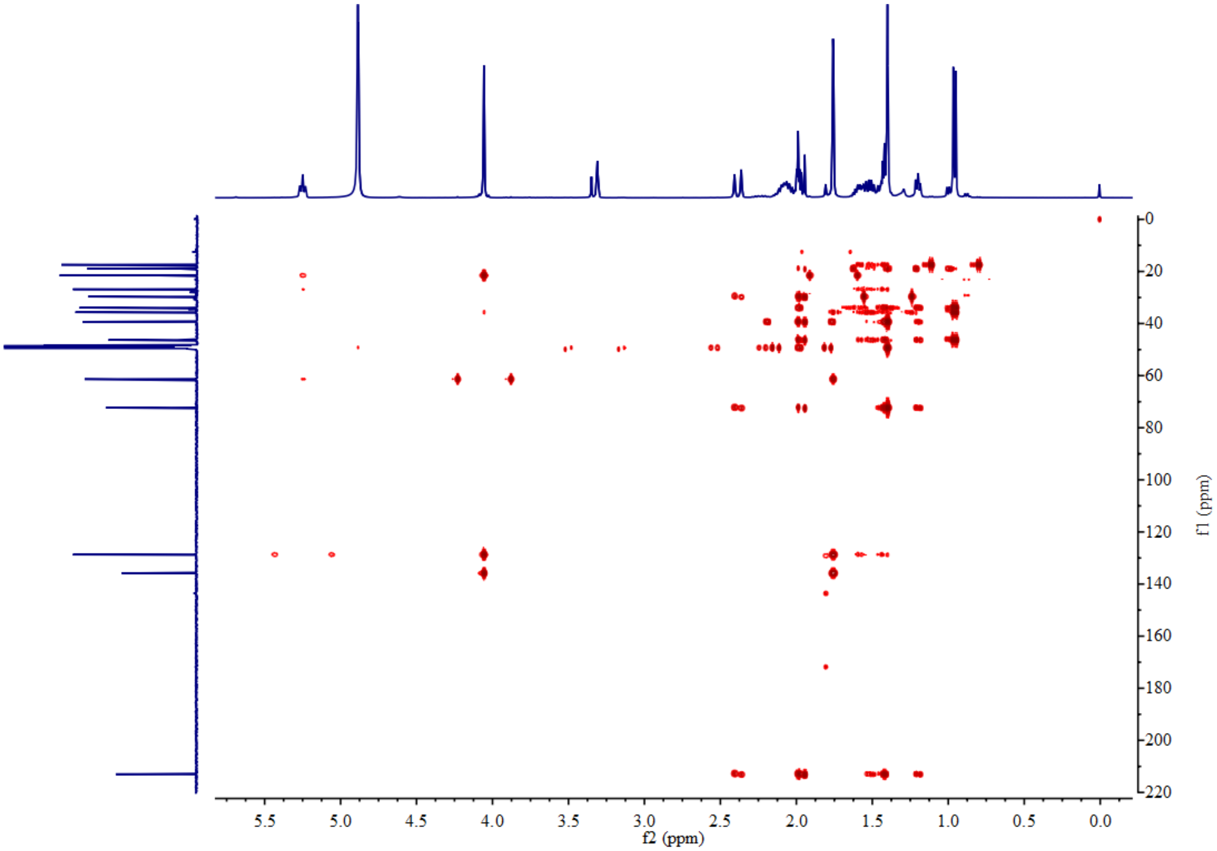

Figure S107 HMBC spectrum of **3e** in methanol-d_4 (400 MHz).

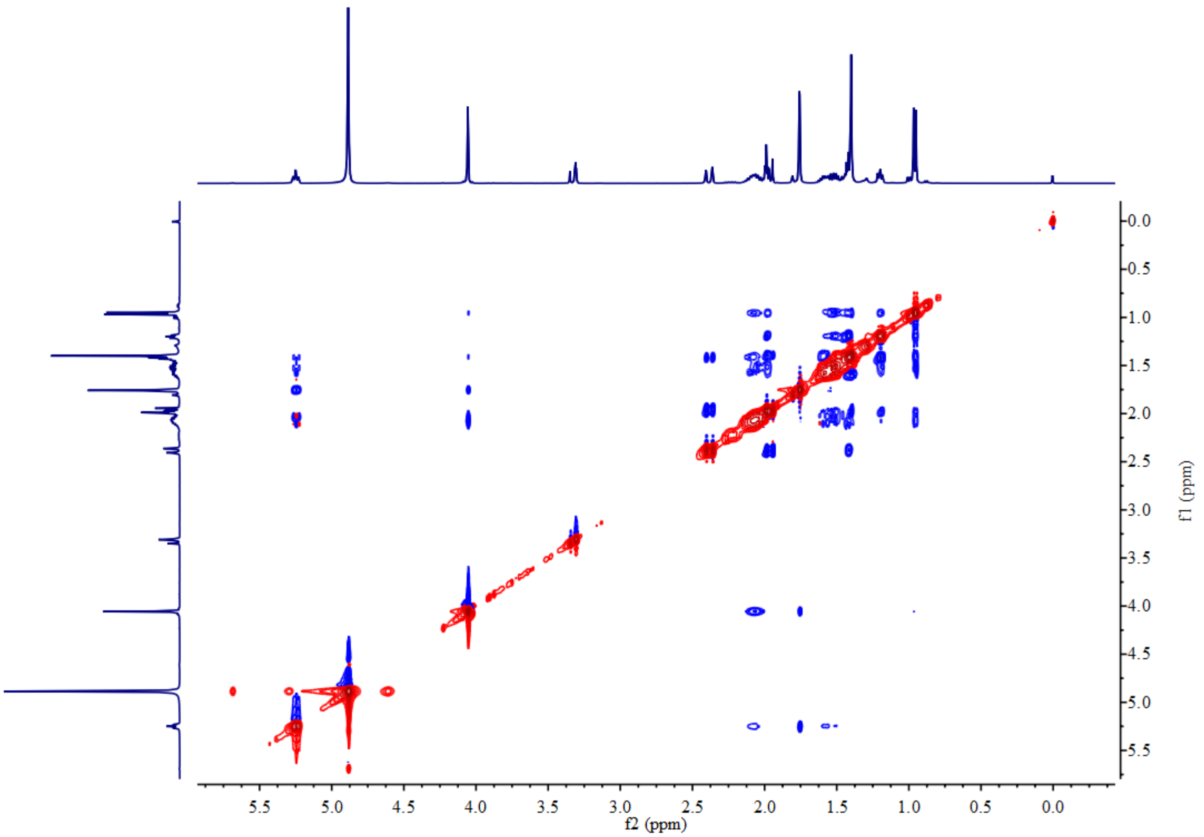

Figure S108 NOESY spectrum of **3e** in methanol-d_4 (400 MHz).

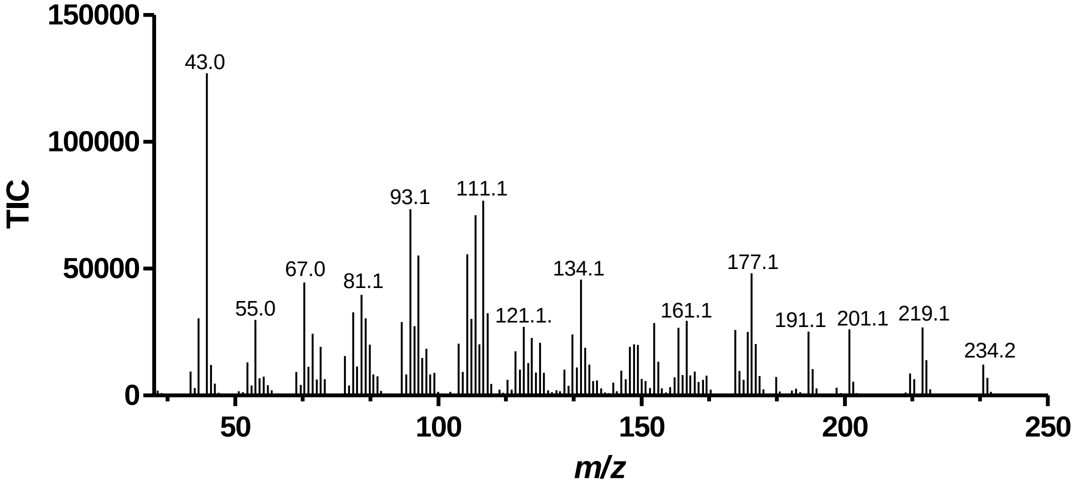

Figure S109 EI mass spectrum of **3e**.

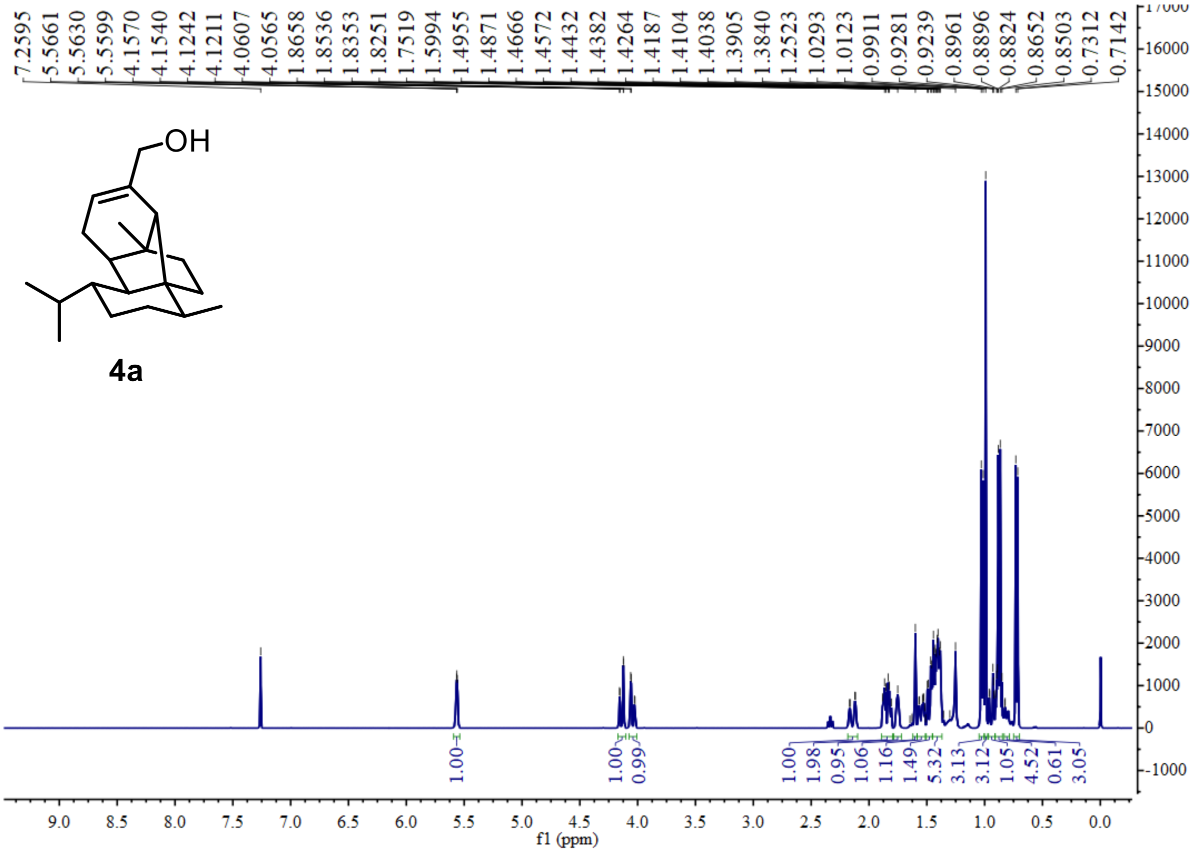

Figure S110 ^{1}H NMR spectrum of **4a** in $CDCl_3$ (400 MHz).

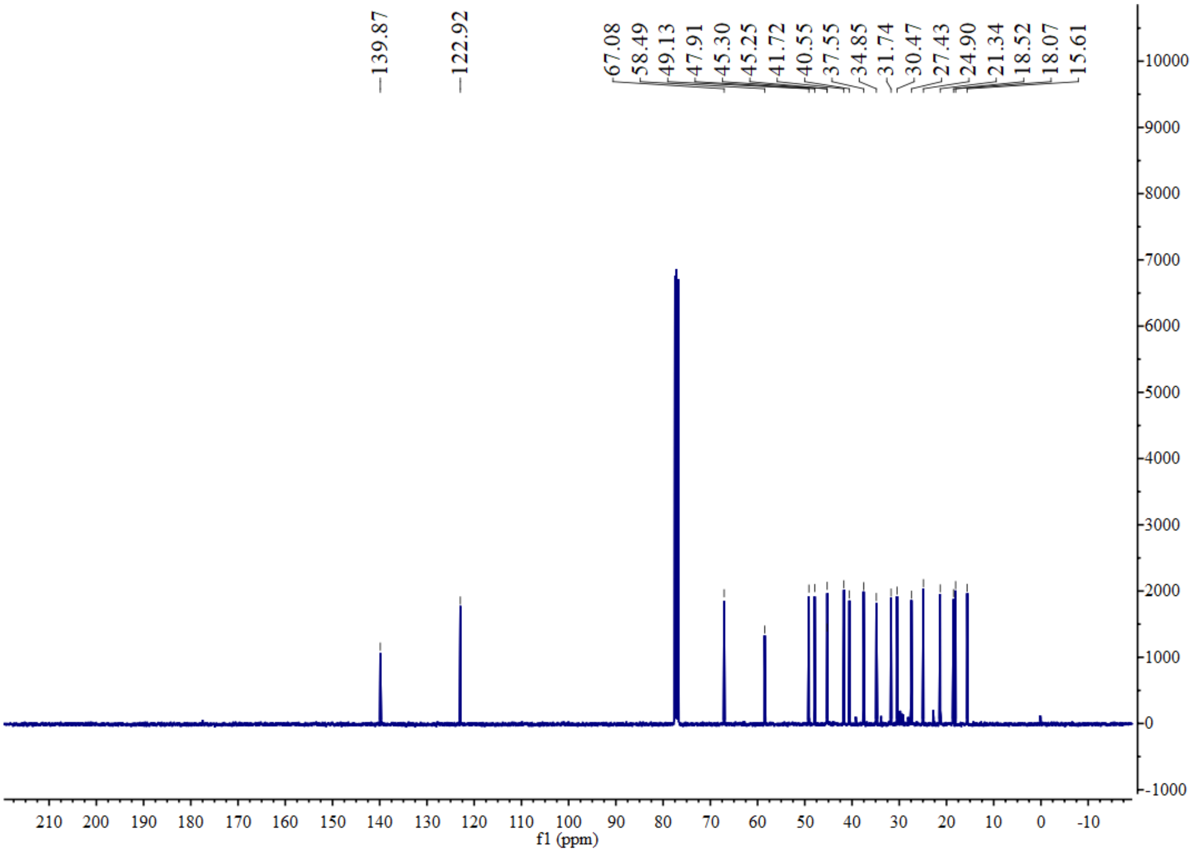

Figure S111 ^{13}C NMR spectrum of **4a** in $CDCl_3$ (100 MHz).

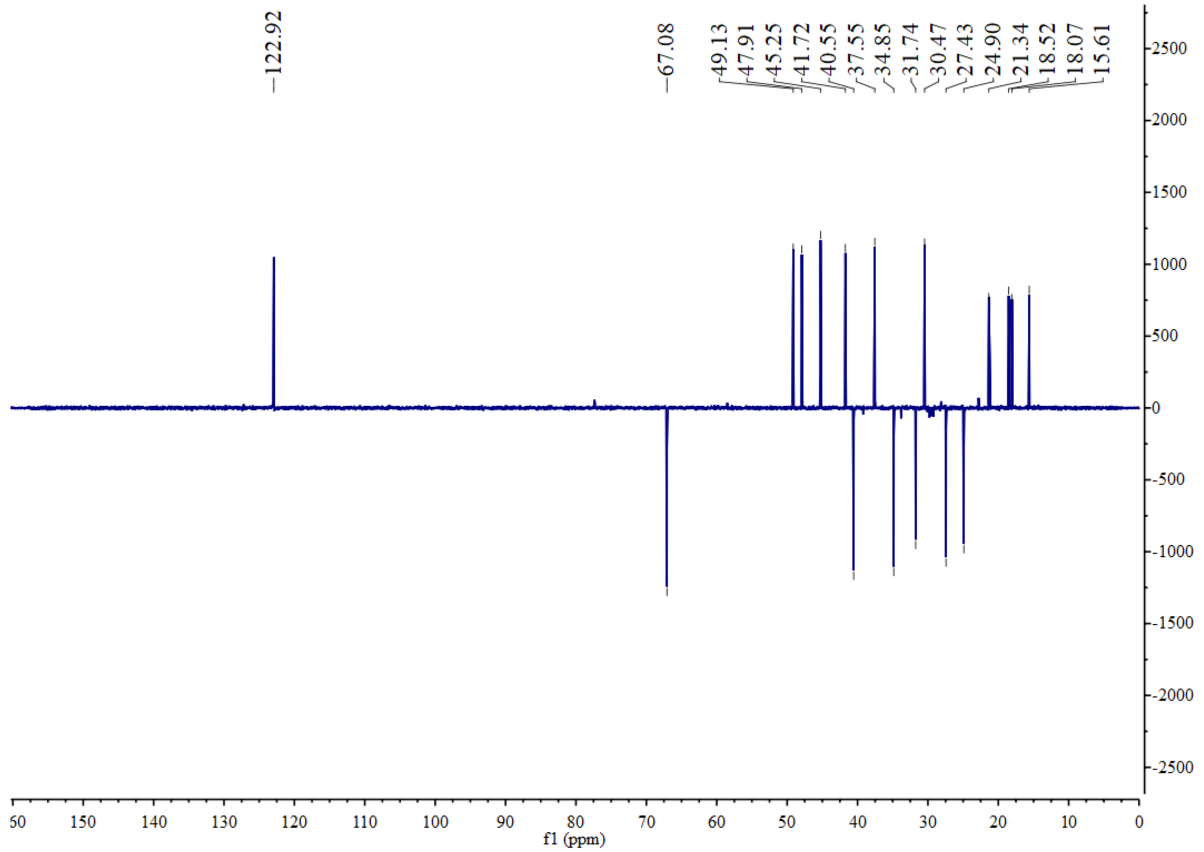

Figure S112 DEPT-135 NMR spectrum of **4a** in $CDCl_3$ (100 MHz).

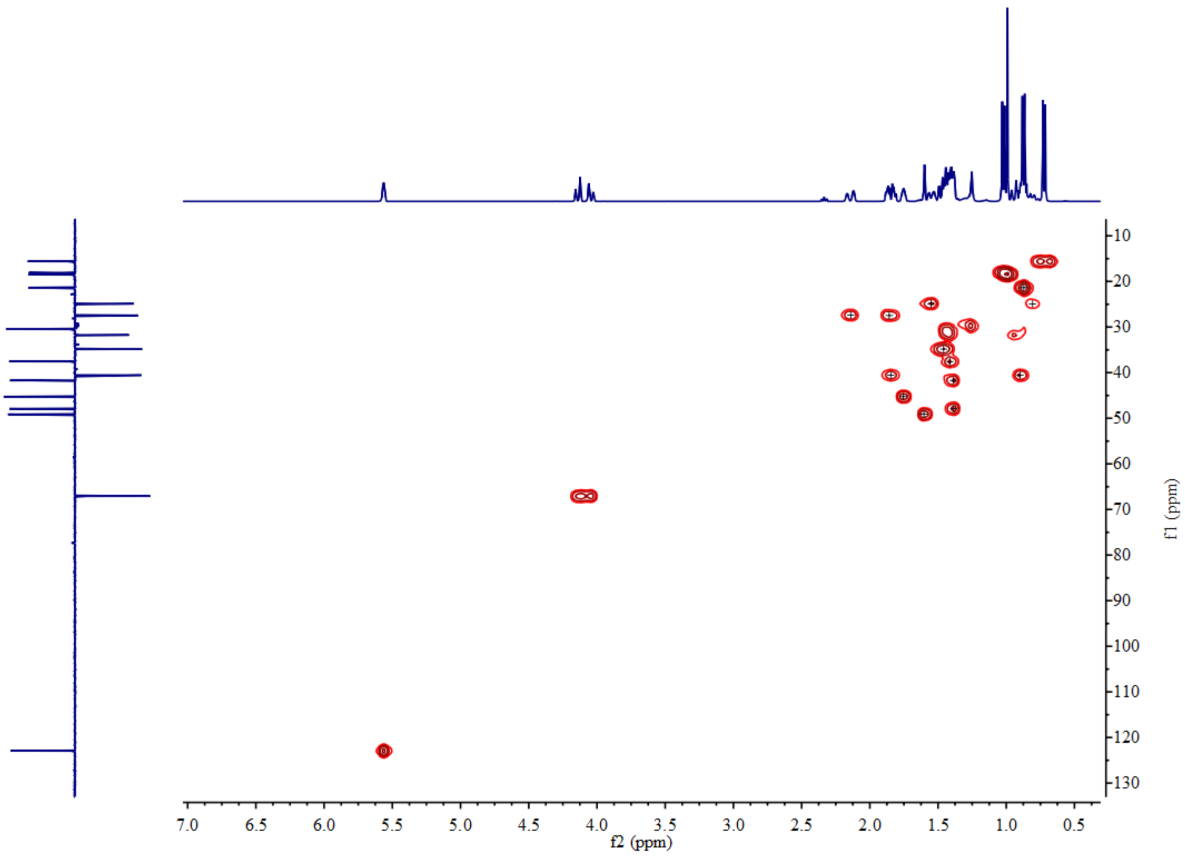

Figure S113 HSQC spectrum of **4a** in $CDCl_3$ (400 MHz).

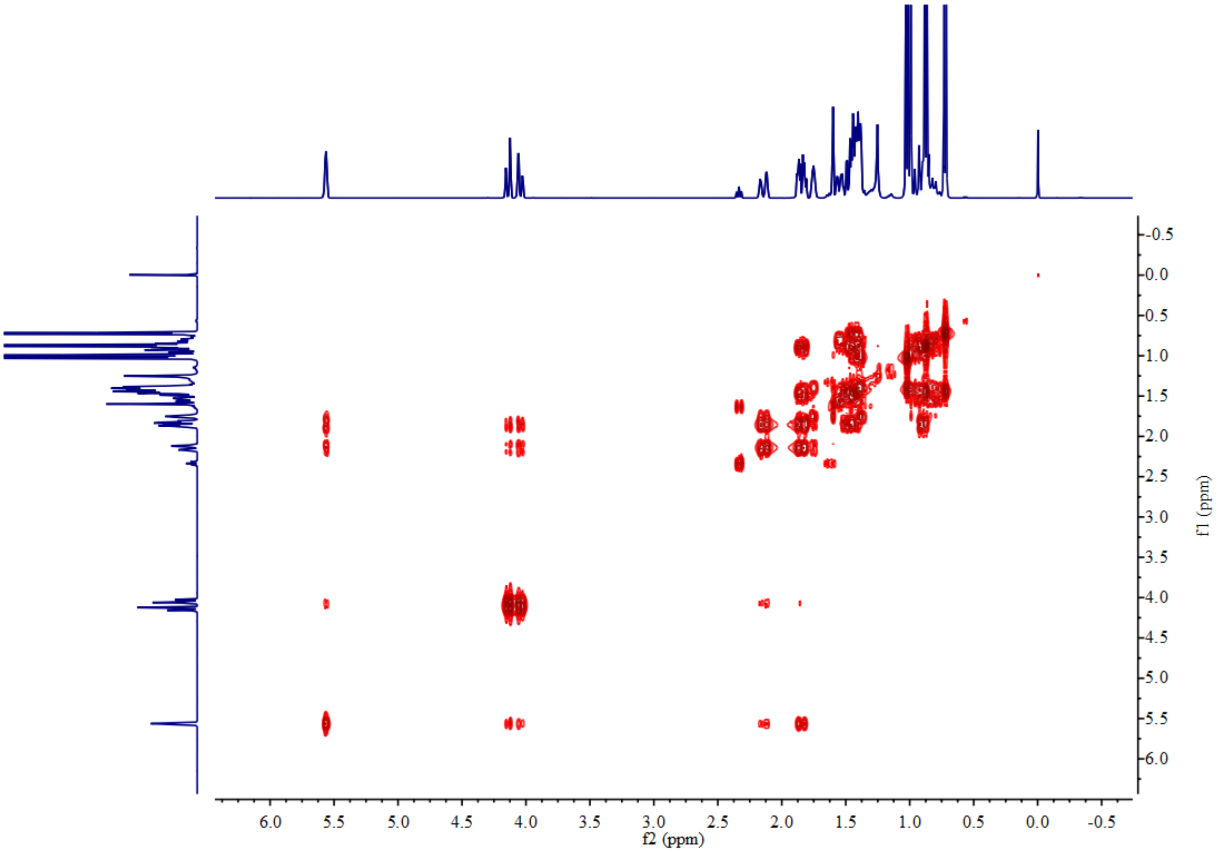

Figure S114 ^{1}H-^{1}H COSY spectrum of **4a** in $CDCl_3$ (400 MHz).

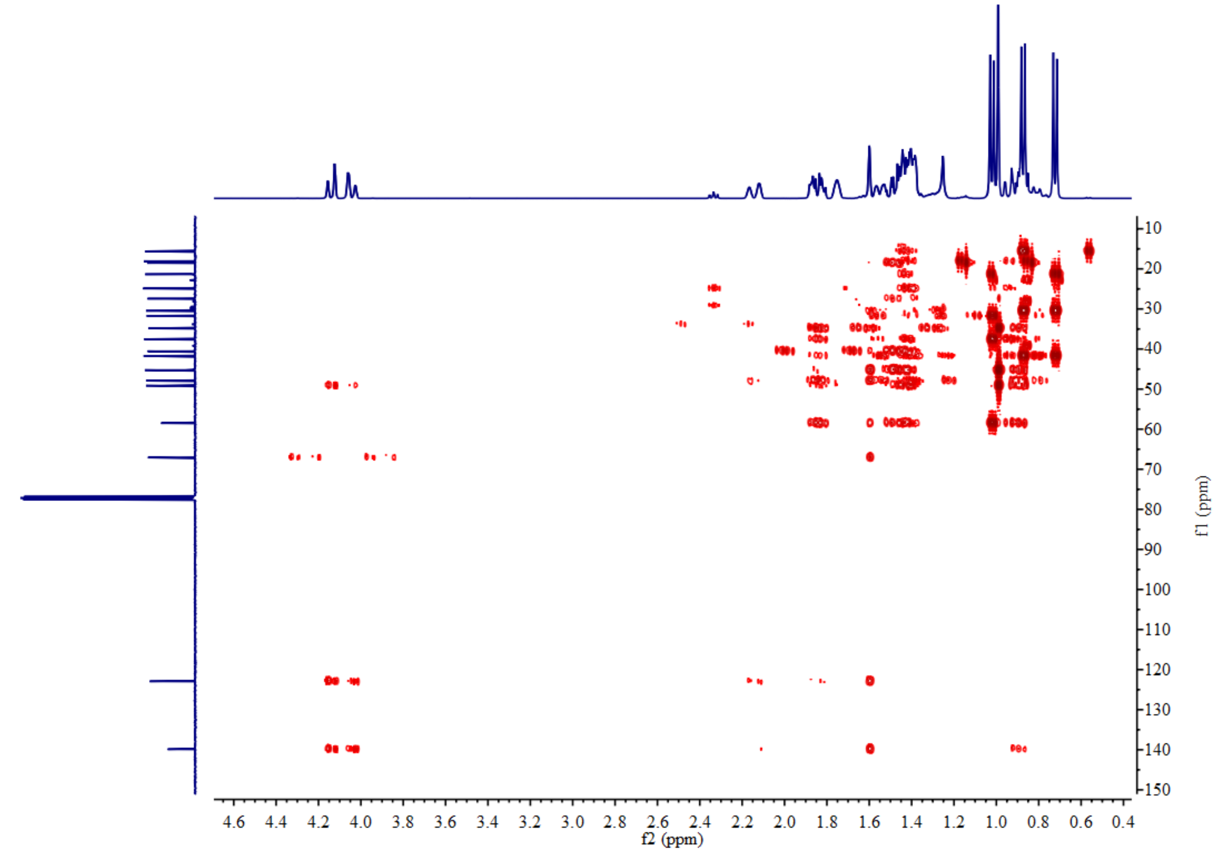

Figure S115 HMBC spectrum of **4a** in $CDCl_3$ (400 MHz).

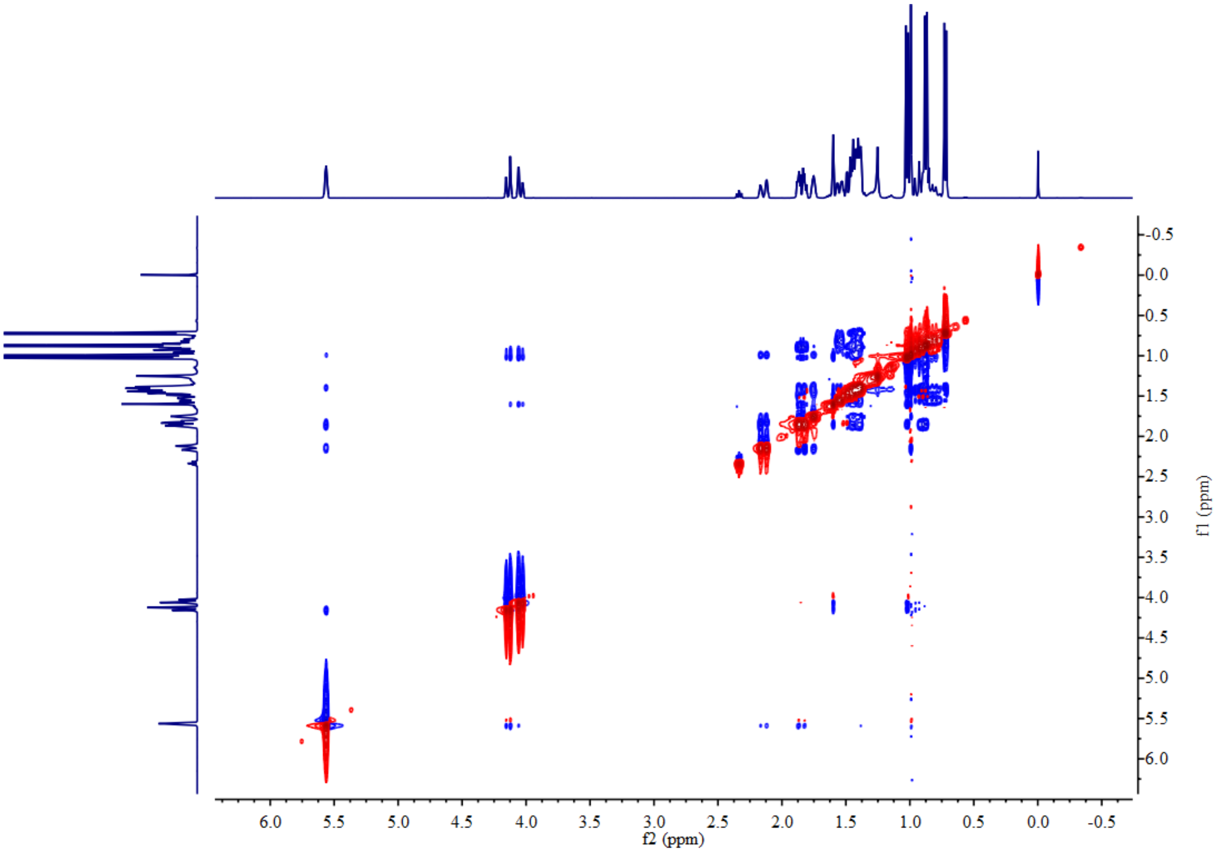

Figure S116 NOESY spectrum of **4a** in $CDCl_3$ (400 MHz).

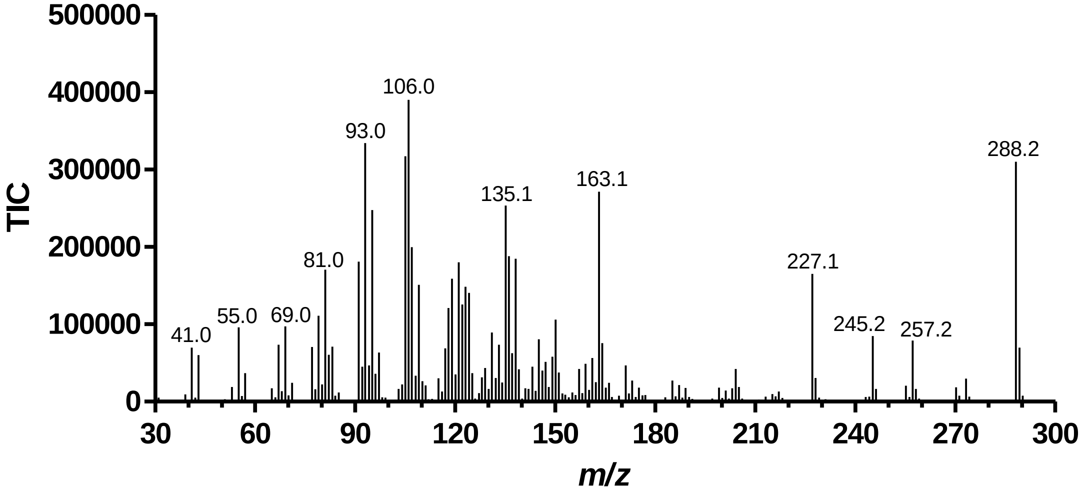

Figure S117 EI mass spectrum of **4a**.

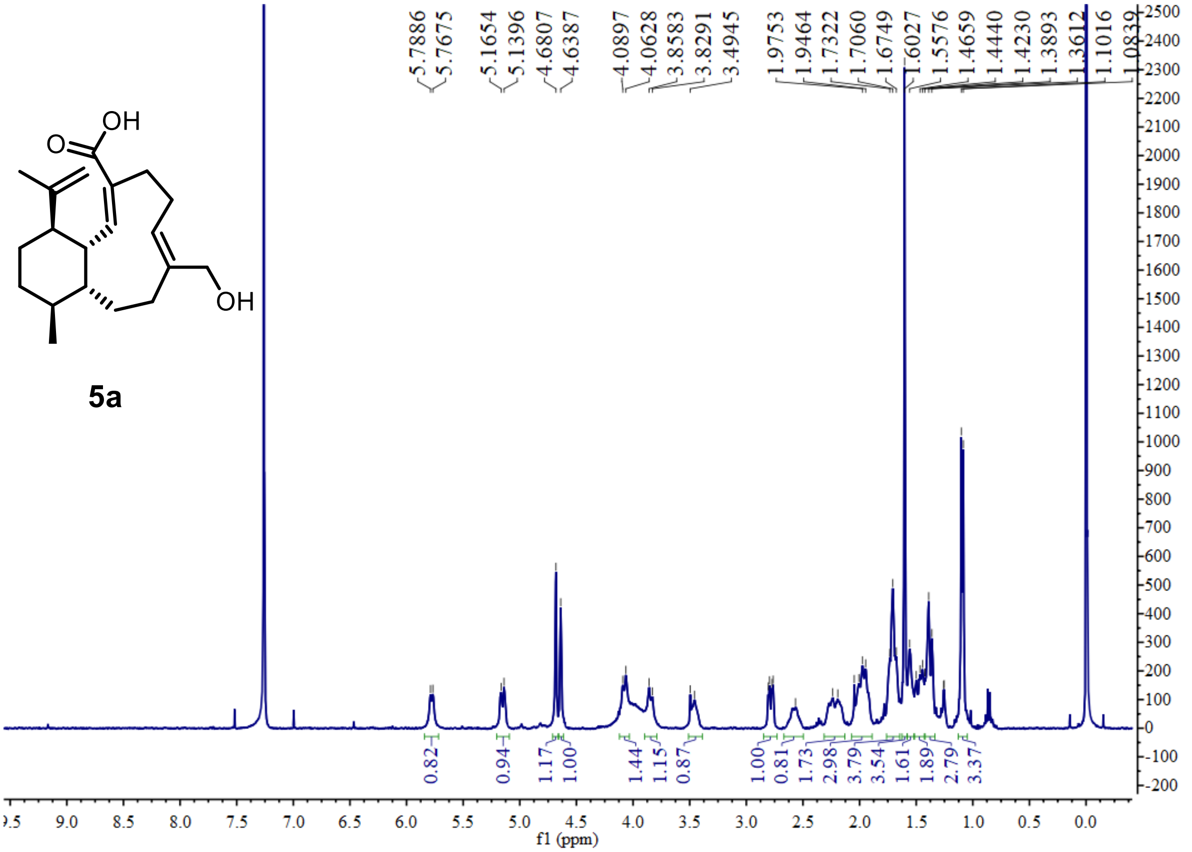

Figure S118 ^{1}H NMR spectrum of **5a** in $CDCl_3$ (400 MHz).

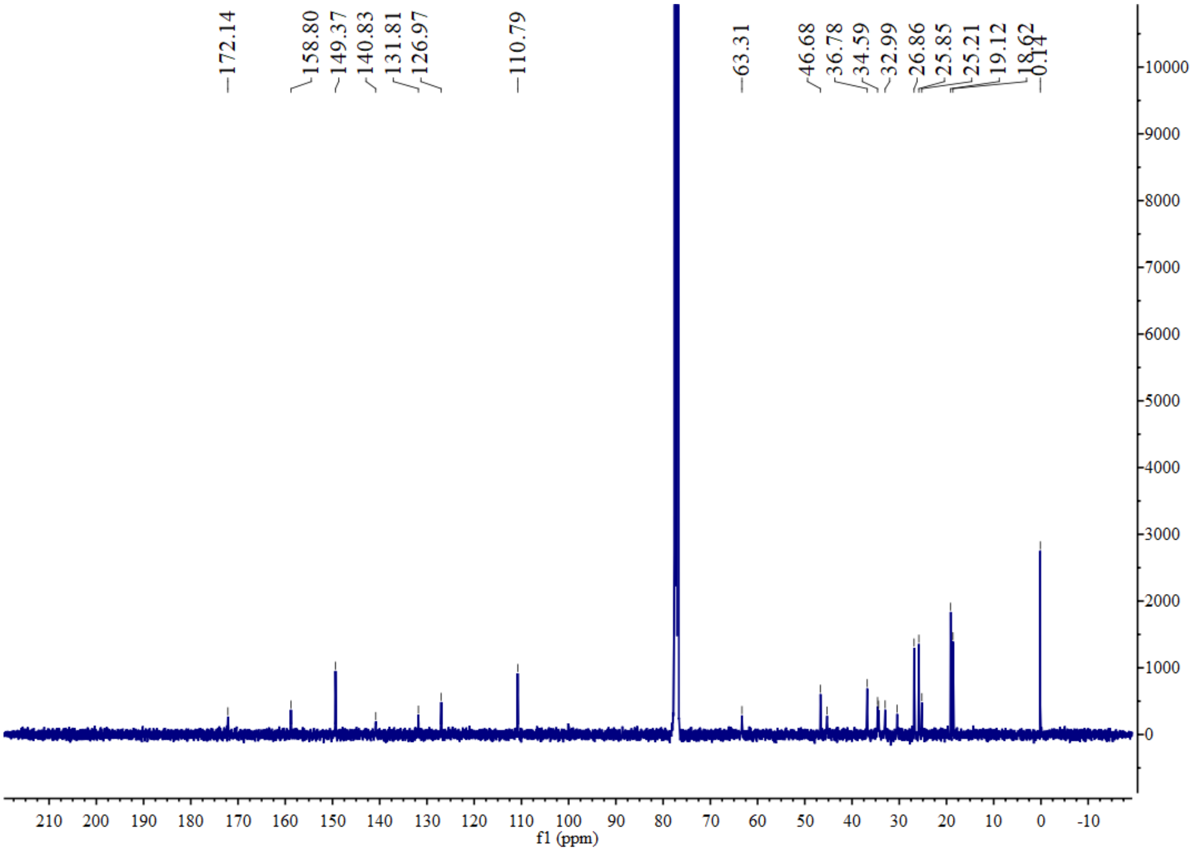

Figure S119 ^{13}C NMR spectrum of **5a** in $CDCl_3$ (100 MHz).

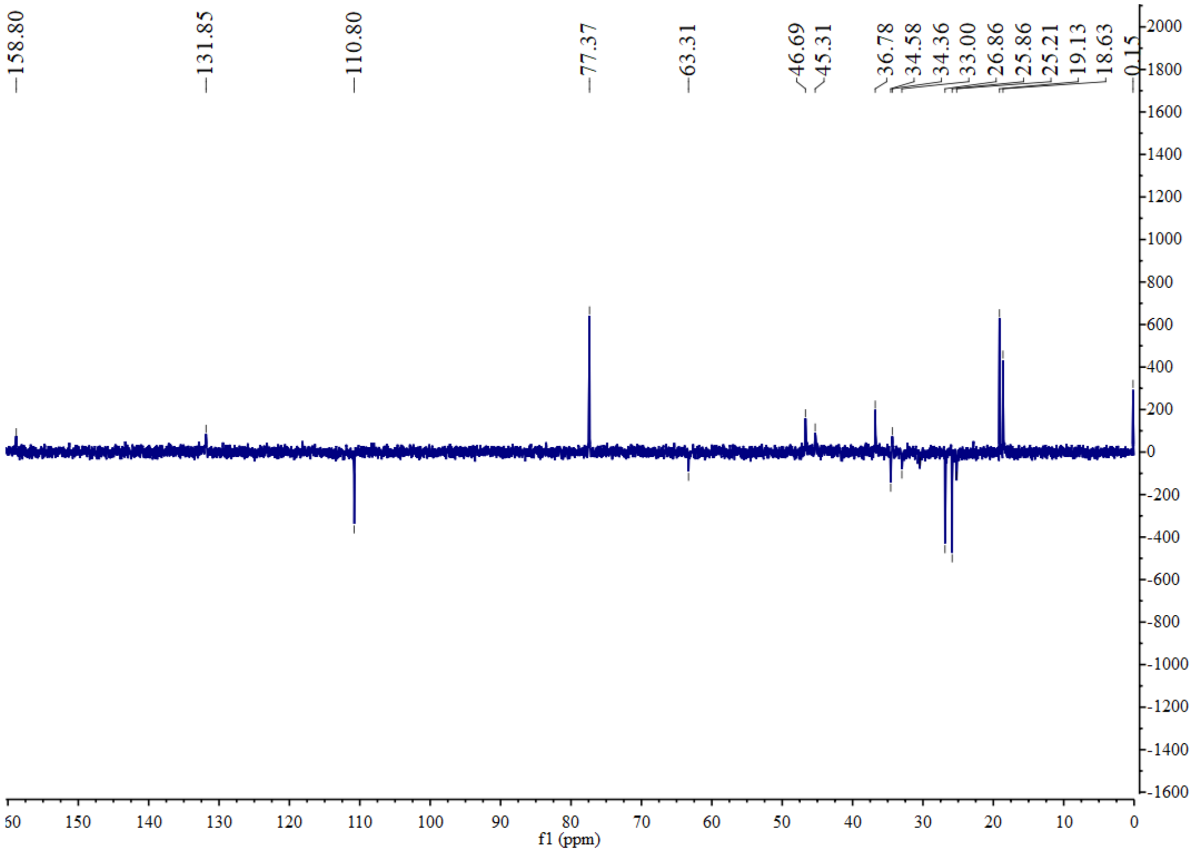

Figure S120 DEPT-135 NMR spectrum of **5a** in $CDCl_3$ (100 MHz).

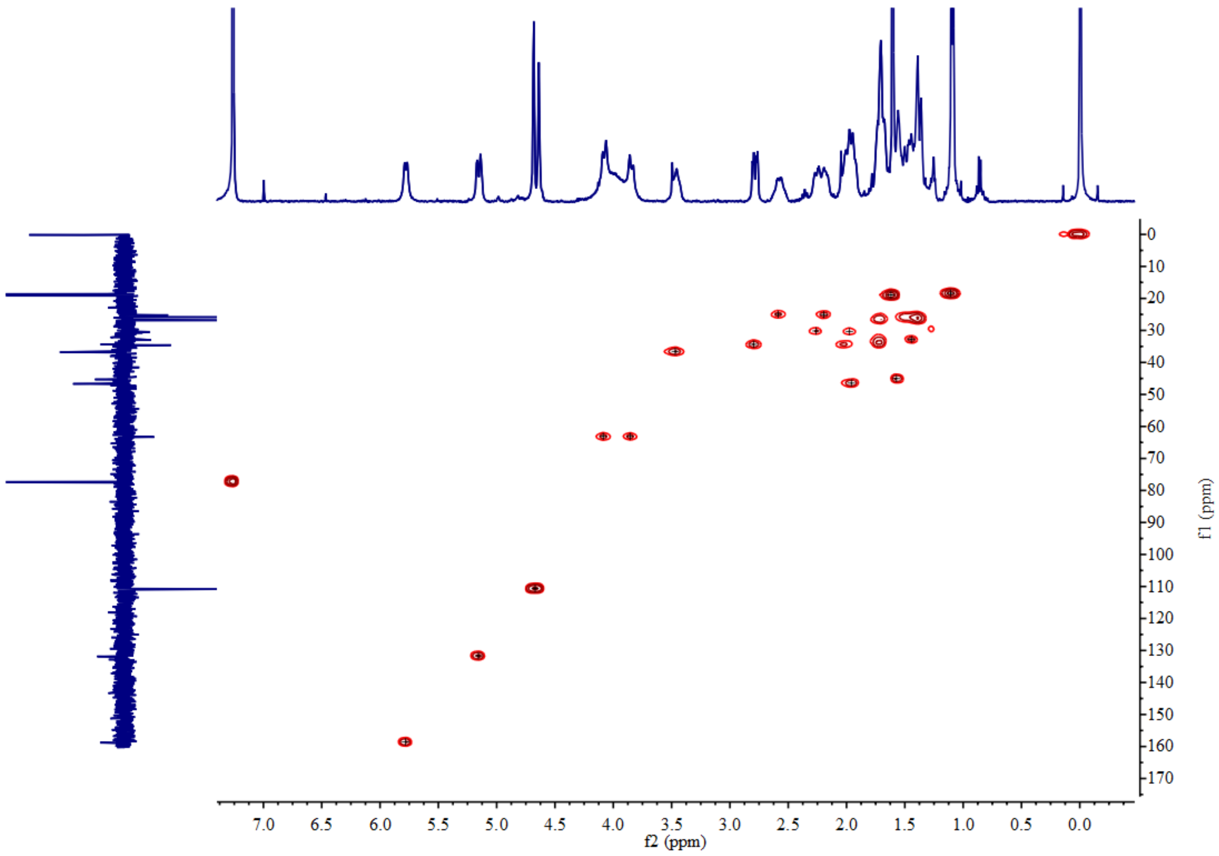

Figure S121 HSQC spectrum of **5a** in $CDCl_3$ (400 MHz).

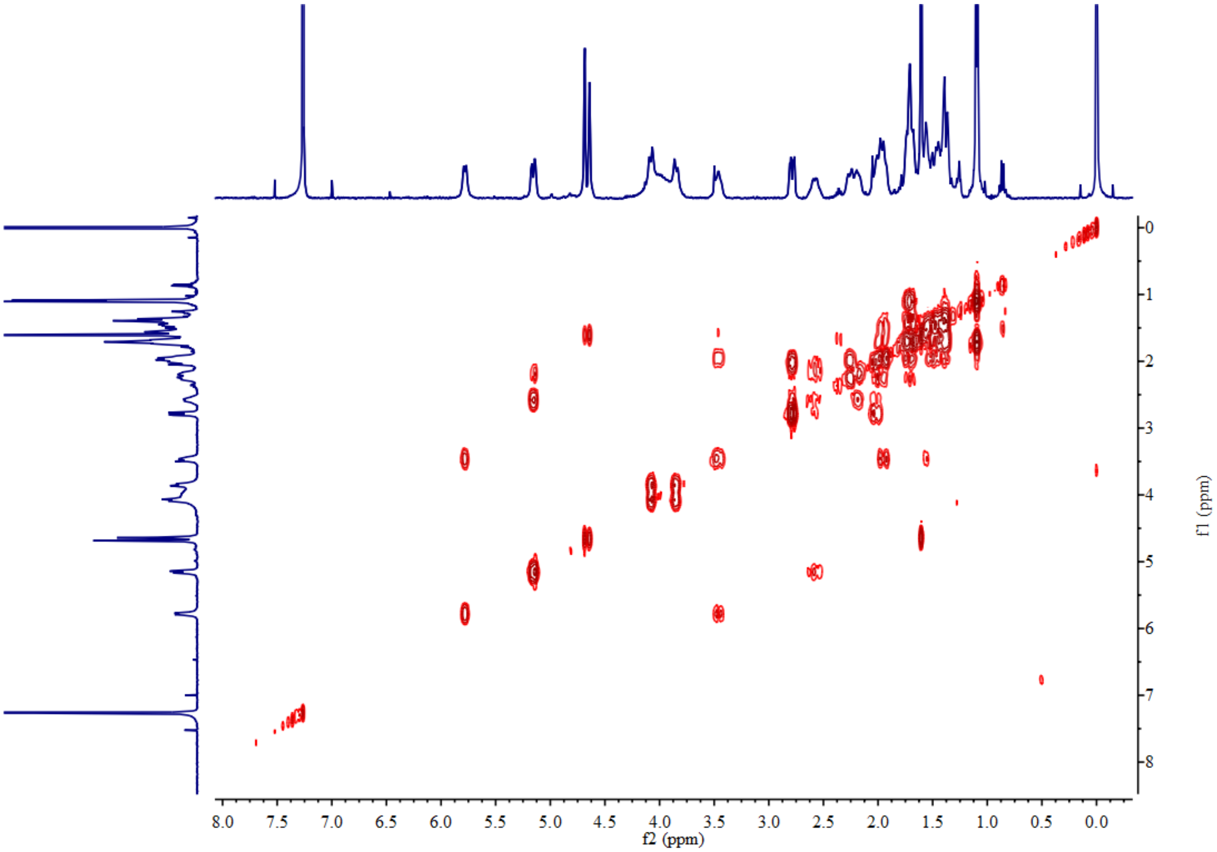

Figure S122 ^1H-^1H COSY spectrum of **5a** in $CDCl_3$ (400 MHz).

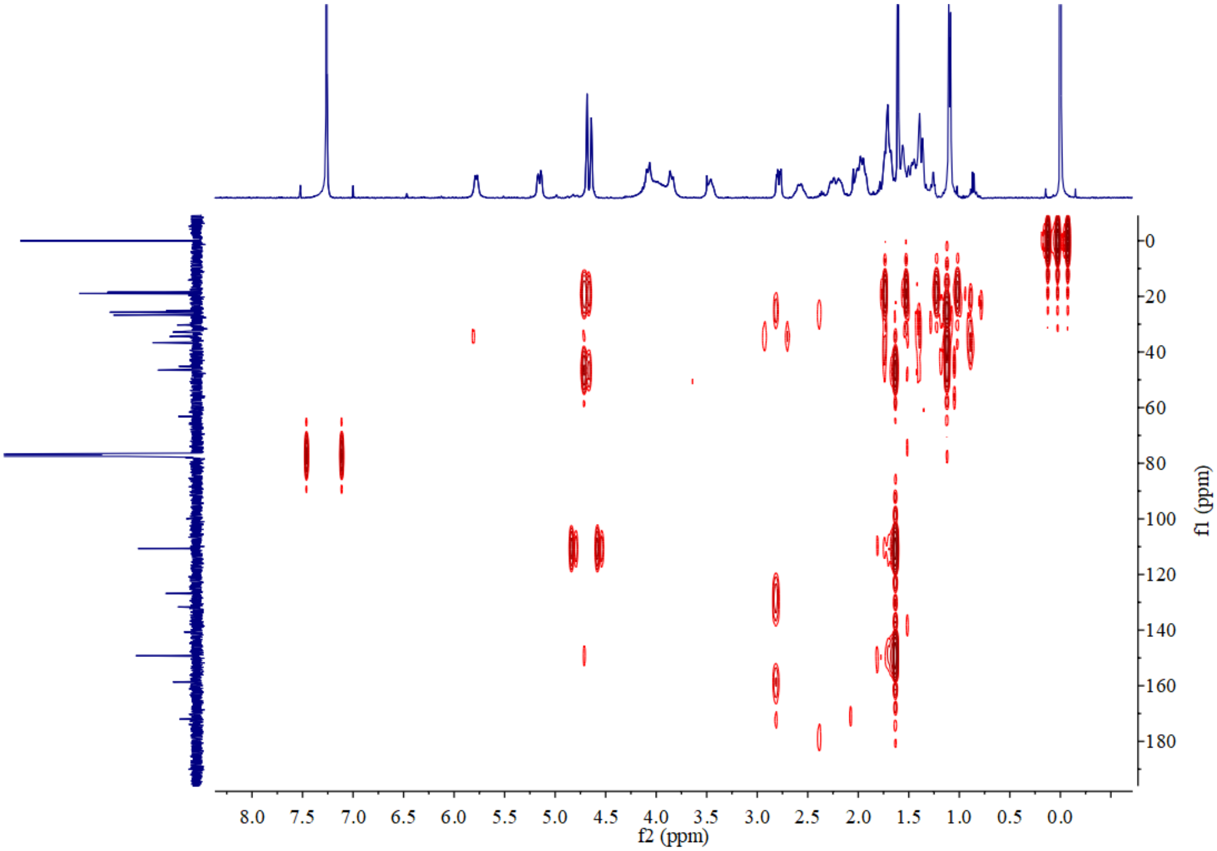

Figure S123 HMBC spectrum of **5a** in $CDCl_3$ (400 MHz).

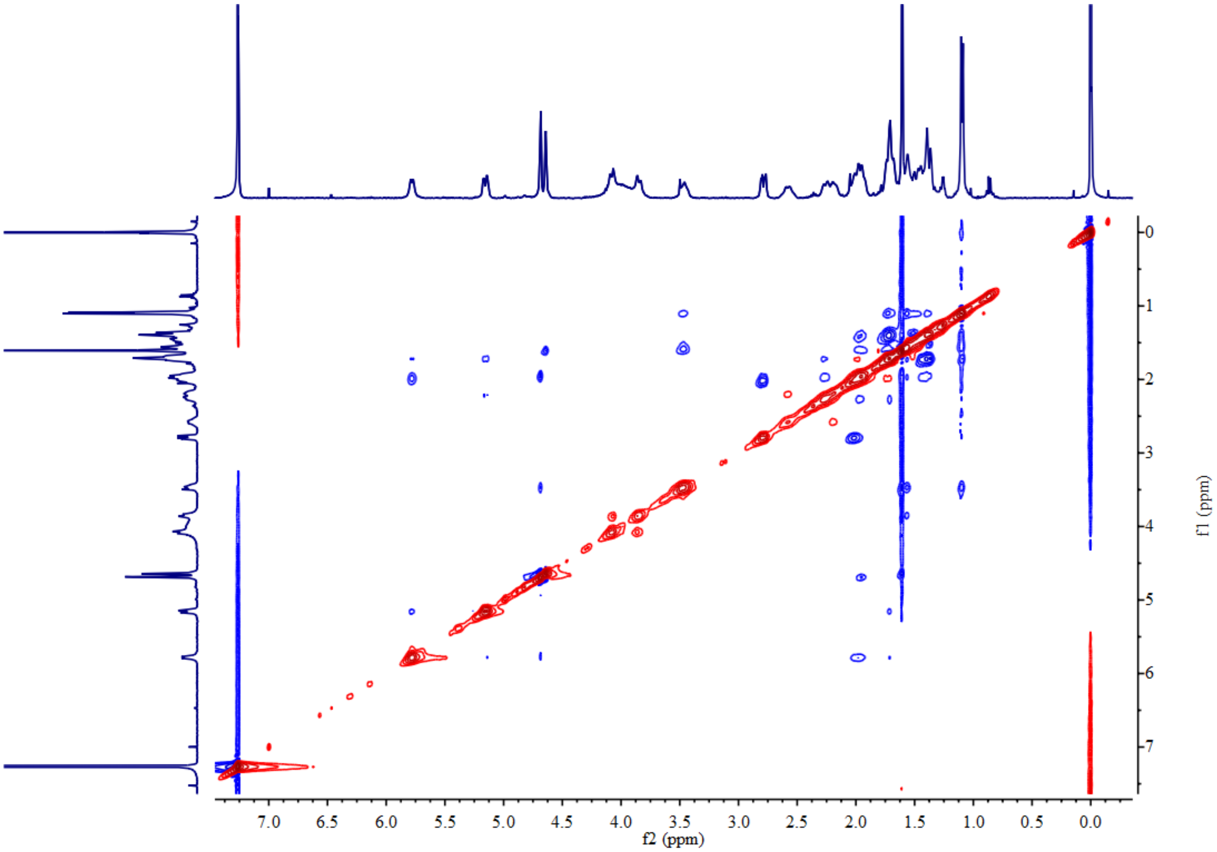

Figure S124 NOESY spectrum of **5a** in $CDCl_3$ (400 MHz).

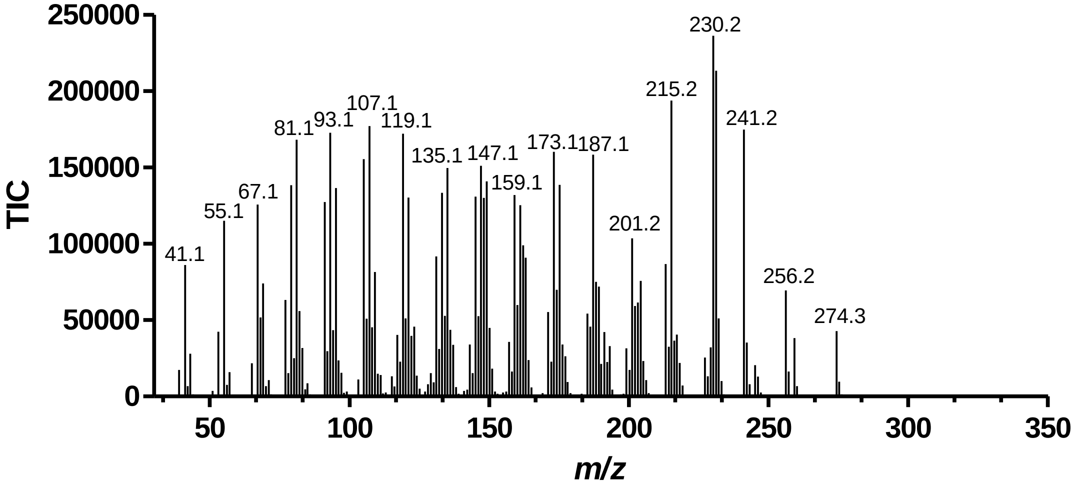

Figure S125 EI mass spectrum of **5a**.

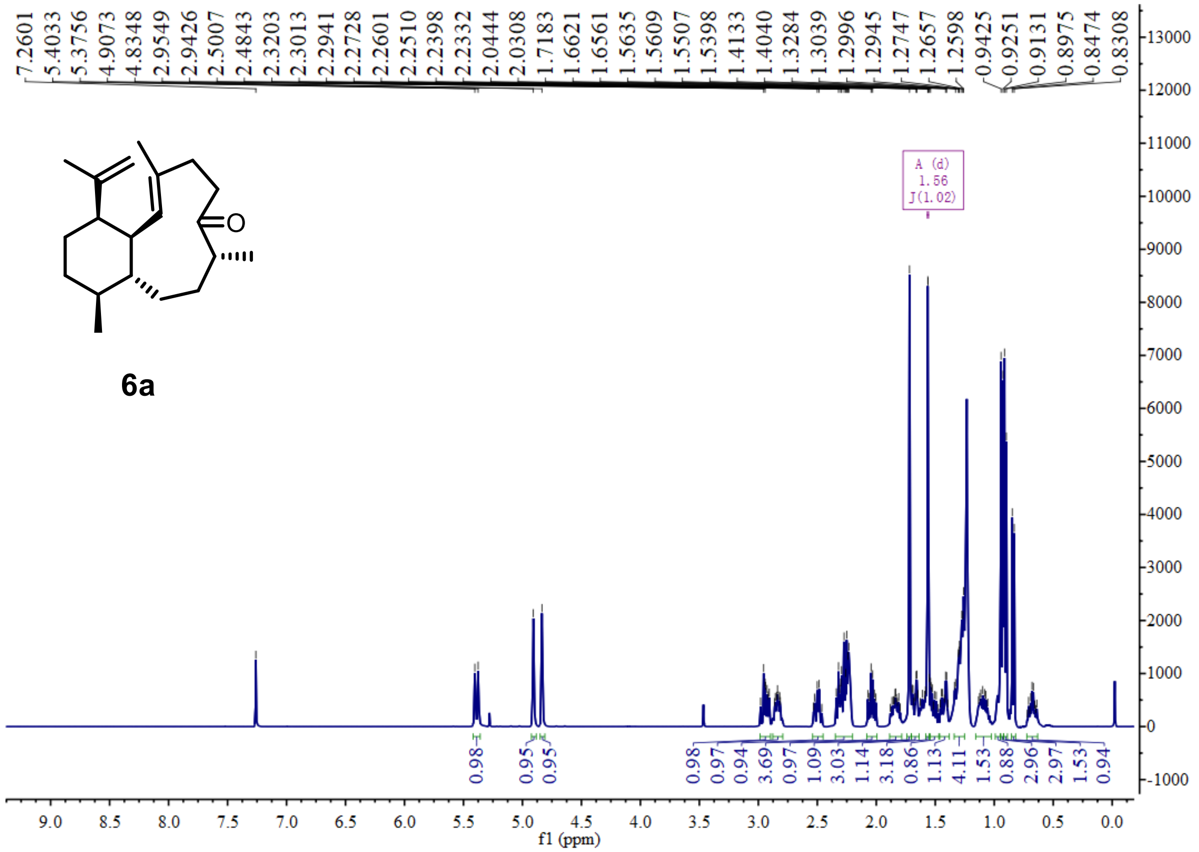

Figure S126 ^{1}H NMR spectrum of **6a** in $CDCl_3$ (400 MHz).

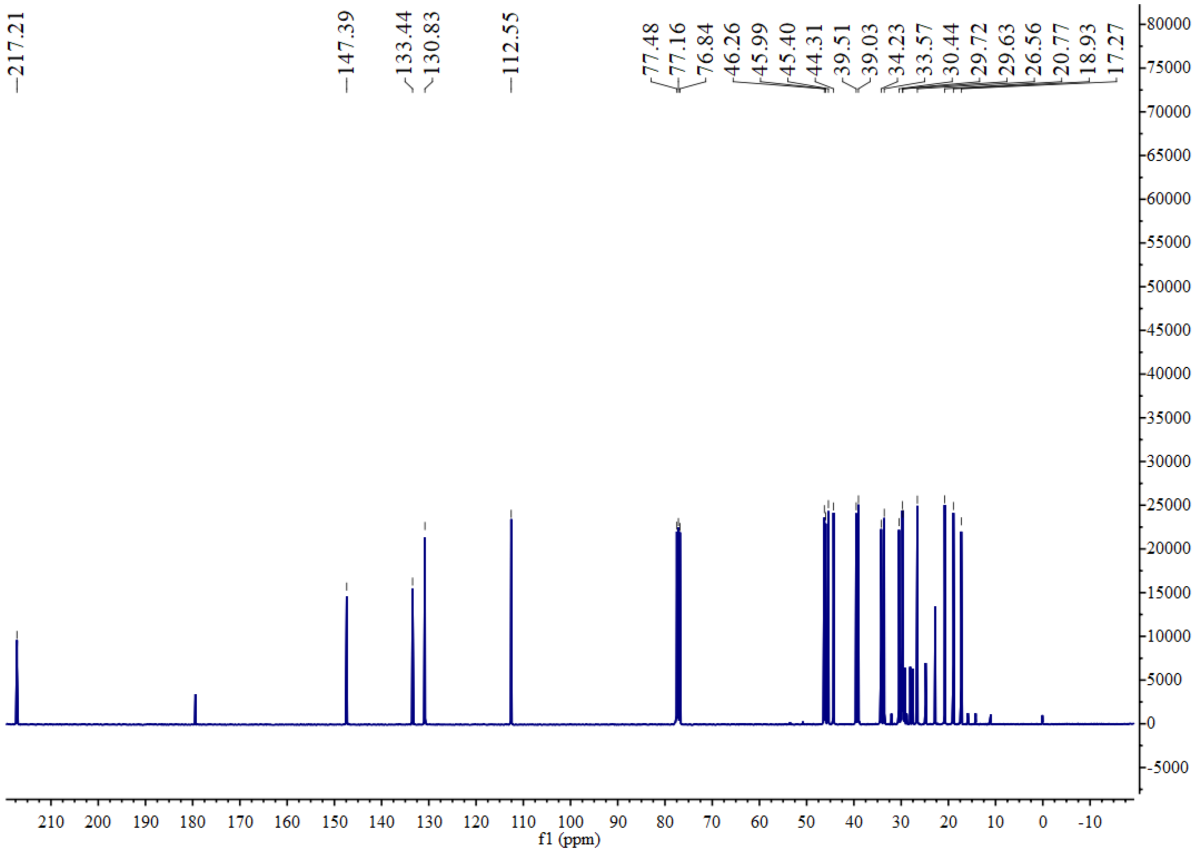

Figure S127 ^{13}C NMR spectrum of **6a** in $CDCl_3$ (100 MHz).

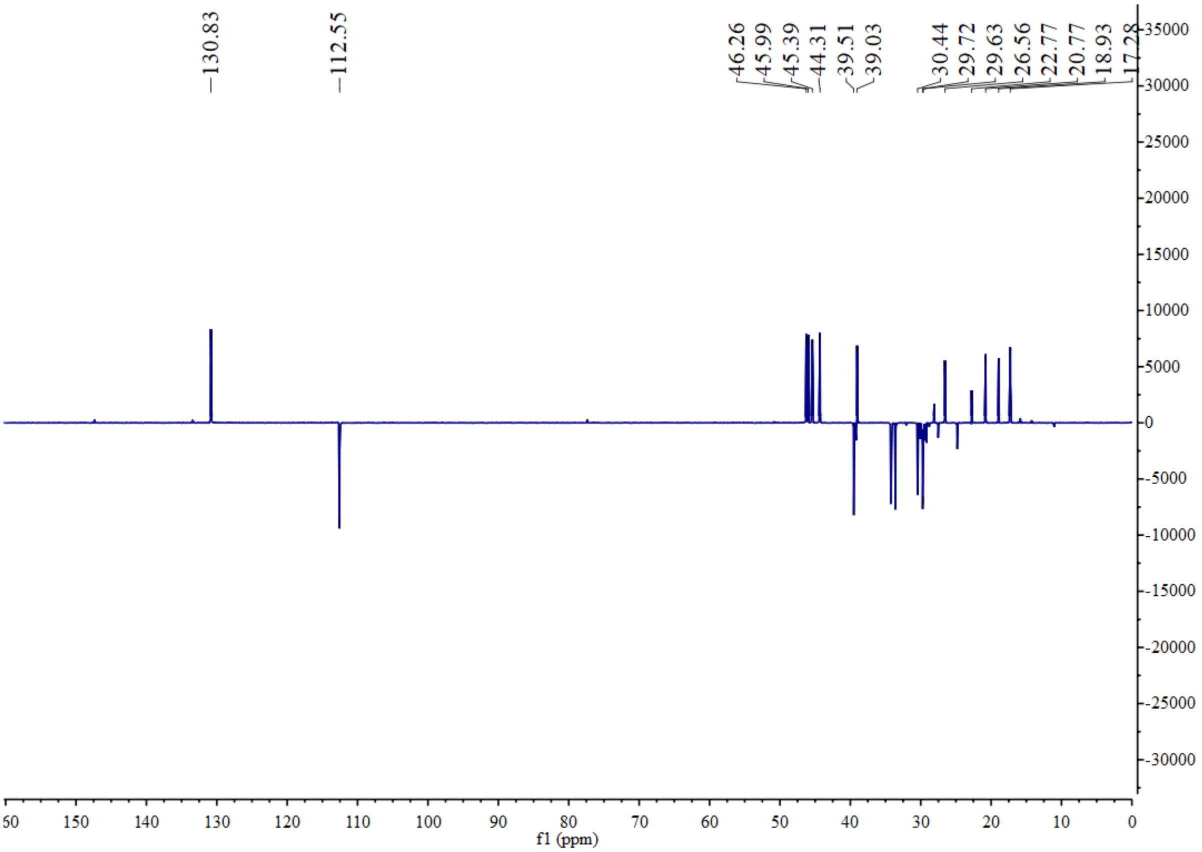

Figure S128 DEPT-135 NMR spectrum of **6a** in $CDCl_3$ (100 MHz).

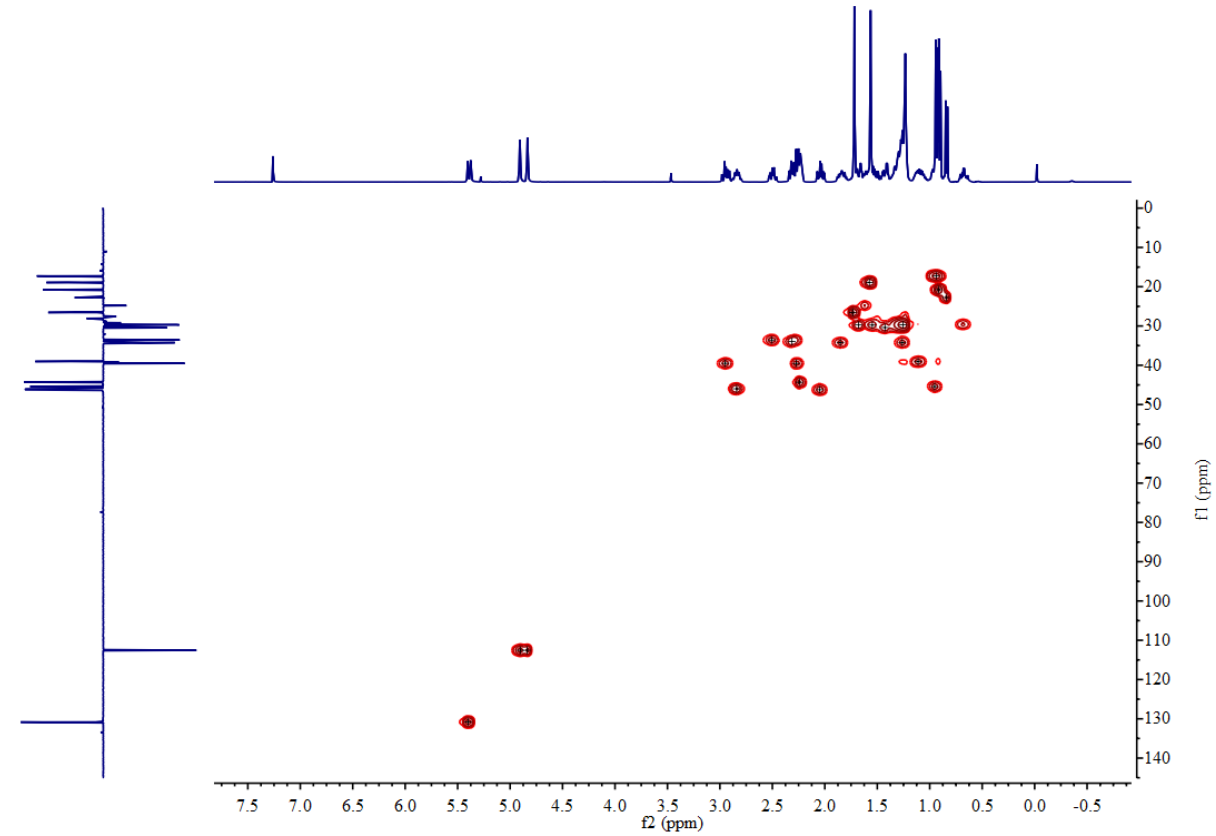

Figure S129 HSQC spectrum of **6a** in $CDCl_3$ (400 MHz).

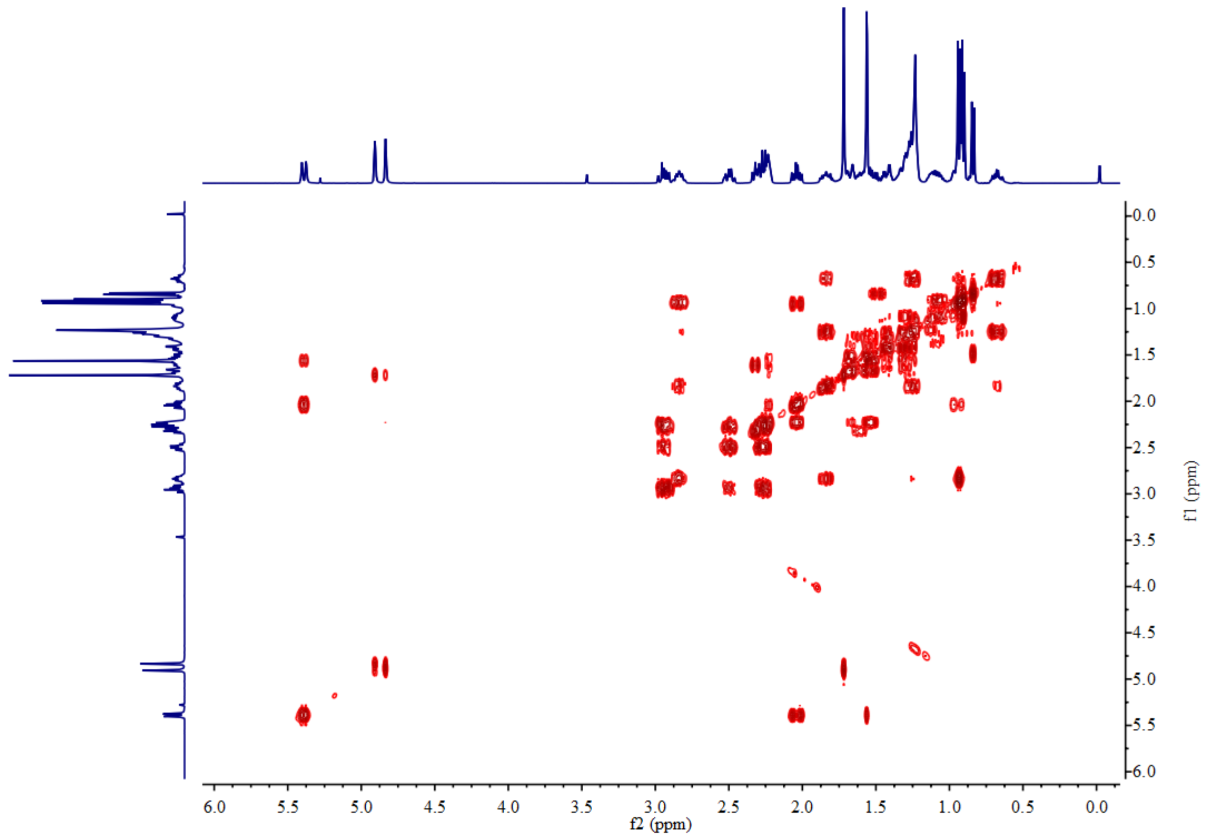

Figure S130 ^1H-^1H COSY spectrum of **6a** in $CDCl_3$ (400 MHz).

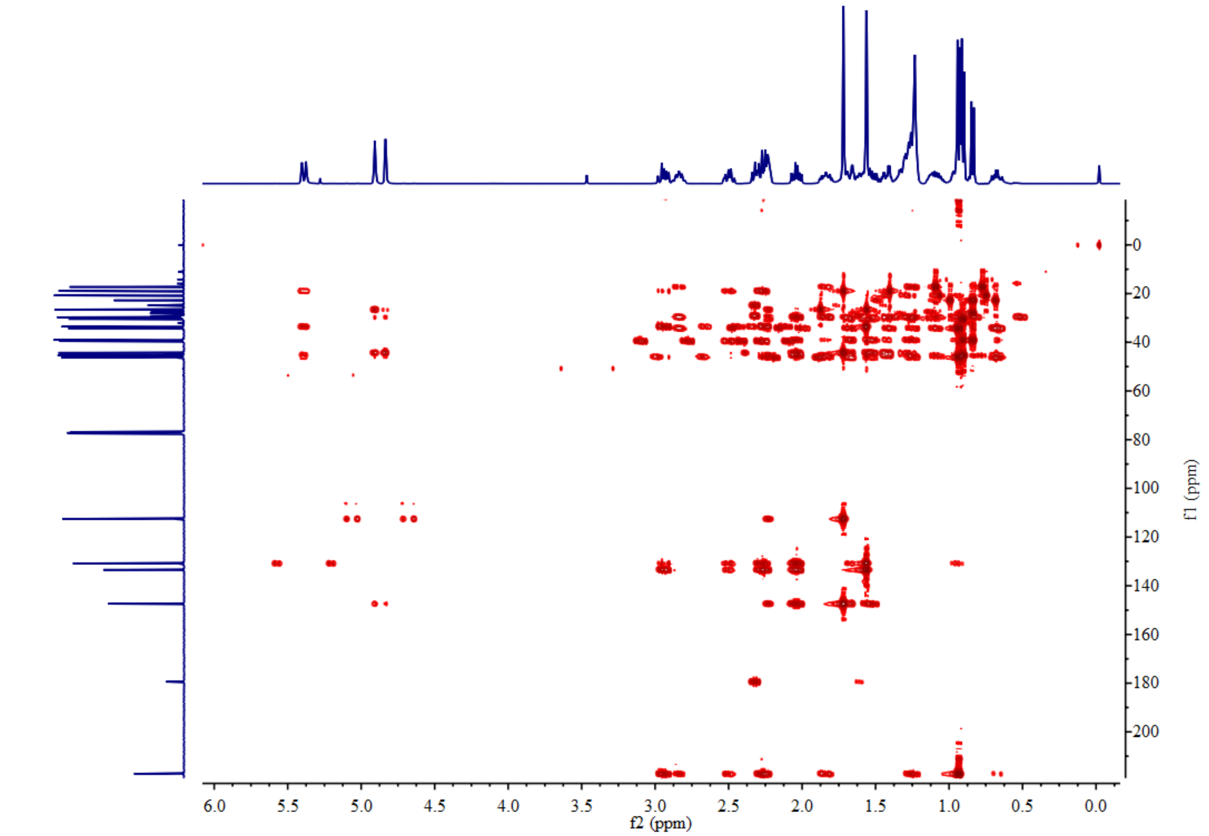

Figure S131 HMBC spectrum of **6a** in $CDCl_3$ (400 MHz).

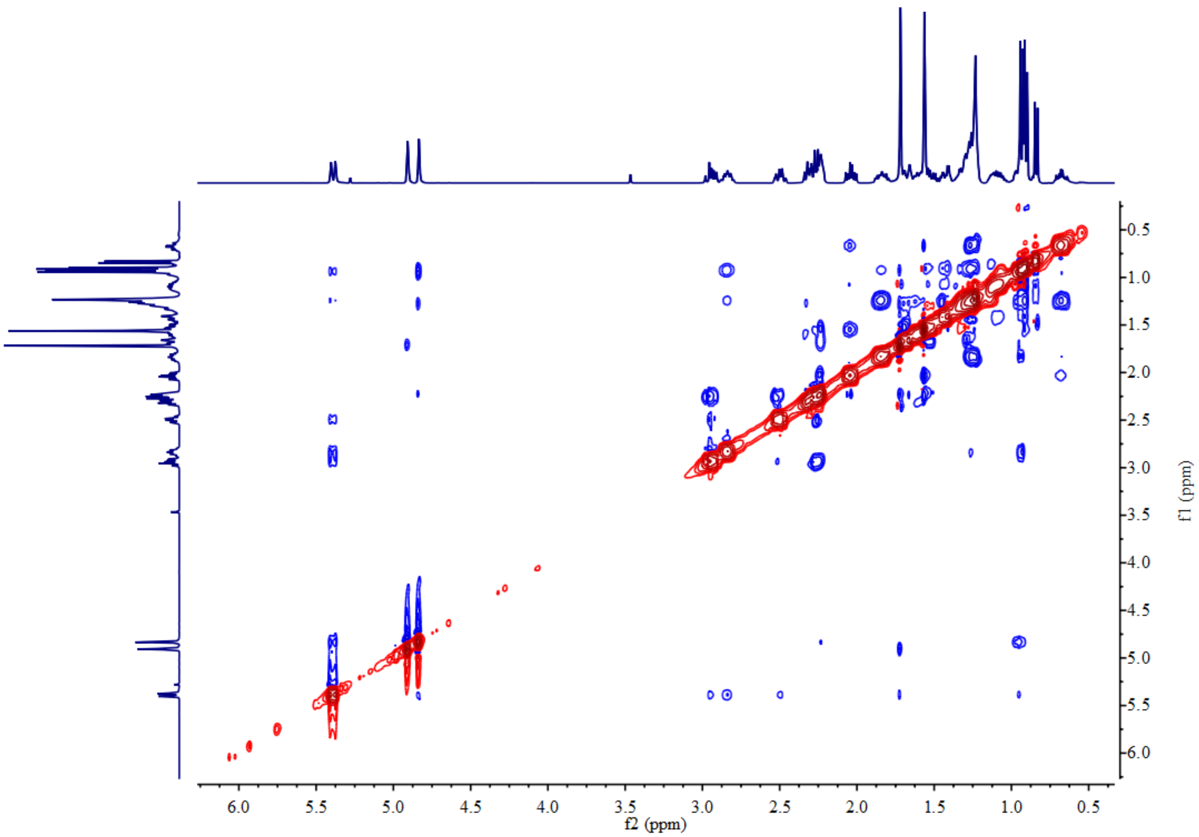

Figure S132 NOESY spectrum of **6a** in $CDCl_3$ (400 MHz).

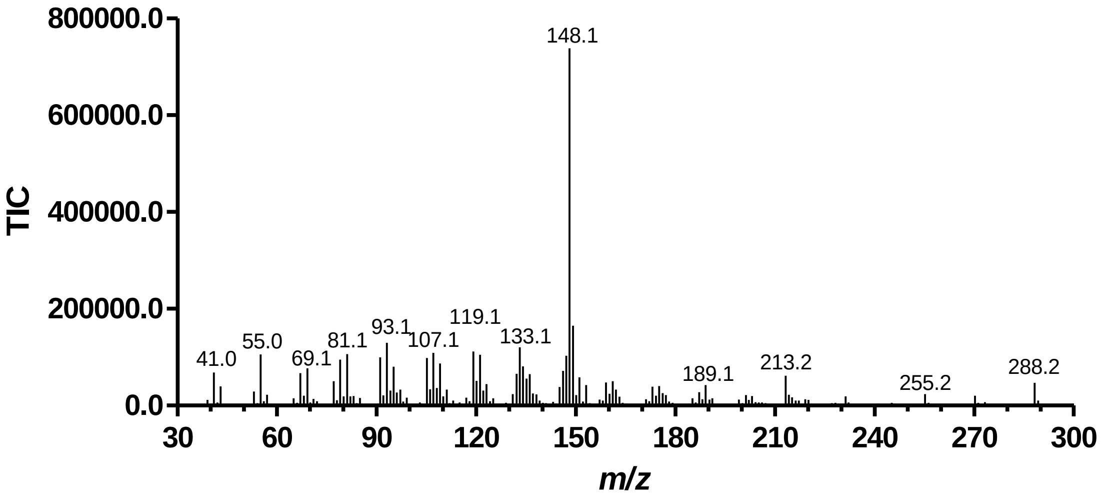

Figure S133 EI mass spectrum of **6a**.

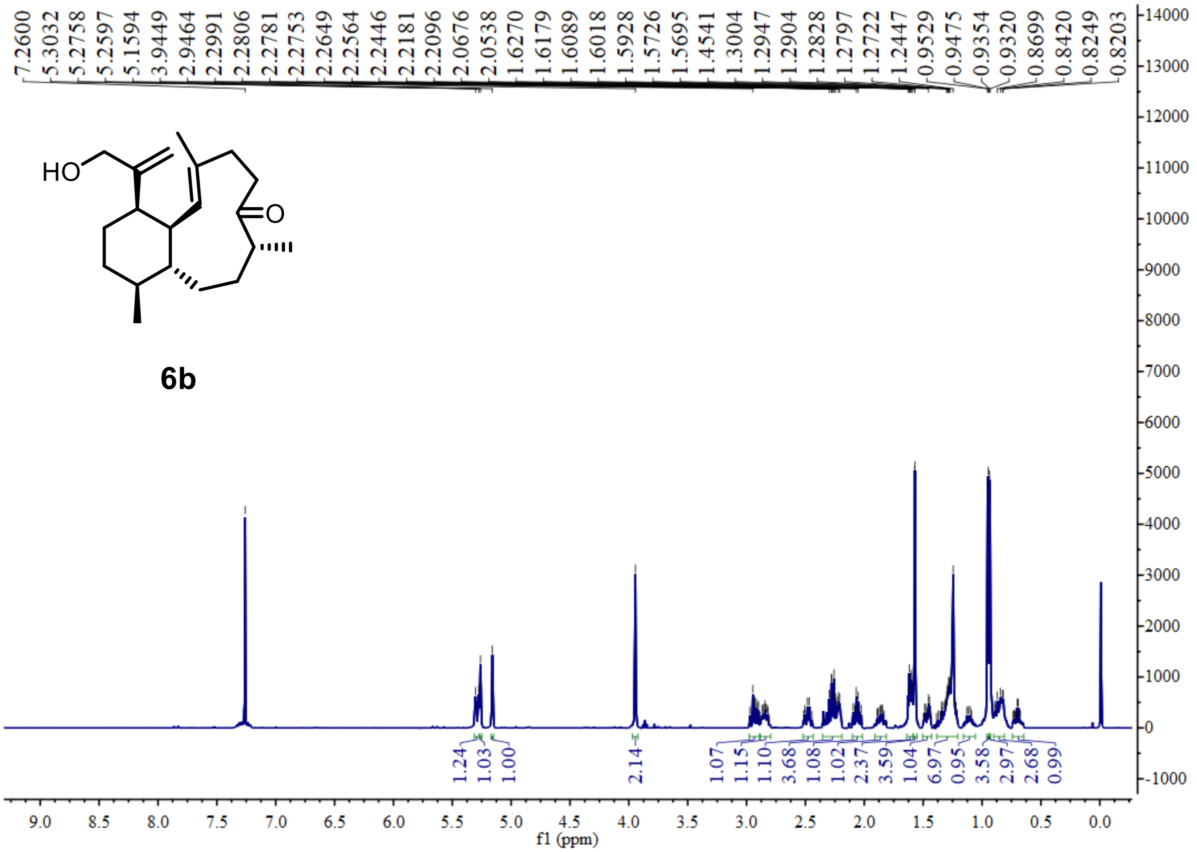

6b

Figure S134 ^{1}H NMR spectrum of **6b** in $CDCl_3$ (400 MHz).

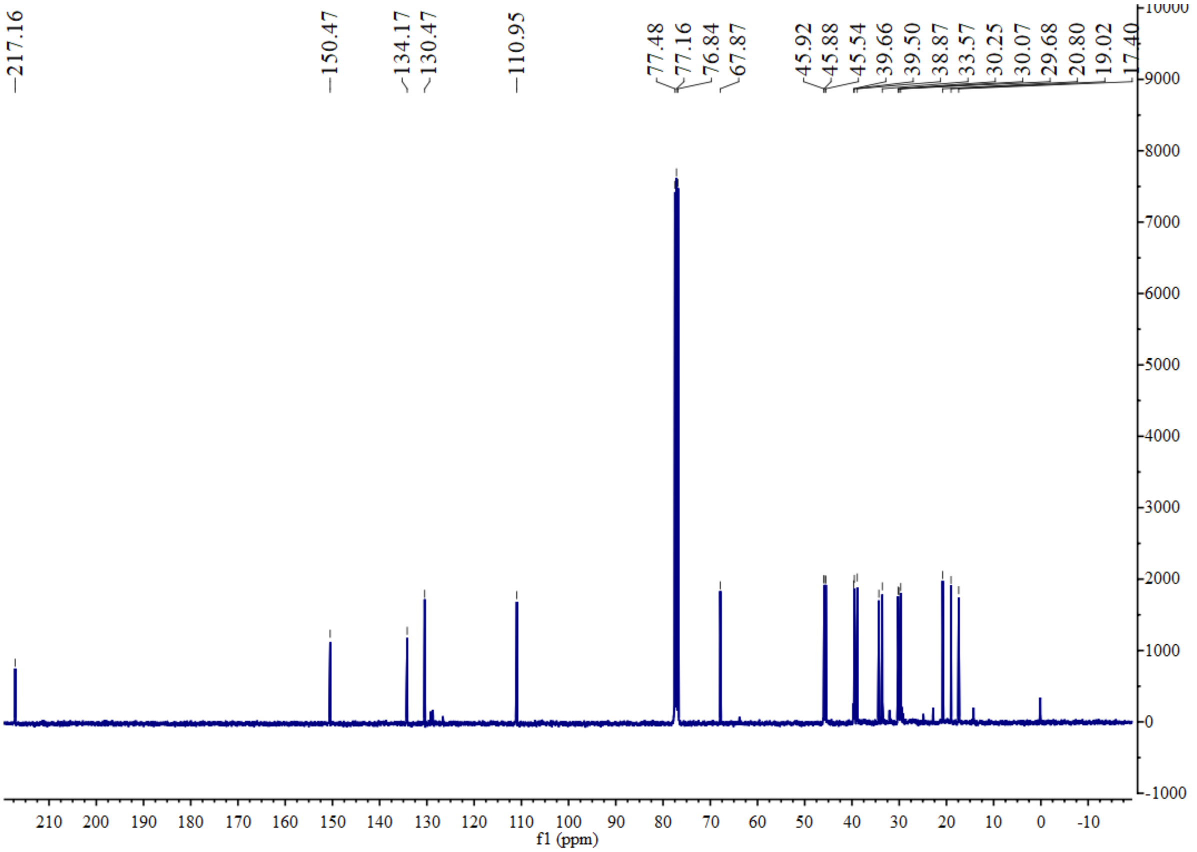

Figure S135 ^{13}C NMR spectrum of **6b** in $CDCl_3$ (100 MHz).

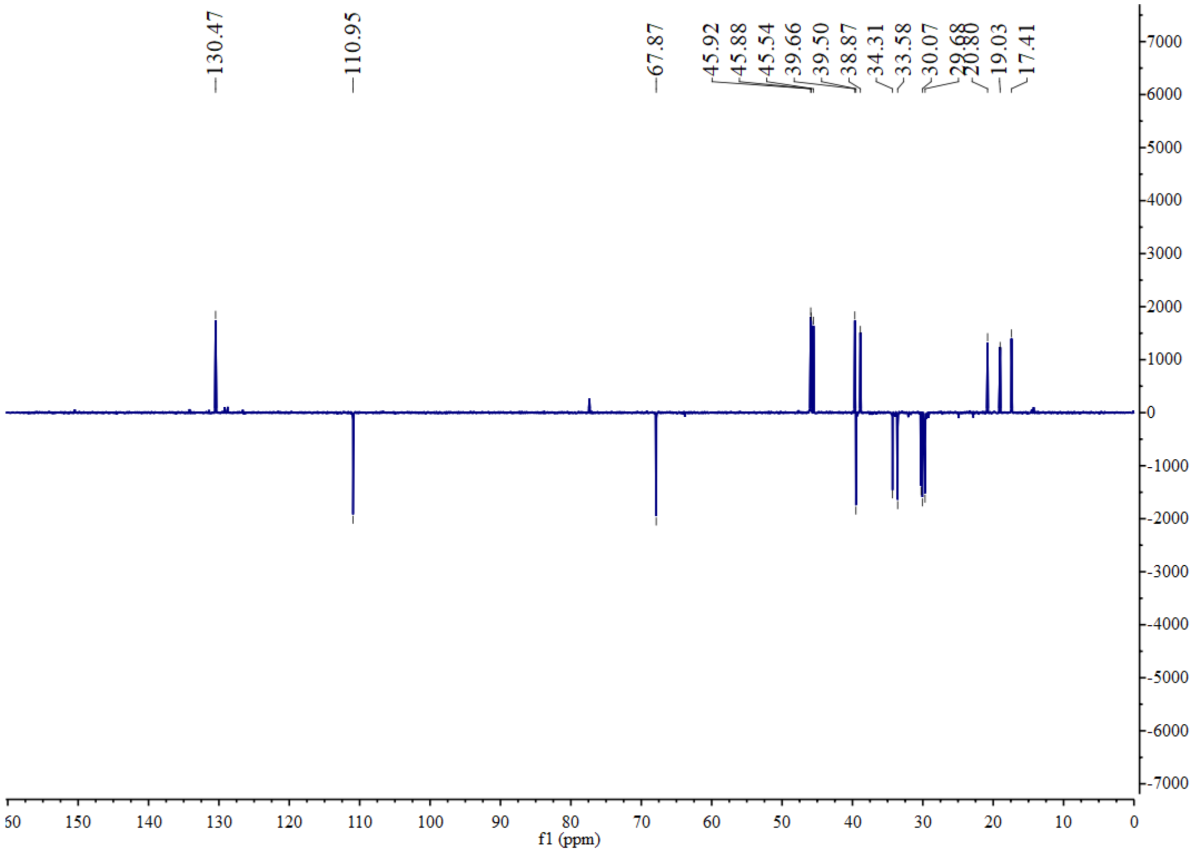

Figure S136 DEPT-135 NMR spectrum of **6b** in $CDCl_3$ (100 MHz).

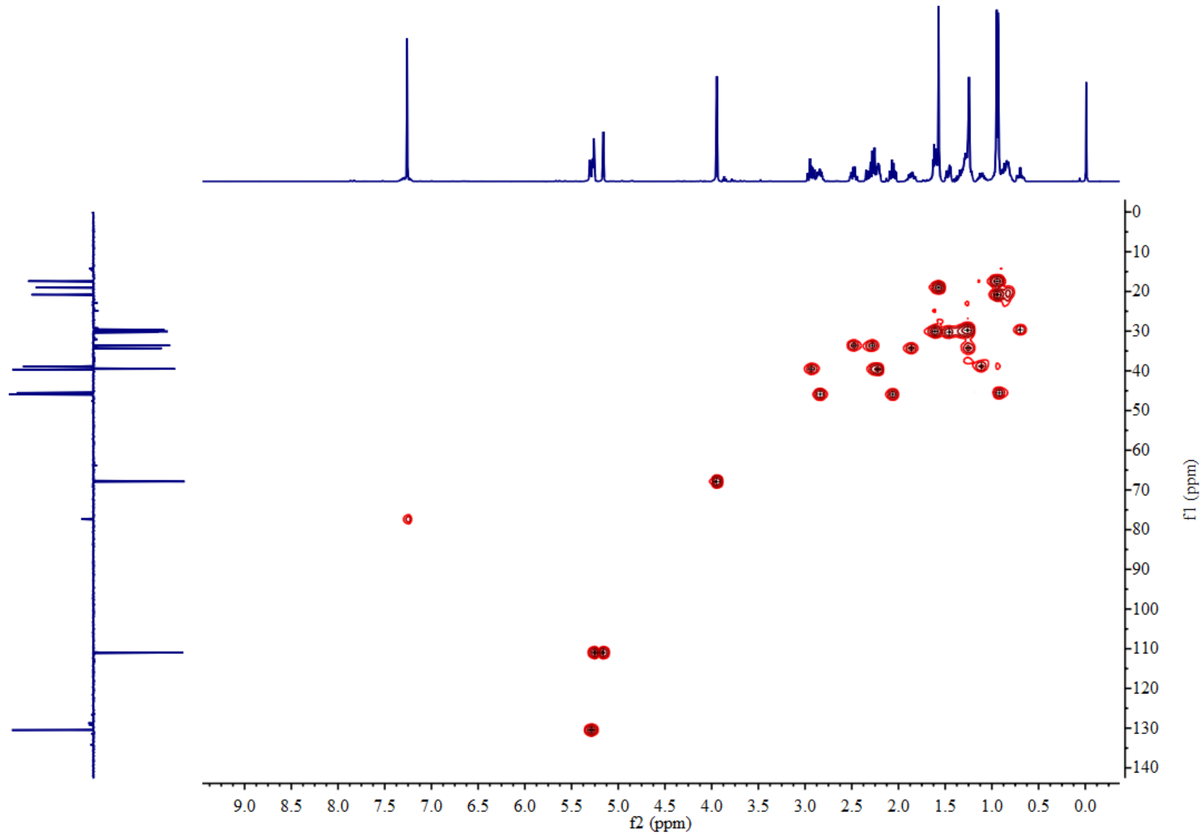

Figure S137 HSQC spectrum of **6b** in $CDCl_3$ (400 MHz).

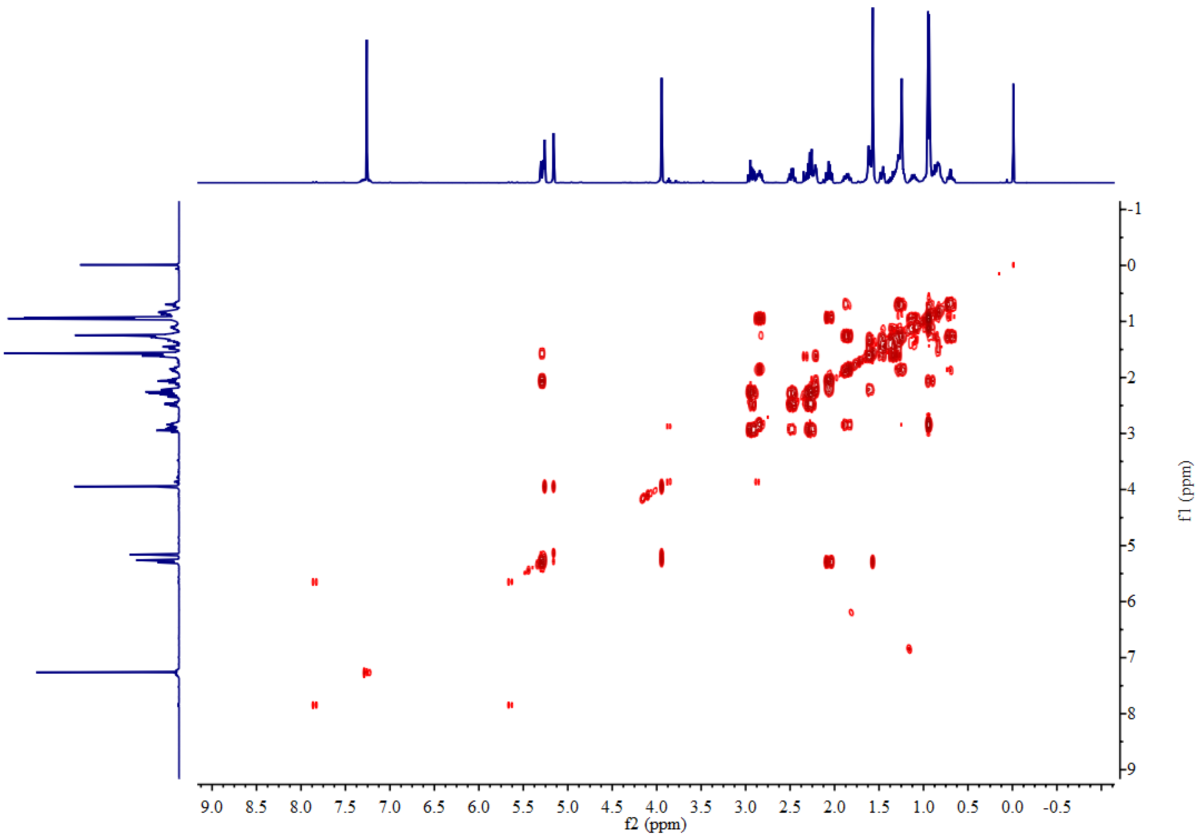

Figure S138 ^{1}H-^{1}H COSY spectrum of **6b** in $CDCl_3$ (400 MHz).

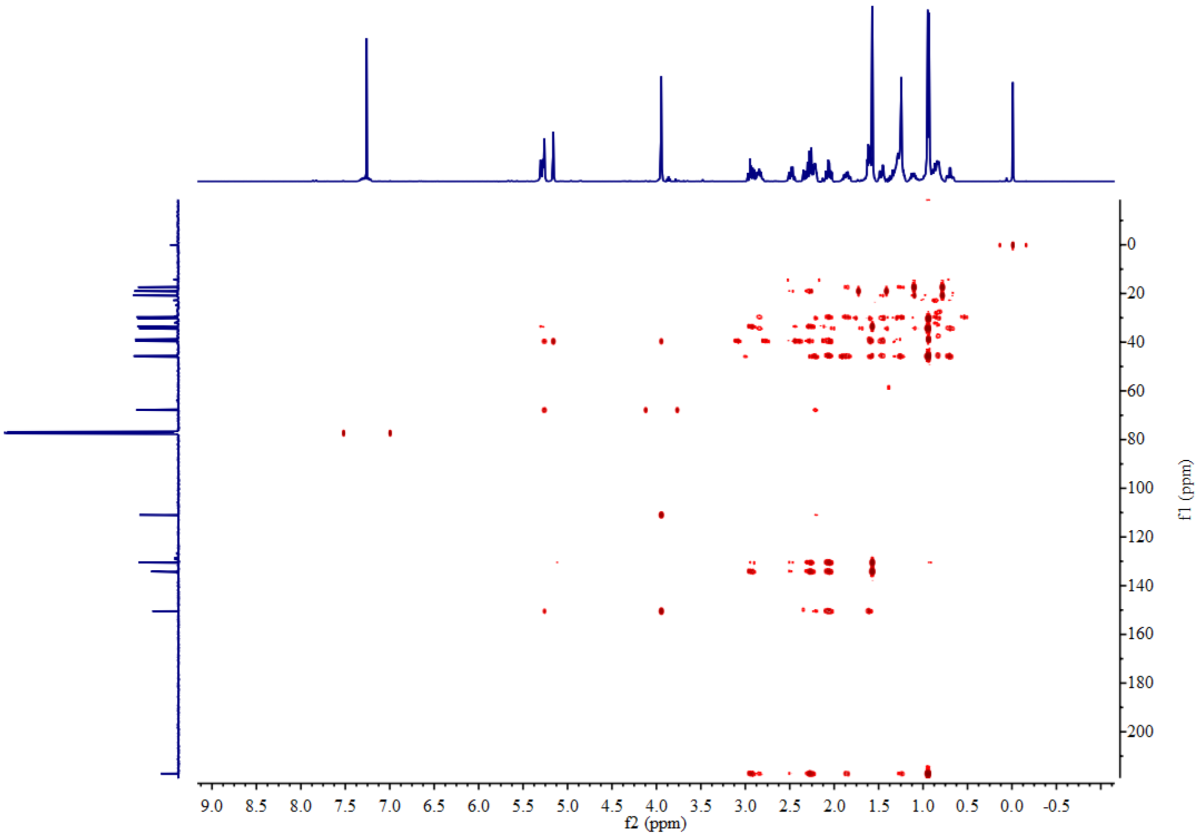

Figure S139 HMBC spectrum of **6b** in $CDCl_3$ (400 MHz).

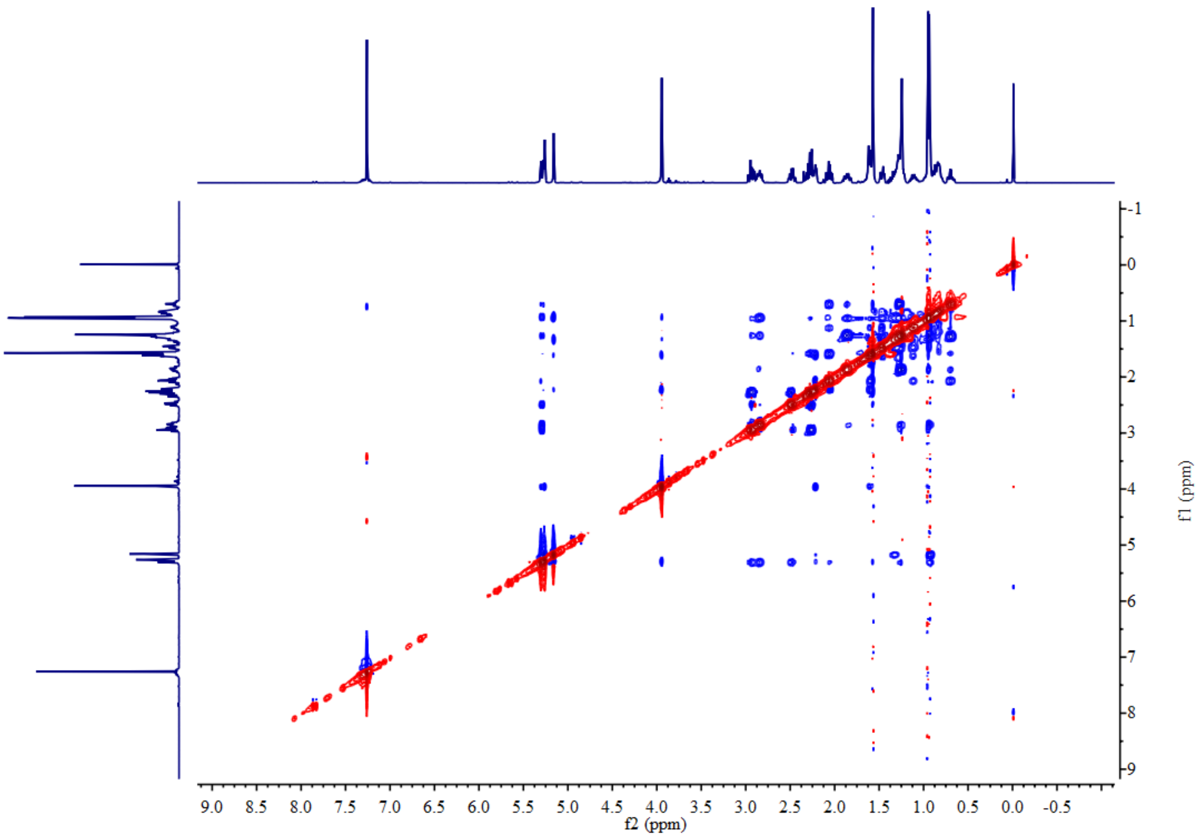

Figure S140 NOESY spectrum of **6b** in $CDCl_3$ (400 MHz).

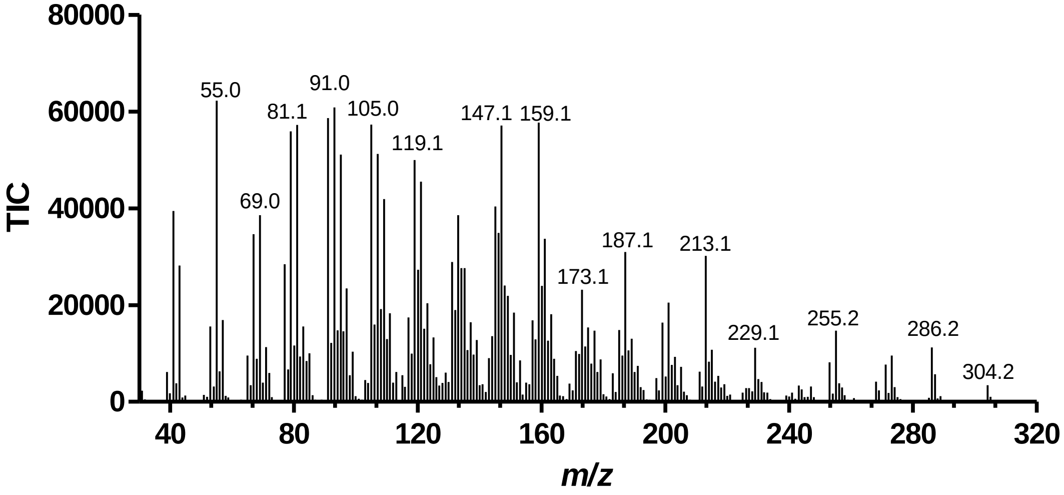

Figure S141 EI mass spectrum of **6b**.

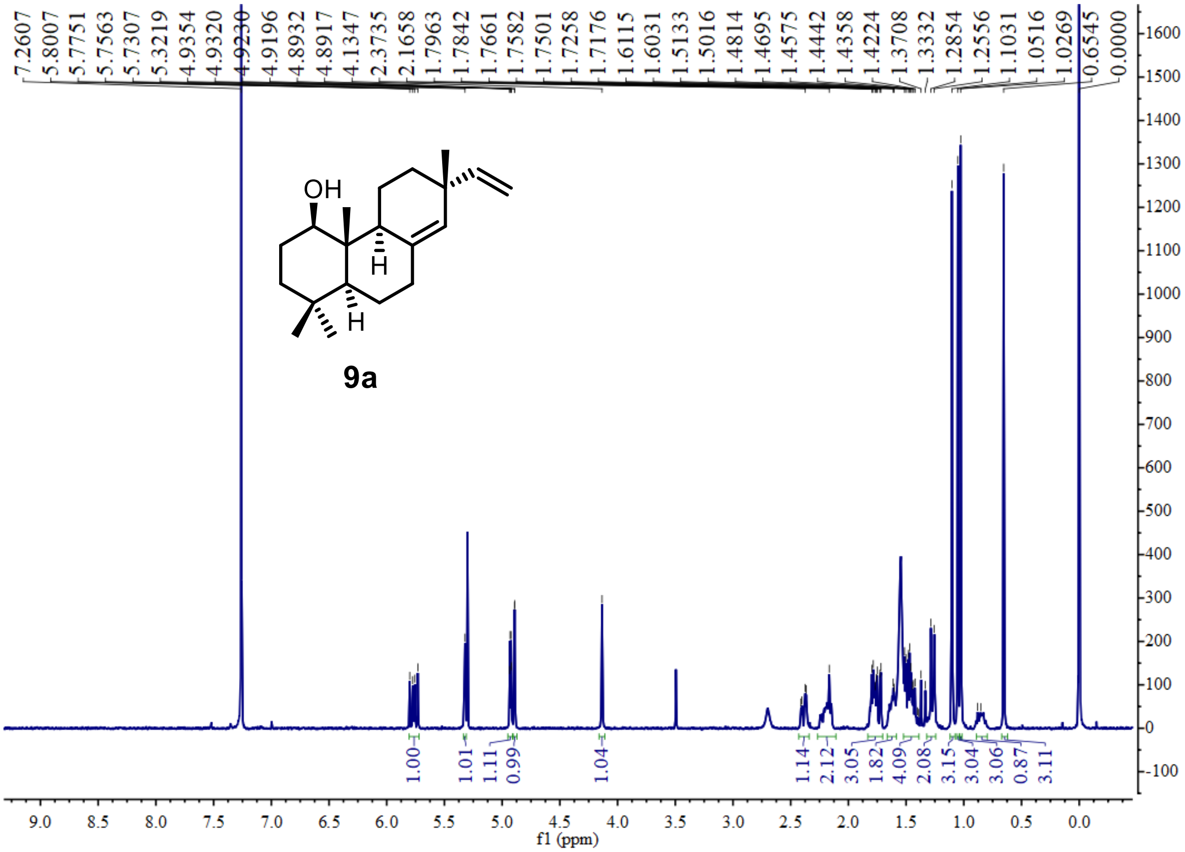

Figure S142 ^{1}H NMR spectrum of **9a** in $CDCl_3$ (400 MHz).

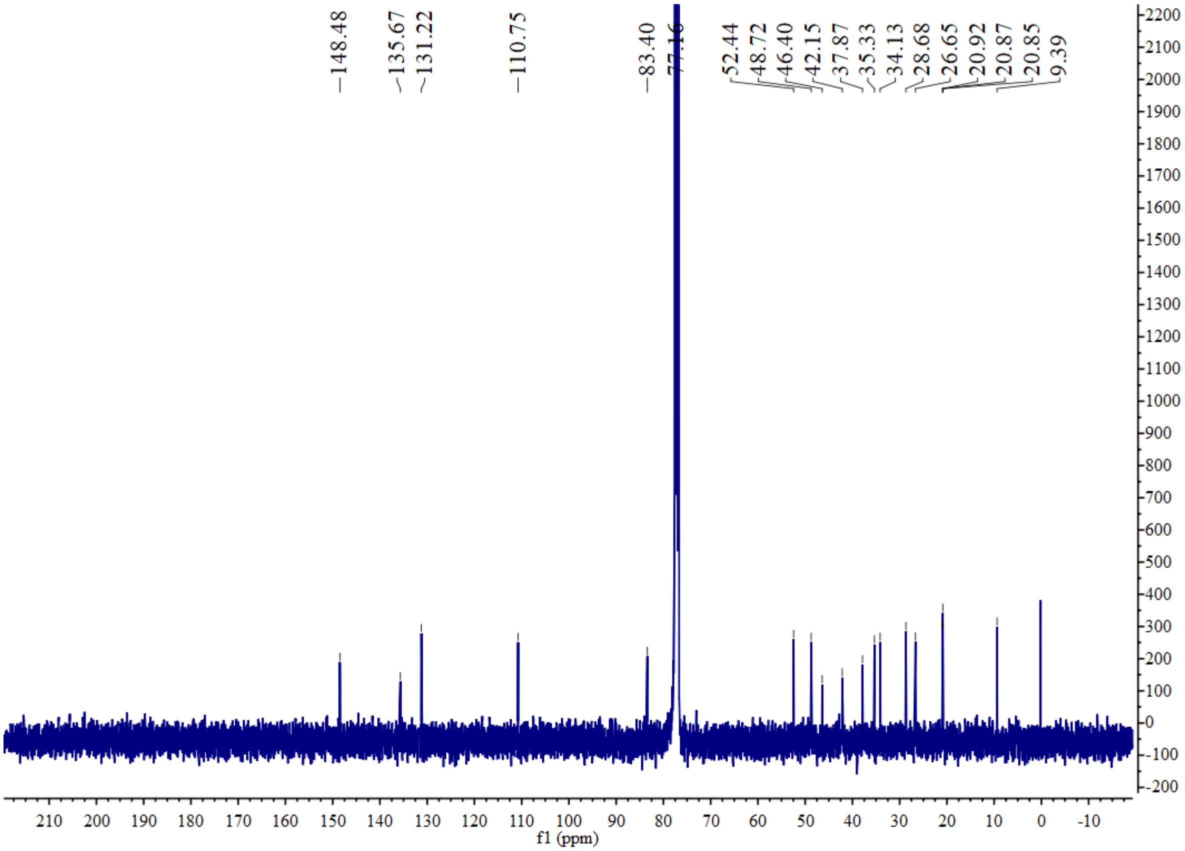

Figure S143 ^{13}C NMR spectrum of **9a** in $CDCl_3$ (100 MHz).

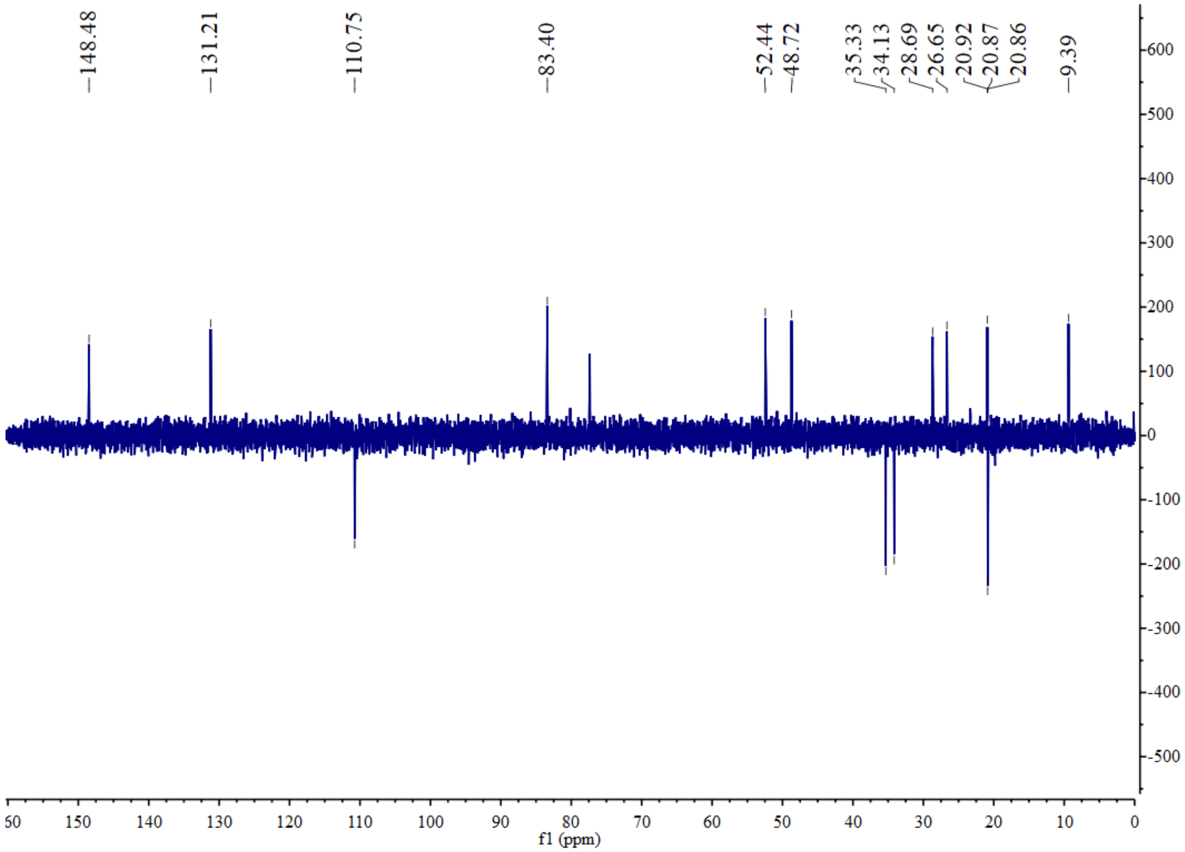

Figure S144 DEPT-135 NMR spectrum of **9a** in $CDCl_3$ (100 MHz).

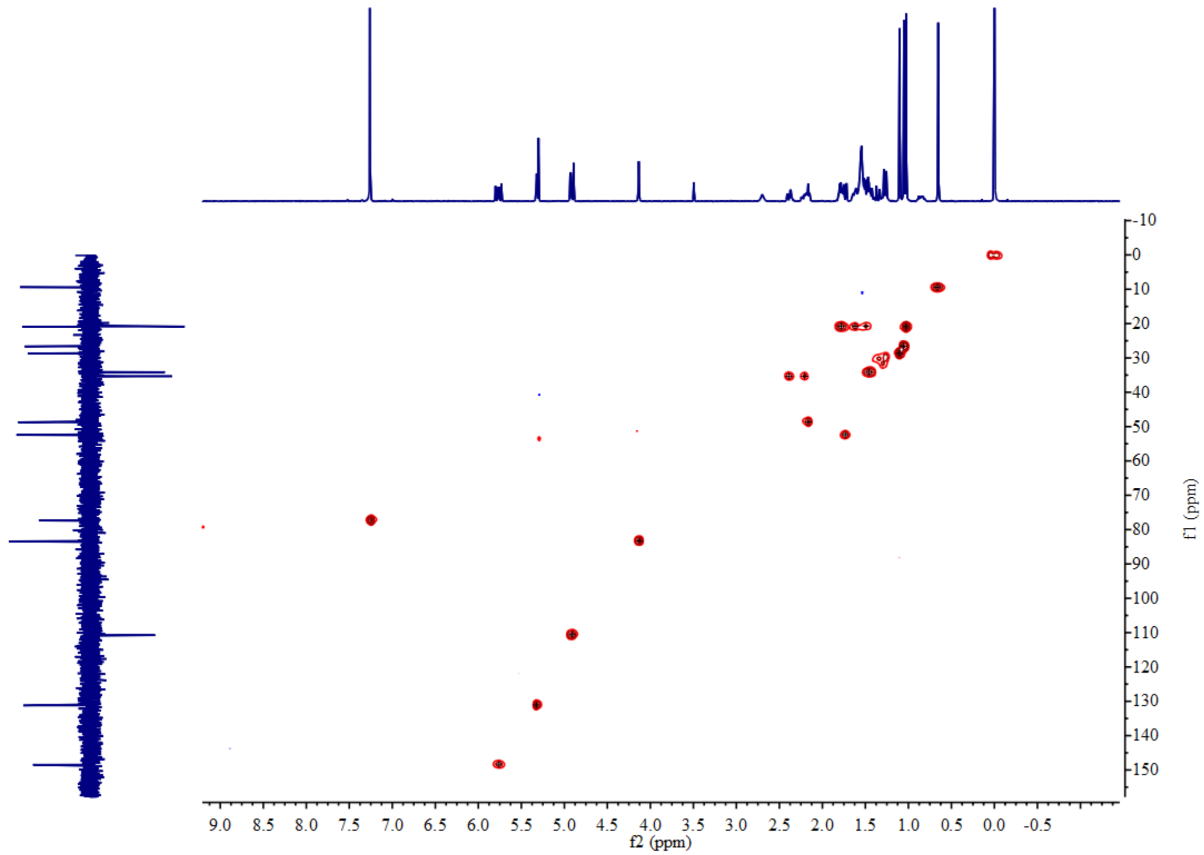

Figure S144 HSQC spectrum of **9a** in $CDCl_3$ (400 MHz).

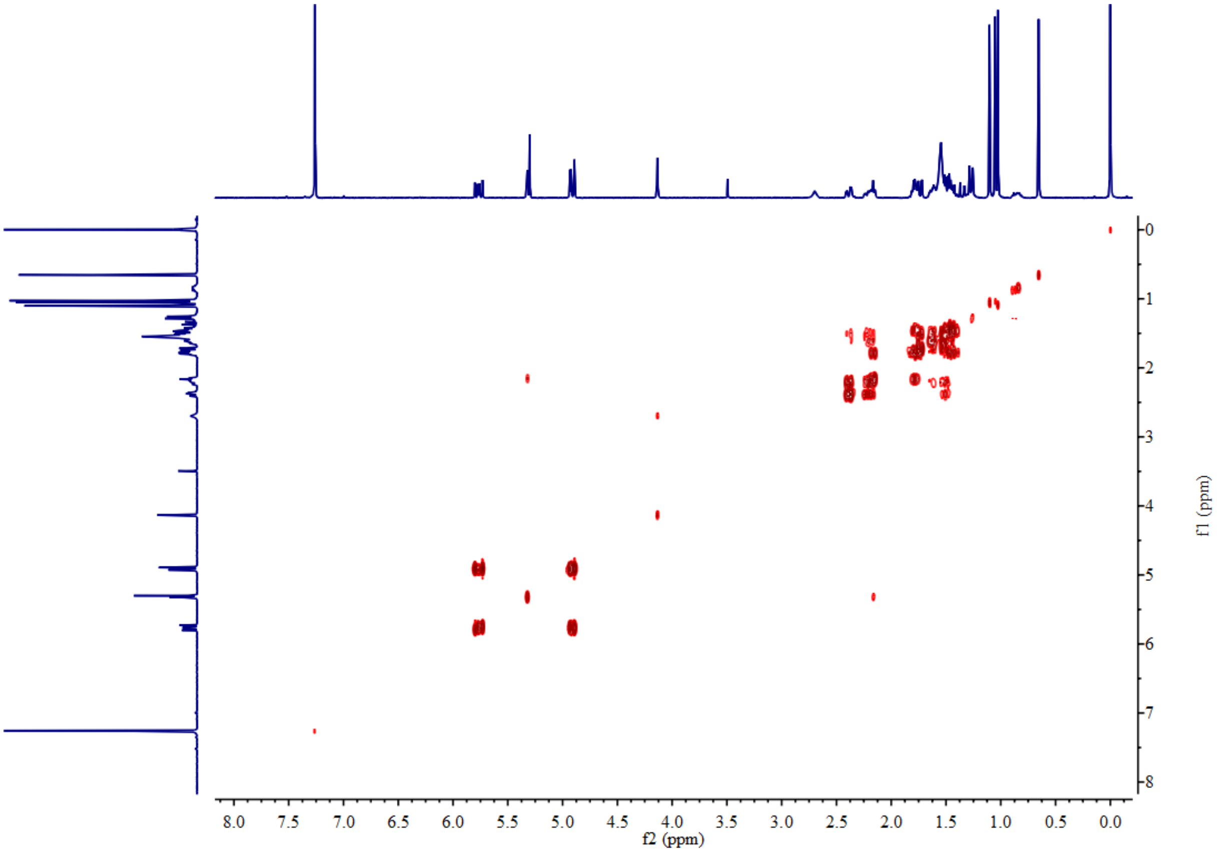

Figure S145 ^{1}H-^{1}H COSY spectrum of **9a** in $CDCl_3$ (400 MHz).

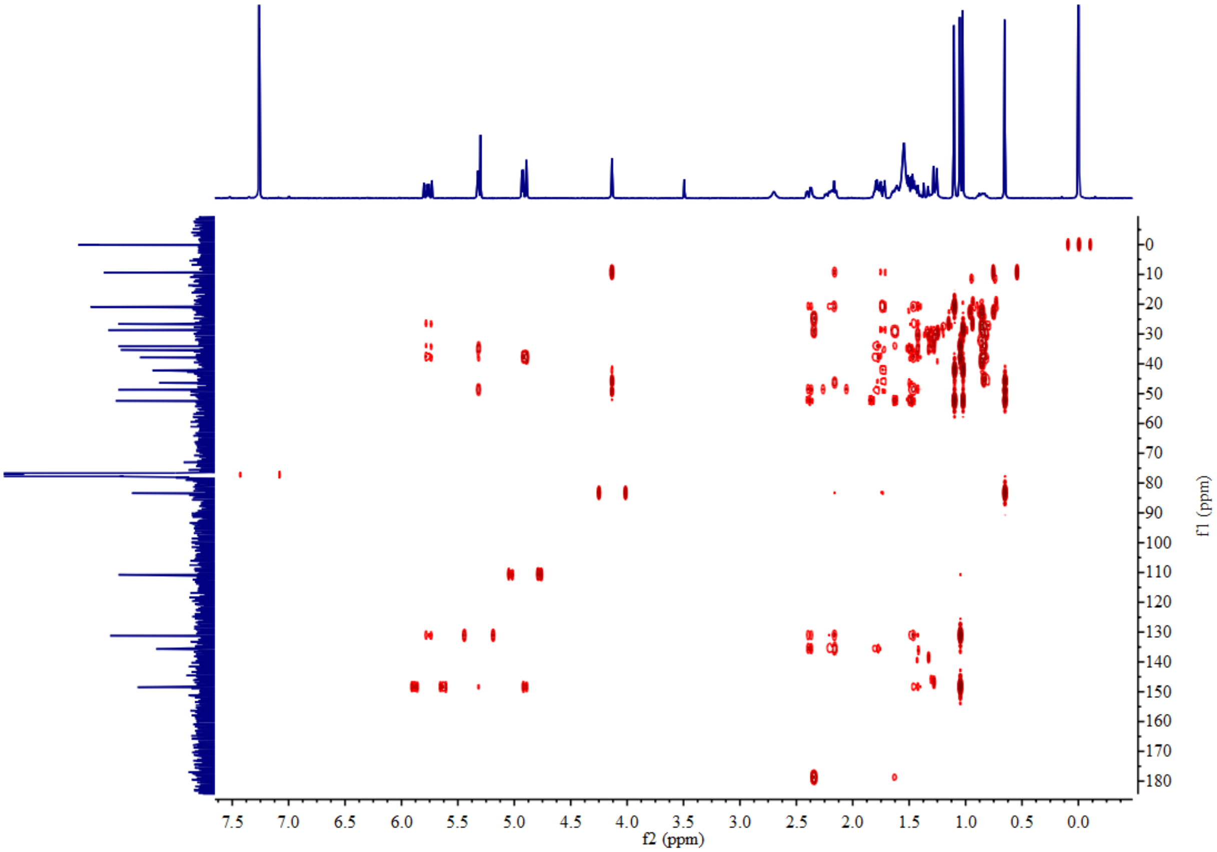

Figure S147 HMBC spectrum of **9a** in $CDCl_3$ (400 MHz).

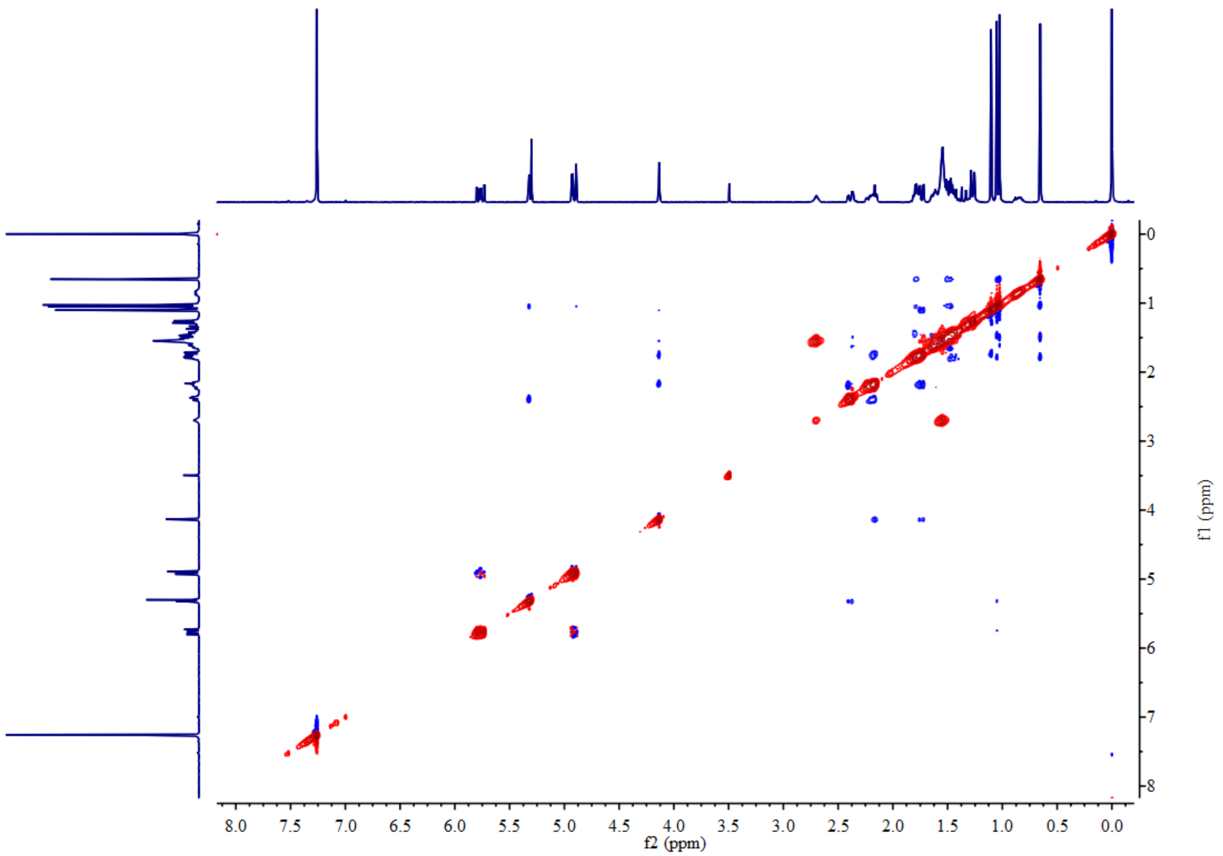

Figure S148 NOESY spectrum of **9a** in $CDCl_3$ (400 MHz).

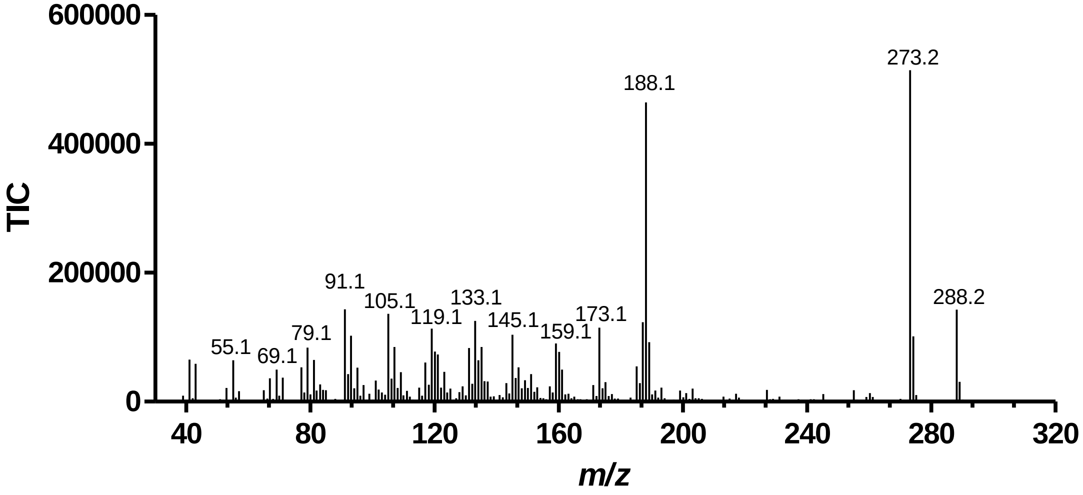

Figure S149 EI mass spectrum of **9a**.

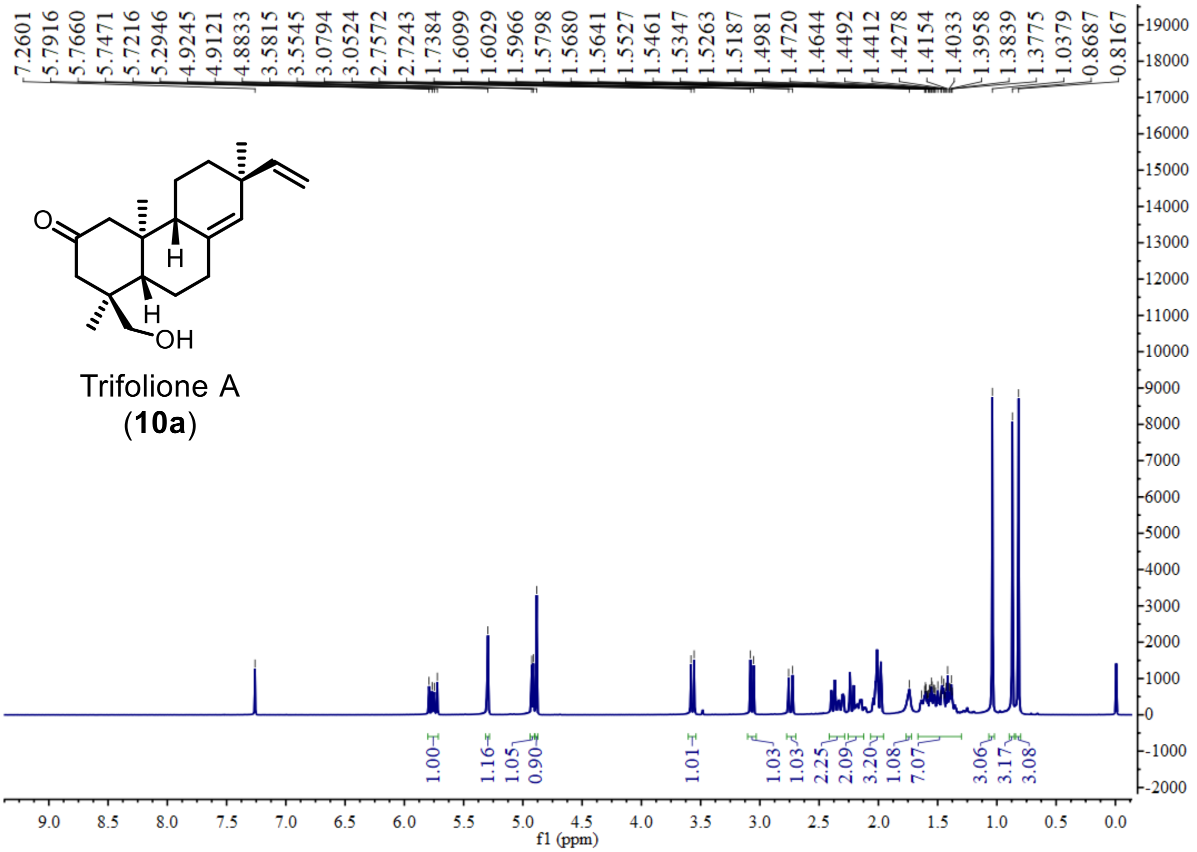

Figure S150 ^{1}H NMR spectrum of **10a** in $CDCl_3$ (400 MHz).

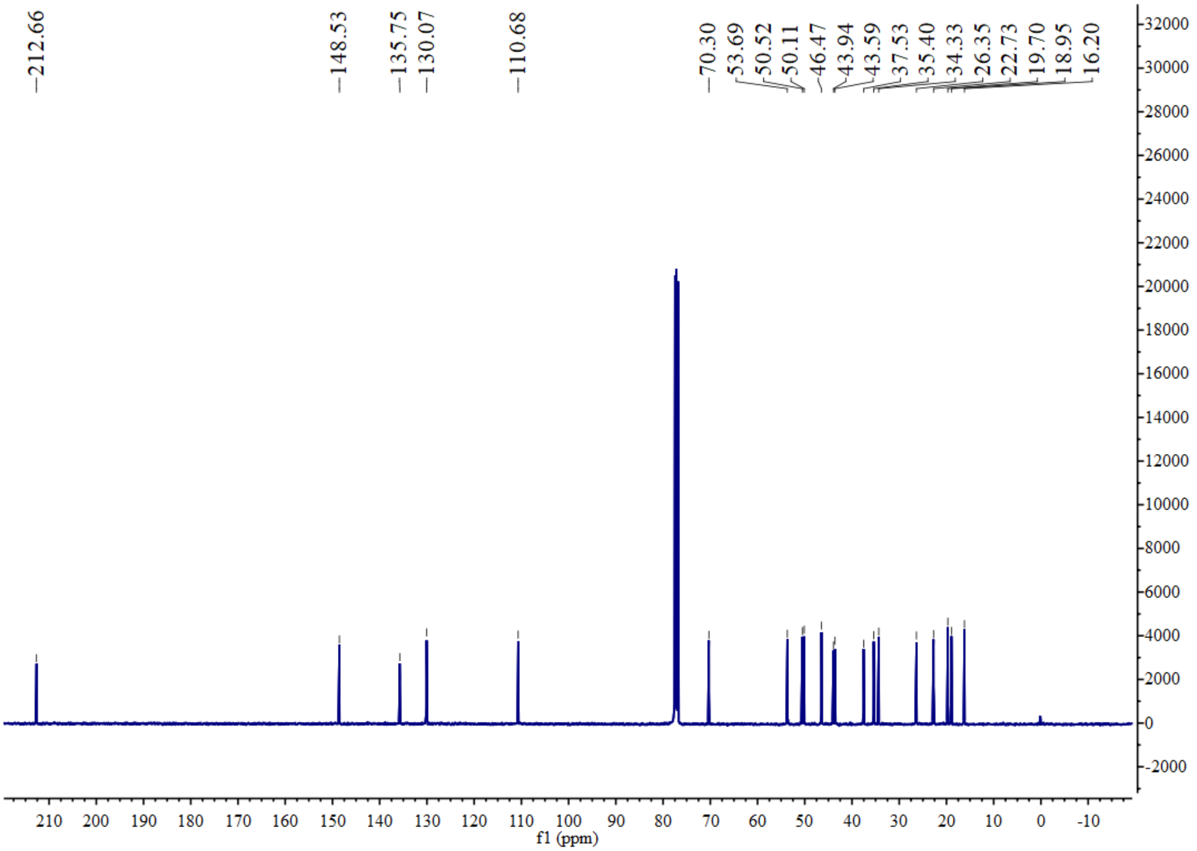

Figure S151 ^{13}C NMR spectrum of **10a** in $CDCl_3$ (100 MHz).

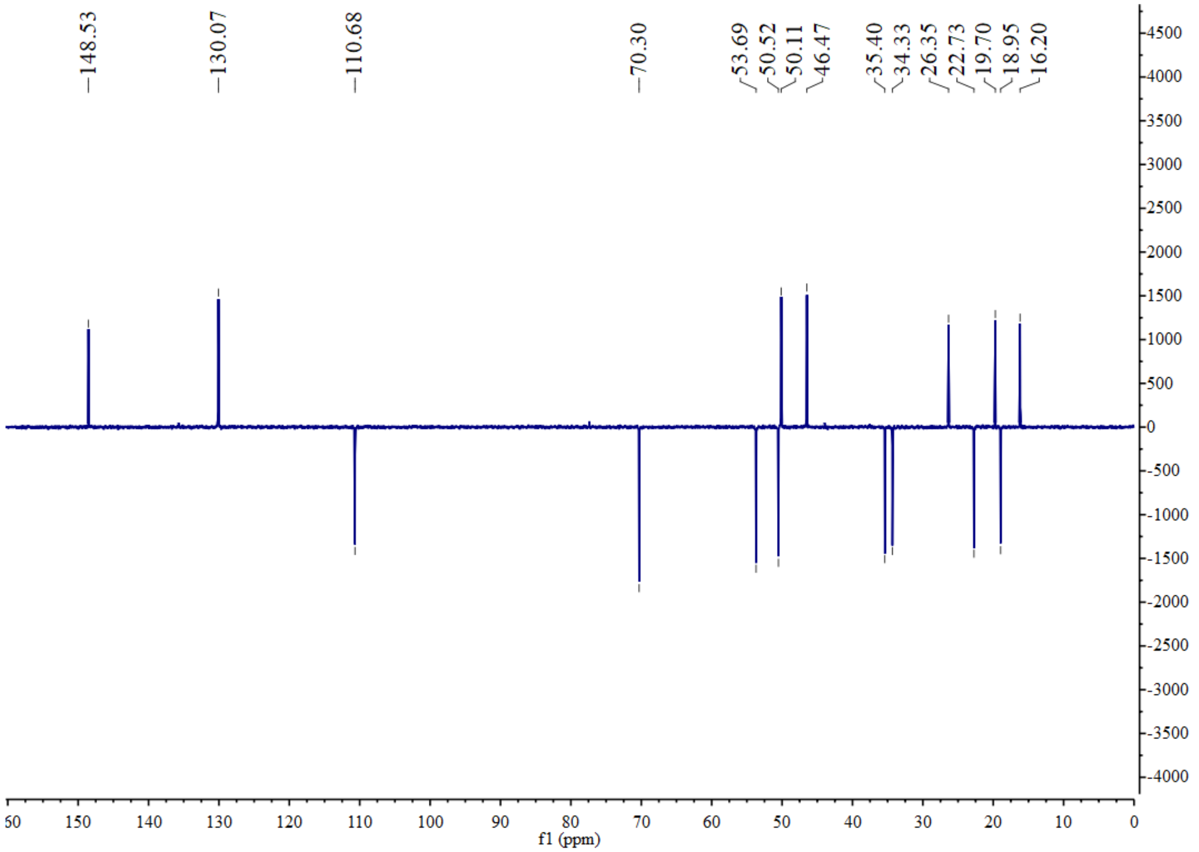

Figure S152 DEPT-135 NMR spectrum of **10a** in $CDCl_3$ (100 MHz).

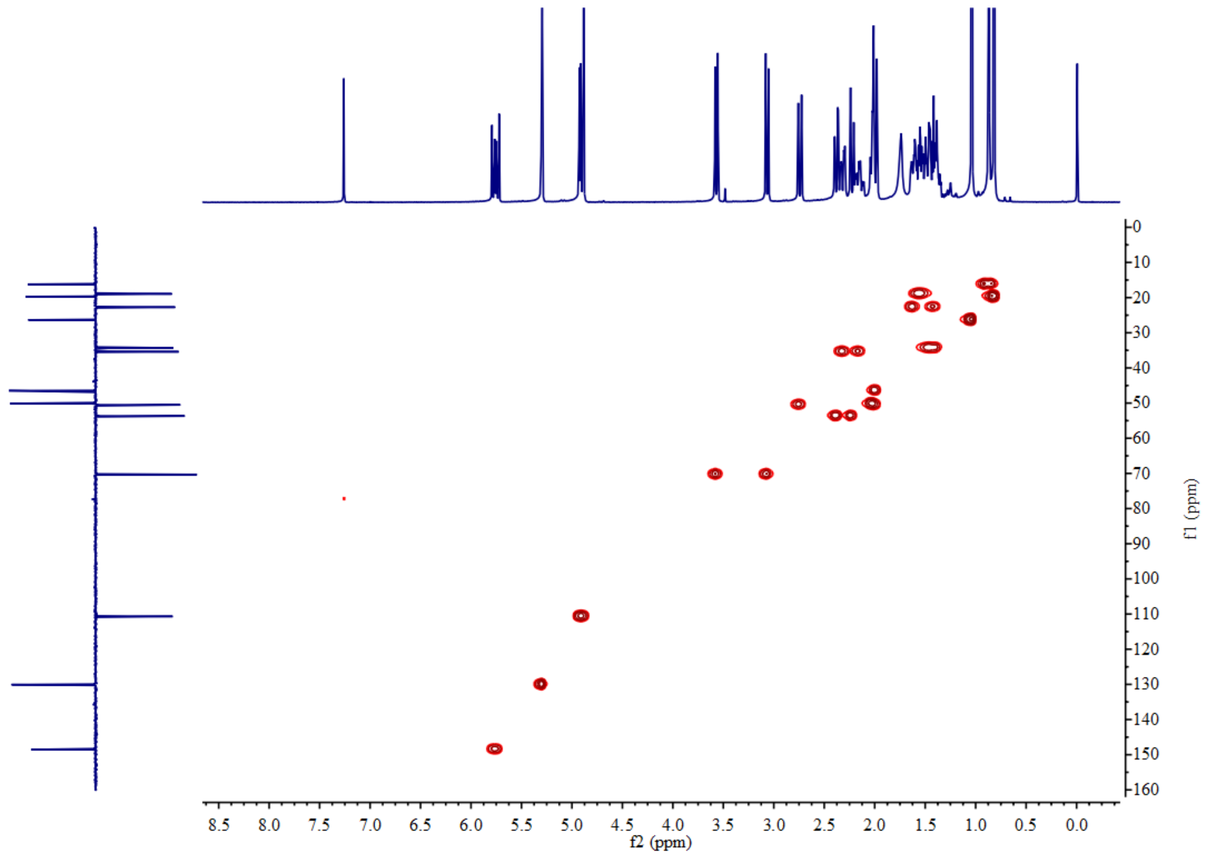

Figure S153 HSQC spectrum of **10a** in $CDCl_3$ (400 MHz).

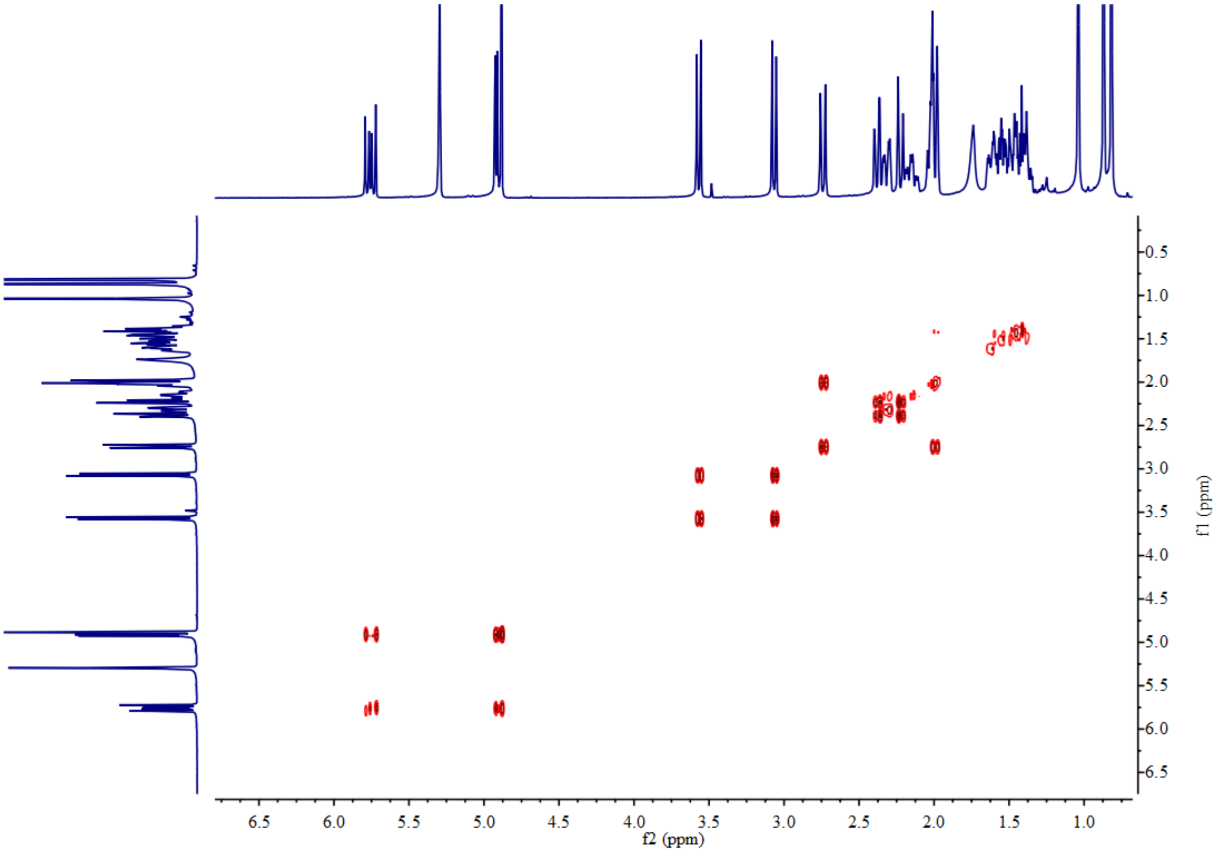

Figure S154 ^{1}H-^{1}H COSY spectrum of **10a** in $CDCl_3$ (400 MHz).

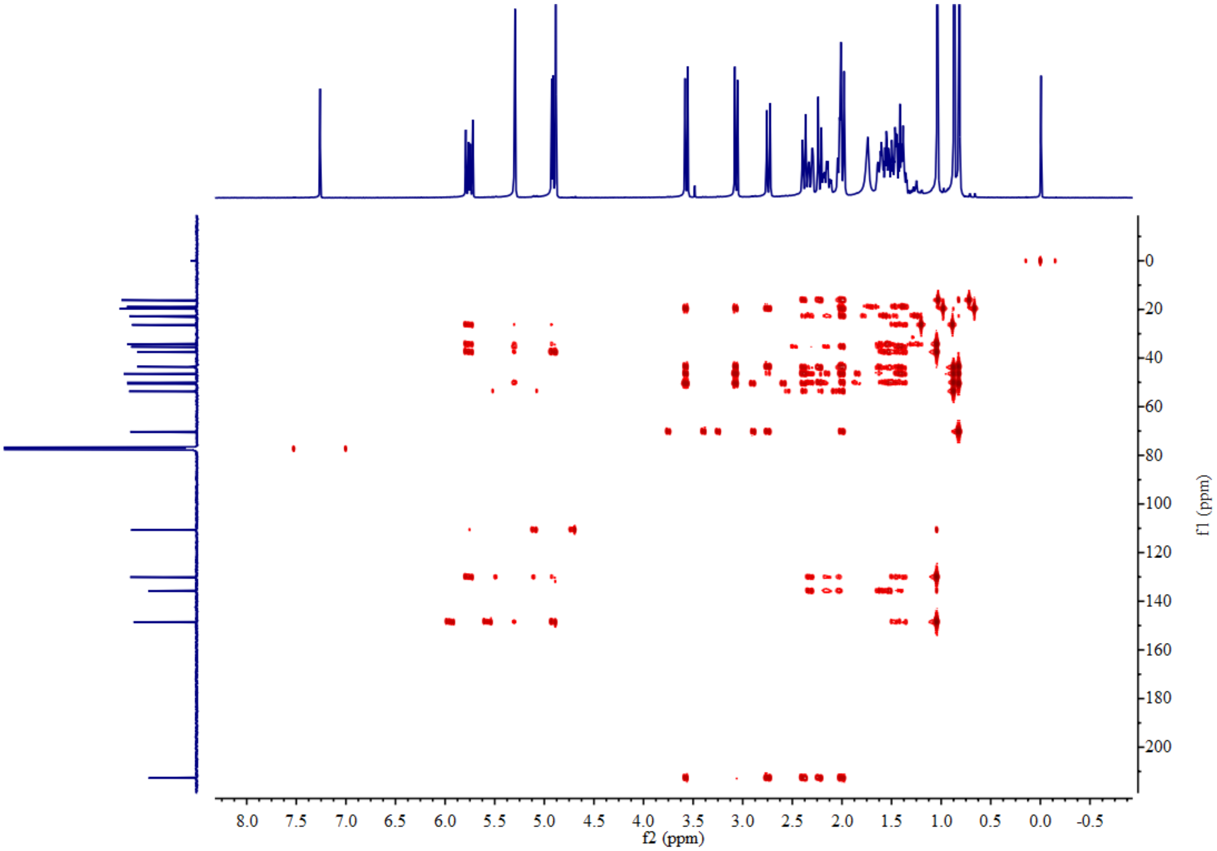

Figure S155 HMBC spectrum of **10a** in $CDCl_3$ (400 MHz).

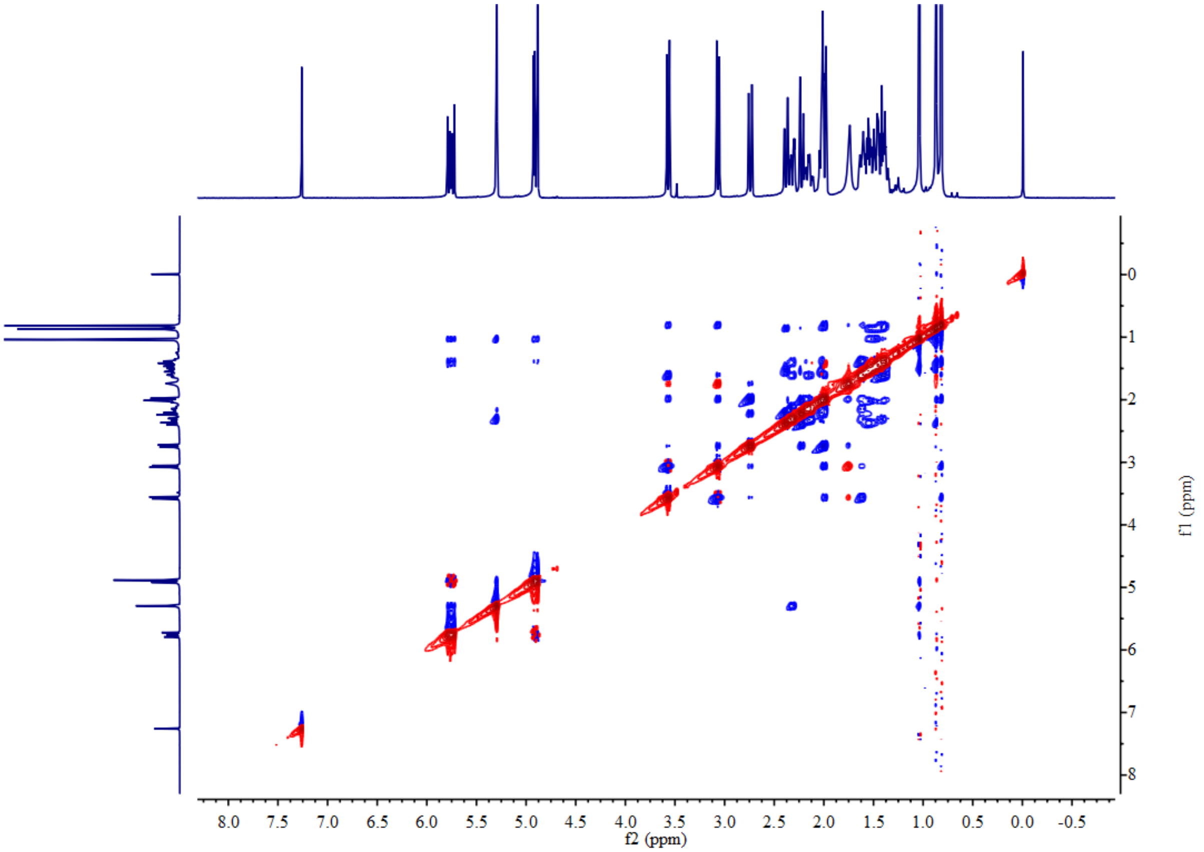

Figure S156 NOESY spectrum of **10a** in $CDCl_3$ (400 MHz).

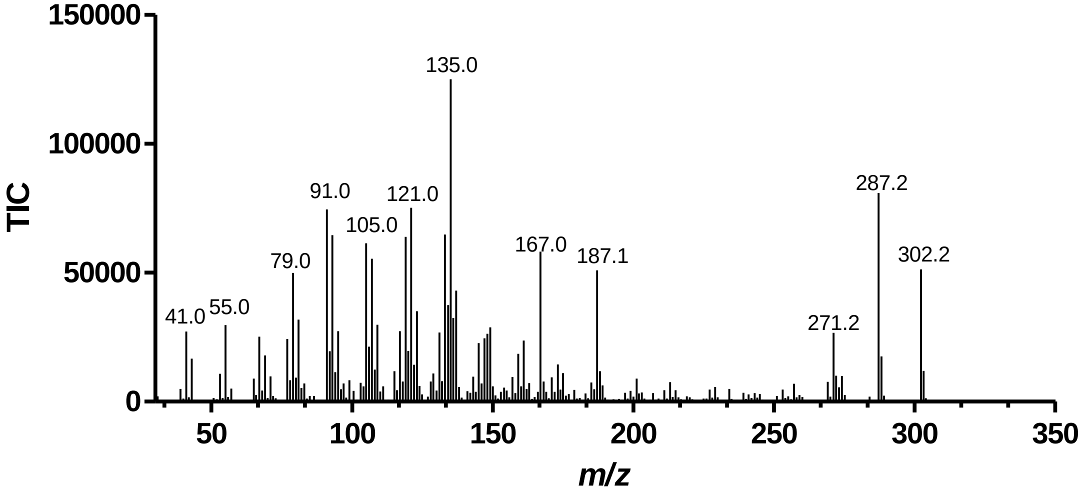

Figure S157 EI mass spectrum of **10a**.

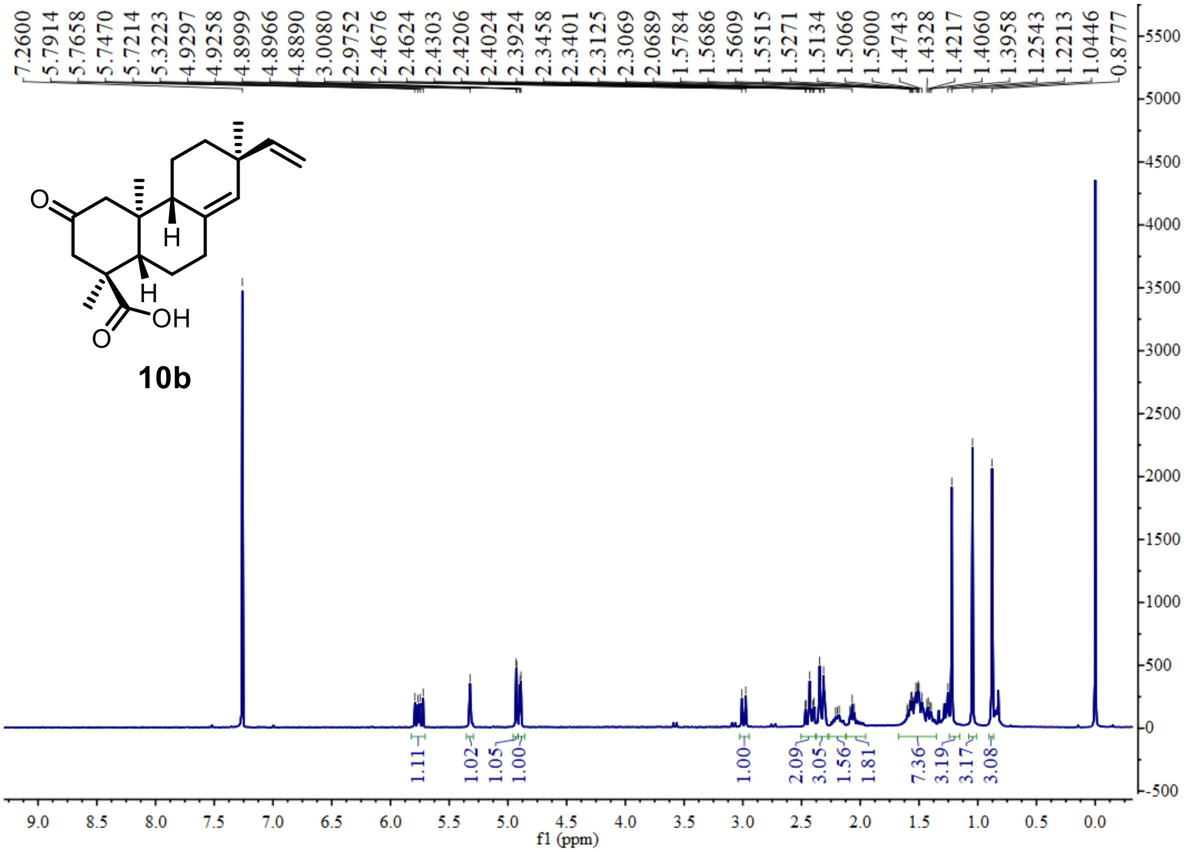

Figure S158 ^{1}H NMR spectrum of **10b** in $CDCl_3$ (400 MHz).

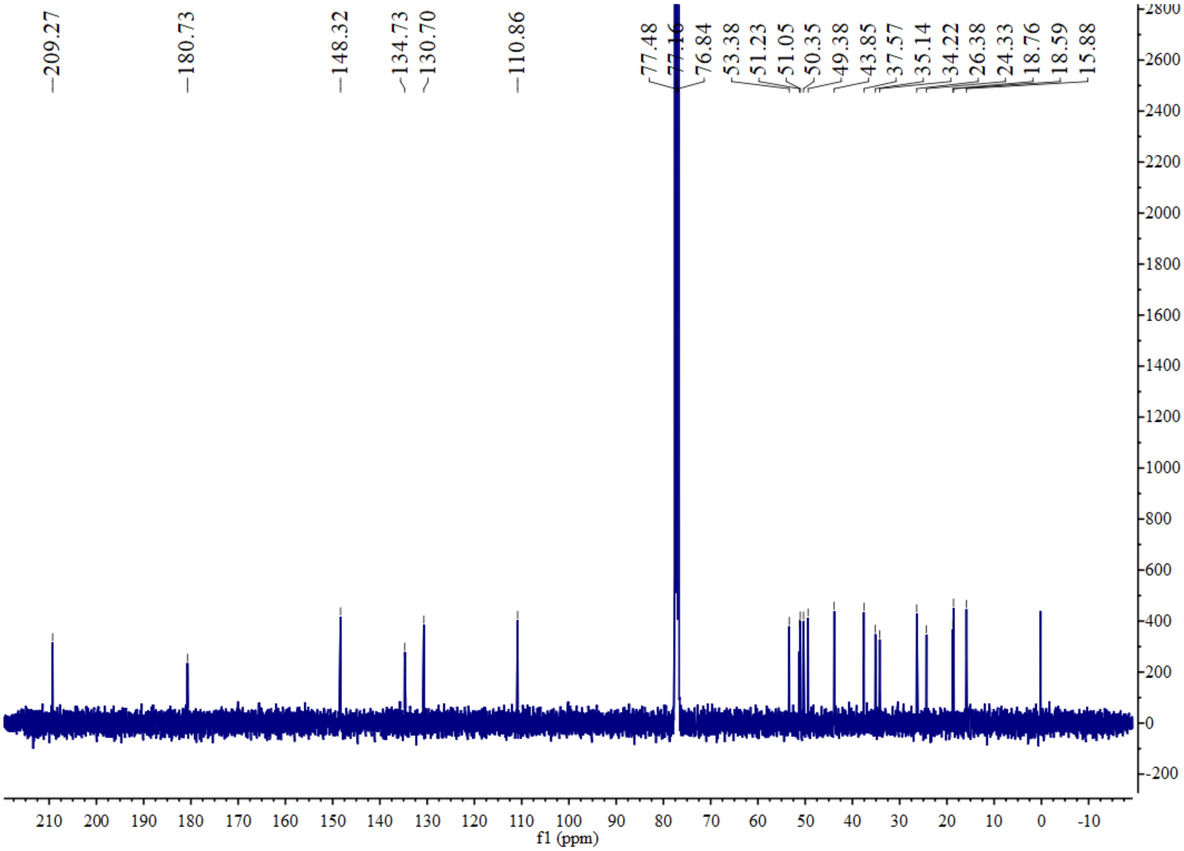

Figure S159^{13}C NMR spectrum of **10b** in $CDCl_3$ (100 MHz).

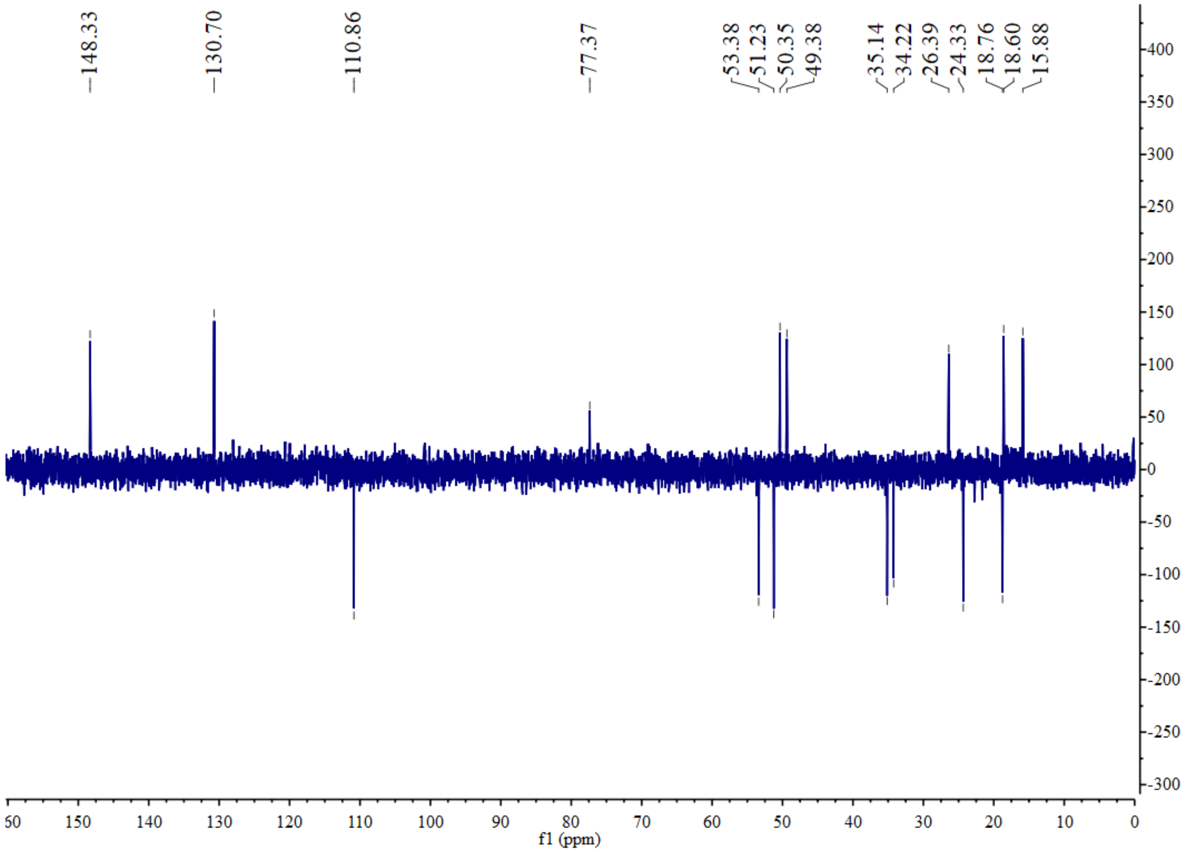

Figure S160 DEPT-135 NMR spectrum of **10b** in $CDCl_3$ (100 MHz).

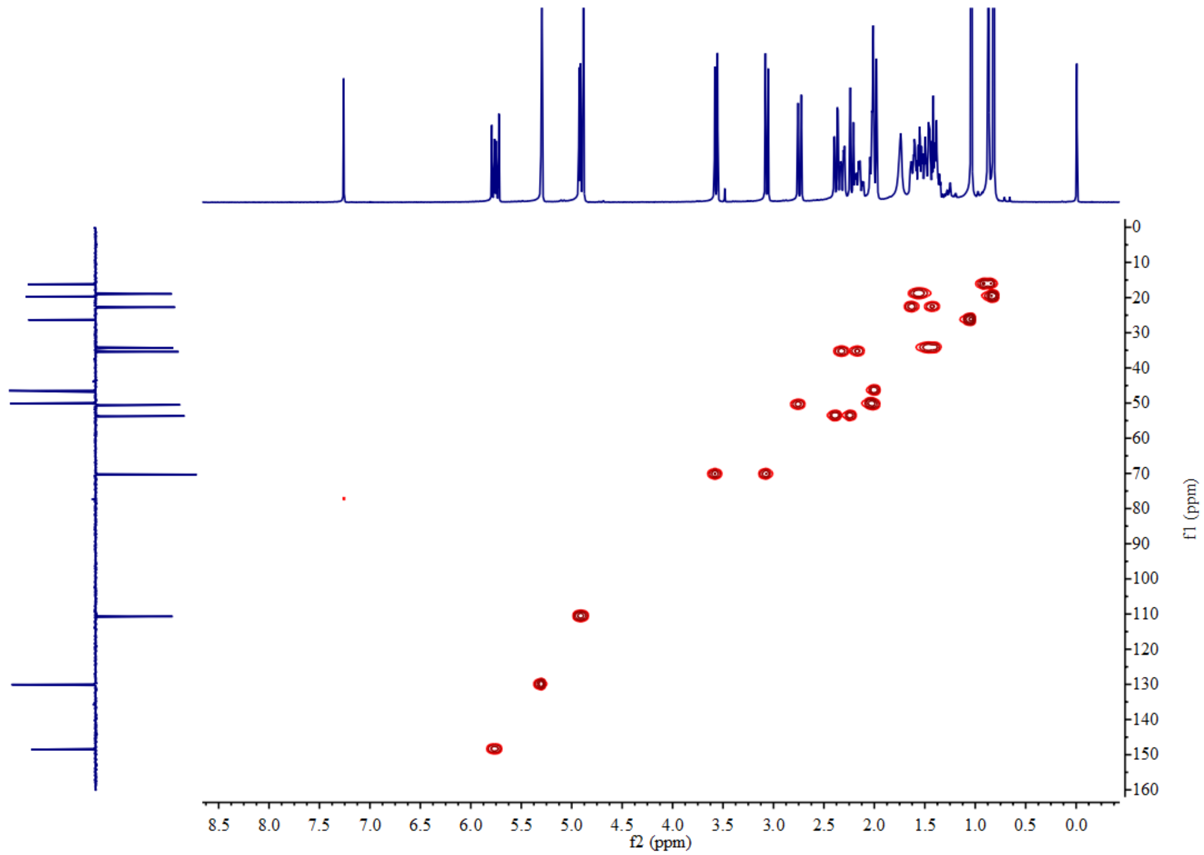

Figure S161 HSQC spectrum of **10b** in $CDCl_3$ (400 MHz).

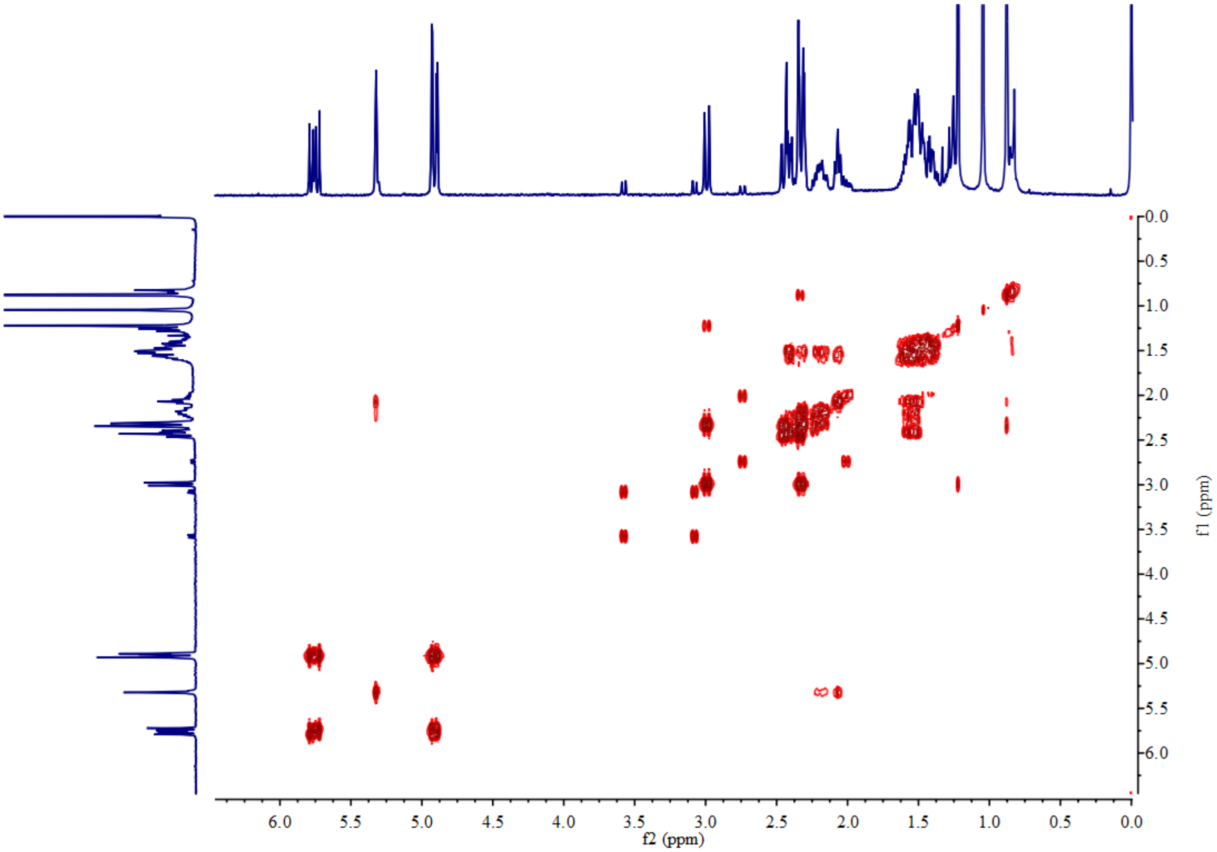

Figure S162 ^{1}H-^{1}H COSY spectrum of **10b** in $CDCl_3$ (400 MHz).

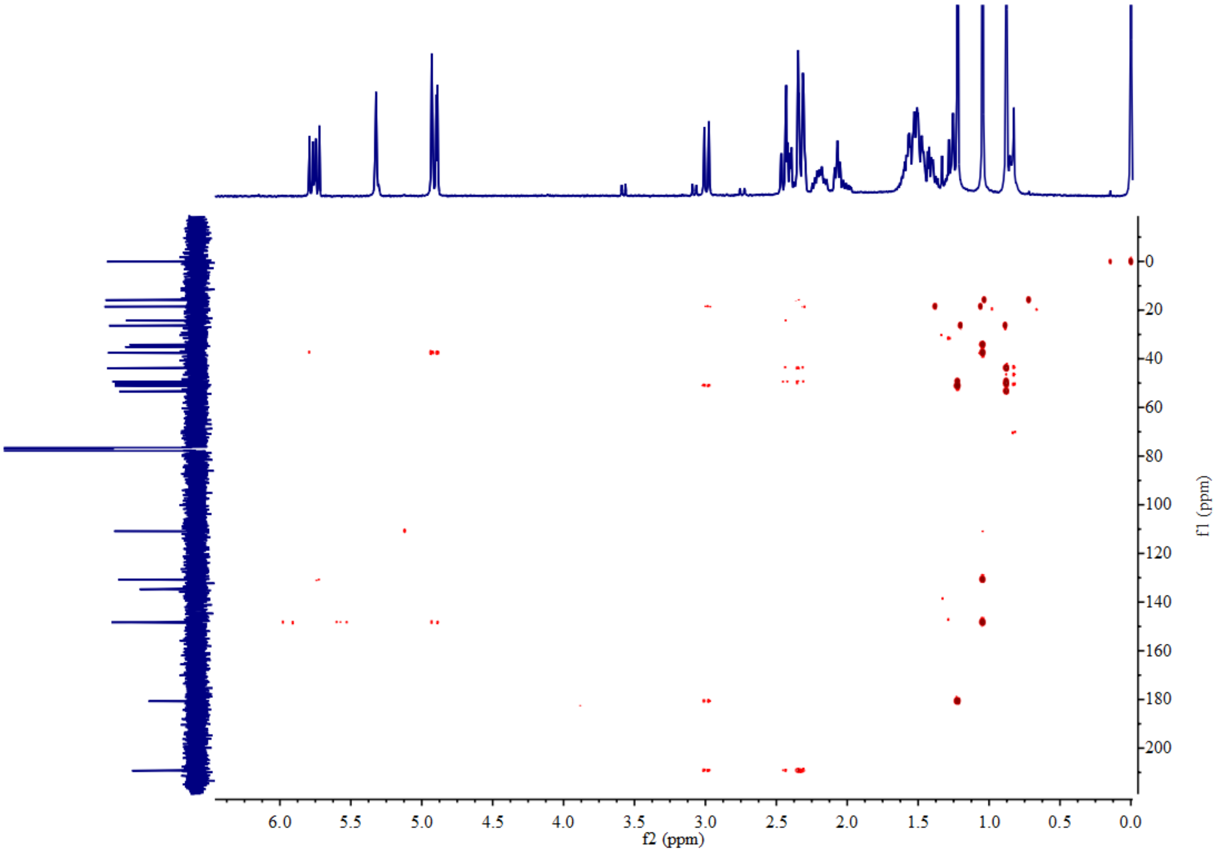

Figure S163 HMBC spectrum of **10b** in $CDCl_3$ (400 MHz).

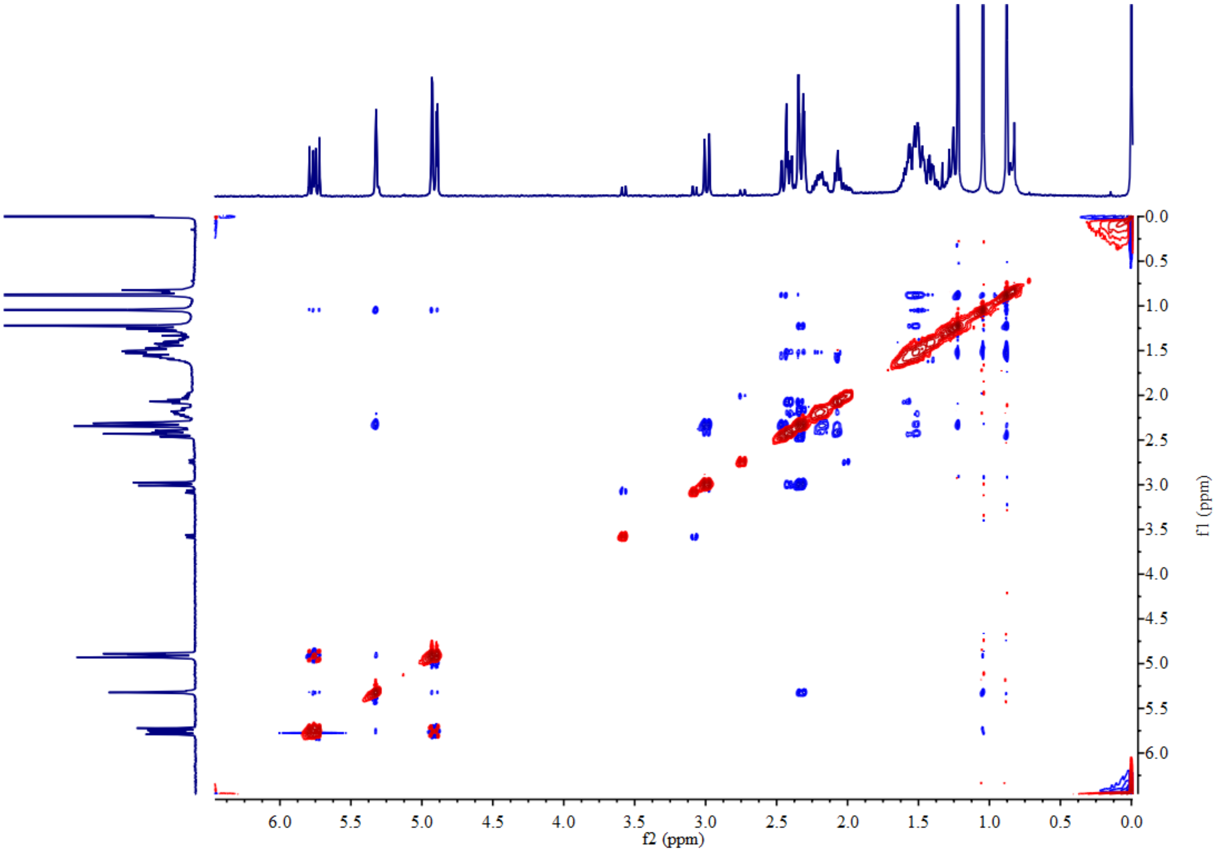

Figure S164 NOESY spectrum of **10b** in $CDCl_3$ (400 MHz).

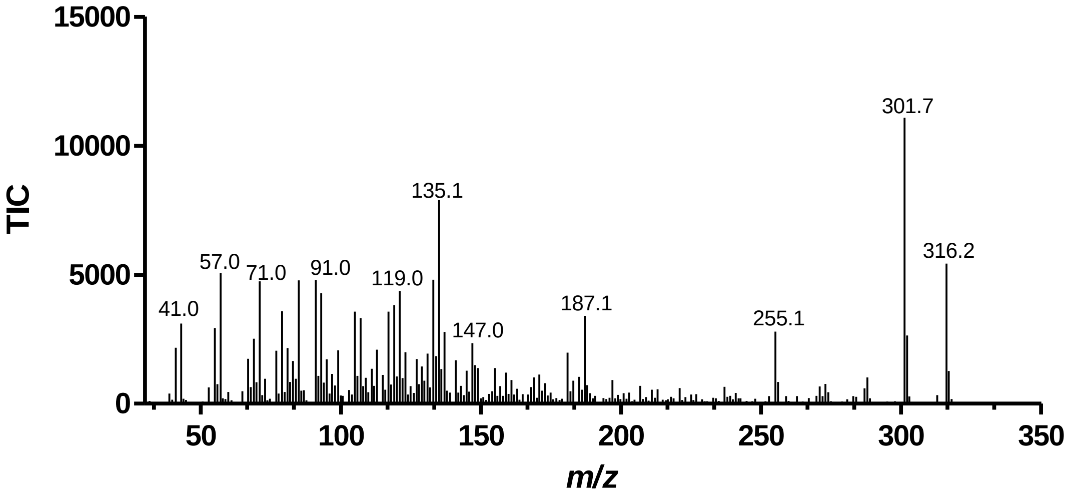

Figure S165 EI mass spectrum of **10b**.

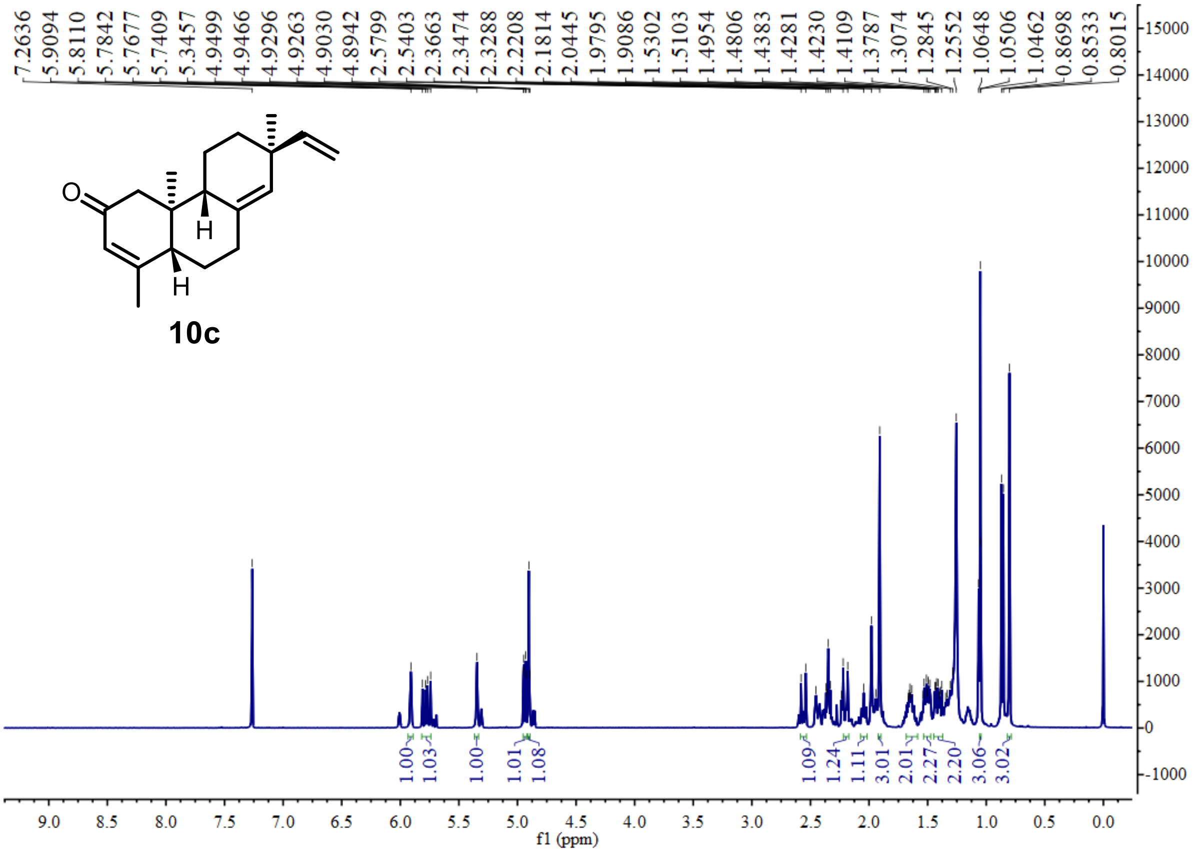

Figure S166 ^{1}H NMR spectrum of **10c** in $CDCl_3$ (400 MHz).

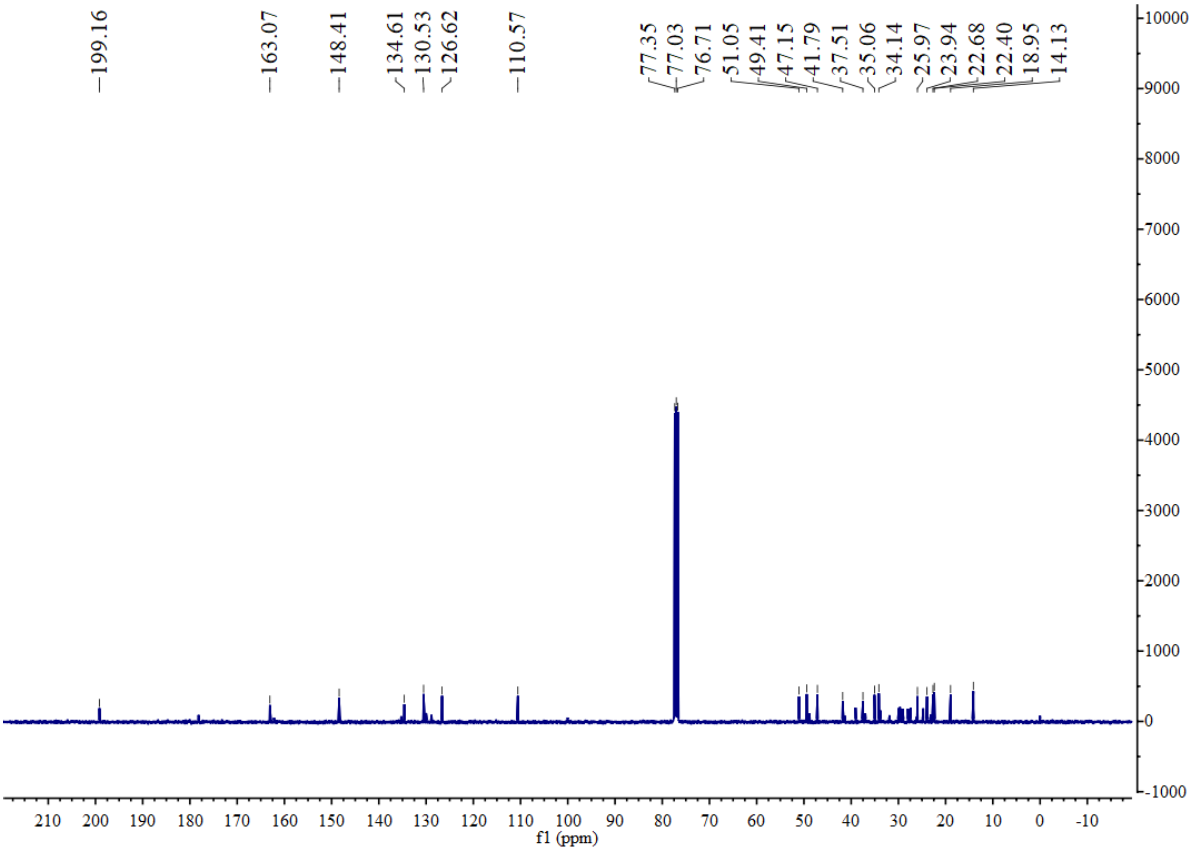

Figure S167 ^{13}C NMR spectrum of **10c** in $CDCl_3$ (100 MHz).

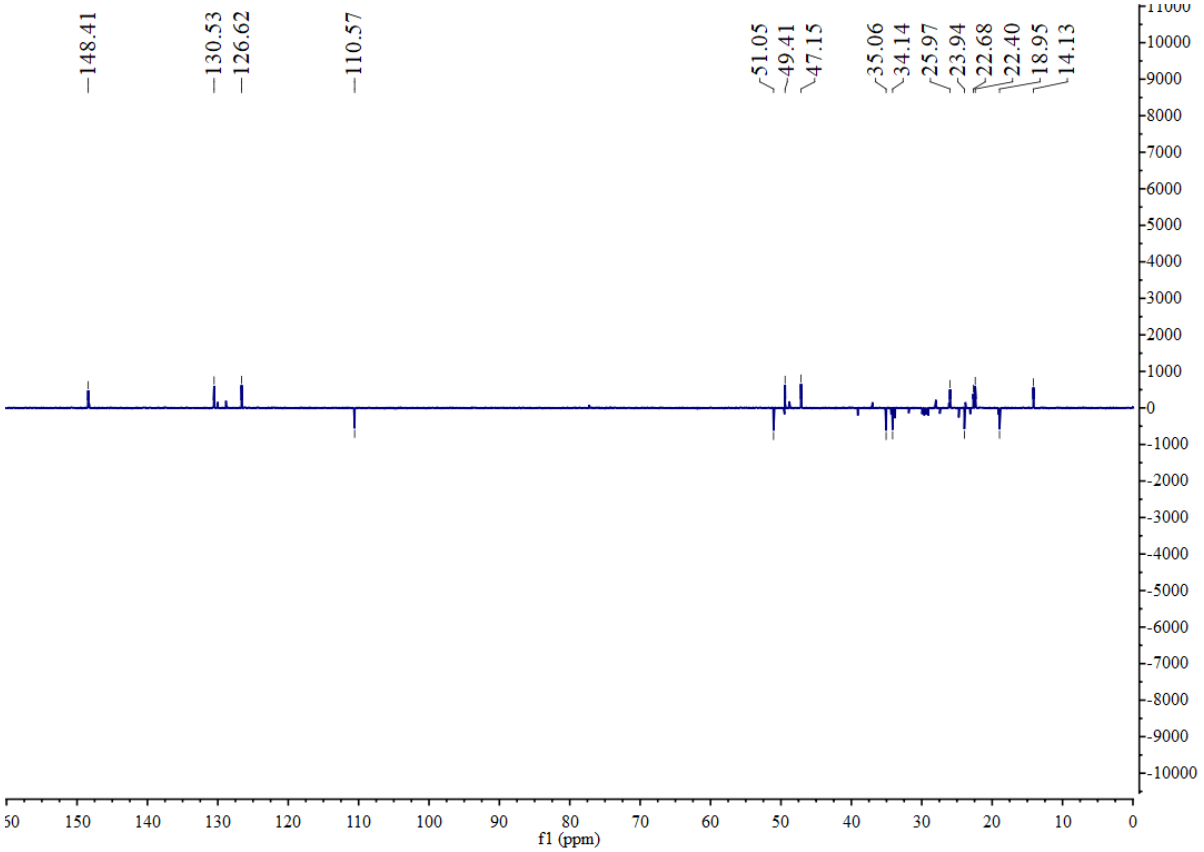

Figure S168 DEPT-135 NMR spectrum of **10c** in $CDCl_3$ (100 MHz).

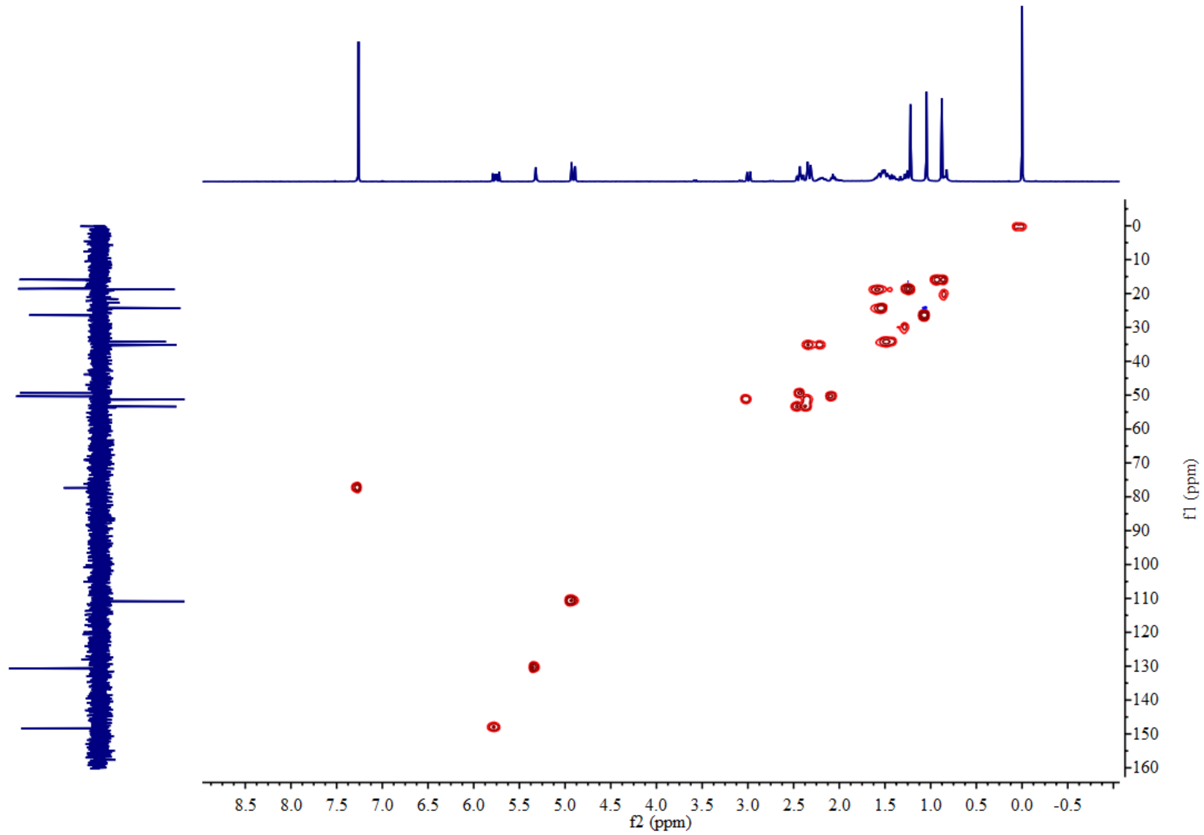

Figure S169 HSQC spectrum of **10c** in $CDCl_3$ (400 MHz).

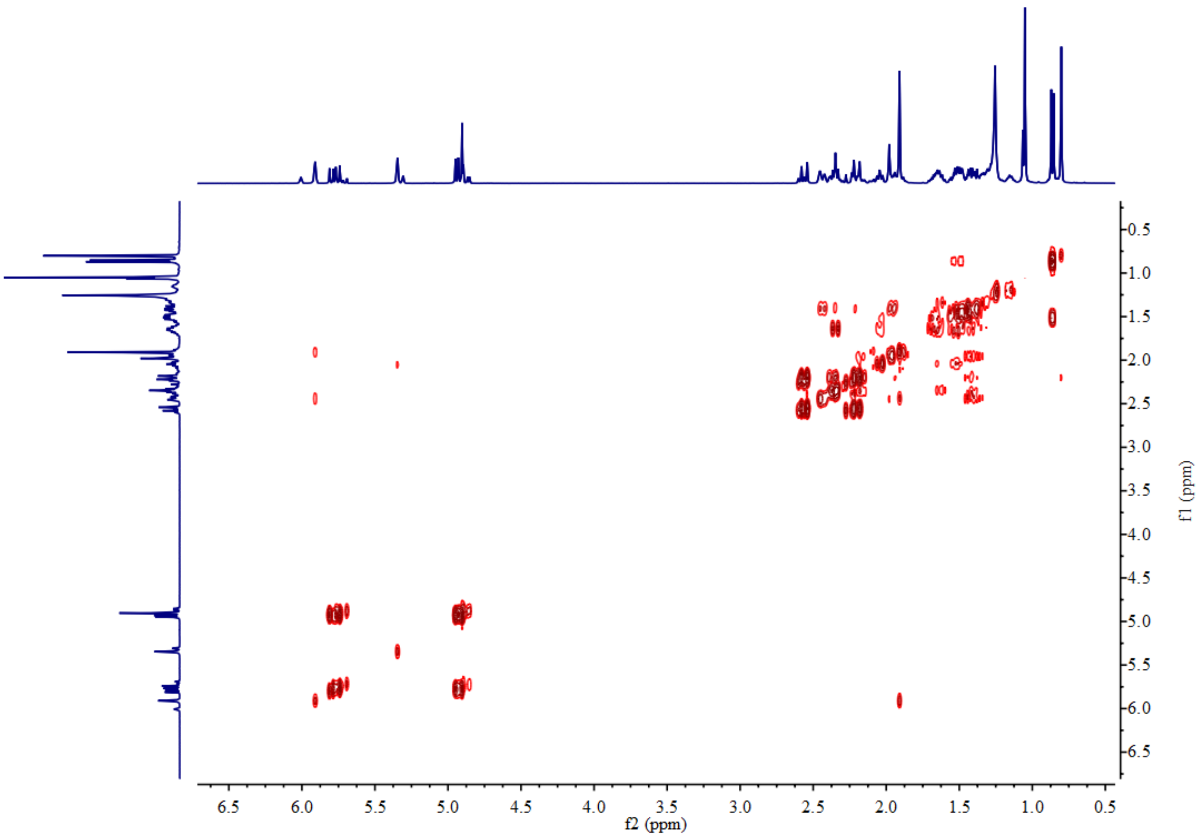

Figure S170 ^{1}H-^{1}H COSY spectrum of **10c** in $CDCl_3$ (400 MHz).

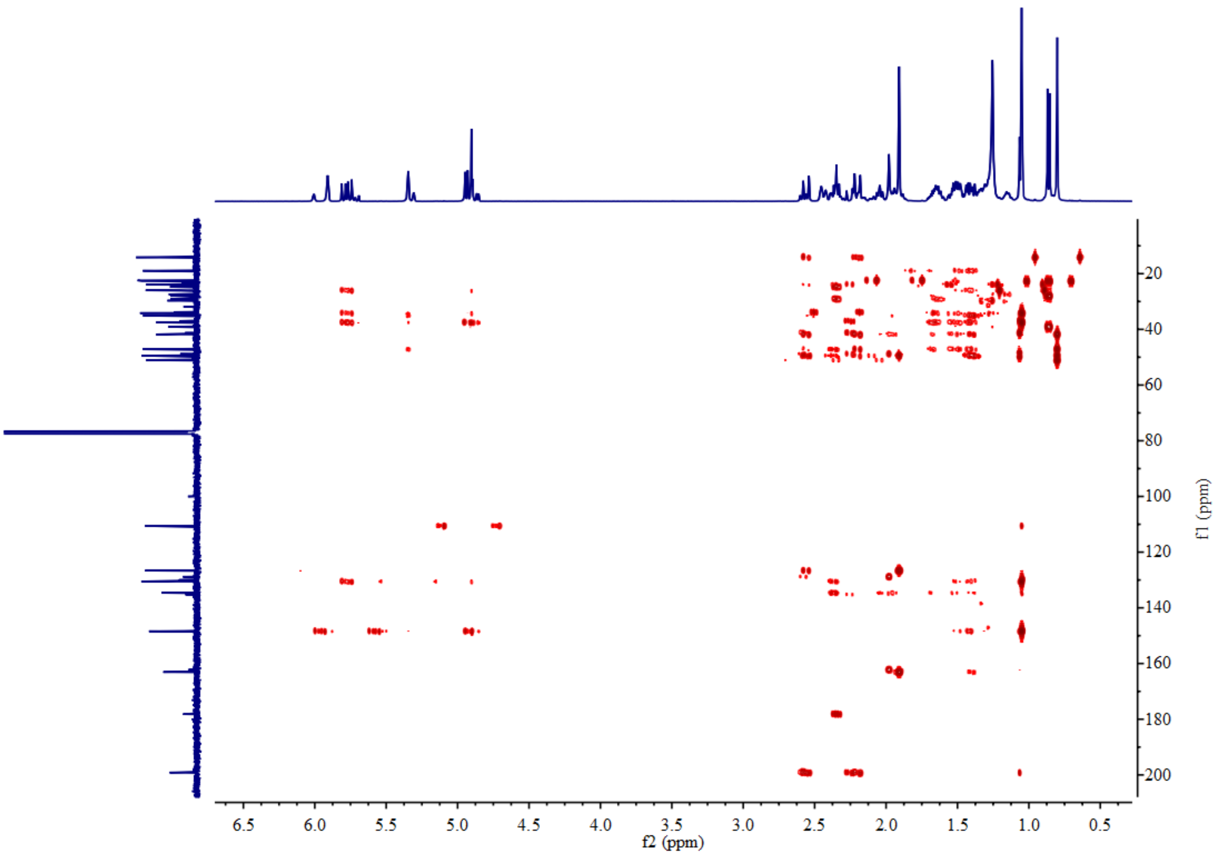

Figure S171 HMBC spectrum of **10c** in $CDCl_3$ (400 MHz).

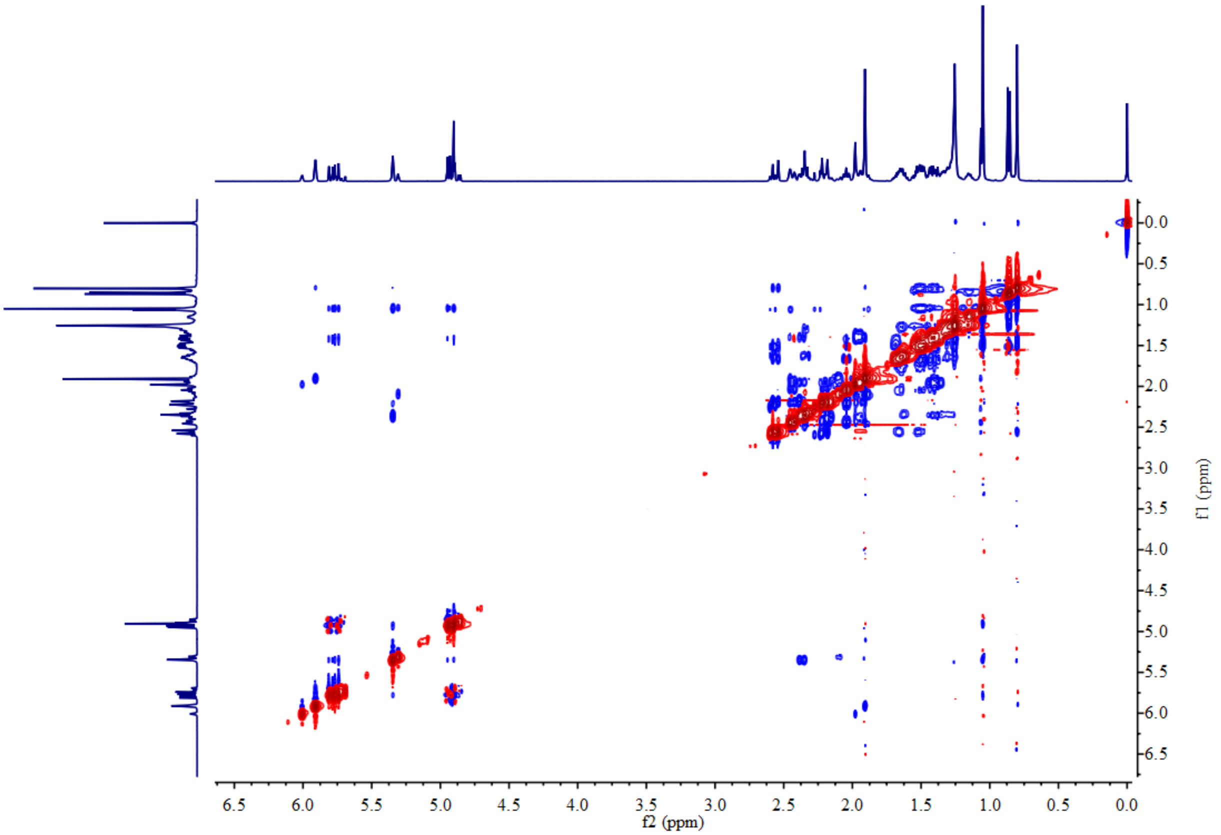

Figure S172 NOESY spectrum of **10c** in $CDCl_3$ (400 MHz).

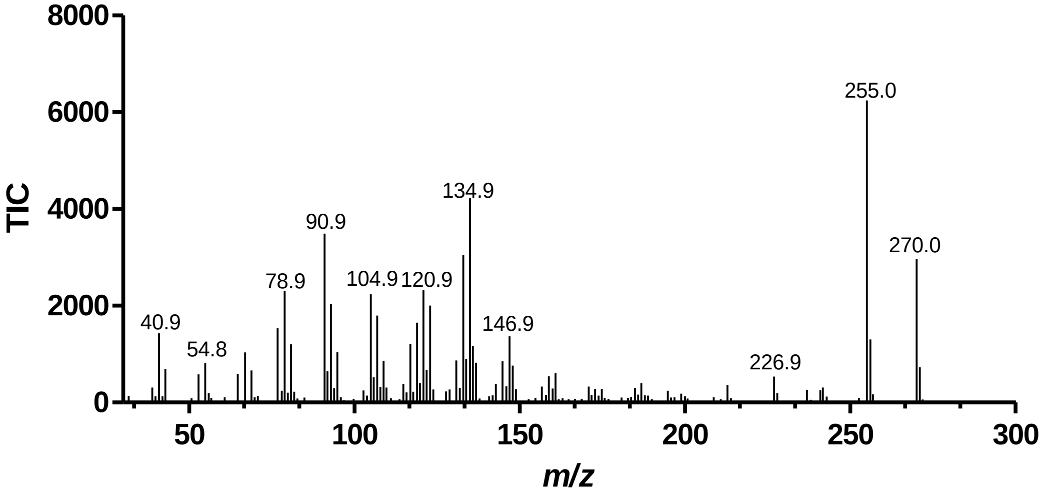

Figure S173 EI mass spectrum of **10c**.

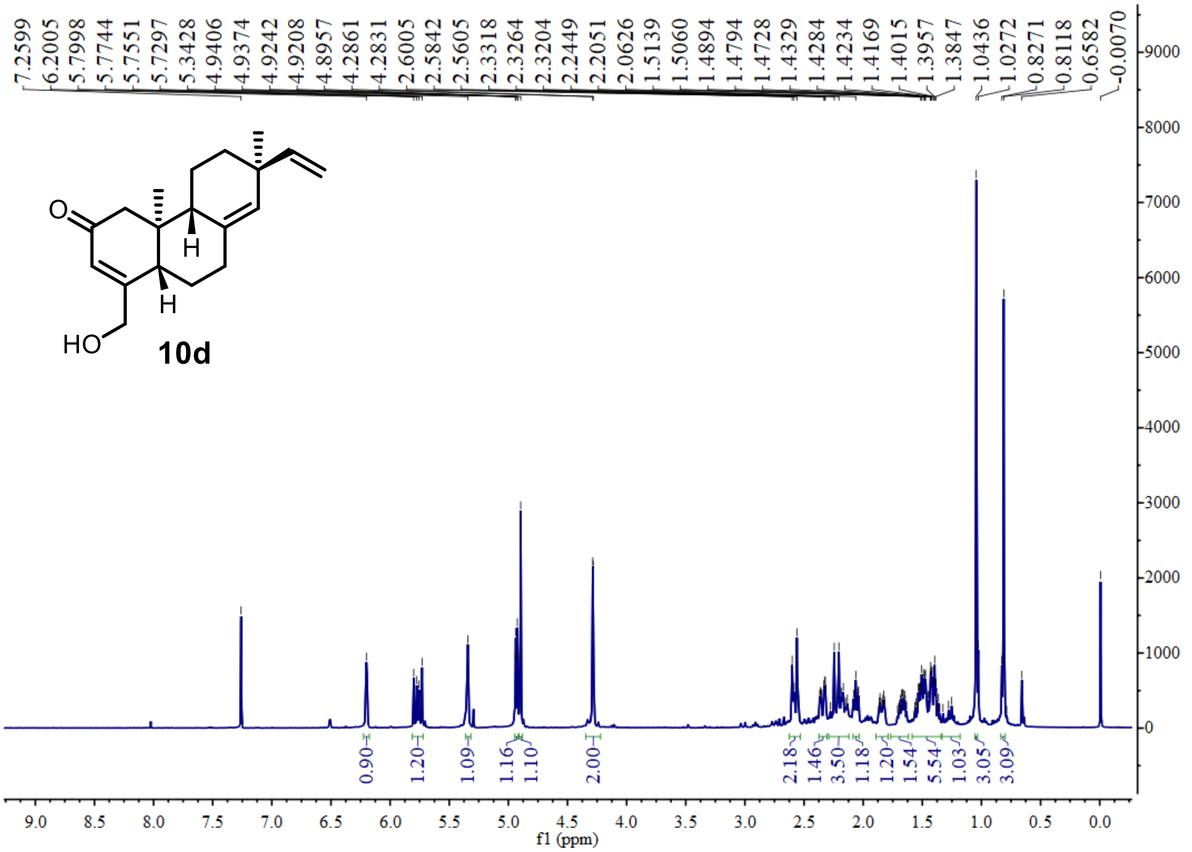

Figure S174 ^{1}H NMR spectrum of **10d** in $CDCl_3$ (400 MHz).

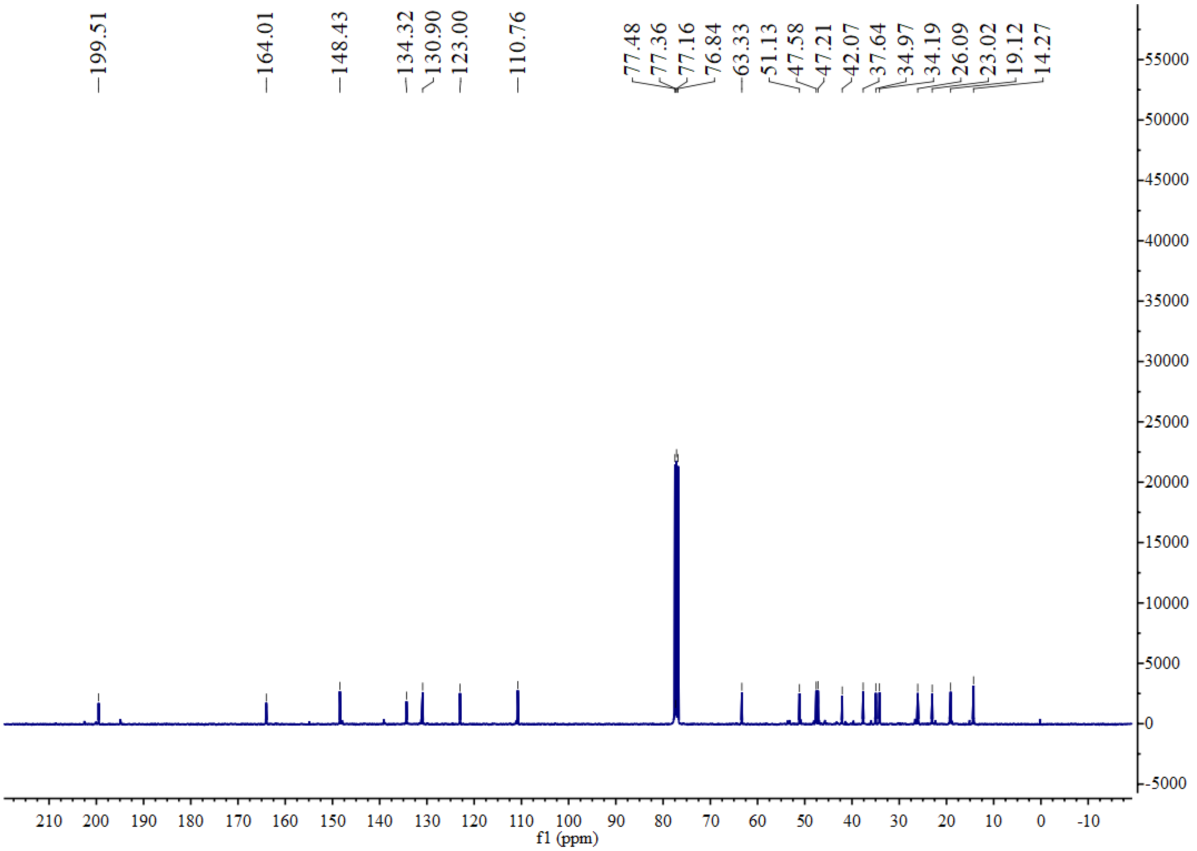

Figure S175 ^{13}C NMR spectrum of **10d** in $CDCl_3$ (100 MHz).

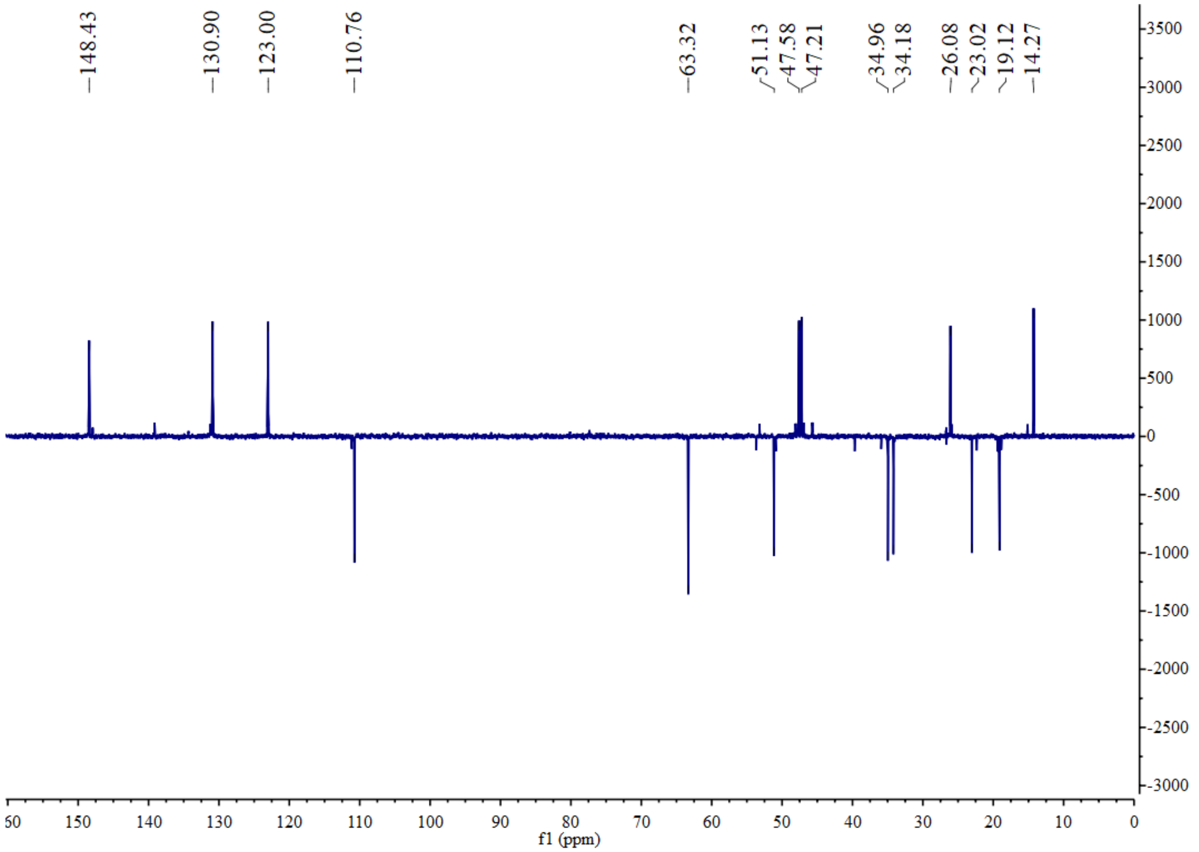

Figure S176 DEPT-135 NMR spectrum of **10d** in $CDCl_3$ (100 MHz).

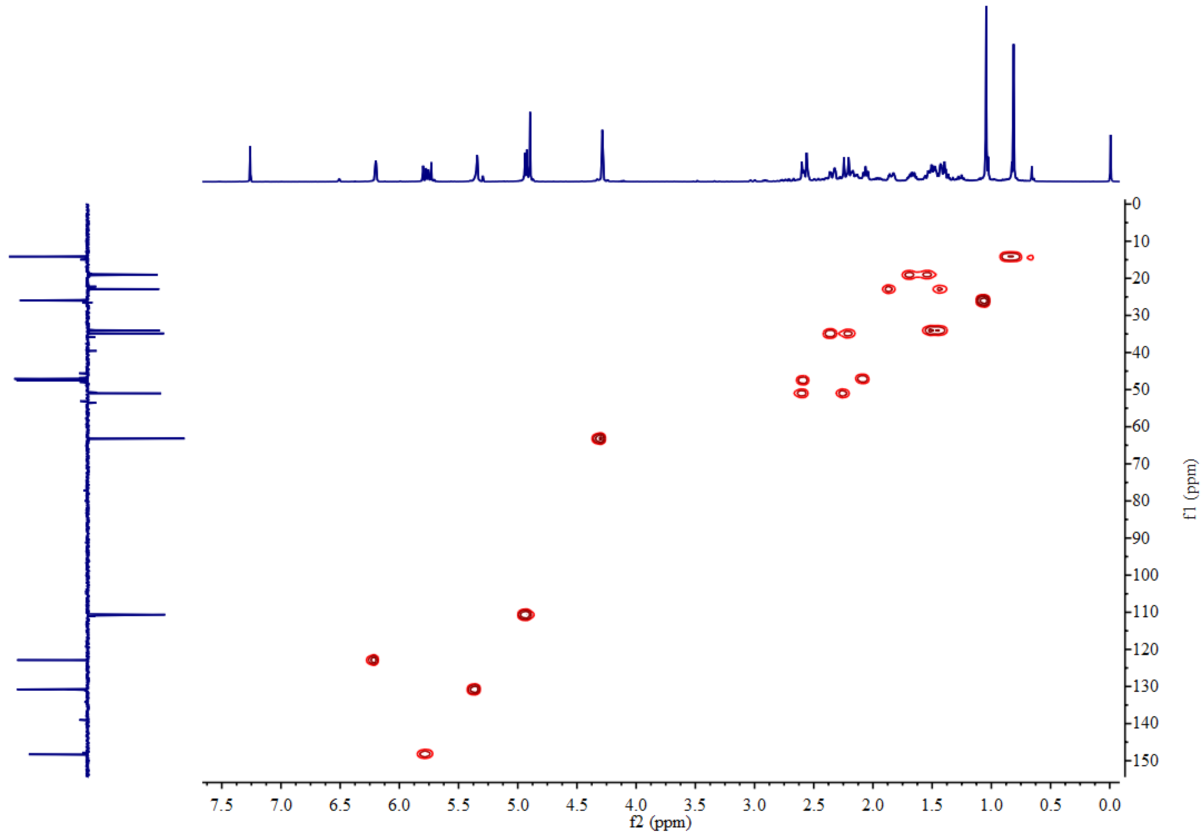

Figure S177 HSQC spectrum of **10d** in $CDCl_3$ (400 MHz).

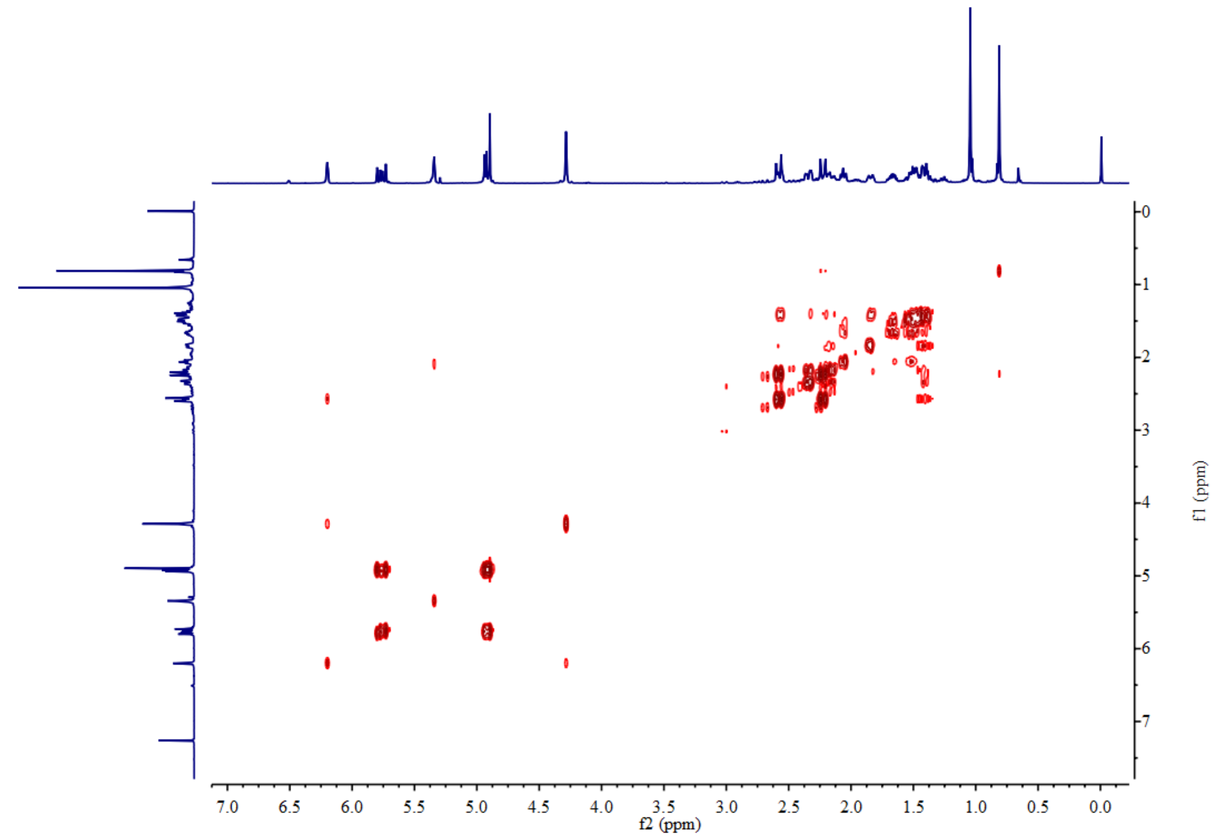

Figure S178 ^1H-^1H COSY spectrum of **10d** in $CDCl_3$ (400 MHz).

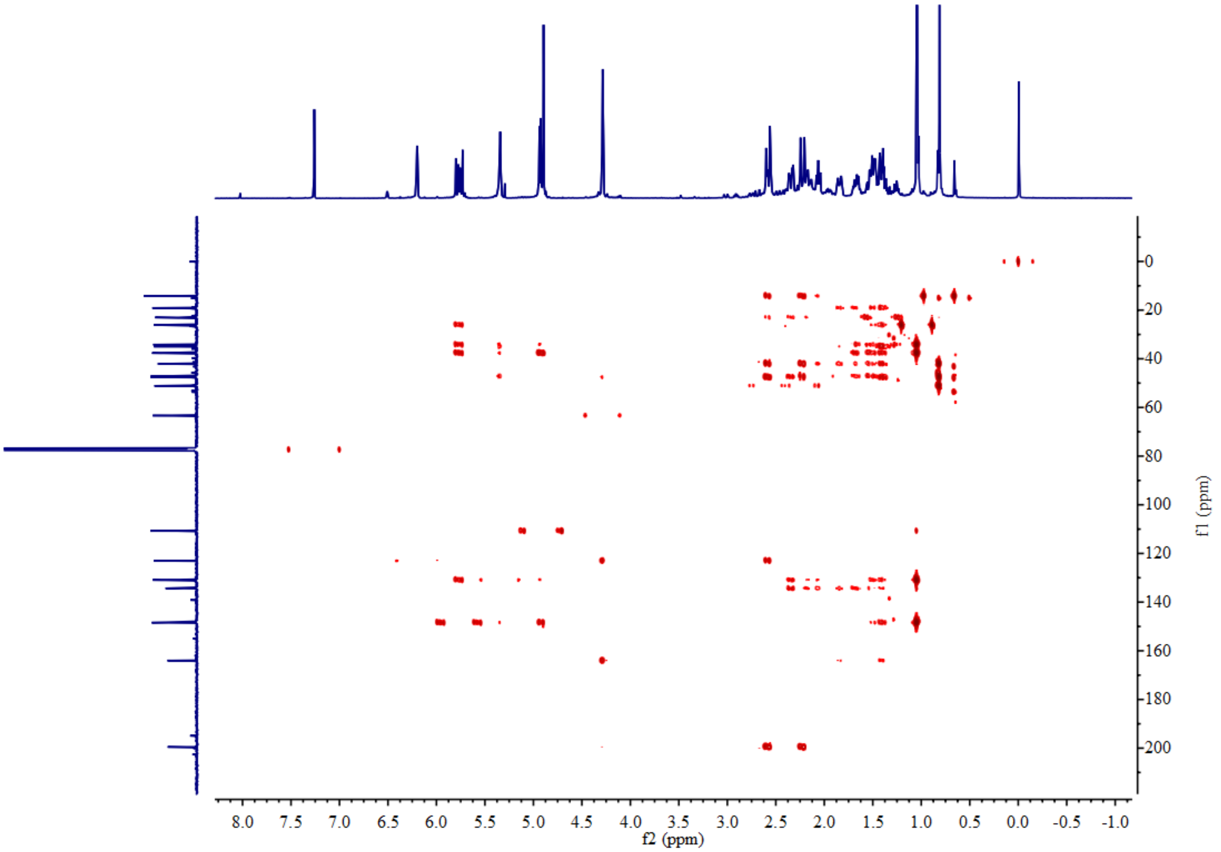

Figure S179 HMBC spectrum of **10d** in $CDCl_3$ (400 MHz).

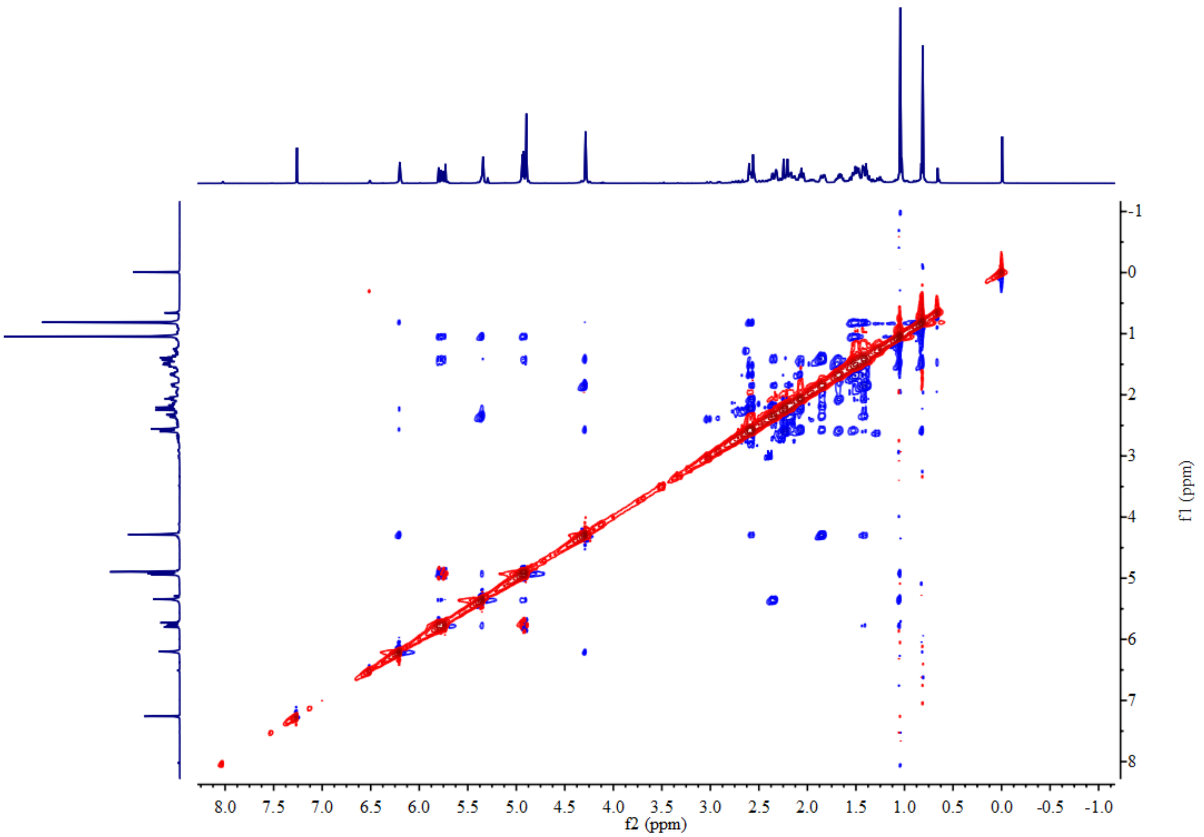

Figure S180 NOESY spectrum of **10d** in $CDCl_3$ (400 MHz).

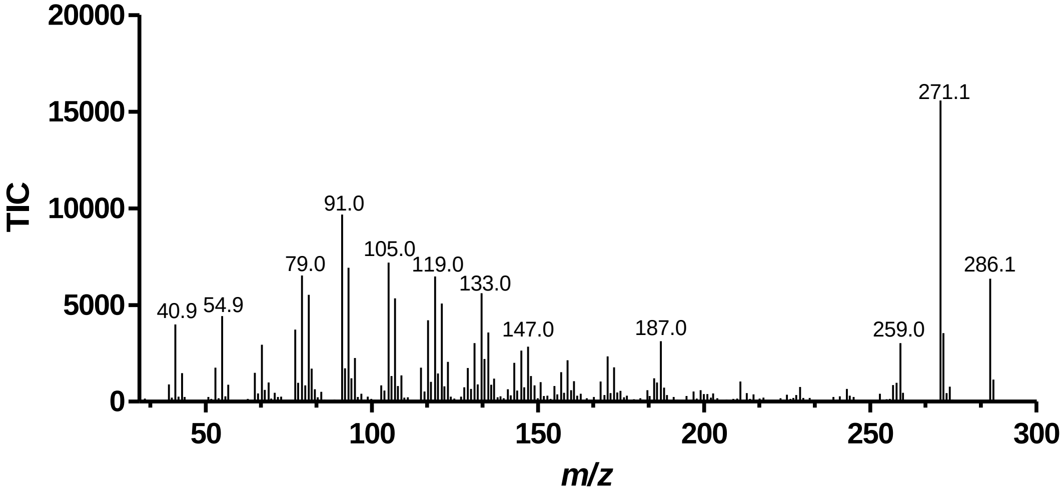

Figure S181 EI mass spectrum of **10d**.

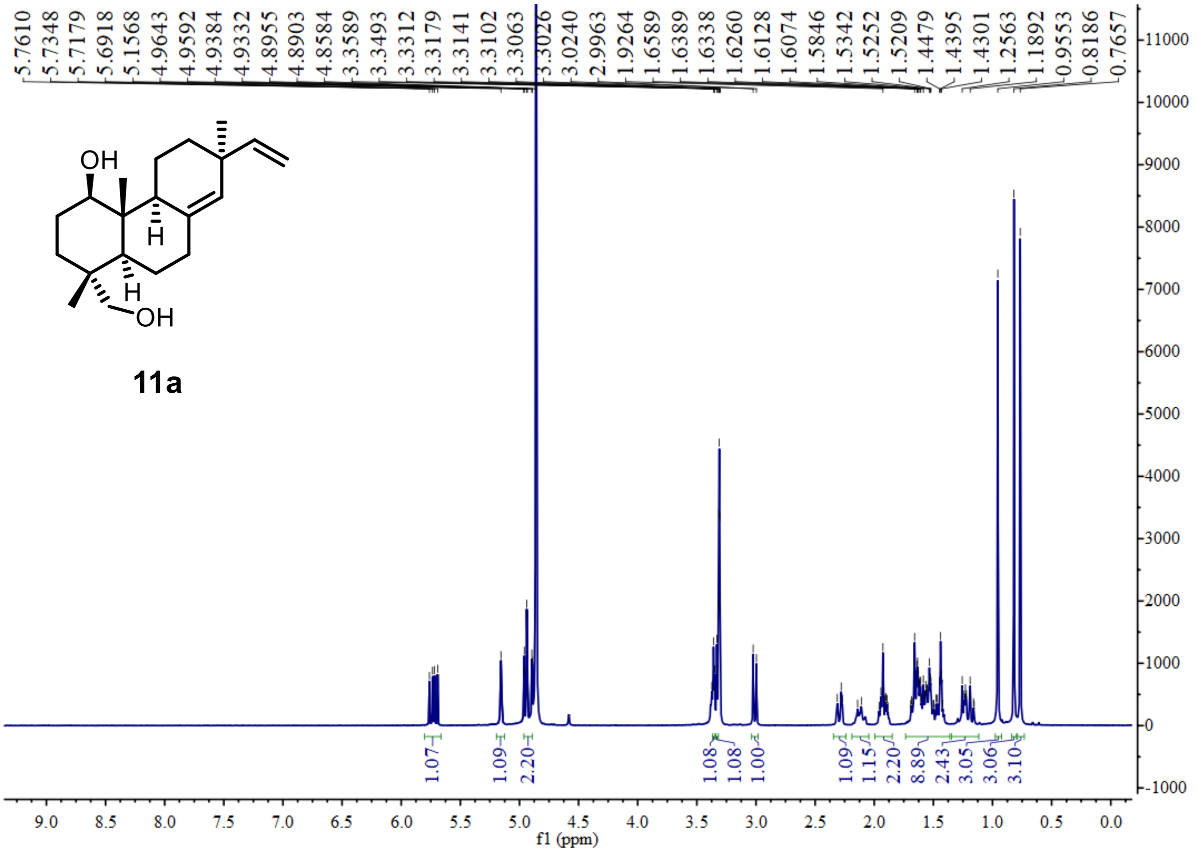

Figure S182 ^{1}H NMR spectrum of **11a** in methanol-d_4 (400 MHz).

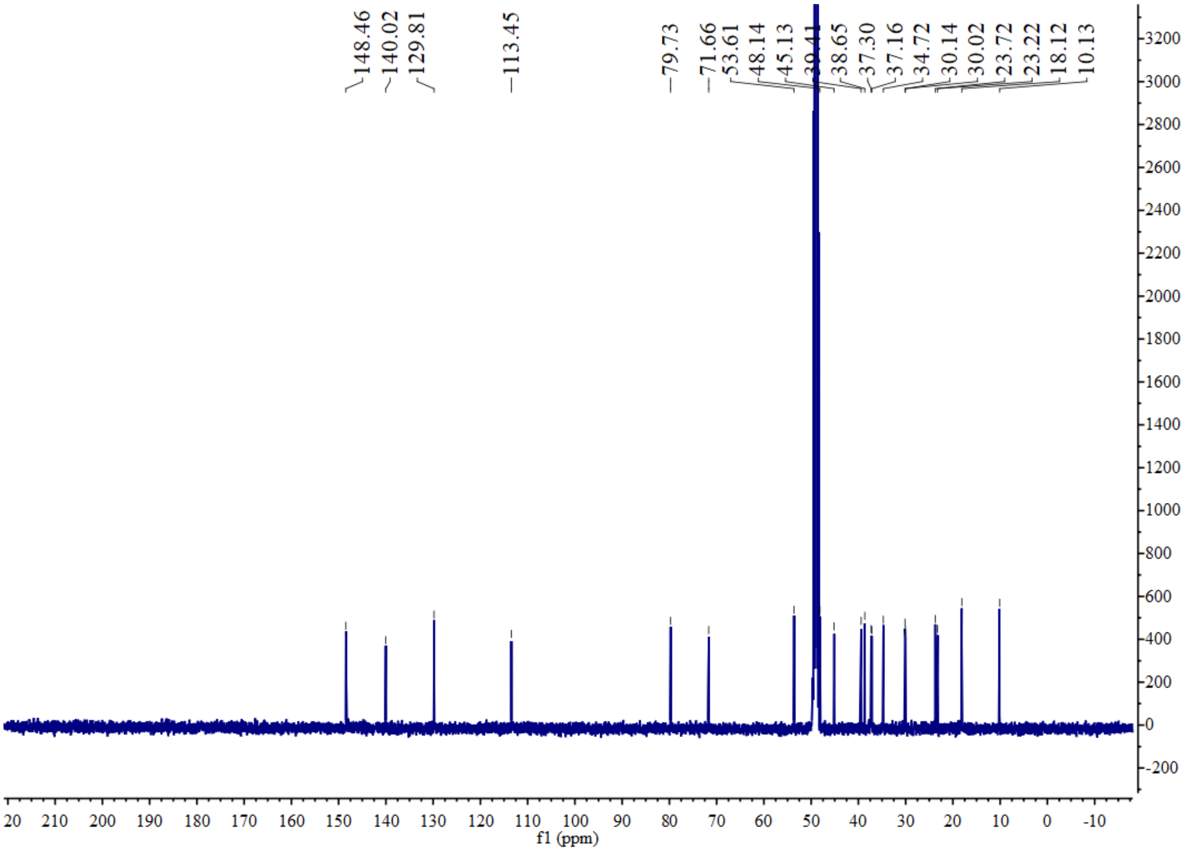

Figure S183 ^{13}C NMR spectrum of **11a** in methanol-d_4 (100 MHz).

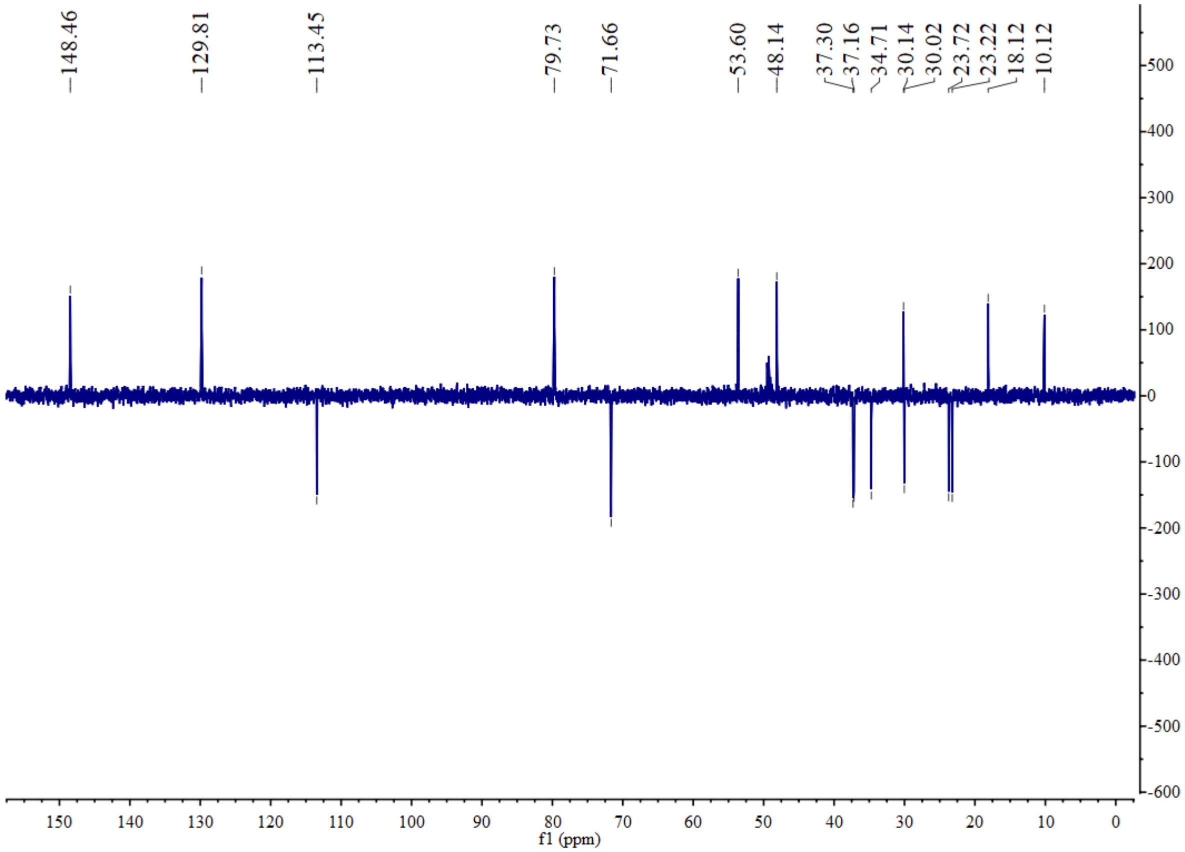

Figure S184 DEPT-135 NMR spectrum of **11a** in methanol-d_4 (100 MHz).

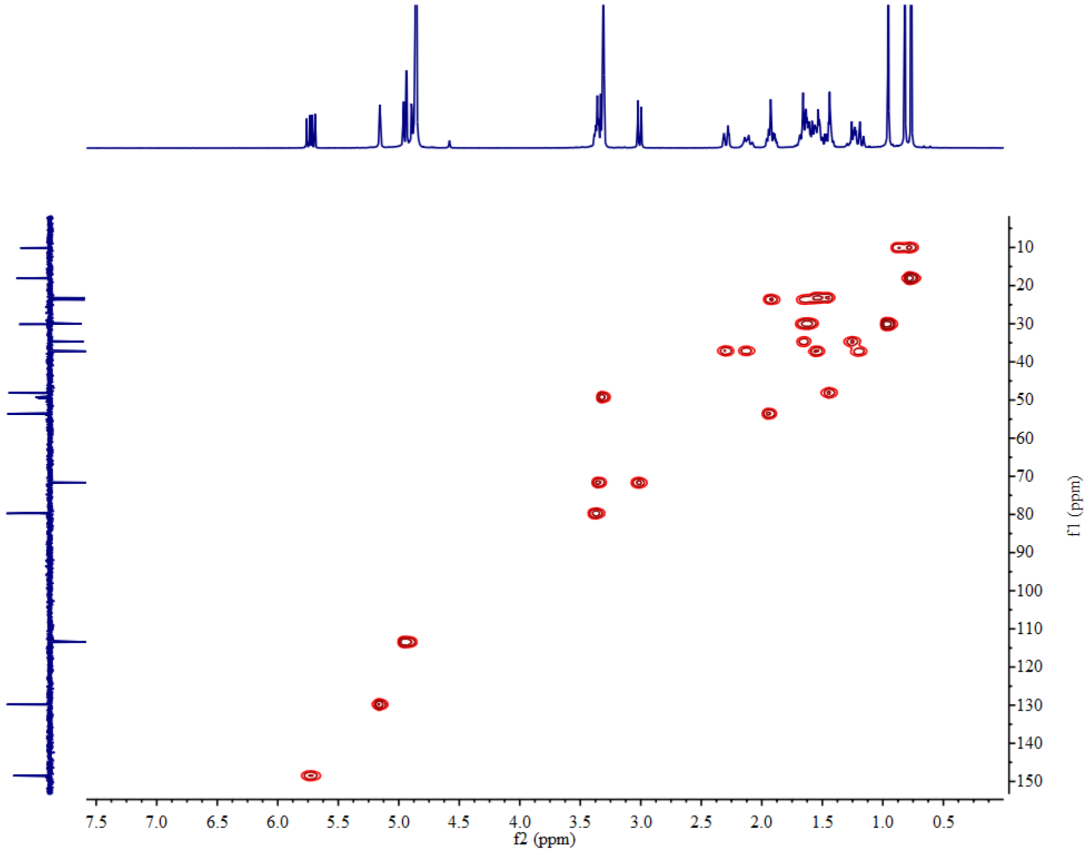

Figure S185 HSQC spectrum of **11a** in methanol-d_4 (400 MHz).

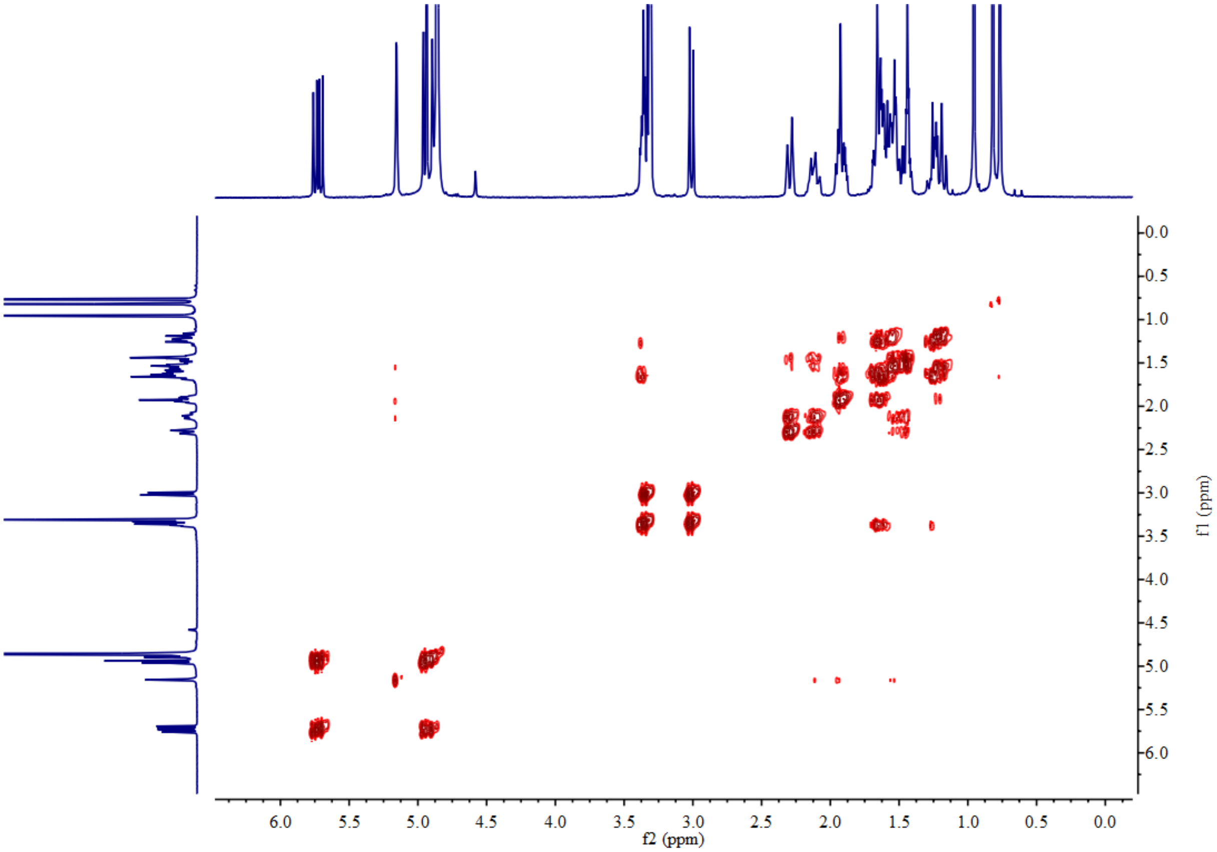

Figure S186 ^{1}H-^{1}H COSY spectrum of **11a** in methanol-d_4 (400 MHz).

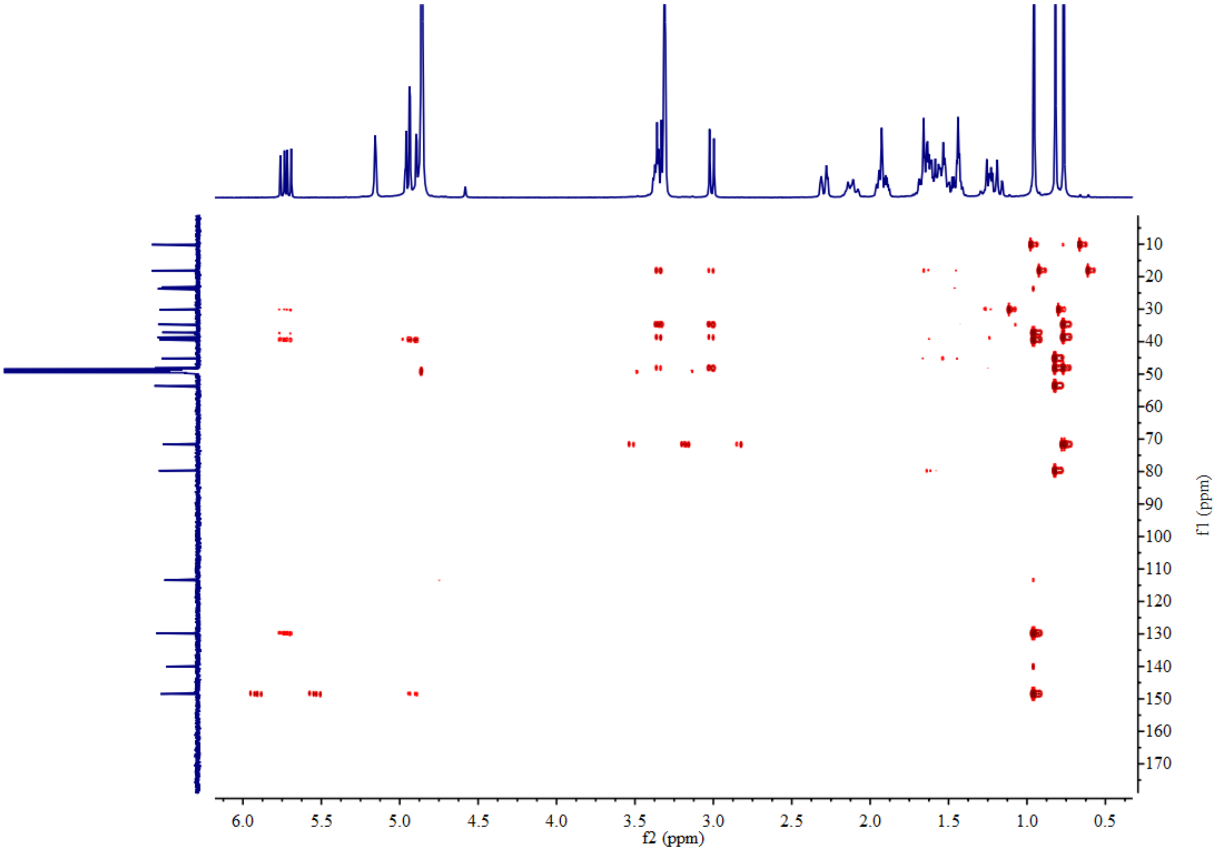

Figure S187 HMBC spectrum of **11a** in methanol-d_4 (400 MHz).

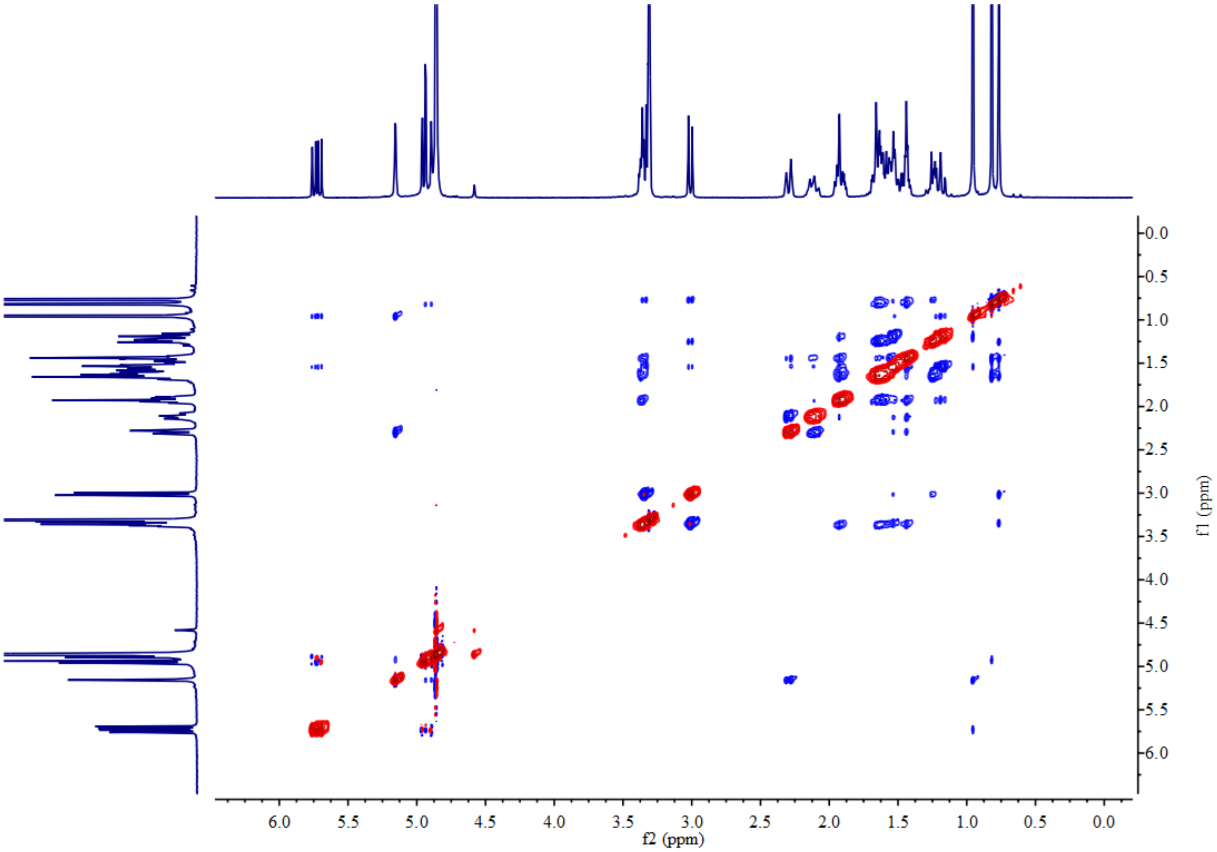

Figure S188 NOESY spectrum of **11a** in methanol-d_4 (400 MHz).

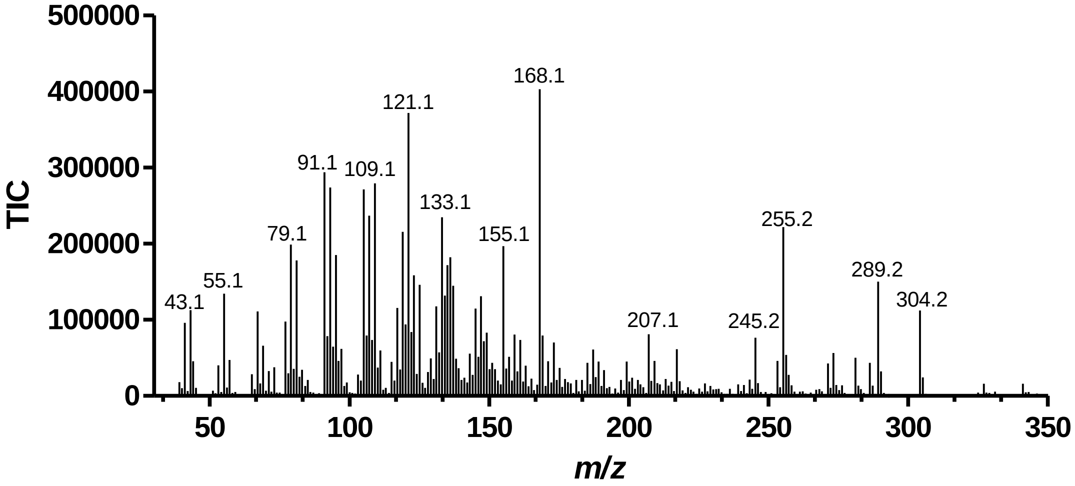

Figure S189 EI mass spectrum of **11a**.

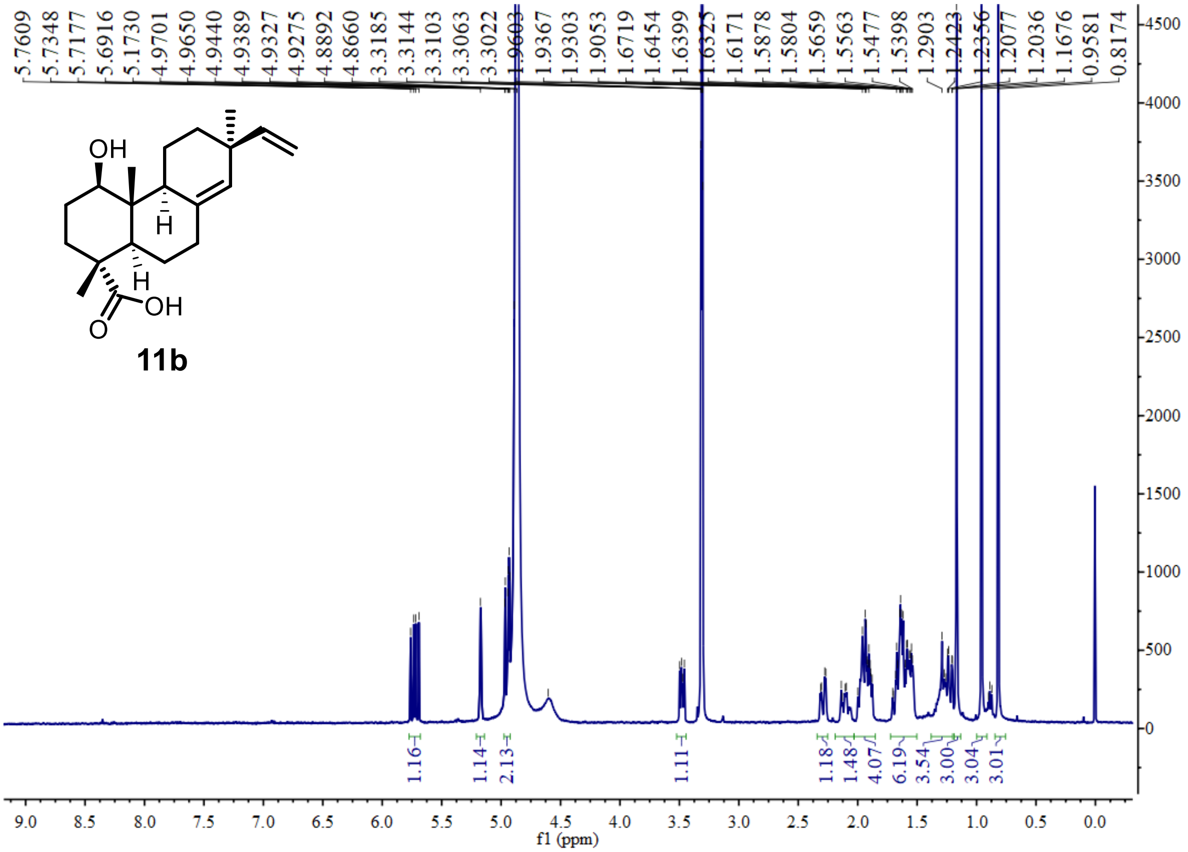

Figure S190 ^{1}H NMR spectrum of **11b** in methanol-d_4 (400 MHz).

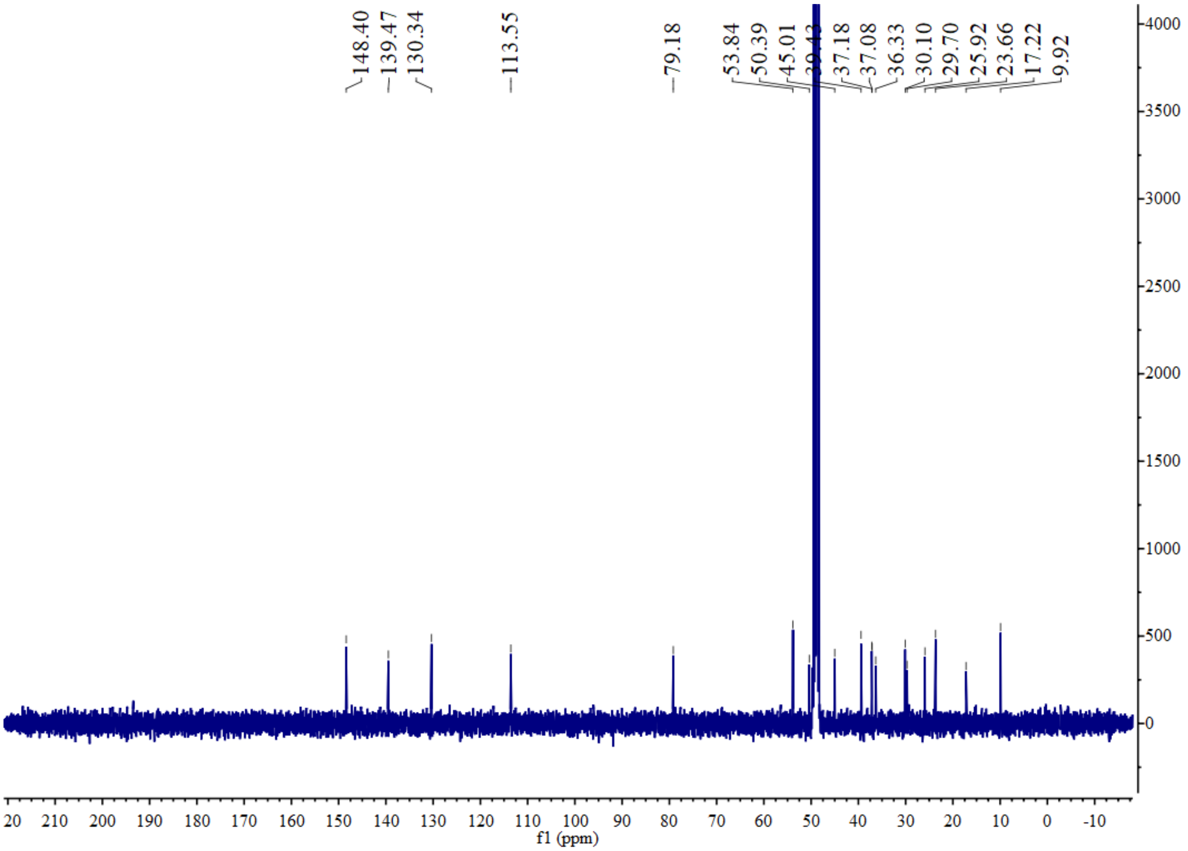

Figure S191 ^{13}C NMR spectrum of **11b** in methanol-d_4 (100 MHz).

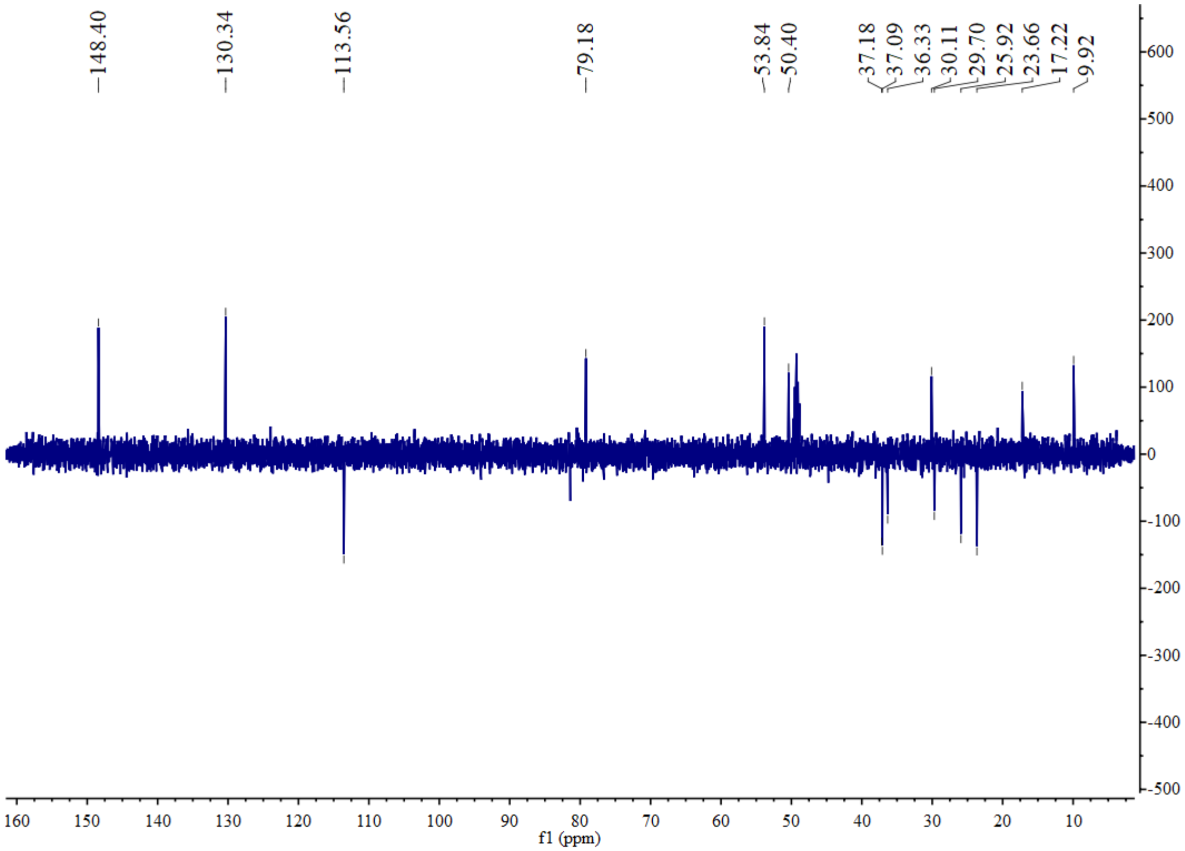

Figure S192 DEPT-135 NMR spectrum of **11b** in methanol-d_4 (100 MHz).

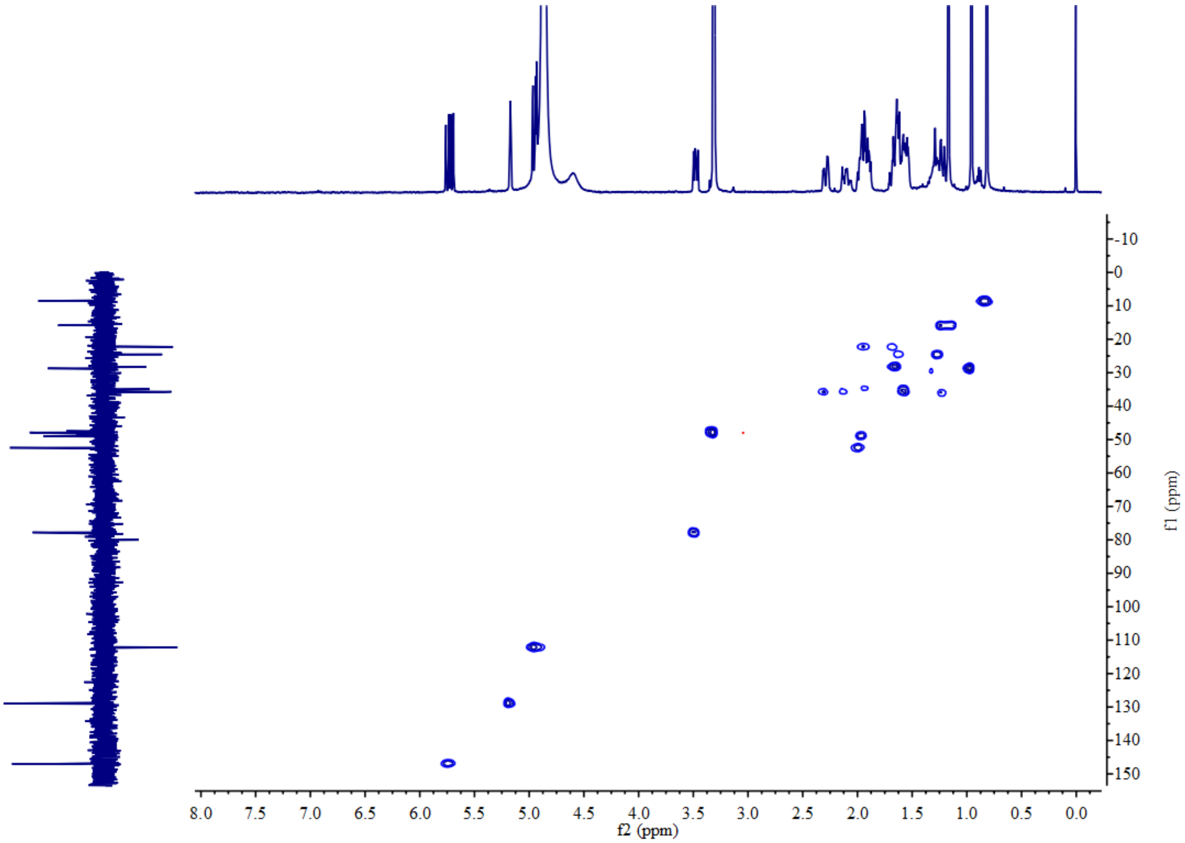

Figure S193 HSQC spectrum of **11b** in methanol-d_4 (400 MHz).

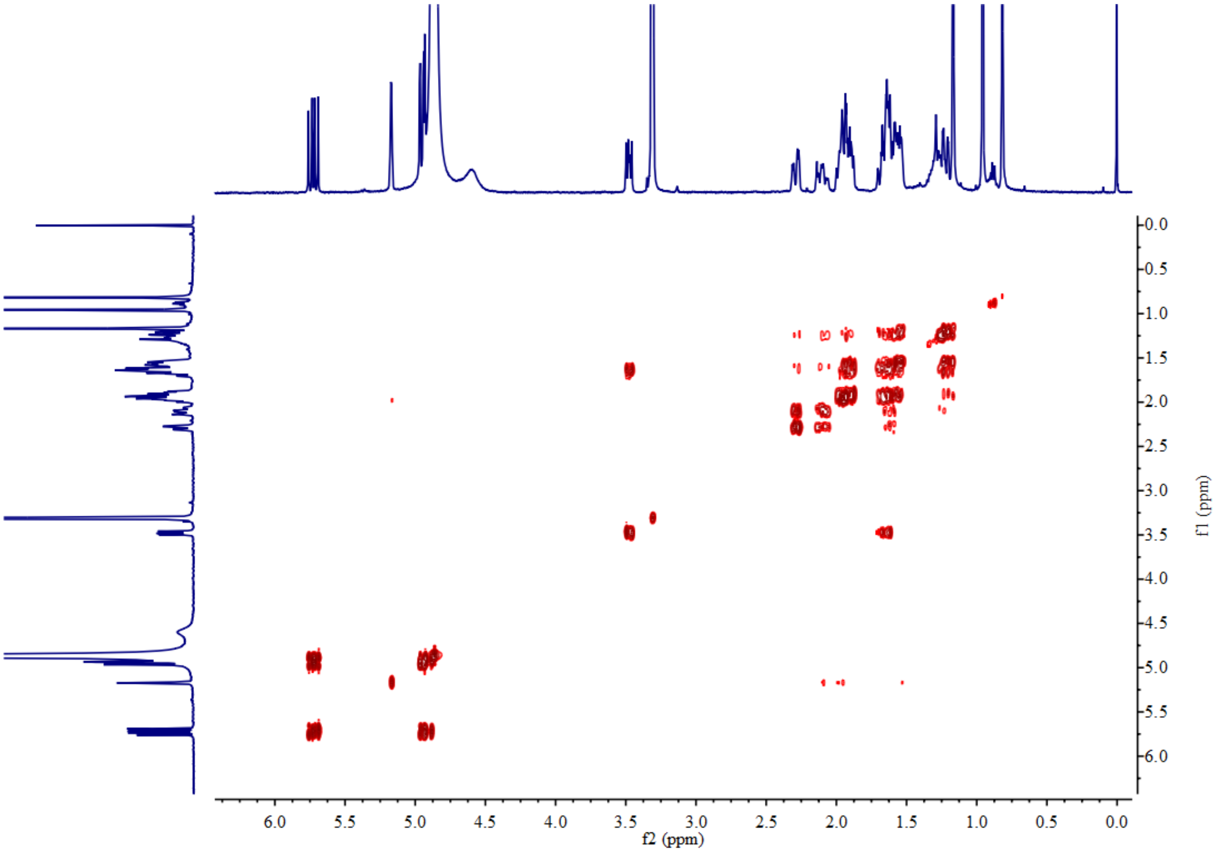

Figure S194 ^{1}H-^{1}H COSY spectrum of **11b** in methanol-d_4 (400 MHz).

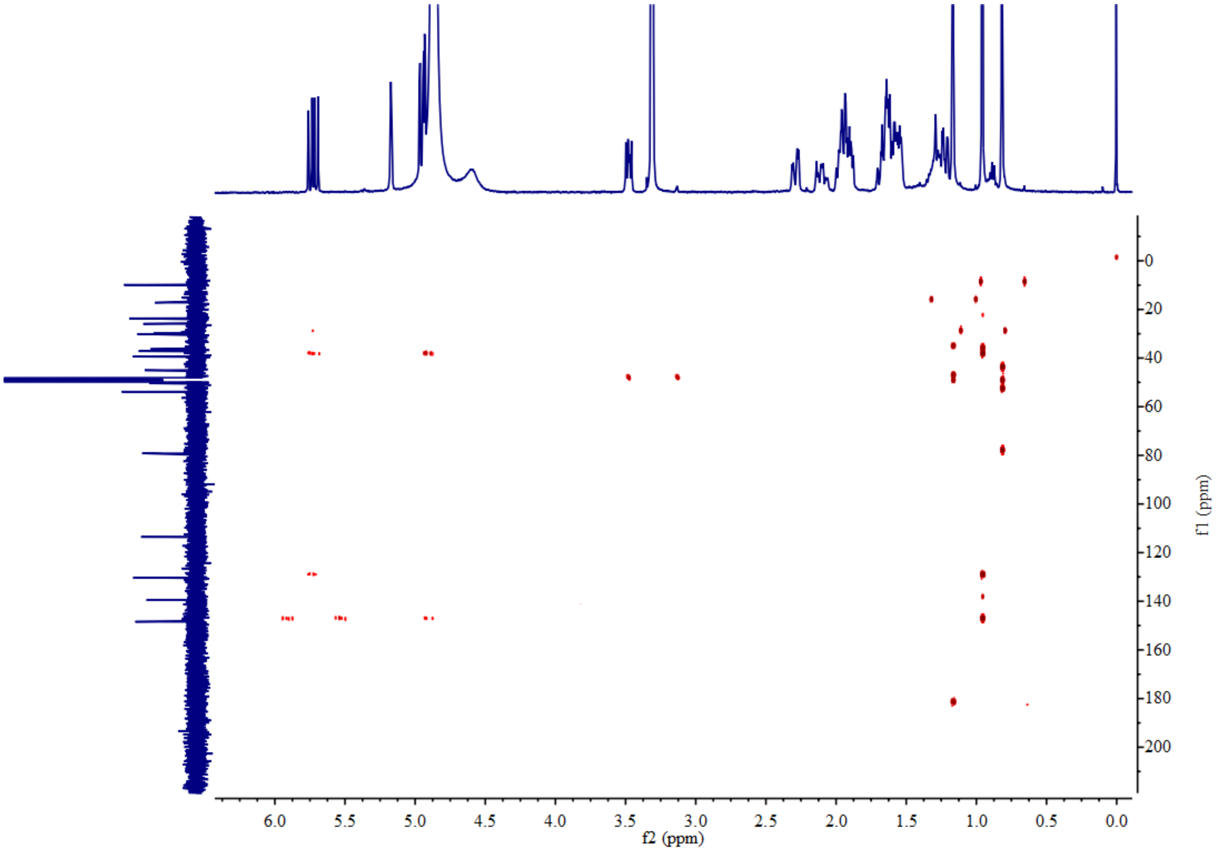

Figure S195 HMBC spectrum of **11b** in methanol-d_4 (400 MHz).

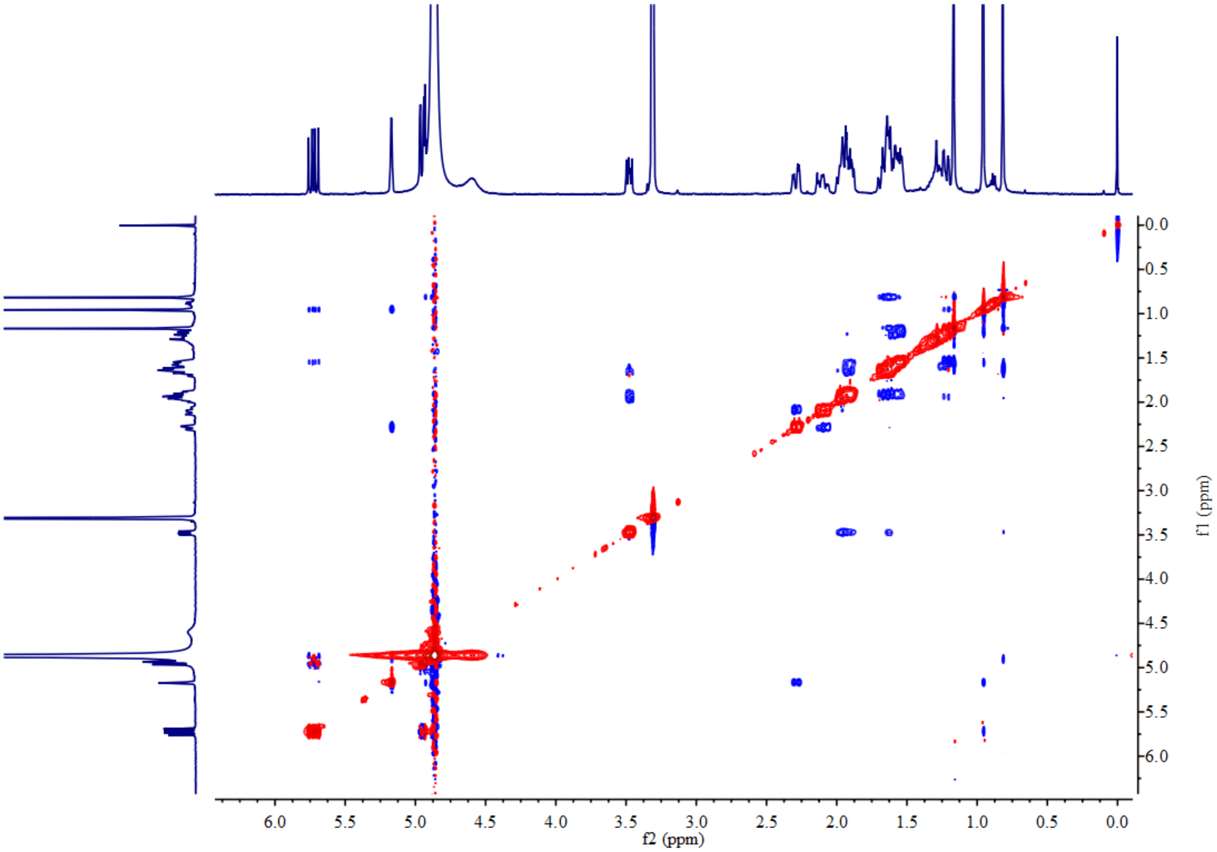

Figure S196 NOESY spectrum of **11b** in methanol-d_4 (400 MHz).

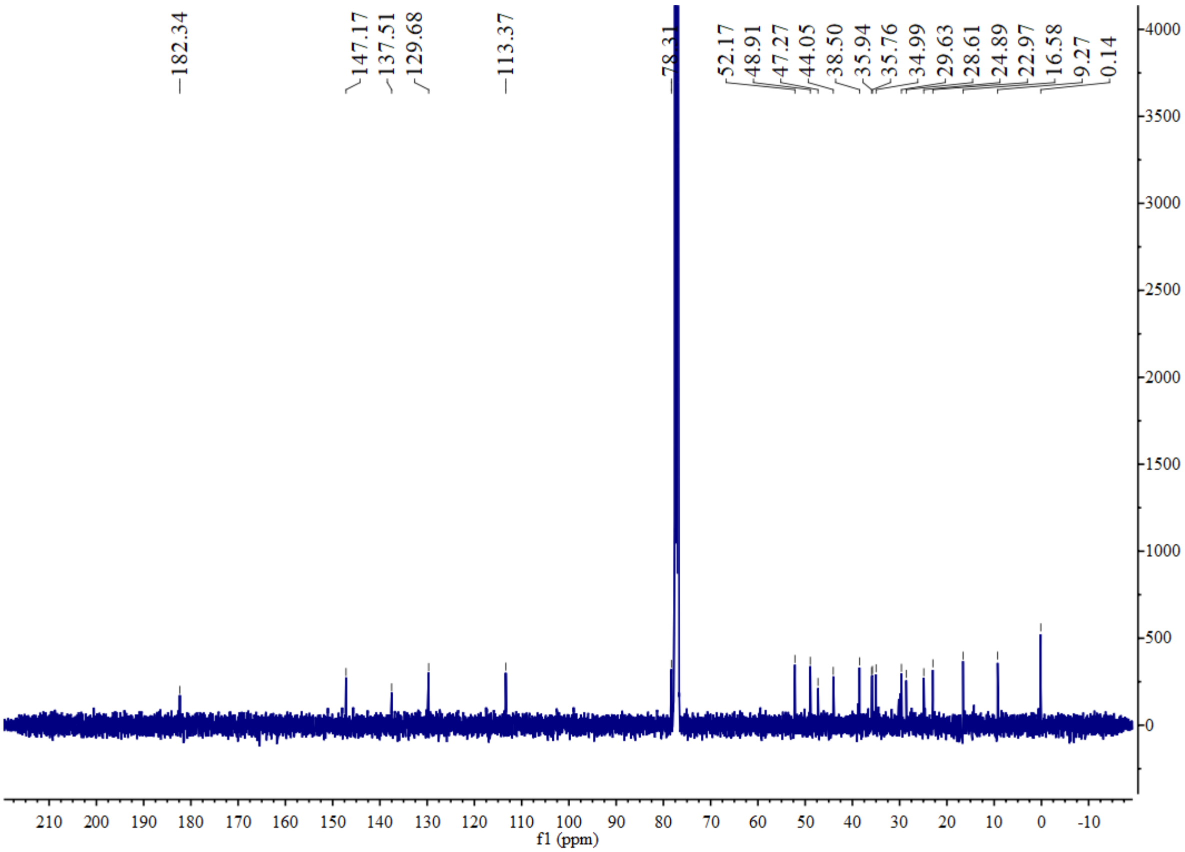

Figure S197 ^{13}C NMR spectrum of **11b** in $CDCl_3$ (100 MHz).

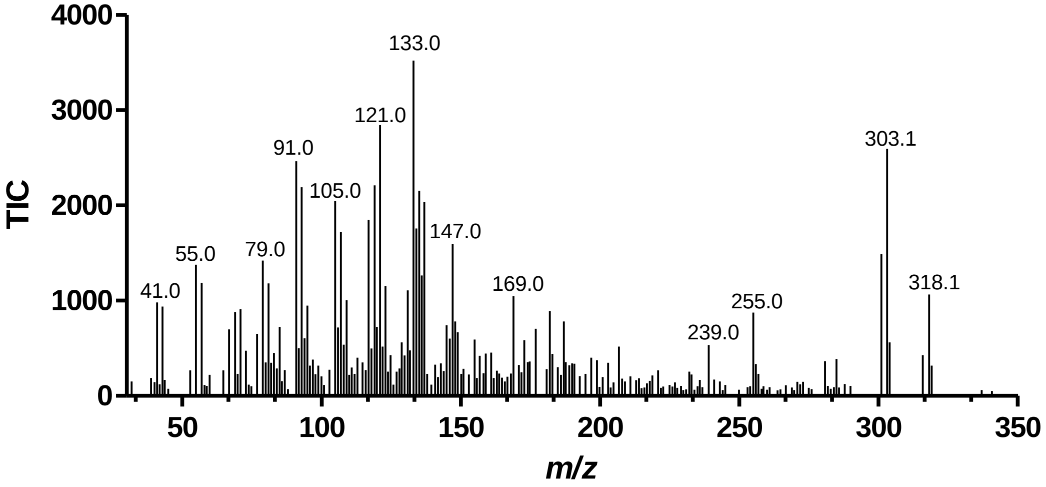

Figure S198 EI mass spectrum of **11b**.

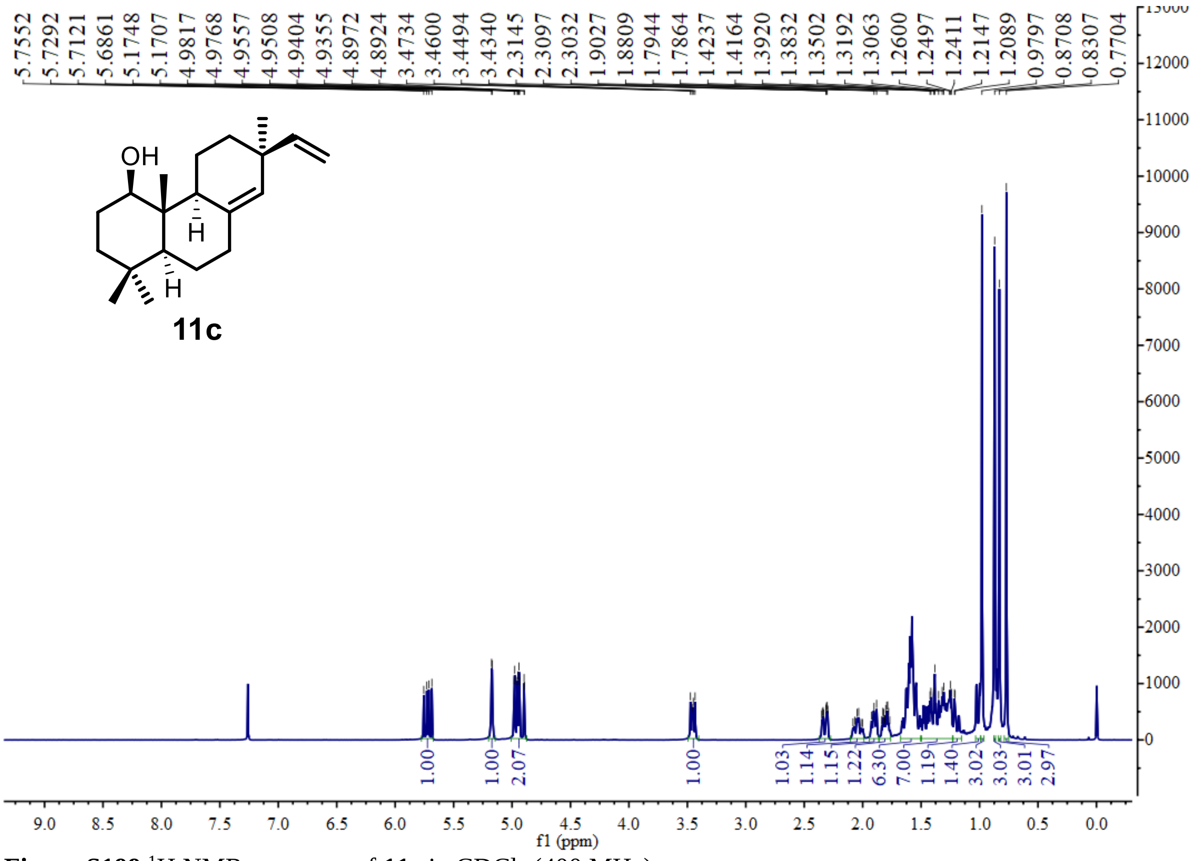

Figure S199 ^{1}H NMR spectrum of **11c** in $CDCl_3$ (400 MHz).

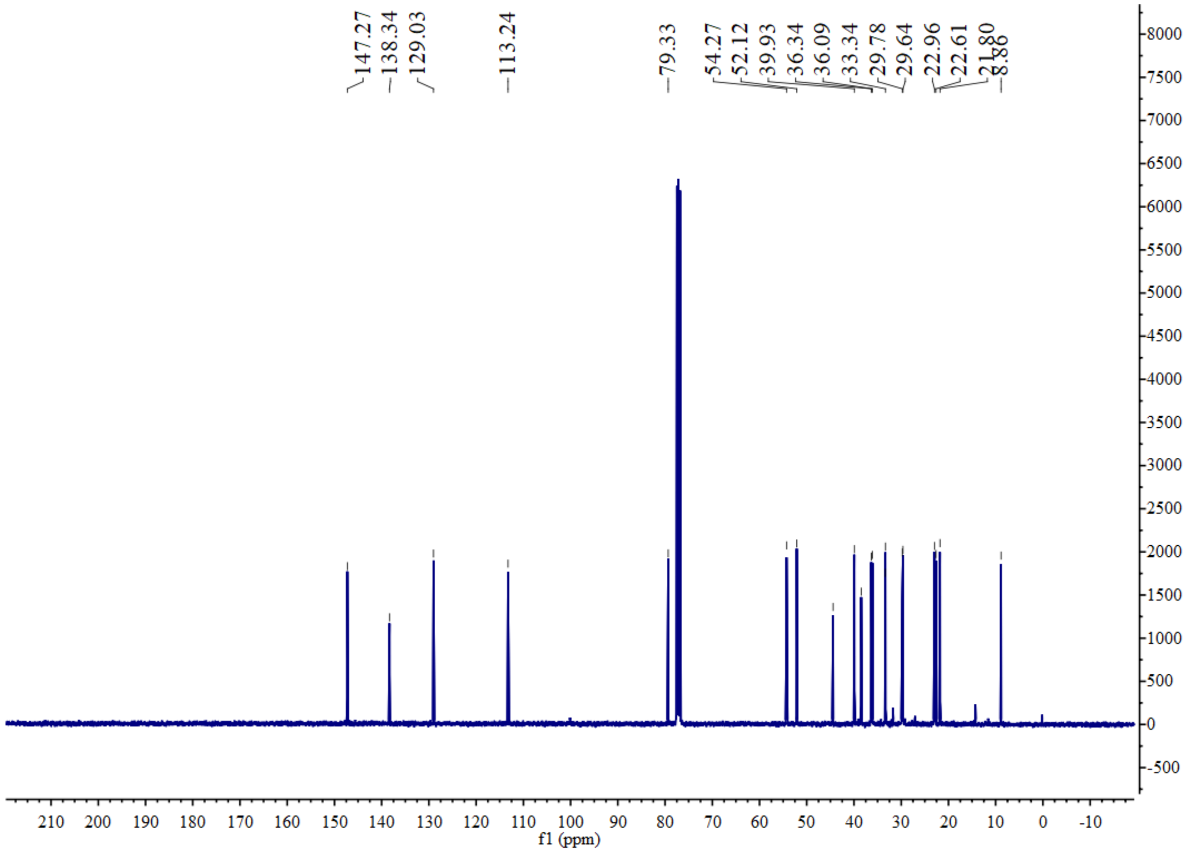

Figure S200 ^{13}C NMR spectrum of **11c** in $CDCl_3$ (100 MHz).

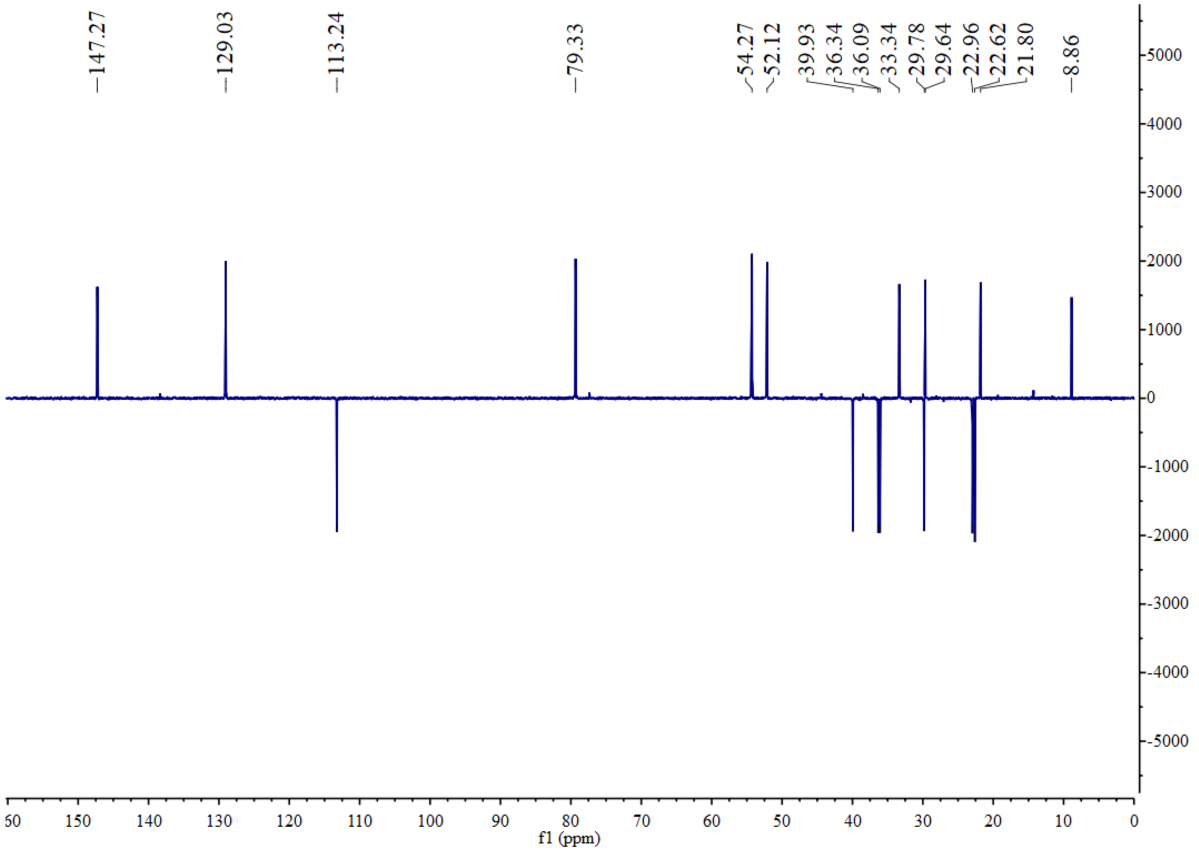

Figure S201 DEPT-135 NMR spectrum of **11c** in $CDCl_3$ (100 MHz).

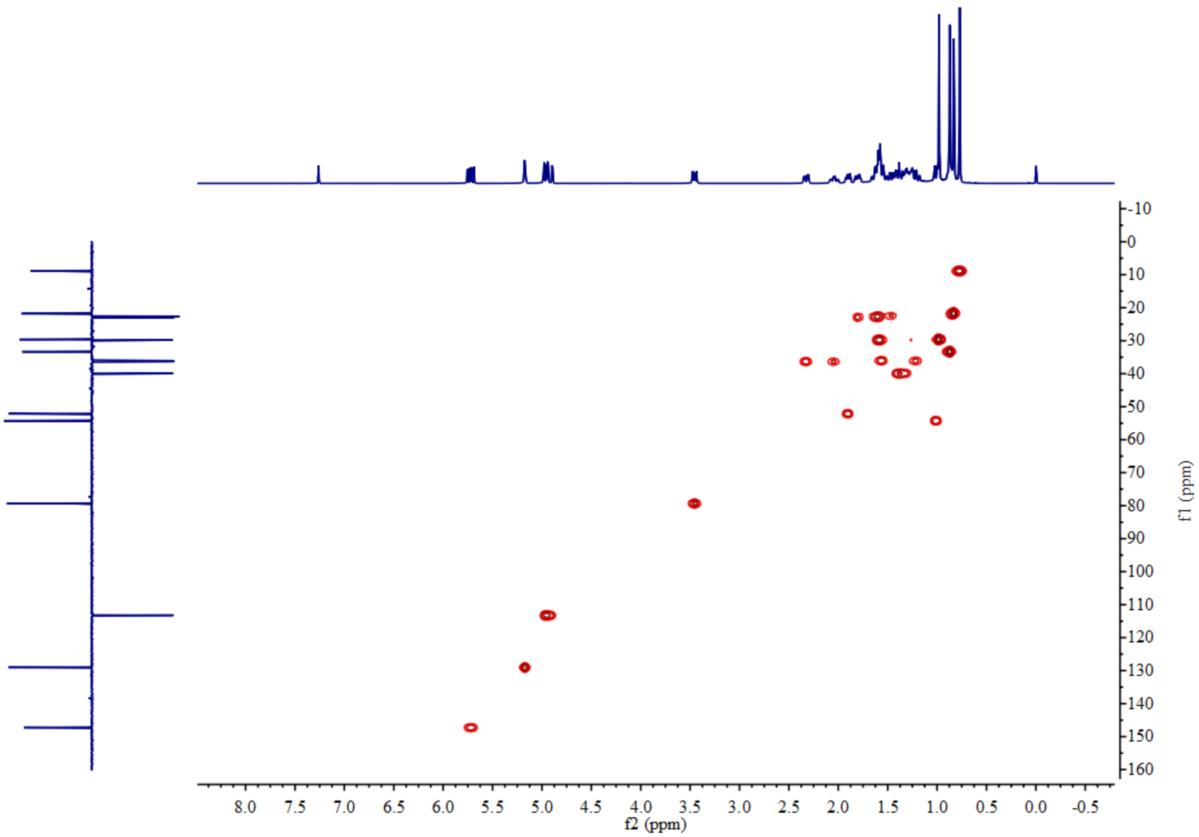

Figure S202 HSQC spectrum of **11c** in $CDCl_3$ (400 MHz).

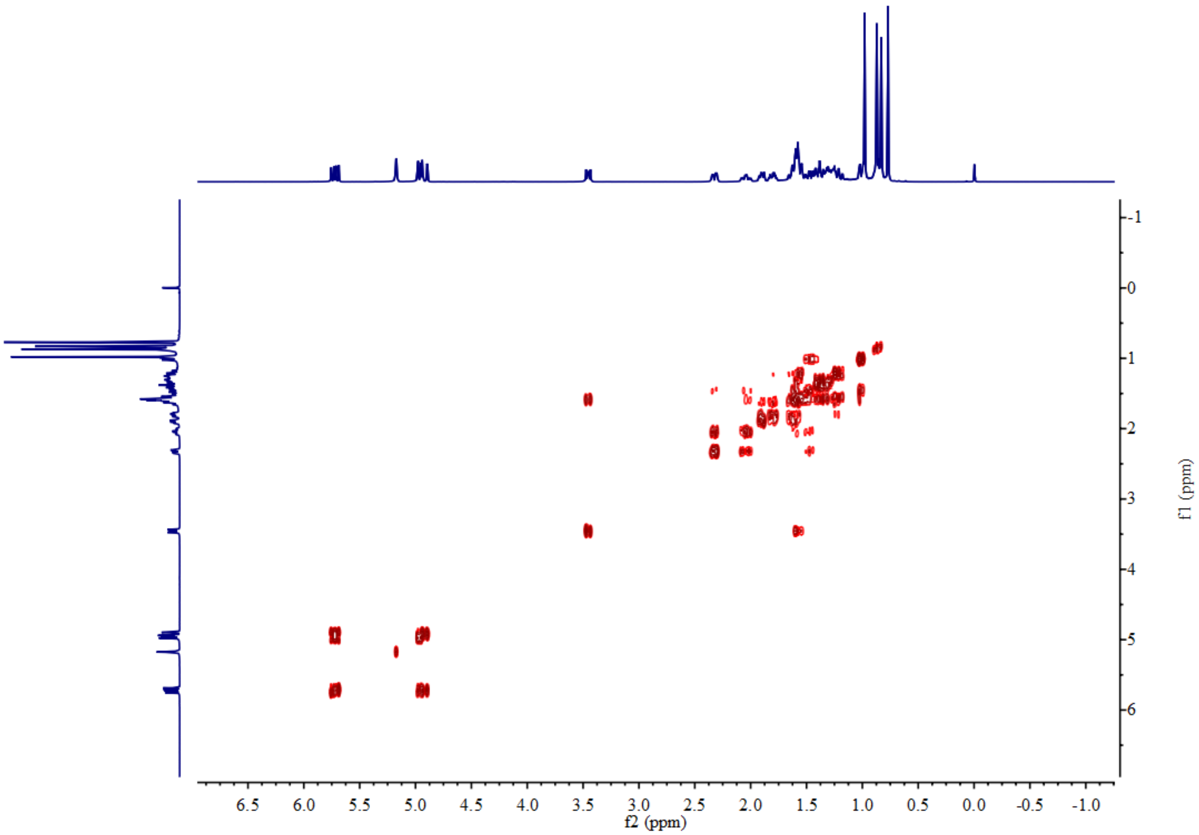

Figure S203 ^1H-^1H COSY spectrum of **11c** in $CDCl_3$ (400 MHz).

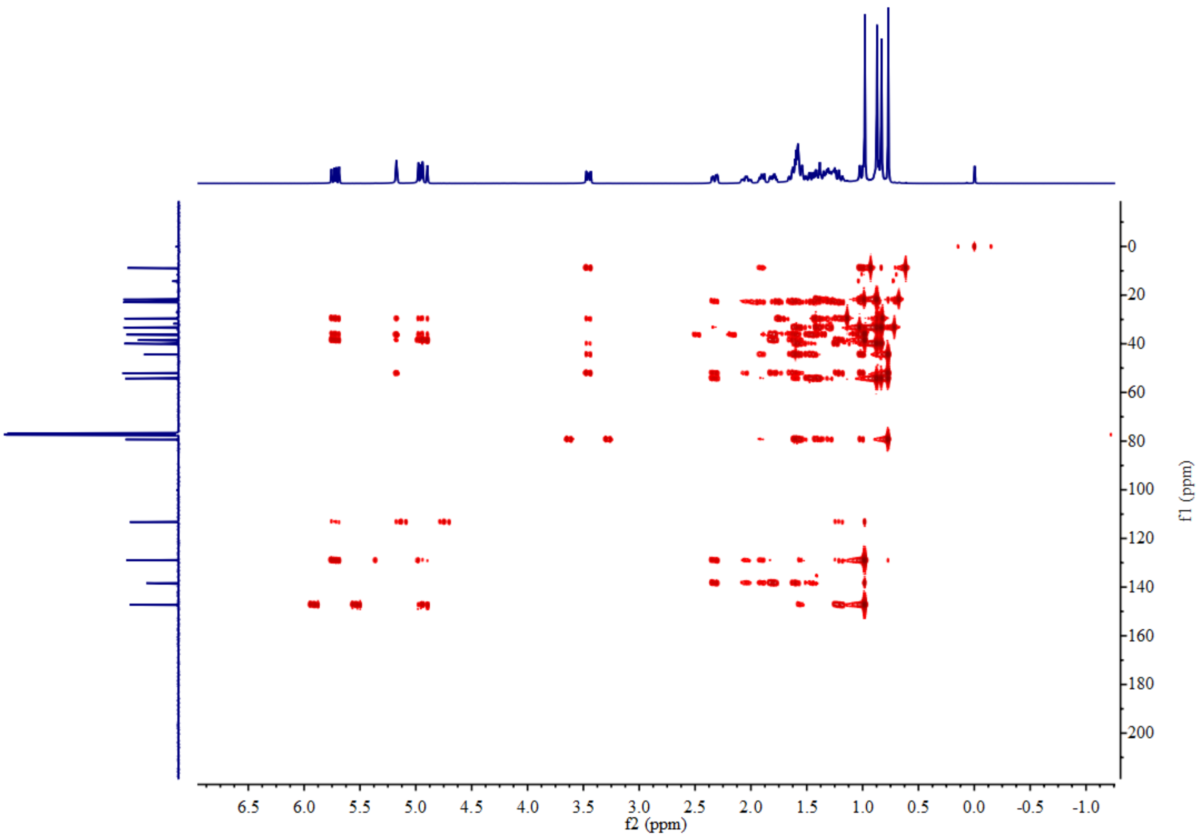

Figure S204 HMBC spectrum of **11c** in $CDCl_3$ (400 MHz).

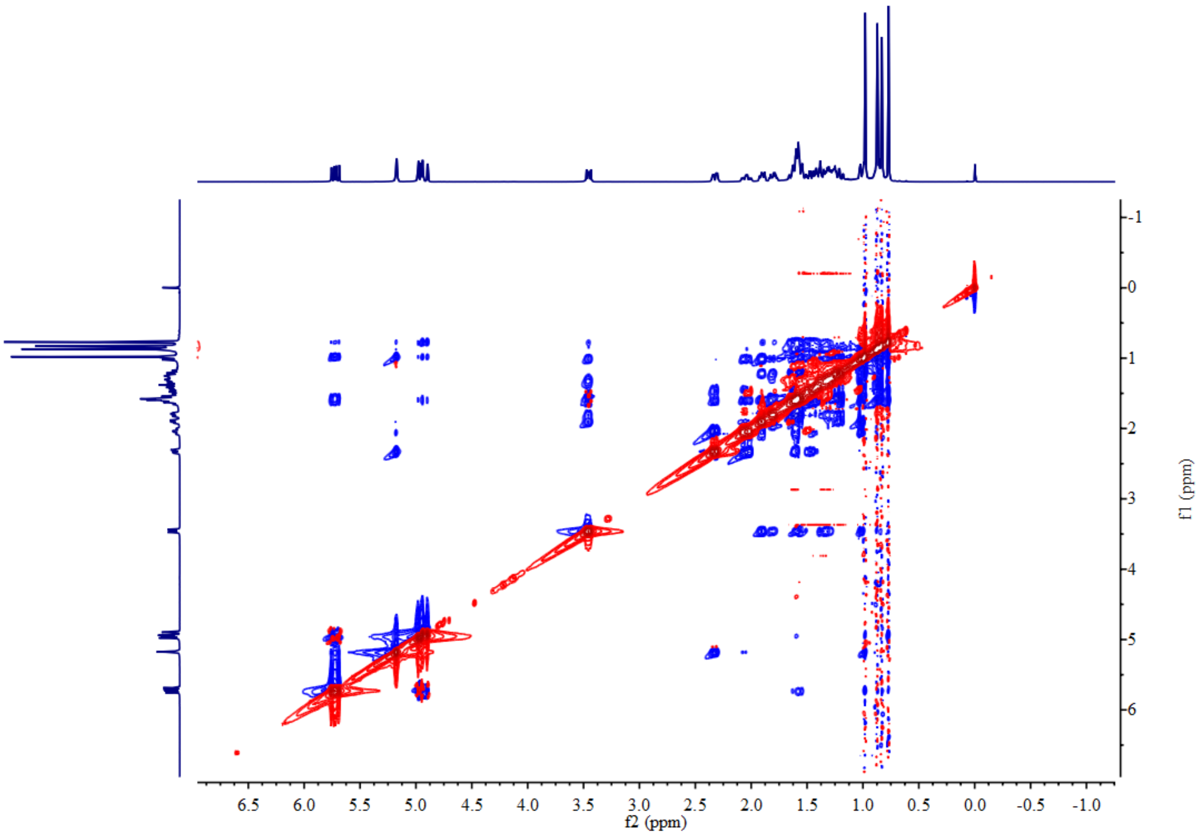

Figure S205 NOESY spectrum of **11c** in $CDCl_3$ (400 MHz).

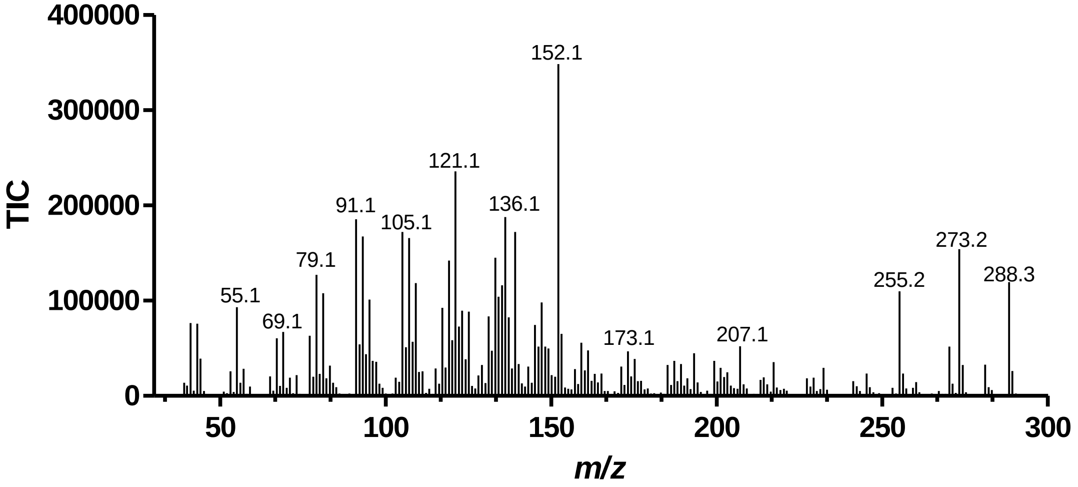

Figure S206 EI mass spectrum of **11c**.

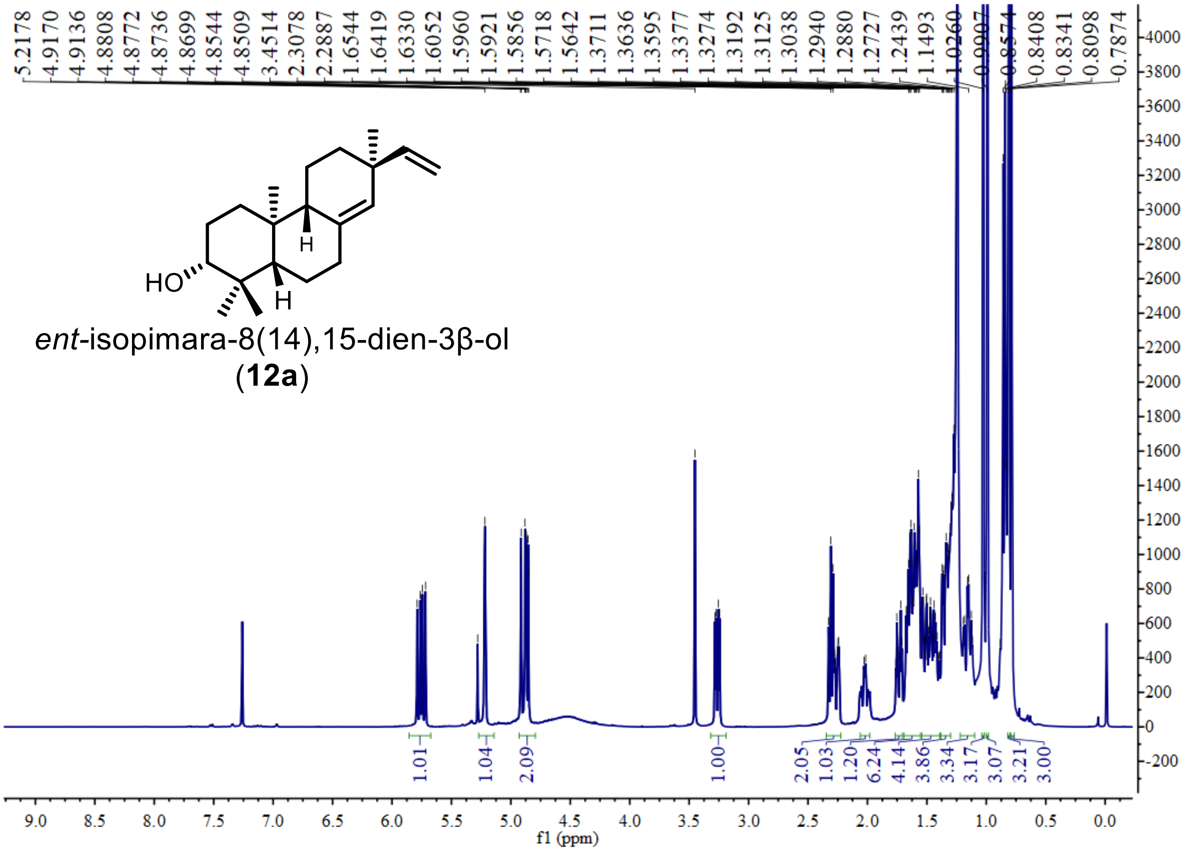

Figure S207 ^{1}H NMR spectrum of **12a** in $CDCl_3$ (400 MHz).

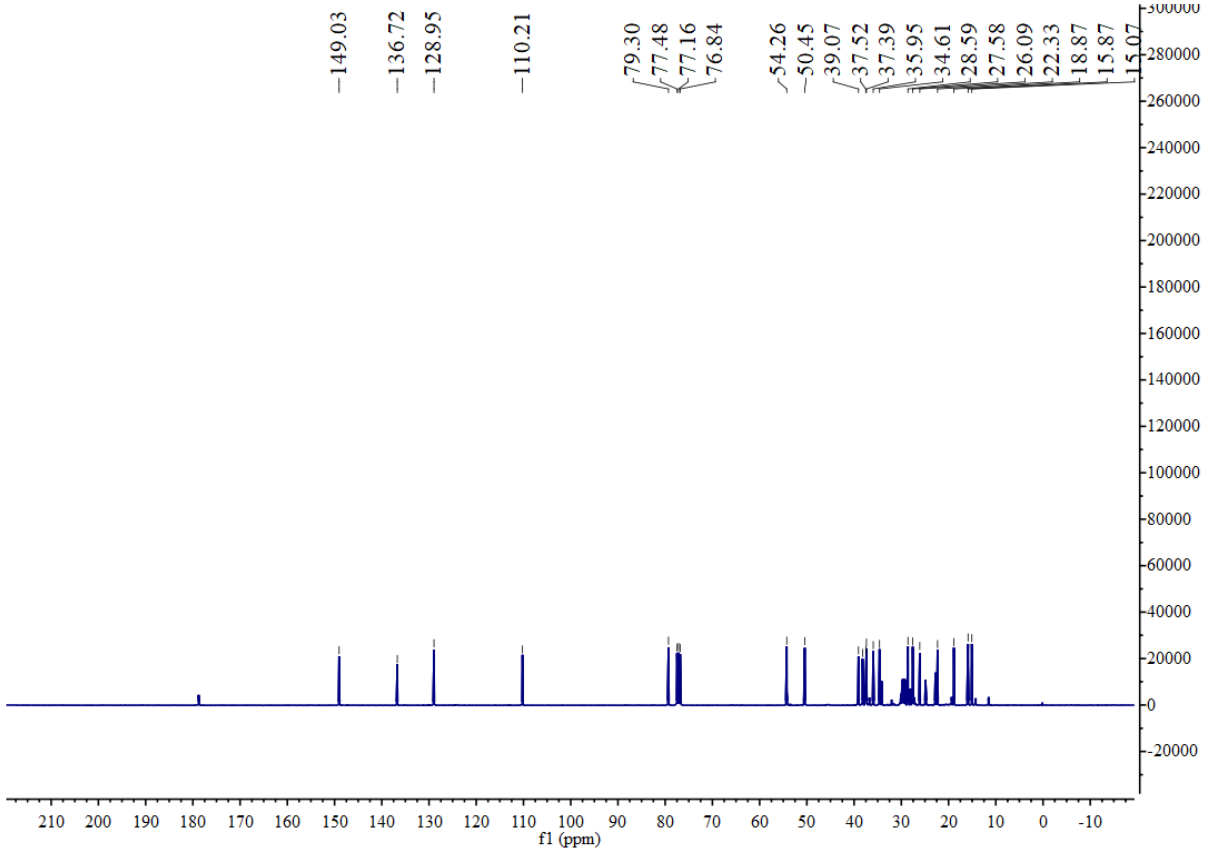

Figure S208 ^{13}C NMR spectrum of **12a** in $CDCl_3$ (100 MHz).

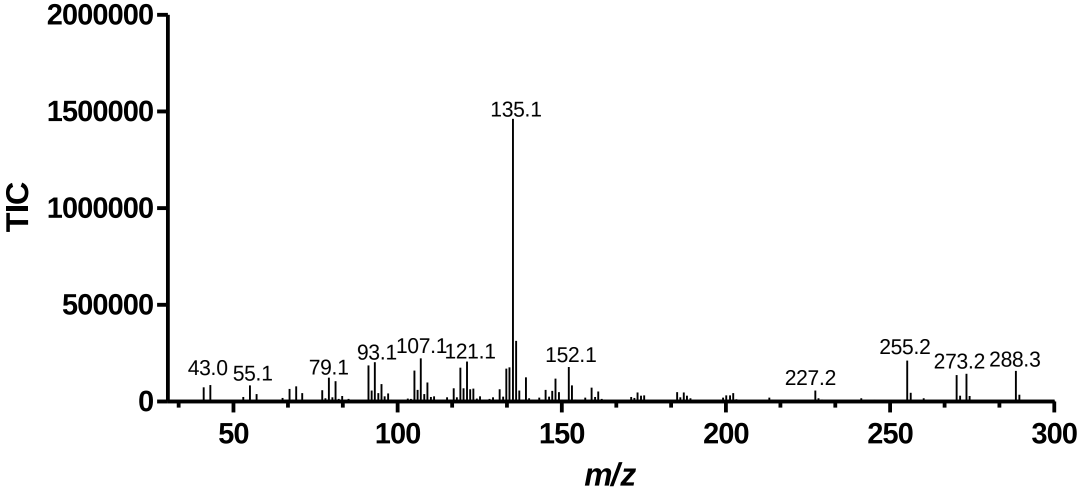

Figure S209 EI mass spectrum of **12a**.

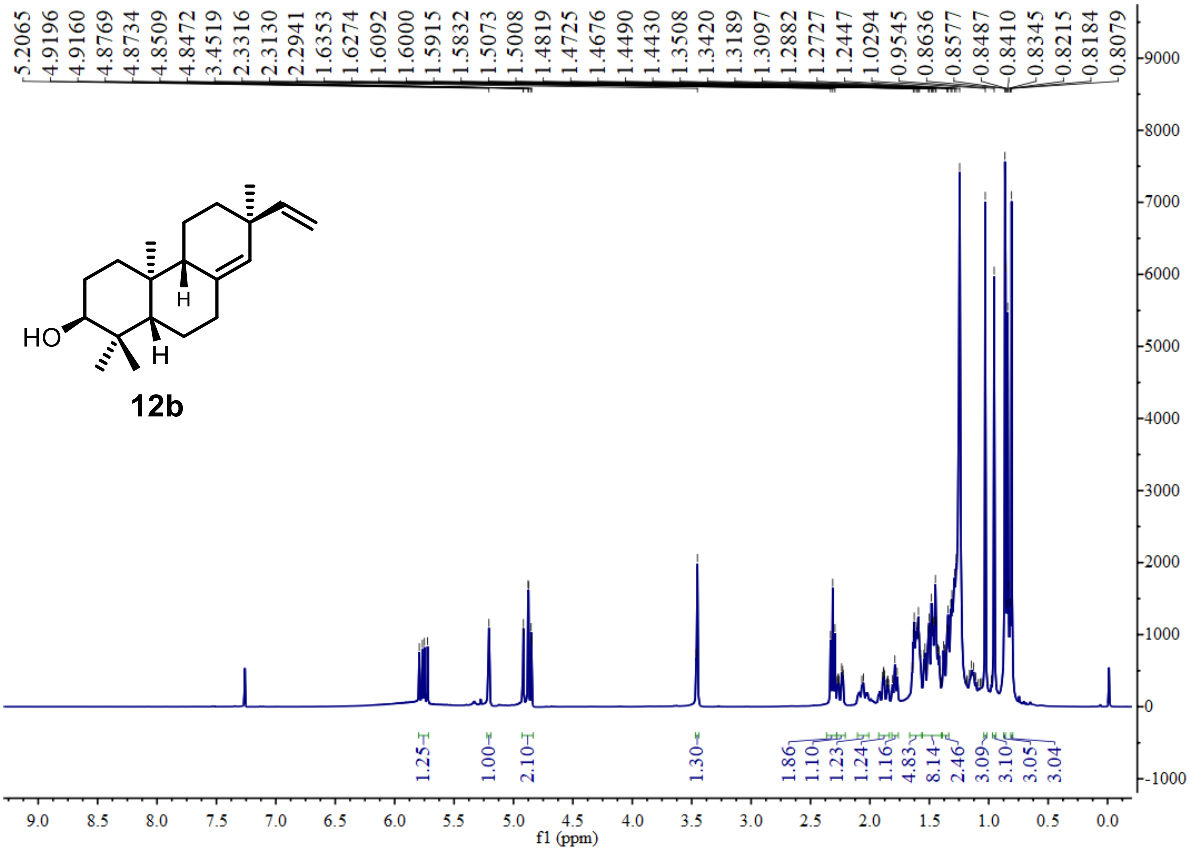

Figure S210 ^{1}H NMR spectrum of **12b** in $CDCl_3$ (400 MHz).

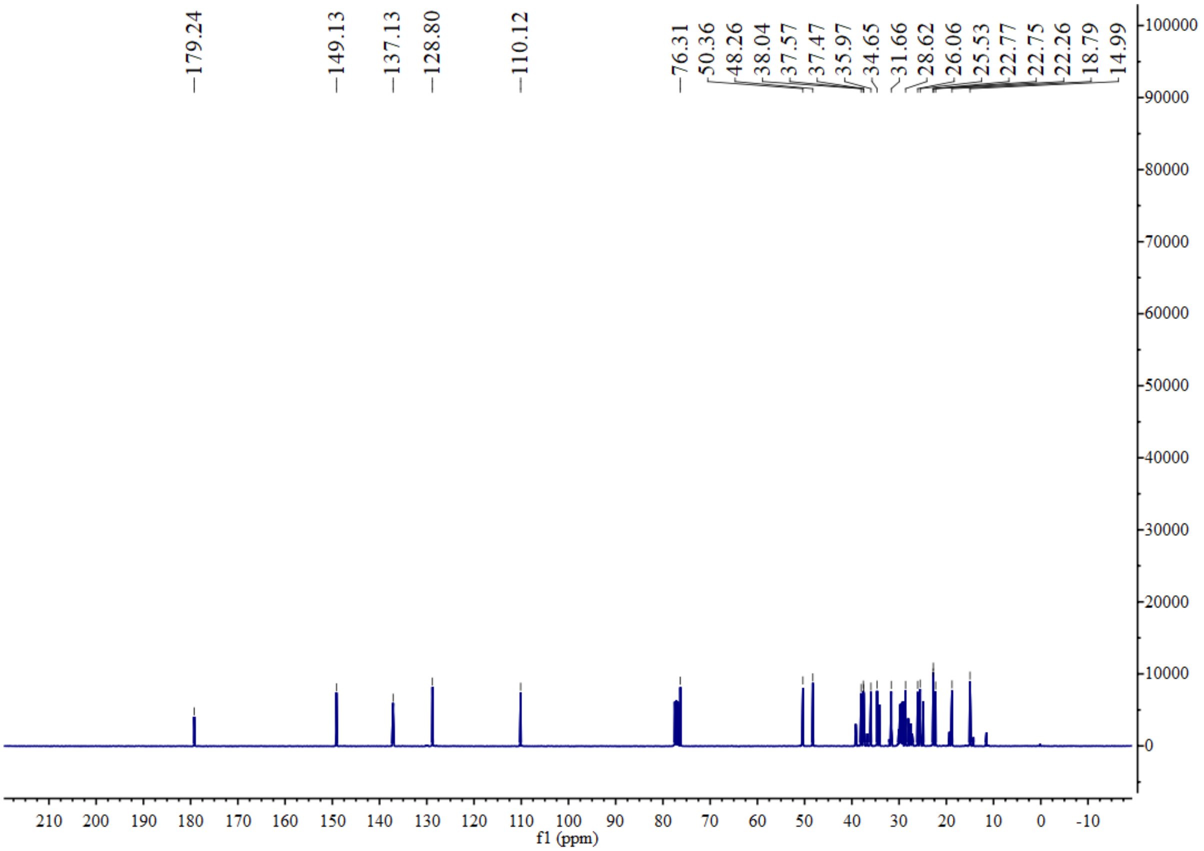

Figure S211 ^{13}C NMR spectrum of **12b** in $CDCl_3$ (100 MHz).

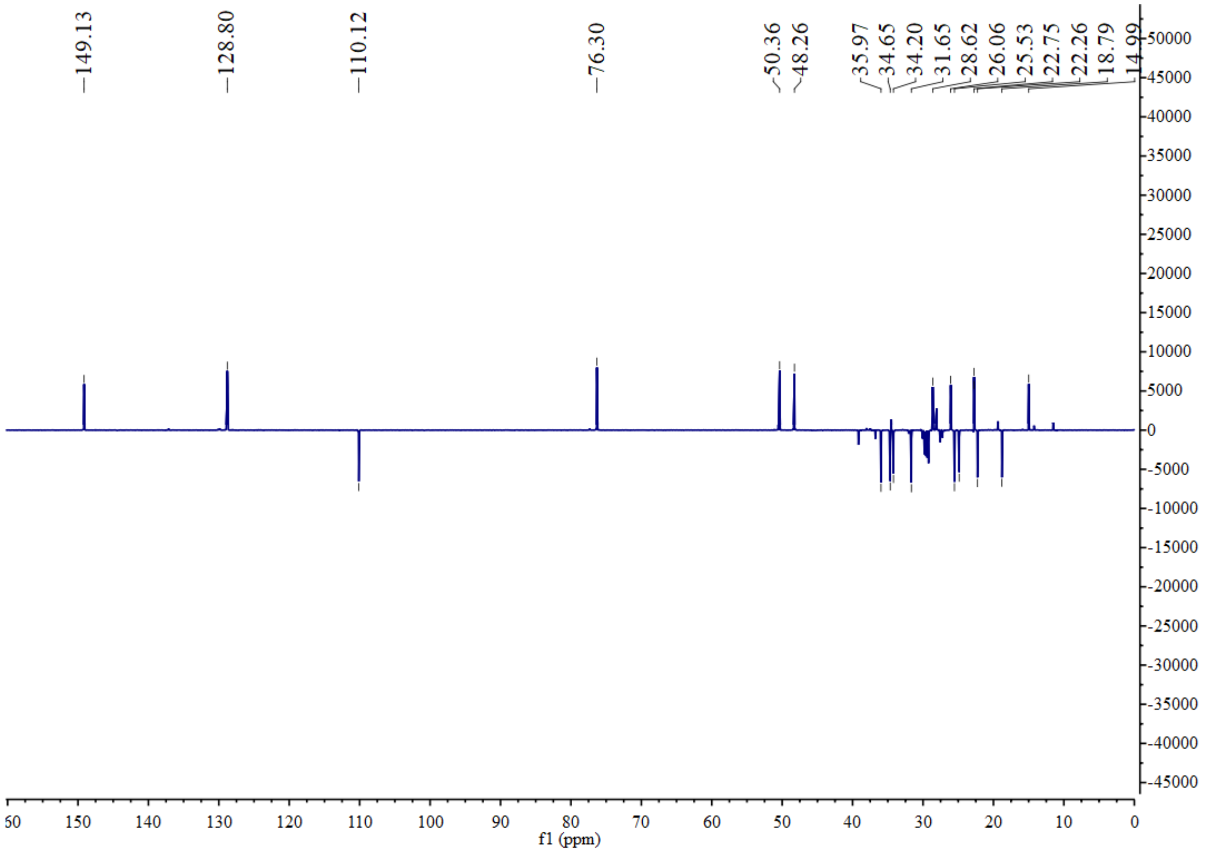

Figure S212 DEPT-135 NMR spectrum of **12b** in $CDCl_3$ (100 MHz).

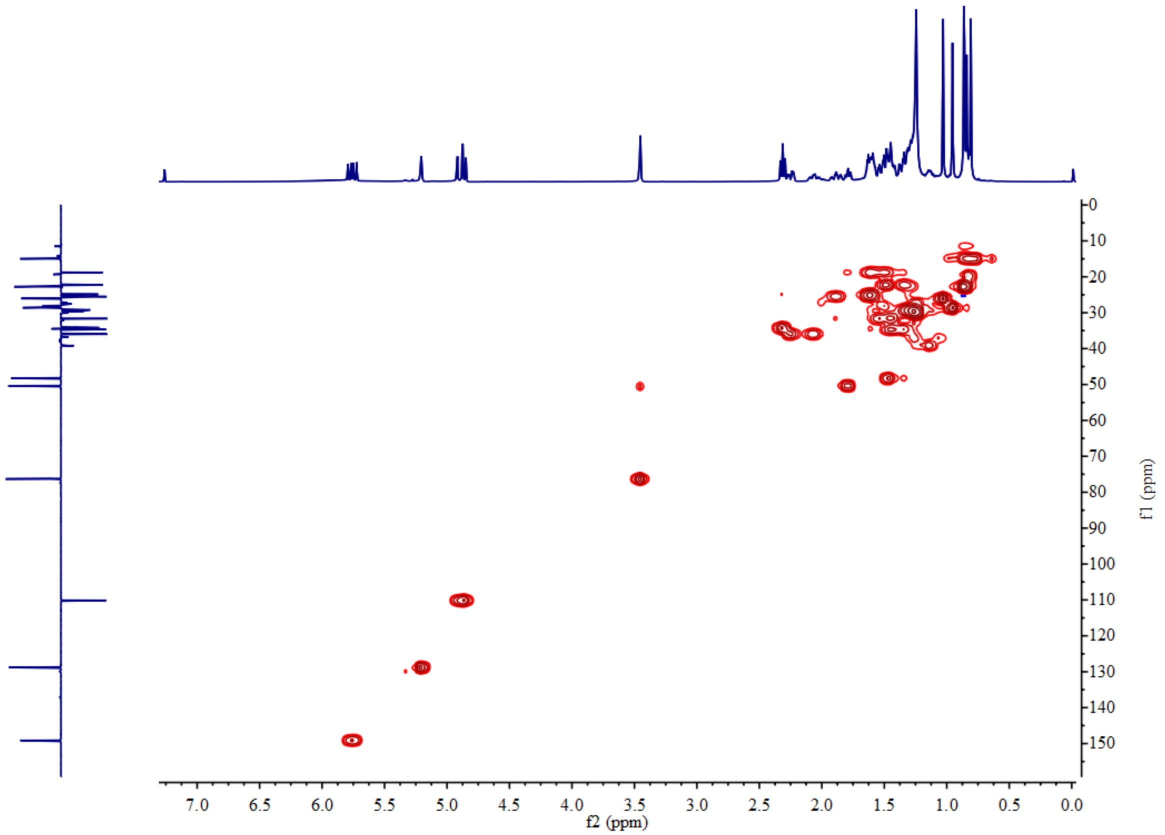

Figure S213 HSQC spectrum of **12b** in $CDCl_3$ (400 MHz).

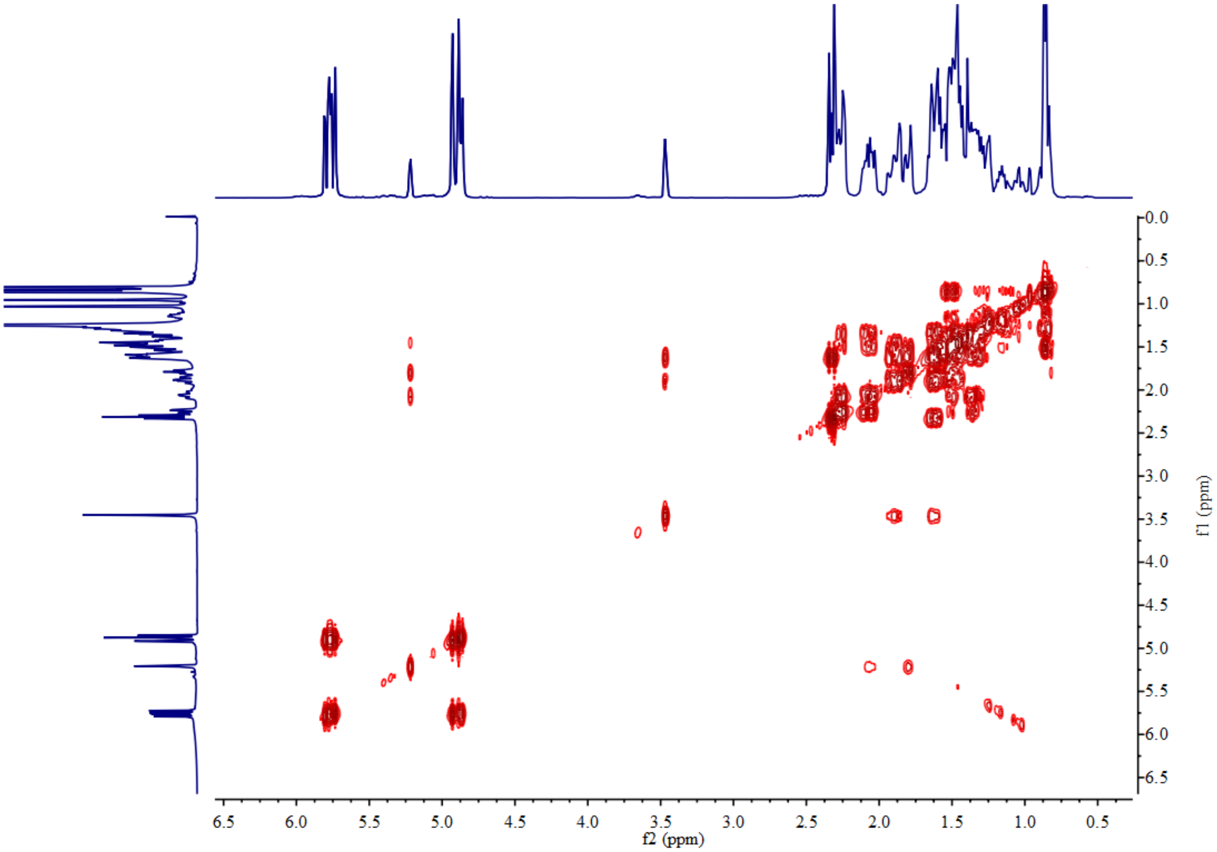

Figure S214 ^{1}H-^{1}H COSY spectrum of **12b** in $CDCl_3$ (400 MHz).

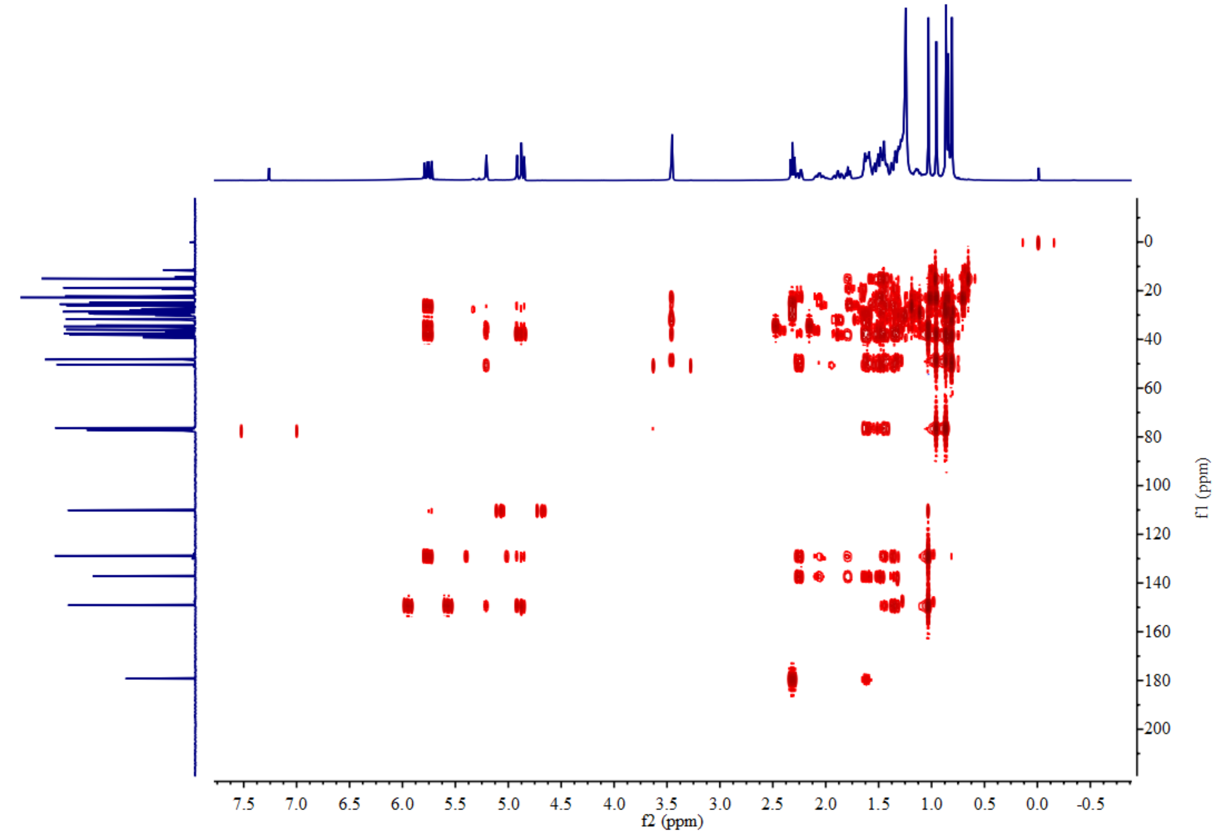

Figure S215 HMBC spectrum of **12b** in $CDCl_3$ (400 MHz).

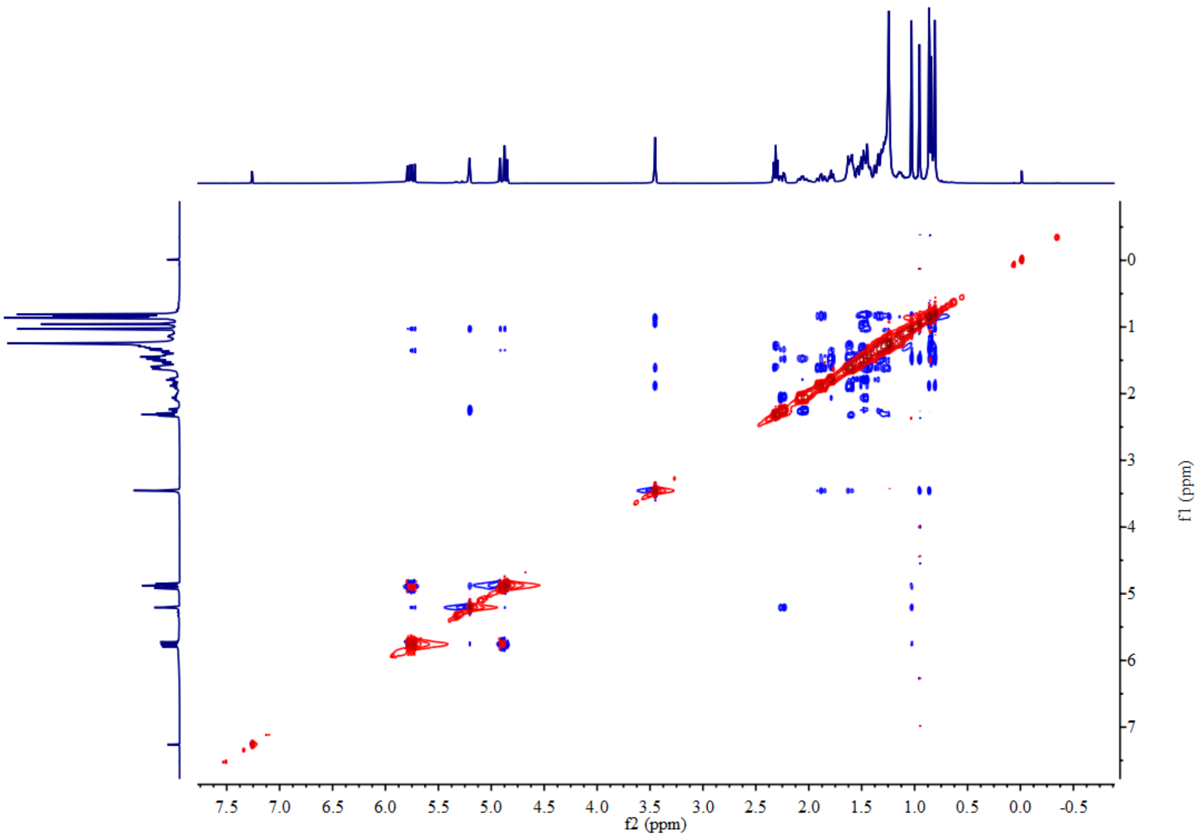

Figure S216 NOESY spectrum of **12b** in $CDCl_3$ (400 MHz).

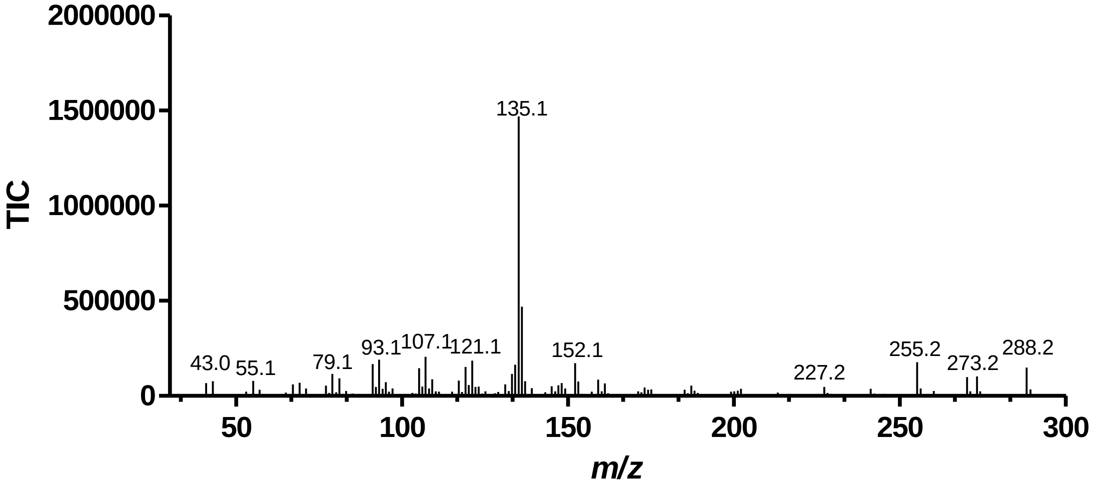

Figure S217 EI mass spectrum of **12b**.

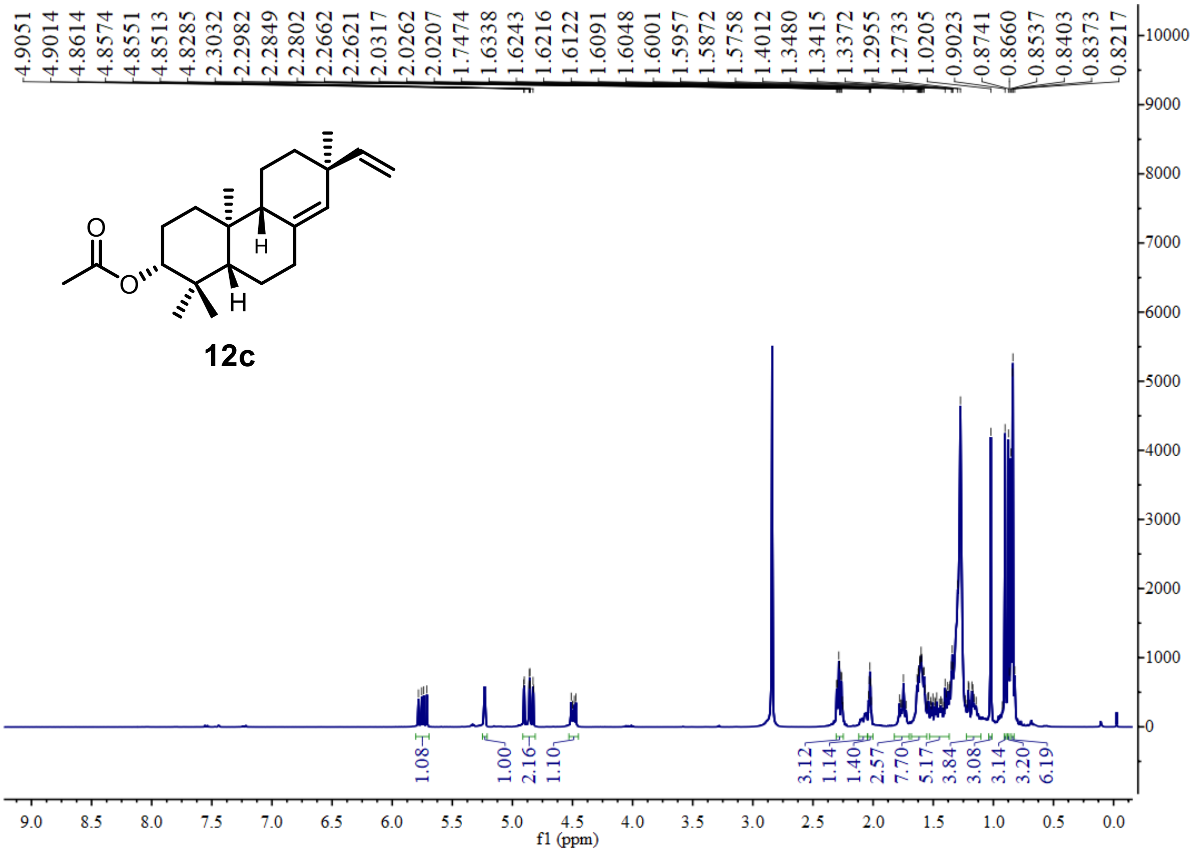

Figure S218 ^{1}H NMR spectrum of **12c** in acetone-d_6 (400 MHz).

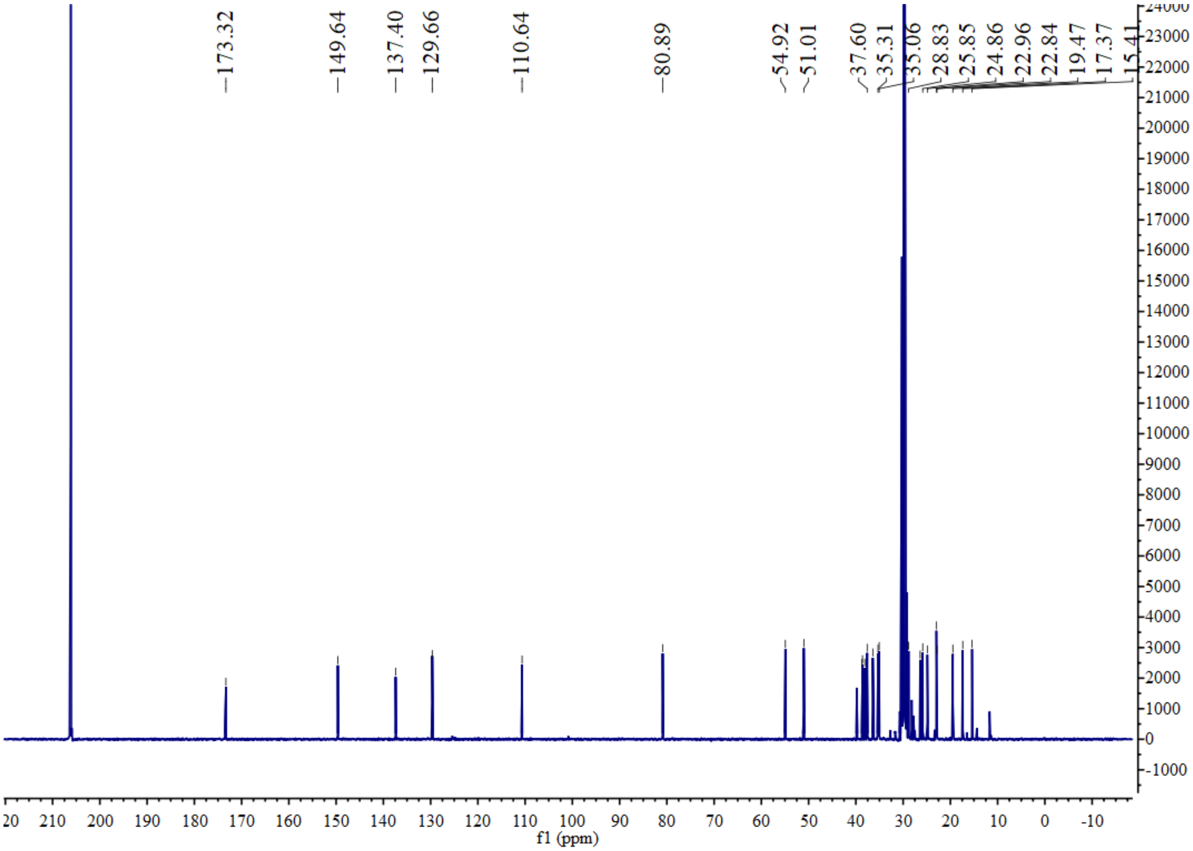

Figure S219 ^{13}C NMR spectrum of **12c** in acetone-d_6 (100 MHz).

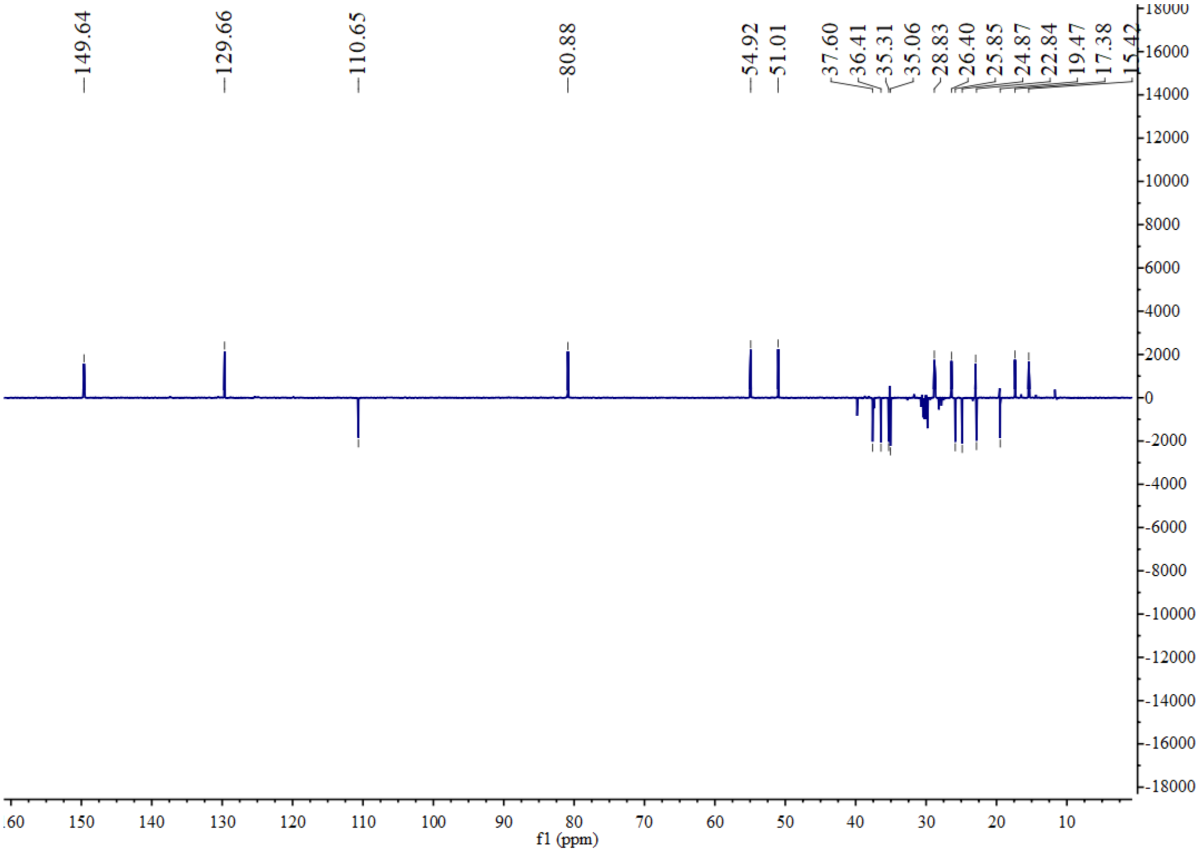

Figure S220 DEPT-135 NMR spectrum of **12c** in acetone-d_6 (100 MHz).

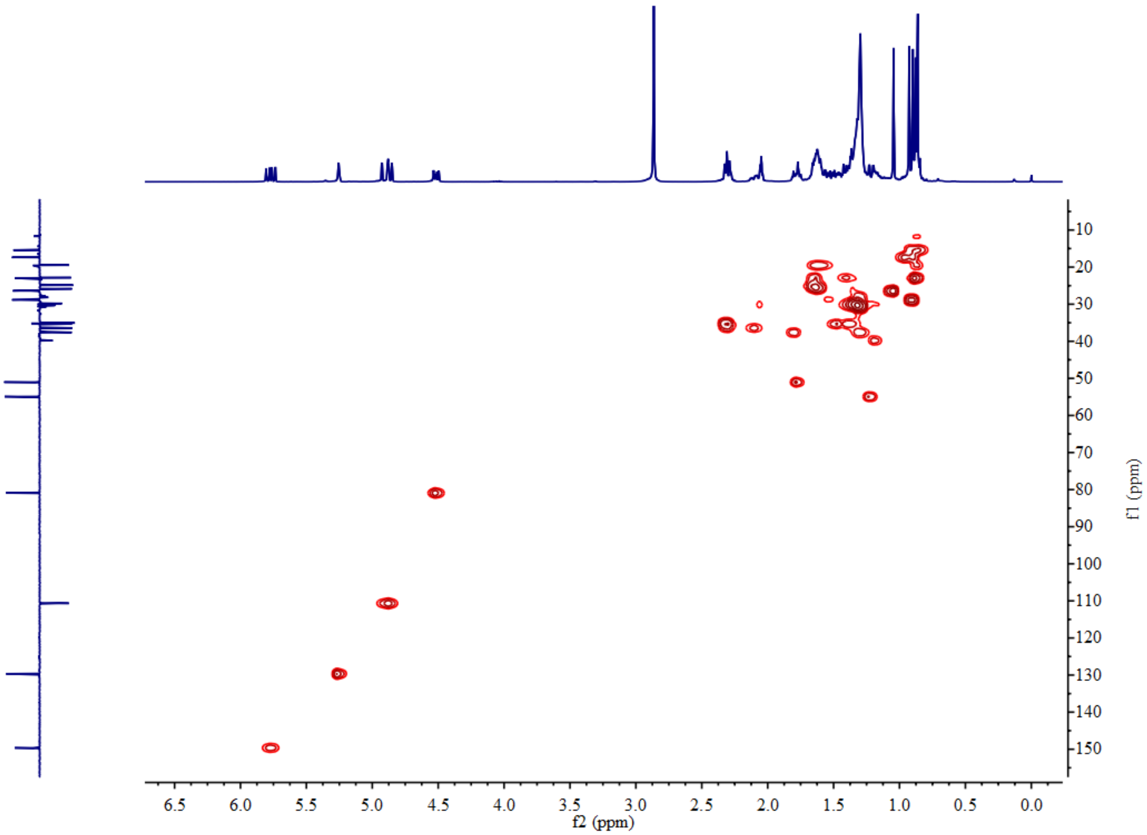

Figure S221 HSQC spectrum of **12**c in acetone-d_6 (400 MHz).

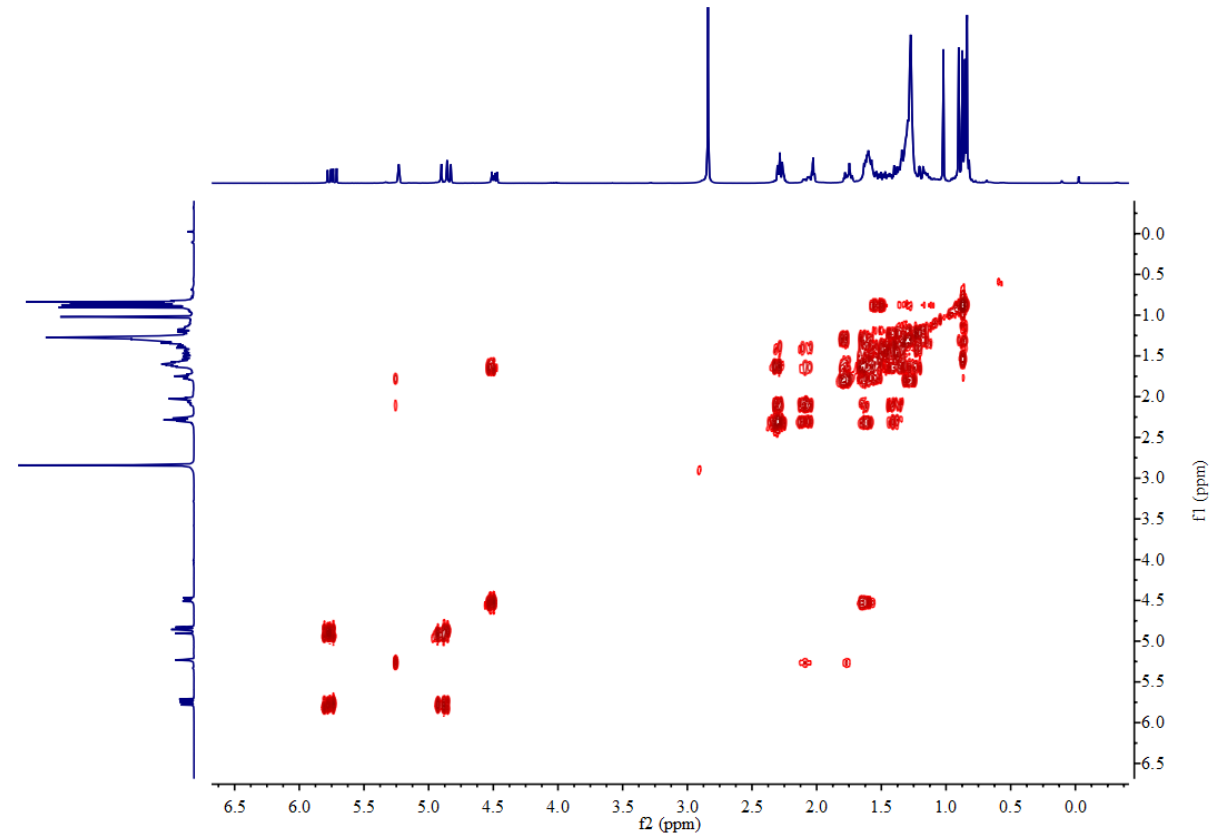

Figure S222 ^{1}H-^{1}H COSY spectrum of **12c** in acetone-d_6 (400 MHz).

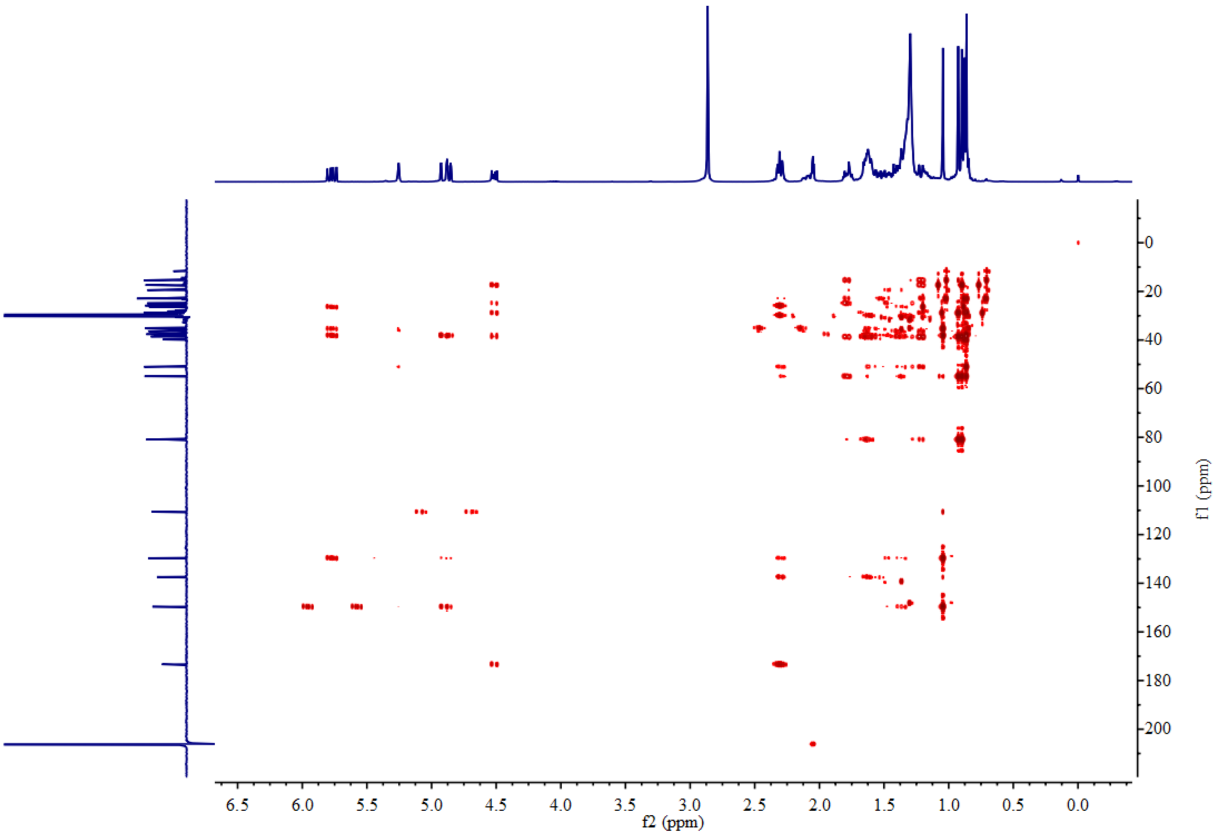

Figure S223 HMBC spectrum of **12c** in acetone-d_6 (400 MHz).

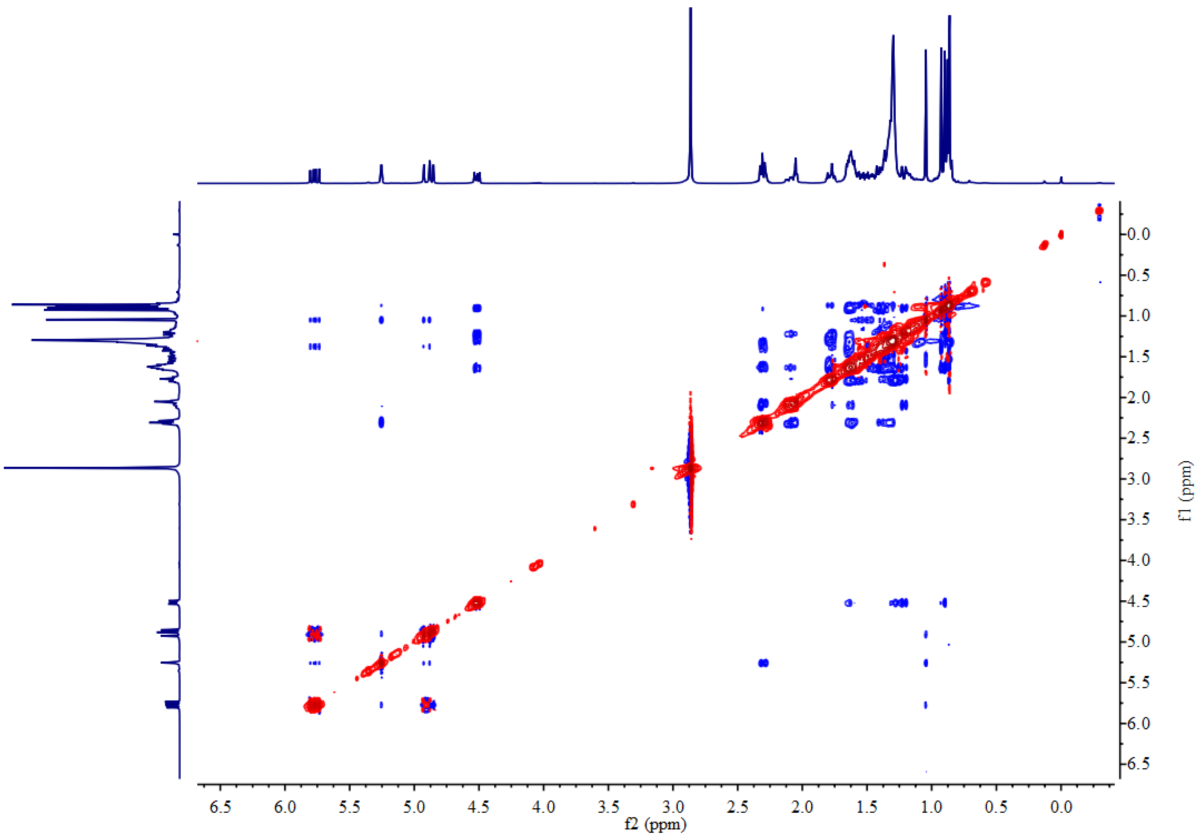

Figure S224 NOESY spectrum of **12c** in acetone-d_6 (400 MHz).

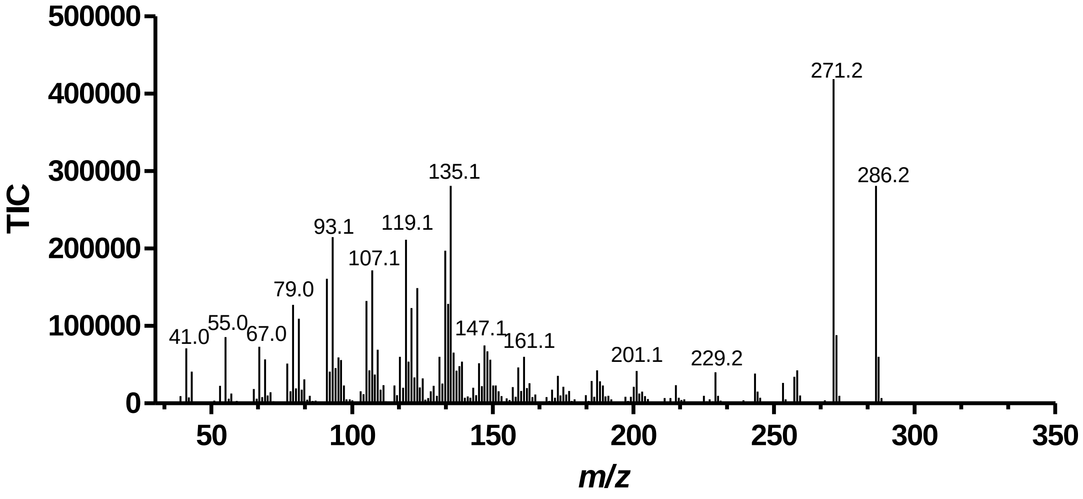

Figure S225 EI mass spectrum of **12c**.

References

(1) Nikodinovic, J., Barrow, K. D., Chuck, J. A. (2003) High yield preparation of genomic DNA from *Streptomyces*. *Bio-Techniques 35*, 932–936.

(2) Sekurova, O. N., Brautaset, T., Sletta, H., Borgos, S. E., Jakobsen M, Ø. M., Ellingsen, T. E., Strøm, A. R., Valla, S., Zotchev, S. B. (2004) *In vivo* analysis of the regulatory genes in the nystatin biosynthetic gene cluster of *Streptomyces noursei* ATCC 11455 reveals their differential control over antibiotic biosynthesis. *J. bacteriol. 186*, 1345–1354.

(3) Hopwood, D. A., Bibb, M. J., Chater, K. F., Kieser, T., Bruton, C. J., Kieser, H. M., Lydiate, D. J., Smith, C. P., Ward, J. M., Schrempf, H., (1985) Genetic manipulation of *Streptomyces*-A laboratory manual, *Cold Spring Harbor Laboratory Press*, New York.

(4) Gerlt J. A. (2017) Genomic enzymology: web tools for leveraging protein family sequence-function space and genome context to discover novel functions. *Biochemistry 56*, 4293–4308.

(5) Ikeda, H., Shin-Ya, K., Nagamitsu, T., Tomoda, H. (2016) Biosynthesis of mercapturic acid derivative of the labdane-type diterpene, cyslabdan that potentiates imipenem activity against methicillin-resistant *Staphylococcus aureus*: cys-labdan is generated by mycothiol-mediated xenobiotic detoxification. *J. Ind. Microbiol. Biotechnol. 43*, 325–342.

(6) Yamada, Y., Komatsu, M., Ikeda, H. (2016) Chemical diversity of labdane-type bicyclic diterpene biosynthesis in Actinomycetales microorganisms. *J. Antibiot(Tokyo). 69*, 515–523.

(7) Rabe, P., Samborskyy, M., Leadlay, P. F., Dickschat, J. S. (2017) Isoafricanol synthase from *Streptomyces malaysiensis*. *Org. Biomol. Chem. 15*, 2353–2358.

(8) Komatsu, M., Tsuda, M., Omura, S., Oikawa, H., Ikeda, H. (2008) Identification and functional analysis of genes controlling biosynthesis of 2-methylisoborneol. *Proc. Natl. Acad. Sci. U. S. A. 105*, 7422–7427.

(9) Dickschat J. S. (2016) Bacterial terpene cyclases. *Nat. Prod. Rep. 33*, 87–110.

(10) Brock, N. L., Ravella, S. R., Schulz, S., Dickschat, J. S. (2013). A detailed view of 2-methylisoborneol biosynthesis. *Angew. Chem. Int. Ed. Eng. 52*, 2100–2104.

(11) Duell, E. R., D'Agostino, P. M., Shapiro, N., Woyke, T., Fuchs, T. M., Gulder, T. (2019) Direct pathway cloning of the sodorifen biosynthetic gene cluster and recombinant generation of its product in *E. coli*. *Microb. Cell Fact. 18*, 32.

(12) Li, Z., Jiang, Y., Zhang, X., Chang, Y., Li, S., Zhang, X., Zheng, S., Geng, C., Men, P., Ma, L., Yang, Y., Gao, Z., Tang, Y., Li, S., (2020) Fragrant venezuelaenes A and B with a 5−5−6−7 tetracyclic skeleton: discovery, biosynthesis, and mechanisms of central catalysts. *ACS Catal. 10*, 5846–5851.

(13) Klapschinski, T. A., Rabe, P., Dickschat, J. S. (2016) Pristinol, a sesquiterpene alcohol with an unusual skeleton from *Streptomyces pristinaespiralis*. *Angew. Chem. Int. Ed. Eng. 55*, 10141–10144.

(14) Hayashi, Y., Matsuura, N., Toshima, H., Itoh, N., Ishikawa, J., Mikami, Y., Dairi, T. (2008) Cloning of the gene cluster responsible for the biosynthesis of brasilicardin A, a unique diterpenoid. *J. Antibiot. 61*, 164–174.

(15) Nakano, C., Hoshino, T. (2009) Characterization of the Rv3377c gene product, a type-B diterpene cyclase, from the *Mycobacterium tuberculosis* H37 genome. *Chembiochem 10*, 2060–2071.

(16) Rabe, P., Rinkel, J., Dolja, E., Schmitz, T., Nubbemeyer, B., Luu, T. H., Dickschat, J. S. (2017) Mechanistic investigations of two bacterial diterpene cyclases: spiroviolene synthase and tsukubadiene synthase. *Angew. Chem. Int. Ed. Eng. 56*, 2776–2779.

(17) Morrone, D., Chambers, J., Lowry, L., Kim, G., Anterola, A., Bender, K., Peters, R. J. (2009) Gibberellin biosynthesis in bacteria: separate *ent*-copalyl diphosphate and *ent*-kaurene synthases in *Bradyrhizobium japonicum*. *FEBS Lett. 583*, 475–480.

(18) Ikeda, C., Hayashi, Y., Itoh, N., Seto, H., Dairi, T. (2007) Functional analysis of eubacterial ent-copalyl diphosphate synthase and pimara-9(11),15-diene synthase with unique primary sequences. *J. Biochem. 141*, 37–45.

(19) Ikeda, H., Ishikawa, J., Hanamoto, A., Shinose, M., Kikuchi, H., Shiba, T., Sakaki, Y., Hattori, M., & Omura, S. (2003). Complete genome sequence and comparative analysis of the industrial microorganism *Streptomyces avermitilis*. *Nat. Biotechnol. 21*, 526–531.

(20) Hamano, Y., Kuzuyama, T., Itoh, N., Furihata, K., Seto, H., Dairi, T. (2002) Functional analysis of eubacterial diterpene cyclases responsible for biosynthesis of a diterpene antibiotic, terpentecin. *J. Biol. Chem. 277*, 37098–37104.

(21) Jiang, J., He, X., Cane, D. E. (2006) Geosmin biosynthesis. *Streptomyces coelicolor* germacradienol/germacrene D synthase converts farnesyl diphosphate to geosmin. *J. Am. Chem. Soc. 128*, 8128–8129.

(22) Hou, A., Dickschat, J. S. (2020) On the mass spectrometric fragmentations of the bacterial sesterterpenes sestermobaraenes A-C. *Beilstein J. Org. Chem. 16*, 2807–2819.

(23) Rabe, P., Rinkel, J., Klapschinski, T. A., Barra, L., Dickschat, J. S. (2016) A method for investigating the stereochemical course of terpene cyclisations. *Org. Biomol. Chem. 14*, 158–164.

(24) Rinkel, J., Steiner, S. T., Dickschat, J. S. (2019) Diterpene biosynthesis in actinomycetes: studies on cattleyene synthase and phomopsene synthase. *Angew. Chem. Int. Ed. 58*, 9230–9233.

(25) Yang, Y. L., Zhang, S., Ma, K., Xu, Y., Tao, Q., Chen, Y., Chen, J., Guo, S., Ren, J., Wang, W., Tao, Y., Yin, W. B., Liu, H. (2017) Discovery and characterization of a new family of diterpene cyclases in bacteria and fungi. *Angew. Chem. Int. Ed. Eng. 56*, 4749–4752.

(26) Rinkel, J., Lauterbach, L., Rabe, P., Dickschat, J. S. (2018) Two diterpene synthases for spiroalbatene and cembrene A from *Allokutzneria albata*. *Angew. Chem. Int. Ed. Eng. 57*, 3238–3241.

(27) Lauterbach, L., Rinkel, J., Dickschat, J. S. (2018) Two Bacterial Diterpene Synthases from *Allokutzneria albata* Produce Bonnadiene, Phomopsene, and Allokutznerene. *Angew. Chem. Int. Ed. Eng. 57*, 8280–8283.

(28) Rinkel, J., Dickschat, J. S. (2019). Mechanistic investigations on multiproduct β-himachalene synthase from *Cryptosporangium arvum*. *Beilstein J. Org. Chem. 15*, 1008–1019.

(29) Rinkel, J., Dickschat, J. S. (2019). Stereochemical investigations on the biosynthesis of achiral (*Z*)-γ-bisabolene in *Cryptosporangium arvum*. *Beilstein J. Org. Chem. 15*, 789–794.

(30) Lauterbach, L., Goldfuss, B., Dickschat, J. S. (2020) Two Diterpene Synthases from *Chryseobacterium*: Chryseodiene Synthase and Wanjudiene Synthase. *Angew. Chem. Int. Ed. Eng. 59*, 11943–11947.

(31) Rinkel, J., Dickschat, J. S. (2019) Characterization of Micromonocyclol Synthase from the Marine Actinomycete *Micromonospora marina*. *Org. Lett. 21*, 9442–9445.

(32) Rinkel, J., Lauterbach, L., Dickschat, J. S. (2017) Spata-13,17-diene Synthase - An enzyme with sesqui-, di-, and sesterterpene synthase activity from *Streptomyces xinghaiensis*. *Angew. Chem. Int. Ed. Eng. 56*, 16385–16389.

(33) Zhu, C., Xu, B., Adpressa, D. A., Rudolf, J. D., Loesgen, S. (2021). Discovery and Biosynthesis of a Structurally Dynamic Antibacterial Diterpenoid. *Angew. Chem. Int. Ed. Eng. 60*, 14163–14170.

(34) Tesso, H., König, W. A., Kubeczka, K. H., Bartnik, M., Glowniak, K. (2005) Secondary metabolites of *Peucedanum tauricum* fruits. *Phytochemistry*, *66*, 707–713.

(35) Fürstner, A., Schlecker, A. (2008) A gold-catalyzed entry into the sesquisabinene and sesquithujene families of terpenoids and formal total syntheses of cedrene and cedrol. *Chemistry 14*, 9181–9191.

(36) Kato, T., Suzuki, M., Kobayashi, T., Moore, B.P. (1980) Synthesis and pheromone activities of optically active neocembrenes and their geometrical isomers, (*E, Z, E*) - and (*E, E, Z*) neocembrenes. *J. Org. Chem. 45*, 1126-1130.

(37) Kenmoku, H., Tanaka, M., Ogiyama, K., Kato, N., Sassa, T. (2004) Identification of (+)-phyllocladene, (-)-sandaracopimaradiene, and (+)-kaurene as new fungal metabolites from fusicoccin-producing *Phomopsis amygdali* F6. *Biosci. Biotechno. Biochem. 68*, 1574–1577.

(38) Yajima, A., Yamaguchi, A., Nukada, T., Yabuta, G. (2007) Asymmetric total synthesis of *ent*-sandaracopimaradiene, a biosynthetic intermediate of oryzalexins. *Biosci. Biotechno. Biochem. 71*, 2822–2829.

(39) Wang, Q., Hillwig, M. L., Wu, Y., Peters, R. J. (2012) CYP701A8: a rice *ent*-kaurene oxidase paralog diverted to more specialized diterpenoid metabolism. *Plant physiol. 158*, 1418–1425.

(40) Mori, K., Waku M. (1985) Synthesis of oryzalexins a, b and c, the diterpenoidal phytoalexins isolated from rice blast leaves infected with *pyricularia oryzae*. *Tetrahedron 41*, 5653–5660.
